# Supplementary material for: Economic and social impacts of COVID-19 and public health measures: results from an anonymous online survey in Thailand, Malaysia, the UK, Italy and Slovenia
Source: BMJ Open. 2021 Jul 20;11(7):e046863. doi: 10.1136/bmjopen-2020-046863 (PMC8295020; doi:10.1136/bmjopen-2020-046863)
Supplement: Supplementary data [file bmjopen-2020-046863supp001.pdf]

Supplementary tables for "Economic and social impacts of COVID-19 and public health measures: results from an anonymous online survey in Thailand, Malaysia, the United Kingdom, Italy and Slovenia"

Anne Osterrieder[1,2], Giulia Cuman[3], Wirichada Pan-ngum[1,4], Phaik Kin Cheah[5], Phee-Kheng Cheah[6], Pimnara Peerawaranun[1], Margherita Silan[7], Miha Orazem[8,9], Ksenija Perkovic[10], Urh Groselj[8,11], Mira Leonie Schneiders[1,2,12], Tassawan Poomchaichote[1,13], Naomi Waithira[1,2], Supa-at Asarath[1], Bhensri Naemiratch[1], Supanat Ruangkajorn[1], Lenart Skof[14], Natinee Kulpijit[1], Constance R.S. Mackworth-Young[15], Darlene Ongkili[16], Rita Chanviriyavuth[1], Mavuto Mukaka[1,2], Phaik Yeong Cheah[1,2,12,,13]

[1]Mahidol Oxford Tropical Medicine Research Unit, Faculty of Tropical Medicine, Mahidol University, Bangkok, 10400, Thailand
[2]Centre for Tropical Medicine & Global Health, Nuffield Department of Medicine, University of Oxford, Oxford, UK
[3]Paediatric Ethics Committee; Research Ethics Committee, University Hospital of Padua, Padua, Italy
[4]Department of Tropical Hygiene, Faculty of Tropical Medicine, Mahidol University, Bangkok, 10400, Thailand
[5]Faculty of Arts and Social Science, Universiti Tunku Abdul Rahman, Kampar, Malaysia
[6]Emergency and Trauma Department, Sabah Women and Children's Hospital, Ministry of Health Malaysia, Kota Kinabalu, Malaysia
[7]Department of Statistical Sciences, University of Padua, Padua, Italy
[8]Faculty of Medicine, University of Ljubljana, Ljubljana, Slovenia
[9]Department of Radiation Oncology, Institute of Oncology Ljubljana, Ljubljana, Slovenia
[10]Institute for Social Studies, Science and Research Centre Koper, Koper, Slovenia
[11]Department of Endocrinology, Diabetes and Metabolic Diseases, University Children's Hospital, University Medical Center, University Children's Hospital Ljubljana, Ljubljana, Slovenia
[12]Ethox Centre, Nuffield Department of Population Health, University of Oxford, Oxford, UK
[13]The SoNAR-Global Network
[14]Institute for Philosophical Studies, Science and Research Centre Koper, Koper, Slovenia
[15]Department of Global Health and Development, London School of Hygiene and Tropical Medicine, London, UK
[16]Emergency and Trauma Department, Queen Elizabeth Hospital, Ministry of Health Malaysia, Kota Kinabalu, Malaysia

Corresponding author: Phaik Yeong Cheah, 420/6 Mahidol-Oxford Tropical Medicine 24 Research Unit, Faculty of Tropical Medicine, Rajvithi Road, Bangkok, Thailand, 10400. Email: phaikyeong@tropmedres.ac

Notes

- There are a total of 37 tables in this document. Suppl. Table 1 reports the distribution of the basic demographic variables in the respondent sample (N= number of respondents), followed by unweighted percentages (unweighted %) in brackets. The values displayed in the cells in Suppl. Tables 2-37 show the number of respondents (N) who replied 'yes' to the respective survey categories, followed by weighted percentages (weighted %) in brackets.
- Because of rounding to the nearest integer, percentages do not always add up to 100% exactly.
- For gender, due to small number in the "other/prefer not to say" category, p-values are presented for comparison between the male and female groups only.

Osterrieder A, *et al. BMJ Open* 2021; 11:e046863. doi: 10.1136/bmjopen-2020-046863

Contents

Supplementary tables for "Economic and social impacts of COVID-19 and public health measures: results from an anonymous online survey in Thailand, Malaysia, the United Kingdom, Italy and Slovenia" 1

Suppl. Table 1 Distribution of respondents by demographic characteristics and country (unweighted data) 4

Suppl. Table 2 Breakdown of economic impacts of COVID-19 and concerns by country 5

Suppl. Table 3 Breakdown of economic impacts of COVID-19 and concerns by country and gender 6

Suppl. Table 4 Breakdown of economic impacts of COVID-19 and concerns by country and education level 7

Suppl. Table 5 Breakdown of economic impacts of COVID-19 and concerns by country and age group 8

Suppl. Table 6 Breakdown of economic impacts of COVID-19 and concerns by country and household size 10

Suppl. Table 7 Breakdown of economic impacts of COVID-19 and concerns by country and whether or not living with children under 18 11

Suppl. Table 8 Breakdown of economic impacts of COVID-19 and concerns by country and type of income 12

Suppl. Table 9 Breakdown of concerns if advised/not allowed physical contact by country 13

Suppl. Table 10 Breakdown of concerns if advised/not allowed physical contact by country and gender 14

Suppl. Table 11 Breakdown of concerns if advised/not allowed physical contact by country and age group 15

Suppl. Table 12 Breakdown of concerns if advised/not allowed physical contact by country and education level 16

Suppl. Table 13 Breakdown of concerns if advised/not allowed physical contact by country and household size 17

Suppl. Table 14 Breakdown of concerns if advised/not allowed physical contact by country and whether or not living with children under 18 17

Suppl. Table 15 Breakdown of concerns if advised/not allowed physical contact by country and income type 19

Suppl. Table 16 Breakdown of maximum number of days that people thought they could cope by country 20

Suppl. Table 17 Breakdown of maximum number of days that people thought they could cope by country and gender 21

Suppl. Table 18 Breakdown of maximum number of days that people thought they could cope by country and age group 22

Suppl. Table 19 Breakdown of maximum number of days that people thought they could cope by country and household size 23

Suppl. Table 20 Breakdown of maximum number of days that people thought they could cope by country and whether or not living with children under 18 24

Suppl. Table 21 Breakdown of maximum number of days that people thought they could cope by country and education level 25

Suppl. Table 22 Breakdown of maximum number of days that people thought they could cope by country and type of income 26

Suppl. Table 23 Breakdown of behavioural changes and acceptance of government public health measures by country 27

Suppl. Table 24 Breakdown of behavioural changes and acceptance of government public health measures by country and gender 28

Suppl. Table 25 Breakdown of behavioural changes and acceptance of government public health measures by country and education level 30

Suppl. Table 26 Breakdown of behavioural changes and acceptance of government public health measures by age group 31

Suppl. Table 27 Breakdown of behavioural changes and acceptance of government public health measures by self-reported level of understanding of COVID-19 33
Suppl. Table 28 Breakdown of self-reported level of understanding of COVID-19 by country 35
Suppl. Table 29 Breakdown of self-reported level of understanding of COVID-19 by demographic characteristics 36
Suppl. Table 30 Breakdown of self-reported understanding of public health measures by self-reported level of understanding of COVID-19 37
Suppl. Table 31 What were the three most common ways people received communication on COVID-19, and what are the three most preferred ways to receive COVID-19 communications? Breakdown by country 38
Suppl. Table 32 What were the three most common ways people received communications on COVID-19, and what are the three most preferred ways to receive COVID-19 communications? Breakdown by country and gender 39
Suppl. Table 33 What were the three most common ways people received communications on COVID-19, and what are the three most preferred ways to receive COVID-19 communications? Breakdown by country and age group 40
Suppl. Table 34 What were the three most common ways people received communications on COVID-19, and what are the three most preferred ways to receive COVID-19 communications? Breakdown by country and education level 41
Suppl. Table 35 Most prevalent topic areas with unclear or conflicting COVID-19 information, and most prevalent 'fake news', breakdown by country 42
Suppl. Table 36 Most prevalent topic areas with unclear or conflicting COVID-19 information, and most prevalent 'fake news', breakdown by country and education level 43
Suppl. Table 37 Most prevalent topic areas with unclear or conflicting COVID-19 information, and most prevalent 'fake news', breakdown by country and self-reported level of understanding of COVID-19 44

Suppl. Table 1 Distribution of respondents by demographic characteristics and country (unweighted data)

Variable and categories	Thailand (N=1,476, 29%)	Malaysia (N=827, 16%)	UK (N=1,009, 20%)	Italy (N=712, 14%)	Slovenia (N=1,034, 20%)	Total (N=5,058)
**Gender**						
Male	704 (48)	298 (36)	426 (42)	222 (31)	366 (35)	2,016 (40)
Female	766 (52)	525 (63)	572 (57)	490 (69)	662 (64)	3,015 (60)
Other/prefer not to say	6 (0)	4 (0)	11 (1)	0 (0)	6 (1)	27 (1)
**Age (years)**						
18-24	83 (6)	139 (17)	54 (5)	75 (11)	62 (6)	413 (8)
25-34	140 (9)	211 (26)	86 (9)	197 (28)	246 (24)	880 (17)
35-64	1,152 (78)	442 (53)	616 (61)	383 (54)	676 (65)	3,269 (65)
65+	101 (7)	35 (4)	253 (25)	57 (8)	50 (5)	496 (10)
**Education level**						
Primary or lower/ secondary	909 (62)	82 (10)	247 (24)	217 (30)	202 (20)	1,657 (33)
Tertiary	567 (38)	745 (90)	762 (76)	495 (70)	832 (80)	3,401 (67)
**Household structure**						
Living alone	134 (9)	74 (9)	206 (20)	106 (15)	97 (9)	617 (12)
Living only with partner/spouse	173 (12)	95 (11)	391 (39)	192 (27)	210 (20)	1,061 (21)
Living with partner/spouse and children; living as single parent with children	847 (57)	312 (38)	260 (26)	188 (26)	518 (50)	2,125 (42)
Living with other relatives/non-relatives/other	322 (22)	346 (42)	152 (15)	226 (32)	209 (20)	1,255 (25)
**Household size**						
1	107 (7)	68 (8)	222 (22)	106 (15)	128 (12)	631 (12)
2	171 (12)	121 (15)	439 (44)	230 (32)	220 (21)	1,181 (23)
3-4	760 (51)	305 (37)	300 (30)	323 (45)	479 (46)	2,167 (43)
≥5	438 (30)	333 (40)	48 (5)	53 (7)	207 (20)	1,079 (21)
**Type of income**						
Fixed salary/benefits/pension	546 (37)	524 (63)	705 (70)	347 (49)	847 (82)	2,969 (59)
Contract and freelance	849 (58)	158 (19)	227 (22)	244 (34)	103 (10)	1,581 (31)
Other/no income	81 (5)	145 (18)	77 (8)	121 (17)	84 (8)	508 (10)
**Living with children under 18**	664 (45)	346 (42)	186 (18)	144 (20)	497 (48)	1,837 (36)
**Living with vulnerable group***	457 (31)	230 (28)	367 (36)	151 (21)	280 (27)	1,485 (29)
**Healthcare provider/worker****	239 (16)	213 (26)	118 (12)	64 (9)	341 (33)	975 (19)

Values in cells are n (%)
* Persons aged 70 or older; pregnant woman; people with serious health conditions
** Included respondents who were not working before COVID-19

Osterrieder A, *et al. BMJ Open* 2021; 11:e046863. doi: 10.1136/bmjopen-2020-046863

Suppl. Table 2 Breakdown of economic impacts of COVID-19 and concerns by country

Values in cells are n (weighted %) of respondents who replied 'yes'.

Variable and categories	Thailand	Malaysia	UK	Italy	Slovenia	Total	P-value
**If you were working before COVID-19, has COVID-19 created any inconvenience for you?**	N=1,255	N=613	N=630	N=526	N=929	N=3,953	
Loss of earnings	(N=1,248) 1,012 (85)	(N=556) 155 (40)	(N=584) 226 (44)	(N=496) 260 (55)	(N=867) 219 (30)	(N=3,751) 1,872 (56)	<0.001
Loss of job	(N=1,191) 233 (23)	(N=532) 44 (16)	(N=551) 51 (10)	(N=471) 59 (13)	(N=832) 15 (3)	(N=3,577) 402 (14)	<0.001
Reduction of working hours	(N=1,210) 492 (42)	(N=546) 228 (52)	(N=570) 201 (39)	(N=484) 233 (48)	(N=862) 319 (41)	(N=3,672) 1,473 (44)	0.107
Closure of workplace	(N=1,207) 425 (36)	(N=562) 289 (53)	(N=591) 296 (51)	(N=484) 167 (39)	(N=833) 63 (8)	(N=3,677) 1,240 (36)	<0.001
**Did you continue to work during COVID-19?**	(N=1,255) 1,019 (79)	(N=613) 532 (70)	(N=630) 460 (70)	(N=526) 388 (67)	(N=929) 768 (79)	(N=3,953) 3,167 (75)	0.011
**What are/were your concerns if advised no physical contact/not allowed to go out/allowed to go out only for essential needs?**	N=1,476	N=827	N=1,009	N=712	N=1,034	N=5,058	
Financial (e.g. loss of income, loss of job)	(N=1,466) 1,215 (86)	(N=775) 419 (60)	(N=950) 271 (32)	(N=678) 315 (41)	(N=1,015) 302 (28)	(N=4,884) 2,522 (53)	<0.001
Professional/ career progression	(N=1,414) 607 (42)	(N=759) 418 (52)	(N=942) 198 (24)	(N=670) 224 (22)	(N=1,001) 219 (17)	(N=4,786) 1,666 (32)	<0.001

Suppl. Table 3 Breakdown of economic impacts of COVID-19 and concerns by country and gender

M = male; F = female; O = other/prefer not to say. Values in cells are n (weighted %) of respondents who replied 'yes'.

**Variable and categories**	**Thailand**			**Malaysia**			**UK**			**Italy**			**Slovenia**			**Total**			
**Gender**	**M**	**F**	**O**	**M**	**F**	**O**	**M**	**F**	**O**	**M**	**F**	**O**	**M**	**F**	**O**	**M**	**F**	**O**	**P-value (for total M vs F)**
**If you were working before COVID-19, has COVID-19 created any inconvenience for you?**	N=606	N=645	N=4	N=230	N=380	N=3	N=261	N=363	N=6	N=184	N=342	N=0	N=332	N=591	N=6	N=1,613	N=2,321	N=19	
Loss of earnings	(N=604) 508 (83)	(N=640) 502 (86)	(N=4) 2 (50)	(N=210) 75 (42)	(N=343) 80 (37)	(N=3) 0 (0)	(N=245) 97 (45)	(N=333) 128 (43)	(N=6) 1 (17)	(N=177) 99 (54)	(N=319) 161 (57)		(N=314) 82 (29)	(N=548) 135 (31)	(N=5) 2 (40)	(N=1,550) 861 (55)	(N=2,183) 1,006 (57)	(N=18) 5 (28)	0.531
Loss of job	(N=576) 104 (20)	(N=611) 129 (25)	(N=4) 0 (0)	(N=202) 17 (18)	(N=327) 27 (15)	(N=3) 0 (0)	(N=233) 21 (19)	(N=313) 30 (11)	(N=5) 0 (0)	(N=168) 19 (10)	(N=303) 40 (17)		(N=301) 3 (1)	(N=526) 12 (4)	(N=5) 0 (0)	(N=1,480) 164 (13)	(N=2,080) 238 (16)	(N=17) 0 (0)	0.157
Reduction of working hours	(N=586) 225 (41)	(N=620) 265 (43)	(N=4) 2 (50)	(N=205) 85 (57)	(N=338) 141 (46)	(N=3) 2 (67)	(N=240) 90 (41)	(N=324) 107 (37)	(N=6) 4 (67)	(N=174) 94 (52)	(N=310) 139 (43)		(N=315) 128 (44)	(N=541) 188 (39)	(N=6) 3 (50)	(N=1,520) 622 (45)	(N=2,133) 840 (42)	(N=19) 11 (58)	0.179
Closure of workplace	(N=581) 194 (35)	(N=622) 231 (37)	(N=4) 0 (0)	(N=208) 109 (48)	(N=351) 178 (60)	(N=3) 2 (67)	(N=251) 124 (50)	(N=334) 169 (51)	(N=6) 3 (50)	(N=172) 65 (38)	(N=312) 102 (41)		(N=302) 19 (7)	(N=526) 43 (9)	(N=5) 1 (20)	(N=1,514) 511 (35)	(N=2,145) 723 (37)	(N=18) 6 (33)	0.365
Did you continue to work during COVID-19?	(N=606) 508 (84)	(N=645) 507 (75)	(N=4) 4 (100)	(N=230) 198 (67)	(N=380) 332 (73)	(N=3) 2 (67)	(N=261) 198 (72)	(N=363) 258 (67)	(N=6) 4 (67)	(N=184) 144 (74)	(N=342) 244 (60)		(N=332) 295 (85)	(N=591) 469 (74)	(N=6) 4 (67)	(N=1,613) 1,343 (78)	(N=2,321) 1,810 (71)	(N=19) 14 (74)	0.010
**What are/were your concerns if advised no physical contact/not allowed to go out/allowed to go out only for essential needs?**	N=704	N=766	N=6	N=298	N=525	N=4	N=261	N=363	N=6	N=222	N=490	N=0	N=366	N=662	N=6	N=2,016	N=3,015	N=27	
Financial	(N=700) 592 (85)	(N=760) 619 (86)	(N=6) 4 (67)	(N=279) 155 (62)	(N=492) 261 (59)	(N=4) 3 (75)	(N=411) 113 (34)	(N=529) 154 (31)	(N=10) 4 (40)	(N=214) 113 (44)	(N=464) 202 (38)		(N=361) 110 (27)	(N=648) 188 (29)	(N=6) 4 (67)	(N=1,965) 1,083 (54)	(N=2,893) 1,424 (53)	(N=26) 15 (58)	0.806
Professional/ career progression	(N=675) 278 (41)	(N=733) 326 (42)	(N=6) 3 (50)	(N=270) 137 (53)	(N=485) 279 (51)	(N=4) 2 (50)	(N=409) 84 (26)	(N=523) 108 (22)	(N=10) 6 (60)	(N=211) 92 (26)	(N=459) 132 (18)		(N=354) 77 (14)	(N=641) 141 (19)	(N=6) 1 (17)	(N=1,919) 668 (32)	(N=2,841) 986 (31)	(N=26) 12 (46)	0.597

Osterrieder A, *et al. BMJ Open* 2021; 11:e046863. doi: 10.1136/bmjopen-2020-046863

Suppl. Table 4 Breakdown of economic impacts of COVID-19 and concerns by country and education level

P/S = primary or lower/secondary education; T = tertiary education. Values in cells are n (weighted %) of respondents who replied 'yes'.

**Variable and categories**	**Thailand**		**Malaysia**		**UK**		**Italy**		**Slovenia**		**Total**		
**Education level**	**P/S**	**T**	**P/S**	**T**	**P/S**	**T**	**P/S**	**T**	**P/S**	**T**	**P/S**	**T**	**P-value (for total)**
**If you were working before COVID-19, has COVID-19 created any inconvenience for you?**	N=785	N=470	N=53	N=560	N=122	N=508	N=136	N=390	N=160	N=769	N=1,256	N=2,697	
Loss of earnings	(N=780)<br>725 (90)	(N=468)<br>287 (62)	(N=50)<br>21 (42)	(N=506)<br>134 (28)	(N=116)<br>55 (58)	(N=468)<br>171 (34)	(N=126)<br>75 (58)	(N=370)<br>185 (52)	(N=150)<br>56 (36)	(N=717)<br>163 (24)	(N=1,222)<br>932 (65)	(N=2,529)<br>940 (38)	<0.001
Loss of job	(N=744)<br>164 (24)	(N=447)<br>69 (16)	(N=50)<br>9 (19)	(N=482)<br>35 (7)	(N=108)<br>12 (13)	(N=443)<br>39 (9)	(N=123)<br>18 (14)	(N=348)<br>41 (12)	(N=140)<br>7 (4)	(N=692)<br>8 (1)	(N=1,165)<br>210 (17)	(N=2,412)<br>192 (8)	<0.001
Reduction of working hours	(N=762)<br>332 (43)	(N=448)<br>160 (37)	(N=48)<br>25 (55)	(N=498)<br>203 (40)	(N=110)<br>42 (49)	(N=460)<br>159 (32)	(N=125)<br>63 (47)	(N=359)<br>170 (49)	(N=144)<br>72 (46)	(N=718)<br>247 (35)	(N=1,189)<br>534 (47)	(N=2,483)<br>939 (37)	<0.001
Closure of workplace	(N=753)<br>262 (36)	(N=454)<br>163 (37)	(N=48)<br>28 (55)	(N=514)<br>261 (49)	(N=116)<br>51 (48)	(N=475)<br>245 (52)	(N=130)<br>59 (44)	(N=354)<br>108 (31)	(N=137)<br>14 (8)	(N=696)<br>49 (7)	(N=1,184)<br>414 (37)	(N=2,493)<br>826 (34)	0.180
Did you continue to work during COVID-19?	(N=785)<br>613 (78)	(N=470)<br>406 (86)	(N=53)<br>34 (65)	(N=560)<br>498 (90)	(N=122)<br>73 (59)	(N=508)<br>387 (77)	(N=136)<br>75 (59)	(N=390)<br>313 (79)	(N=160)<br>115 (74)	(N=769)<br>653 (85)	(N=1,256)<br>910 (71)	(N=2,697)<br>2,257 (83)	<0.001
**What are/were your concerns if advised no physical contact/not allowed to go out/allowed to go out only for essential needs?**	N=909	N=567	N=82	N=745	N=247	N=762	N=217	N=495	N=202	N=832	N=1,657	N=3,401	
Financial	(N=904)<br>828 (89)	(N=562)<br>387 (68)	(N=75)<br>46 (62)	(N=700)<br>373 (55)	(N=232)<br>64 (34)	(N=718)<br>207 (31)	(N=205)<br>96 (39)	(N=473)<br>219 (46)	(N=193)<br>71 (29)	(N=822)<br>231 (27)	(N=1,609)<br>1,105 (59)	(N=3,275)<br>1,417 (41)	<0.001
Professional/ career progression	(N=865)<br>326 (39)	(N=549)<br>281 (54)	(N=72)<br>36 (50)	(N=687)<br>382 (59)	(N=228)<br>21 (16)	(N=714)<br>177 (31)	(N=198)<br>42 (15)	(N=472)<br>182 (37)	(N=192)<br>37 (13)	(N=809)<br>182 (22)	(N=1,555)<br>462 (30)	(N=3,231)<br>1,204 (36)	0.004

Suppl. Table 5 Breakdown of economic impacts of COVID-19 and concerns by country and age group

Values in cells are n (weighted %) of respondents who replied 'yes'.

Suppl. Table 5a Breakdown of economic impacts of COVID-19 and concerns by country and age group

Values in cells are n (weighted %) of respondents who replied 'yes'.

**Variable and categories**	**Thailand**				**Malaysia**				**UK**			
**Age group**	**18-24**	**25-34**	**35-64**	**65+**	**18-24**	**25-34**	**35-64**	**65+**	**18-24**	**25-34**	**35-64**	**65+**
**If you were working before COVID-19, has COVID-19 created any inconvenience for you?**	N=35	N=120	N=1,027	N=73	N=43	N=176	N=378	N=16	N=34	N=70	N=466	N=60
Loss of earnings	(N=34) 28 (61)	(N=120) 75 (76)	(N=1,021) 851 (89)	(N=73) 58 (80)	(N=41) 15 (54)	(N=166) 33 (38)	(N=334) 98 (34)	(N=15) 9 (57)	(N=31) 16 (71)	(N=69) 16 (38)	(N=427) 168 (41)	(N=57) 26 (46)
Loss of job	(N=34) 15 (32)	(N=114) 21 (25)	(N=972) 183 (20)	(N=71) 14 (22)	(N=40) 10 (42)	(N=164) 12 (14)	(N=314) 20 (10)	(N=14) 2 (13)	(N=30) 5 (19)	(N=68) 5 (8)	(N=401) 35 (9)	(N=52) 6 (8)
Reduction of working hours	(N=34) 18 (42)	(N=113) 55 (54)	(N=991) 401 (42)	(N=72) 18 (23)	(N=38) 18 (44)	(N=168) 67 (75)	(N=325) 136 (49)	(N=15) 7 (50)	(N=32) 17 (74)	(N=68) 14 (27)	(N=416) 145 (36)	(N=54) 25 (45)
Closure of workplace	(N=34) 21 (60)	(N=117) 45 (42)	(N=984) 340 (35)	(N=72) 19 (24)	(N=40) 29 (65)	(N=167) 64 (51)	(N=340) 184 (48)	(N=15) 12 (83)	(N=32) 19 (75)	(N=68) 38 (52)	(N=434) 215 (49)	(N=57) 24 (44)
Did you continue to work during COVID-19?	(N=35) 19 (70)	(N=120) 101 (82)	(N=1,027) 838 (80)	(N=73) 61 (81)	(N=43) 32 (40)	(N=176) 163 (67)	(N=378) 330 (82)	(N=16) 7 (43)	(N=34) 19 (33)	(N=70) 60 (85)	(N=466) 346 (72)	(N=60) 35 (56)
**What are/were your concerns if advised no physical contact/not allowed to go out/allowed to go out only for essential needs?**	N=83	N=140	N=1,152	N=101	N=139	N=211	N=442	N=35	N=54	N=86	N=616	N=253
Financial	(N=81) 59 (69)	(N=139) 102 (84)	(N=1,145) 985 (89)	(N=101) 69 (78)	(N=134) 83 (51)	(N=204) 115 (82)	(N=408) 211 (64)	(N=29) 10 (42)	(N=52) 30 (62)	(N=82) 29 (37)	(N=581) 195 (35)	(N=235) 17 (6)
Professional/ career progression	(N=82) 58 (61)	(N=133) 68 (48)	(N=1,106) 452 (39)	(N=93) 29 (31)	(N=130) 96 (64)	(N=206) 142 (68)	(N=395) 173 (43)	(N=28) 7 (26)	(N=51) 40 (64)	(N=83) 36 (40)	(N=572) 118 (17)	(N=236) 4 (2)

Osterrieder A, *et al. BMJ Open* 2021; 11:e046863. doi: 10.1136/bmjopen-2020-046863

Suppl. Table 5b Breakdown of economic impacts of COVID-19 and concerns by country and <u>age group</u>

Values in cells are n (weighted %) of respondents who replied 'yes'.

**Variable and categories**	**Italy**				**Slovenia**				**Total**				
**Age group**	**18-24**	**25-34**	**35-64**	**65+**	**18-24**	**25-34**	**35-64**	**65+**	**18-24**	**25-34**	**35-64**	**65+**	**P-value (for total)**
**If you were working before COVID-19, has COVID-19 created any inconvenience for you?**	N=31	N=159	N=324	N=12	N=37	N=222	N=646	N=24	N=180	N=747	N=2,841	N=185	
Loss of earnings	(N=31) 24 (67)	(N=154) 73 (47)	(N=299) 155 (54)	(N=12) 8 (87)	(N=37) 15 (45)	(N=216) 52 (25)	(N=595) 144 (29)	(N=19) 8 (39)	(N=174) 98 (59)	(N=725) 249 (47)	(N=2,676) 1,416 (56)	(N=176) 109 (67)	0.044
Loss of job	(N=30) 4 (10)	(N=151) 18 (12)	(N=282) 35 (12)	(N=8) 2 (42)	(N=37) 2 (5)	(N=211) 4 (2)	(N=567) 9 (3)	(N=17) 0 (0)	(N=171) 36 (25)	(N=708) 60 (13)	(N=2,536) 282 (12)	(N=162) 24 (17)	0.053
Reduction of working hours	(N=30) 18 (58)	(N=152) 69 (47)	(N=292) 143 (50)	(N=10) 3 (16)	(N=36) 22 (67)	(N=213) 77 (40)	(N=593) 212 (39)	(N=20) 8 (38)	(N=170) 93 (55)	(N=714) 282 (51)	(N=2,617) 1,037 (43)	(N=171) 61 (31)	0.016
Closure of workplace	(N=31) 22 (66)	(N=154) 54 (43)	(N=289) 85 (32)	(N=10) 6 (86)	(N=36) 8 (25)	(N=210) 19 (12)	(N=570) 35 (6)	(N=17) 1 (3)	(N=173) 99 (59)	(N=716) 220 (40)	(N=2,617) 859 (32)	(N=171) 62 (35)	<0.001
Did you continue to work during COVID-19?	(N=31) 16 (66)	(N=159) 118 (71)	(N=324) 250 (70)	(N=12) 4 (13)	(N=37) 22 (56)	(N=222) 187 (83)	(N=646) 540 (81)	(N=24) 19 (72)	(N=180) 108 (54)	(N=747) 629 (78)	(N=2,841) 2,304 (78)	(N=185) 126 (68)	<0.001
**What are/were your concerns if advised no physical contact/not allowed to go out/allowed to go out only for essential needs?**	N=75	N=197	N=383	N=57	N=62	N=246	N=676	N=50	N=413	N=880	N=3,269	N=496	
Financial	(N=75) 36 (46)	(N=195) 102 (52)	(N=356) 168 (48)	(N=52) 9 (20)	(N=62) 26 (45)	(N=243) 66 (24)	(N=664) 205 (36)	(N=46) 5 (4)	(N=404) 234 (57)	(N=863) 414 (60)	(N=3,154) 1,764 (58)	(N=463) 110 (30)	<0.001
Professional/ career progression	(N=75) 25 (30)	(N=194) 97 (48)	(N=350) 99 (23)	(N=51) 3 (1)	(N=61) 28 (44)	(N=242) 80 (29)	(N=654) 109 (15)	(N=44) 2 (1)	(N=399) 247 (57)	(N=858) 423 (48)	(N=3,077) 951 (28)	(N=452) 45 (11)	<0.001

Suppl. Table 6 Breakdown of economic impacts of COVID-19 and concerns by country and household size

Values in cells are n (weighted %) of respondents who replied 'yes'.

Variable and categories	Thailand		Malaysia		UK		Italy		Slovenia		Total		
**Household size (number of persons in the household)**	**1-4**	**≥5**	**1-4**	**≥5**	**1-4**	**≥5**	**1-4**	**≥5**	**1-4**	**≥5**	**1-4**	**≥5**	**P-value (for total)**
**If you were working before COVID-19, has COVID-19 created any inconvenience for you?**	N=862	N=393	N=376	N=237	N=592	N=38	N=491	N=35	N=743	N=186	N=3,064	N=889	
Loss of earnings	(N=857) 685 (84)	(N=391) 327 (85)	(N=348) 97 (36)	(N=208) 58 (45)	(N=547) 213 (43)	(N=37) 13 (51)	(N=464) 243 (55)	(N=32) 17 (68)	(N=693) 181 (30)	(N=174) 38 (32)	(N=2,909) 1,419 (53)	(N=842) 453 (64)	0.003
Loss of job	(N=821) 150 (21)	(N=370) 83 (26)	(N=335) 22 (13)	(N=197) 22 (22)	(N=515) 49 (9)	(N=36) 2 (21)	(N=442) 59 (14)	(N=29) 0 (0)	(N=666) 13 (3)	(N=166) 2 (3)	(N=2,779) 293 (12)	(N=798) 109 (20)	0.005
Reduction of working hours	(N=835) 335 (41)	(N=375) 157 (44)	(N=346) 143 (50)	(N=200) 85 (56)	(N=532) 185 (37)	(N=38) 16 (59)	(N=454) 216 (47)	(N=30) 17 (68)	(N=686) 247 (39)	(N=176) 72 (50)	(N=2,853) 1,126 (42)	(N=819) 347 (49)	0.037
Closure of workplace	(N=832) 278 (34)	(N=375) 147 (40)	(N=349) 168 (45)	(N=213) 121 (67)	(N=553) 280 (51)	(N=38) 16 (42)	(N=451) 153 (38)	(N=33) 14 (55)	(N=666) 51 (8)	(N=167) 12 (8)	(N=2,851) 930 (34)	(N=826) 310 (41)	0.057
Did you continue to work during COVID-19?	(N=862) 703 (78)	(N=393) 316 (81)	(N=376) 330 (72)	(N=237) 202 (67)	(N=592) 430 (71)	(N=38) 30 (58)	(N=491) 363 (67)	(N=35) 25 (63)	(N=743) 612 (79)	(N=186) 156 (79)	(N=3,064) 2,438 (75)	(N=889) 729 (75)	0.873
**What are/were your concerns if advised no physical contact/not allowed to go out/allowed to go out only for essential needs?**	N=1,038	N=438	N=494	N=333	N=961	N=48	N=659	N=53	N=827	N=207	N=3,979	N=1,079	
Financial	(N=1,031) 860 (87)	(N=435) 355 (82)	(N=461) 234 (59)	(N=314) 185 (62)	(N=906) 258 (32)	(N=44) 13 (34)	(N=627) 285 (40)	(N=51) 30 (66)	(N=813) 249 (26)	(N=202) 53 (37)	(N=3,838) 1,886 (50)	(N=1,046) 636 (66)	<0.001
Professional/ career progression	(N=996) 411 (38)	(N=418) 196 (49)	(N=454) 228 (47)	(N=305) 190 (59)	(N=899) 187 (23)	(N=43) 11 (32)	(N=620) 200 (21)	(N=50) 24 (46)	(N=799) 180 (16)	(N=202) 39 (20)	(N=3,768) 1,206 (28)	(N=1,018) 460 (46)	<0.001

Osterrieder A, et al. BMJ Open 2021; 11:e046863. doi: 10.1136/bmjopen-2020-046863

Suppl. Table 7 Breakdown of economic impacts of COVID-19 and concerns by country and whether or not living with children under 18

Y = living with children under 18; N = not living with children under 18. Values in cells are n (weighted %) of respondents who replied 'yes'.

**Variable and categories**	**Thailand**		**Malaysia**		**UK**		**Italy**		**Slovenia**		**Total**		
**Living with children under 18**	**Y**	**N**	**Y**	**N**	**Y**	**N**	**Y**	**N**	**Y**	**N**	**Y**	**N**	**P-value (for total)**
**If you were working before COVID-19, has COVID-19 created any inconvenience for you?**	N=546	N=709	N=276	N=337	N=158	N=472	N=112	N=414	N=462	N=467	N=1,554	N=2,399	
Loss of earnings	(N=545) 483 (91)	(N=703) 529 (79)	(N=239) 66 (44)	(N=317) 89 (37)	(N=144) 52 (46)	(N=440) 174 (43)	(N=98) 58 (61)	(N=398) 202 (54)	(N=428) 100 (30)	(N=439) 119 (31)	(N=1,454) 759 (62)	(N=2,297) 1,113 (53)	0.005
Loss of job	(N=525) 121 (27)	(N=666) 112 (19)	(N=227) 20 (26)	(N=305) 24 (10)	(N=139) 10 (13)	(N=412) 41 (9)	(N=92) 12 (9)	(N=379) 47 (14)	(N=409) 6 (3)	(N=423) 9 (3)	(N=1,392) 169 (18)	(N=2,185) 233 (12)	0.008
Reduction of working hours	(N=531) 240 (47)	(N=679) 252 (38)	(N=230) 102 (55)	(N=316) 126 (50)	(N=145) 48 (38)	(N=425) 153 (39)	(N=99) 48 (52)	(N=385) 185 (49)	(N=427) 165 (45)	(N=435) 154 (38)	(N=1,432) 603 (47)	(N=2,240) 870 (41)	0.047
Closure of workplace	(N=528) 216 (43)	(N=679) 209 (30)	(N=247) 141 (66)	(N=315) 148 (44)	(N=151) 73 (46)	(N=440) 223 (52)	(N=96) 39 (44)	(N=388) 128 (38)	(N=413) 27 (7)	(N=420) 36 (9)	(N=1,435) 496 (38)	(N=2,242) 744 (35)	0.268
Did you continue to work during COVID-19?	(N=546) 412 (74)	(N=709) 607 (84)	(N=276) 242 (65)	(N=337) 290 (74)	(N=158) 124 (71)	(N=472) 336 (69)	(N=112) 85 (73)	(N=414) 303 (65)	(N=462) 386 (81)	(N=467) 382 (78)	(N=1,554) 1,249 (74)	(N=2,399) 1,918 (75)	0.655
**What are/were your concerns if advised no physical contact/not allowed to go out/allowed to go out only for essential needs?**	N=664	N=812	N=346	N=481	N=186	N=823	N=144	N=568	N=497	N=537	N=1,837	N=3,221	
Financial	(N=660) 594 (92)	(N=806) 621 (80)	(N=323) 194 (59)	(N=452) 225 (62)	(N=174) 59 (35)	(N=776) 212 (32)	(N=135) 76 (61)	(N=543) 239 (37)	(N=486) 139 (33)	(N=529) 163 (24)	(N=1,778) 1,062 (64)	(N=3,106) 1,460 (47)	<0.001
Professional/ career progression	(N=637) 230 (37)	(N=777) 377 (45)	(N=315) 182 (53)	(N=444) 236 (51)	(N=171) 58 (35)	(N=771) 140 (21)	(N=134) 46 (35)	(N=536) 178 (19)	(N=483) 98 (19)	(N=518) 121 (15)	(N=1,740) 614 (35)	(N=3,046) 1,052 (30)	0.033

Suppl. Table 8 Breakdown of economic impacts of COVID-19 and concerns by country and type of income

FBP = fixed salary, benefits/pension; CF = contract and freelance; O = other/no income. Values in cells are n (weighted %) of respondents who replied 'yes'.

**Variable and categories**	**Thailand**			**Malaysia**			**UK**			**Italy**			**Slovenia**			**Total**			
**Type of income**	**FBP**	**CF**	**O**	**FBP**	**CF**	**O**	**FBP**	**CF**	**O**	**FBP**	**CF**	**O**	**FBP**	**CF**	**O**	**FBP**	**CF**	**O**	**P-value (for total)**
**If you were working before COVID-19, has COVID-19 created any inconvenience for you?**	N=495	N=738	N=22	N=475	N=125	N=13	N=397	N=210	N=23	N=278	N=228	N=20	N=788	N=101	N=40	N=2,433	N=1,402	N=118	
Loss of earnings	(N=493) 320 (74)	(N=733) 674 (91)	(N=22) 18 (89)	(N=428) 69 (26)	(N=117) 79 (65)	(N=11) 7 (92)	(N=361) 91 (28)	(N=200) 125 (67)	(N=23) 10 (50)	(N=253) 87 (39)	(N=224) 157 (75)	(N=19) 16 (95)	(N=731) 128 (21)	(N=96) 70 (77)	(N=40) 21 (53)	(N=2,266) 695 (38)	(N=1,370) 1,105 (81)	(N=115) 72 (69)	<0.001
Loss of job	(N=478) 78 (21)	(N=692) 148 (23)	(N=21) 7 (47)	(N=420) 18 (8)	(N=101) 24 (31)	(N=11) 2 (78)	(N=350) 20 (6)	(N=179) 30 (17)	(N=22) 1 (6)	(N=247) 6 (3)	(N=206) 45 (27)	(N=18) 8 (36)	(N=709) 6 (2)	(N=83) 5 (6)	(N=40) 4 (10)	(N=2,204) 128 (8)	(N=1,261) 252 (22)	(N=112) 22 (27)	<0.001
Reduction of working hours	(N=479) 226 (52)	(N=710) 259 (36)	(N=21) 7 (45)	(N=429) 163 (51)	(N=106) 60 (56)	(N=11) 5 (12)	(N=358) 89 (24)	(N=189) 102 (60)	(N=23) 10 (48)	(N=256) 111 (45)	(N=210) 113 (56)	(N=18) 9 (26)	(N=735) 227 (33)	(N=89) 67 (81)	(N=38) 25 (70)	(N=2,257) 816 (41)	(N=1,304) 601 (47)	(N=111) 56 (49)	0.042
Closure of workplace	(N=480) 195 (44)	(N=706) 224 (30)	(N=21) 6 (43)	(N=438) 214 (52)	(N=113) 67 (54)	(N=11) 8 (89)	(N=376) 188 (47)	(N=192) 98 (56)	(N=23) 10 (51)	(N=252) 63 (27)	(N=213) 94 (54)	(N=19) 10 (68)	(N=710) 33 (5)	(N=85) 20 (20)	(N=38) 10 (23)	(N=2,256) 693 (33)	(N=1,309) 503 (40)	(N=112) 44 (46)	0.015
Did you continue to work during COVID-19?	(N=495) 418 (83)	(N=738) 584 (77)	(N=22) 17 (78)	(N=475) 437 (83)	(N=125) 86 (42)	(N=13) 9 (25)	(N=397) 319 (79)	(N=210) 126 (57)	(N=23) 15 (62)	(N=278) 234 (81)	(N=228) 146 (51)	(N=20) 8 (15)	(N=788) 682 (84)	(N=101) 63 (57)	(N=40) 23 (59)	(N=2,433) 2,090 (82)	(N=1,402) 1,005 (65)	(N=118) 72 (53)	<0.001
**What are/were your concerns if advised no physical contact/not allowed to go out/allowed to go out only for essential needs?**	N=546	N=849	N=81	N=524	N=158	N=145	N=705	N=227	N=77	N=347	N=244	N=121	N=847	N=103	N=84	N=2,969	N=1,581	N=508	
Financial	(N=543) 402 (81)	(N=843) 753 (89)	(N=80) 60 (76)	(N=488) 231 (58)	(N=149) 110 (83)	(N=138) 78 (39)	(N=658) 131 (22)	(N=219) 116 (56)	(N=73) 24 (34)	(N=324) 102 (30)	(N=238) 165 (66)	(N=116) 48 (43)	(N=830) 190 (23)	(N=102) 74 (61)	(N=83) 38 (40)	(N=2,843) 1,056 (40)	(N=1,551) 1,218 (79)	(N=490) 248 (46)	<0.001
Professional/ career progression	(N=530) 221 (43)	(N=804) 348 (41)	(N=80) 38 (37)	(N=481) 247 (41)	(N=142) 81 (71)	(N=136) 90 (56)	(N=657) 104 (17)	(N=212) 66 (36)	(N=73) 28 (40)	(N=319) 71 (15)	(N=235) 112 (38)	(N=116) 41 (22)	(N=821) 156 (14)	(N=97) 35 (23)	(N=83) 28 (33)	(N=2,808) 799 (24)	(N=1,490) 642 (43)	(N=488) 225 (40)	<0.001

12

Suppl. Table 9 Breakdown of concerns if advised/not allowed physical contact by country

Values in cells are n (weighted %) of respondents who replied 'yes'.

Variable and categories	Thailand	Malaysia	UK	Italy	Slovenia	Total	P-value (for total)
**What are/were your concerns if advised no physical contact/not allowed to go out/allowed to go out only for essential needs?**	N=1,476	N=827	N=1,009	N=712	N=1,034	N=5,058	
Caring responsibilities (e.g. childcare, caring for elderly parents, not having access to care)	(N=1,454) 890 (62)	(N=772) 456 (57)	(N=946) 325 (31)	(N=681) 312 (46)	(N=1,006) 423 (35)	(N=4,859) 2,406 (47)	<0.001
Physical health (e.g. not being able to attend doctor appointments, medication supply for illnesses, lack of exercise)	(N=1,457) 910 (61)	(N=782) 501 (66)	(N=961) 587 (61)	(N=687) 393 (63)	(N=1,007) 437 (45)	(N=4,894) 2,828 (59)	<0.001
Recreational (e.g. not being able to access recreational facilities like cinemas or restaurants, cancelled sports or cultural events)	(N=1,425) 580 (38)	(N=763) 407 (49)	(N=963) 571 (58)	(N=683) 352 (47)	(N=1,011) 636 (65)	(N=4,845) 2,546 (51)	<0.001
Sports (e.g. participating in competitive or professional sports activities)	(N=1,400) 546 (38)	(N=755) 302 (39)	(N=943) 214 (22)	(N=675) 174 (24)	(N=997) 331 (36)	(N=4,770) 1,567 (32)	<0.001
Mental health and wellbeing (e.g. boredom, loneliness, anxiety, depression)	(N=1,427) 798 (55)	(N=769) 476 (61)	(N=970) 699 (75)	(N=691) 448 (60)	(N=1,008) 436 (43)	(N=4,865) 2,857 (58)	<0.001
Living arrangements (e.g. not enough living space, passing on illness to family members, domestic abuse)	(N=1,419) 646 (45)	(N=753) 289 (46)	(N=943) 215 (24)	(N=674) 114 (16)	(N=999) 177 (15)	(N=4,788) 1,441 (31)	<0.001
Infrastructure (e.g. access to transport, network services, internet access)	(N=1,409) 651 (46)	(N=750) 308 (45)	(N=935) 212 (24)	(N=672) 163 (28)	(N=996) 195 (19)	(N=4,762) 1,529 (33)	<0.001
Social (e.g. not being able to see friends or attend social or family events)	(N=1,440) 768 (52)	(N=773) 474 (56)	(N=974) 768 (79)	(N=686) 525 (70)	(N=1,015) 725 (69)	(N=4,888) 3,260 (64)	<0.001
Religious and spiritual (e.g. not being able to go to church, mosque, temple etc.)	(N=1,433) 591 (42)	(N=769) 393 (58)	(N=942) 162 (17)	(N=670) 95 (18)	(N=998) 201 (19)	(N=4,812) 1,442 (31)	<0.001

Osterrieder A, *et al. BMJ Open* 2021; 11:e046863. doi: 10.1136/bmjopen-2020-046863

Suppl. Table 10 Breakdown of concerns if advised/not allowed physical contact by country and gender

M = male; F = female; O = other/prefer not to say. Values in cells are n (weighted %) of respondents who replied 'yes'.

**Variable and categories**	**Thailand**			**Malaysia**			**UK**			**Italy**			**Slovenia**			**Total**			
**Gender**	**M**	**F**	**O**	**M**	**F**	**O**	**M**	**F**	**O**	**M**	**F**	**O**	**M**	**F**	**O**	**M**	**F**	**O**	**P-value (for total M vs F)**
**What are/were your concerns if advised no physical contact/not allowed to go out/allowed to go out only for essential needs?**	N=704	N=766	N=6	N=298	N=525	N=4	N=426	N=572	N=11	N=222	N=490	N=0	N=366	N=662	N=6	N=2,016	N=3,015	N=27	
Caring responsibilities	(N=697) 430 (61)	(N=751) 456 (62)	(N=6) 4 (67)	(N=282) 170 (53)	(N=486) 284 (62)	(N=4) 2 (50)	(N=407) 124 (27)	(N=529) 197 (35)	(N=10) 4 (40)	(N=213) 82 (36)	(N=468) 230 (56)		(N=356) 124 (25)	(N=644) 297 (44)	(N=6) 2 (33)	(N=1,955) 930 (42)	(N=2,878) 1,464 (52)	(N=26) 12 (46)	<0.001
Physical health	(N=698) 443 (60)	(N=753) 463 (61)	(N=6) 4 (67)	(N=282) 184 (59)	(N=496) 314 (74)	(N=4) 3 (75)	(N=414) 255 (62)	(N=537) 323 (61)	(N=10) 9 (90)	(N=213) 106 (56)	(N=474) 287 (70)		(N=356) 148 (44)	(N=645) 287 (46)	(N=6) 2 (33)	(N=1,963) 1,136 (56)	(N=2,905) 1,674 (61)	(N=26) 18 (69)	0.058
Recreational	(N=681) 267 (39)	(N=738) 310 (38)	(N=6) 3 (50)	(N=275) 160 (54)	(N=484) 246 (44)	(N=4) 1 (25)	(N=411) 253 (61)	(N=542) 309 (56)	(N=10) 9 (90)	(N=215) 126 (54)	(N=468) 226 (41)		(N=359) 239 (71)	(N=646) 395 (59)	(N=6) 2 (33)	(N=1,941) 1,045 (54)	(N=2,878) 1,486 (47)	(N=26) 15 (58)	0.007
Sports	(N=670) 276 (40)	(N=724) 268 (35)	(N=6) 2 (33)	(N=275) 131 (47)	(N=476) 170 (29)	(N=4) 1 (25)	(N=410) 104 (23)	(N=524) 105 (21)	(N=9) 5 (56)	(N=212) 76 (32)	(N=463) 98 (17)		(N=353) 150 (44)	(N=638) 179 (28)	(N=6) 2 (33)	(N=1,920) 737 (38)	(N=2,825) 820 (27)	(N=25) 10 (40)	<0.001
Mental health and wellbeing	(N=684) 377 (55)	(N=737) 418 (55)	(N=6) 3 (50)	(N=279) 167 (62)	(N=486) 307 (61)	(N=4) 2 (50)	(N=414) 287 (73)	(N=545) 402 (77)	(N=11) 10 (91)	(N=216) 122 (56)	(N=475) 326 (63)		(N=357) 128 (40)	(N=645) 305 (46)	(N=6) 3 (50)	(N=1,950) 1,081 (57)	(N=2,888) 1,758 (60)	(N=27) 18 (67)	0.326
Living arrangements	(N=679) 323 (46)	(N=734) 320 (44)	(N=6) 3 (50)	(N=275) 106 (48)	(N=474) 182 (42)	(N=4) 1 (25)	(N=409) 79 (21)	(N=525) 131 (27)	(N=9) 5 (56)	(N=211) 40 (19)	(N=463) 74 (14)		(N=354) 53 (12)	(N=639) 121 (18)	(N=6) 3 (50)	(N=1,928) 601 (31)	(N=2,835) 828 (31)	(N=25) 12 (48)	0.948
Infrastructure	(N=672) 316 (46)	(N=731) 332 (47)	(N=6) 3 (50)	(N=276) 129 (42)	(N=470) 177 (48)	(N=4) 2 (50)	(N=407) 102 (27)	(N=520) 106 (21)	(N=8) 4 (50)	(N=209) 51 (29)	(N=463) 112 (27)		(N=353) 60 (14)	(N=637) 133 (24)	(N=6) 2 (33)	(N=1,917) 658 (32)	(N=2,821) 860 (34)	(N=24) 11 (46)	0.536
Social	(N=689) 369 (53)	(N=745) 395 (51)	(N=6) 4 (67)	(N=280) 179 (62)	(N=489) 294 (48)	(N=4) 1 (25)	(N=412) 321 (79)	(N=551) 438 (79)	(N=11) 9 (82)	(N=215) 163 (66)	(N=471) 362 (74)		(N=360) 245 (70)	(N=649) 475 (69)	(N=6) 5 (83)	(N=1,956) 1,277 (65)	(N=2,905) 1,964 (63)	(N=27) 19 (70)	0.503
Religious and spiritual	(N=689) 290 (41)	(N=738) 298 (44)	(N=6) 3 (50)	(N=279) 140 (55)	(N=486) 251 (61)	(N=4) 2 (50)	(N=408) 73 (19)	(N=524) 86 (14)	(N=10) 3 (30)	(N=208) 33 (21)	(N=462) 62 (15)		(N=355) 77 (24)	(N=637) 124 (14)	(N=6) 0 (0)	(N=1,939) 613 (33)	(N=2,847) 821 (30)	(N=26) 8 (31)	0.367

Osterrieder A, *et al. BMJ Open* 2021; 11:e046863. doi: 10.1136/bmjopen-2020-046863

Suppl. Table 11 Breakdown of concerns if advised/not allowed physical contact by country and age group

Values in cells are n (weighted %) of respondents who replied 'yes'.

**Variable and categories**	**Thailand**			**Malaysia**			**UK**			**Italy**			**Slovenia**			**Total**			
**Age group**	**18-34**	**35-64**	**65+**	**18-34**	**35-64**	**65+**	**18-34**	**35-64**	**65+**	**18-34**	**35-64**	**65+**	**18-34**	**35-64**	**65+**	**18-34**	**35-64**	**65+**	**P-value (for total)**
**What are/were your concerns if advised no physical contact/not allowed to go out/allowed to go out only for essential needs?**	N=223	N=1,152	N=101	N=350	N=442	N=35	N=140	N=616	N=253	N=272	N=383	N=57	N=308	N=676	N=50	N=1,293	N=3,269	N=496	
Caring responsibilities	(N=217) 137 (71)	(N=1,138) 717 (64)	(N=99) 36 (37)	(N=333) 189 (56)	(N=407) 249 (57)	(N=32) 18 (66)	(N=131) 27 (20)	(N=581) 242 (41)	(N=234) 56 (23)	(N=270) 83 (30)	(N=361) 208 (55)	(N=50) 21 (43)	(N=304) 98 (30)	(N=656) 317 (44)	(N=46) 8 (16)	(N=1,255) 534 (46)	(N=3,143) 1,733 (53)	(N=461) 139 (32)	<0.001
Physical health	(N=218) 150 (63)	(N=1,139) 712 (63)	(N=100) 48 (47)	(N=336) 205 (60)	(N=413) 269 (65)	(N=33) 27 (98)	(N=134) 76 (61)	(N=586) 354 (60)	(N=241) 157 (64)	(N=270) 137 (45)	(N=365) 217 (57)	(N=52) 39 (90)	(N=305) 131 (40)	(N=655) 284 (42)	(N=47) 22 (59)	(N=1,263) 699 (56)	(N=3,158) 1,836 (57)	(N=473) 293 (66)	0.044
Recreational	(N=212) 121 (47)	(N=1,118) 425 (35)	(N=95) 34 (34)	(N=331) 183 (55)	(N=403) 209 (44)	(N=29) 15 (40)	(N=136) 96 (66)	(N=589) 339 (57)	(N=238) 136 (53)	(N=270) 169 (66)	(N=362) 166 (44)	(N=51) 17 (38)	(N=302) 213 (71)	(N=663) 395 (60)	(N=46) 28 (70)	(N=1,251) 782 (59)	(N=3,135) 1,534 (47)	(N=459) 230 (48)	0.003
Sports	(N=212) 99 (47)	(N=1,096) 428 (38)	(N=92) 19 (18)	(N=329) 140 (47)	(N=397) 154 (31)	(N=29) 8 (29)	(N=133) 40 (28)	(N=575) 133 (22)	(N=235) 41 (14)	(N=269) 93 (40)	(N=356) 74 (19)	(N=50) 7 (20)	(N=301) 114 (41)	(N=653) 206 (36)	(N=43) 11 (31)	(N=1,244) 486 (42)	(N=3,077) 995 (31)	(N=449) 86 (21)	<0.001
Mental health and wellbeing	(N=212) 146 (63)	(N=1,118) 613 (55)	(N=97) 39 (42)	(N=335) 230 (69)	(N=402) 227 (52)	(N=32) 19 (69)	(N=136) 118 (86)	(N=591) 439 (74)	(N=243) 142 (62)	(N=270) 191 (65)	(N=366) 227 (59)	(N=55) 30 (57)	(N=304) 169 (52)	(N=657) 253 (40)	(N=47) 14 (40)	(N=1,257) 854 (67)	(N=3,134) 1,759 (56)	(N=474) 244 (51)	<0.001
Living arrangements	(N=213) 105 (50)	(N=1,111) 518 (48)	(N=95) 23 (26)	(N=330) 142 (47)	(N=394) 137 (45)	(N=29) 10 (40)	(N=134) 47 (35)	(N=576) 144 (24)	(N=233) 24 (10)	(N=270) 60 (21)	(N=353) 52 (16)	(N=51) 2 (14)	(N=304) 76 (22)	(N=651) 100 (17)	(N=44) 1 (1)	(N=1,251) 430 (38)	(N=3,085) 951 (32)	(N=452) 60 (15)	<0.001
Infrastructure	(N=214) 117 (54)	(N=1,101) 502 (46)	(N=94) 32 (34)	(N=331) 149 (42)	(N=390) 152 (46)	(N=29) 7 (47)	(N=134) 37 (31)	(N=569) 133 (23)	(N=232) 42 (16)	(N=269) 59 (22)	(N=353) 91 (28)	(N=50) 13 (35)	(N=302) 63 (18)	(N=649) 121 (19)	(N=45) 11 (19)	(N=1,250) 425 (37)	(N=3,062) 999 (33)	(N=450) 105 (28)	0.112
Social	(N=216) 147 (59)	(N=1,126) 573 (50)	(N=98) 48 (46)	(N=334) 212 (55)	(N=408) 240 (55)	(N=31) 22 (60)	(N=136) 115 (83)	(N=592) 459 (77)	(N=246) 194 (79)	(N=268) 220 (84)	(N=366) 266 (69)	(N=52) 39 (63)	(N=304) 239 (79)	(N=662) 453 (65)	(N=49) 33 (69)	(N=1,258) 933 (69)	(N=3,154) 1,991 (62)	(N=476) 336 (64)	0.156
Religious and spiritual	(N=213) 86 (45)	(N=1,120) 468 (43)	(N=100) 37 (37)	(N=334) 180 (65)	(N=406) 198 (51)	(N=29) 15 (61)	(N=133) 14 (15)	(N=574) 111 (19)	(N=235) 37 (13)	(N=268) 27 (12)	(N=352) 64 (17)	(N=50) 4 (25)	(N=304) 51 (15)	(N=650) 142 (19)	(N=44) 8 (24)	(N=1,252) 358 (35)	(N=3,102) 983 (31)	(N=458) 101 (28)	0.198

Suppl. Table 12 Breakdown of concerns if advised/not allowed physical contact by country and education level

P/S = primary or lower/secondary education; T = tertiary education. Values in cells are n (weighted %) of respondents who replied 'yes'.

**Variable and categories**	**Thailand**		**Malaysia**		**UK**		**Italy**		**Slovenia**		**Total**		
**Education level**	**P/S**	**T**	**P/S**	**T**	**P/S**	**T**	**P/S**	**T**	**P/S**	**T**	**P/S**	**T**	**P-value (for total)**
**What are/were your concerns if advised no physical contact/not allowed to go out/allowed to go out only for essential needs?**	N=909	N=567	N=82	N=745	N=247	N=762	N=217	N=495	N=202	N=832	N=1,657	N=3,401	
Caring responsibilities	(N=894) 571 (63)	(N=560) 319 (57)	(N=74) 42 (57)	(N=698) 414 (60)	(N=231) 78 (30)	(N=715) 247 (32)	(N=204) 98 (47)	(N=477) 214 (45)	(N=190) 67 (31)	(N=816) 356 (40)	(N=1,593) 856 (49)	(N=3,266) 1,550 (43)	0.002
Physical health	(N=894) 565 (60)	(N=563) 345 (63)	(N=75) 53 (66)	(N=707) 448 (63)	(N=238) 146 (63)	(N=723) 441 (59)	(N=208) 123 (66)	(N=479) 270 (56)	(N=191) 78 (47)	(N=816) 359 (43)	(N=1,606) 965 (60)	(N=3,288) 1,863 (56)	0.045
Recreational	(N=870) 281 (34)	(N=555) 299 (57)	(N=72) 33 (47)	(N=691) 374 (55)	(N=236) 120 (52)	(N=727) 451 (64)	(N=204) 95 (45)	(N=479) 257 (52)	(N=192) 123 (66)	(N=819) 513 (62)	(N=1,574) 652 (46)	(N=3,271) 1,894 (60)	<0.001
Sports	(N=855) 317 (36)	(N=545) 229 (43)	(N=71) 25 (38)	(N=684) 277 (43)	(N=230) 34 (17)	(N=713) 180 (26)	(N=203) 44 (23)	(N=472) 130 (27)	(N=190) 75 (39)	(N=807) 256 (32)	(N=1,549) 495 (32)	(N=3,221) 1,072 (32)	0.953
Mental health and wellbeing	(N=877) 486 (54)	(N=550) 312 (59)	(N=74) 46 (61)	(N=695) 430 (62)	(N=238) 174 (76)	(N=732) 525 (74)	(N=209) 137 (58)	(N=482) 311 (63)	(N=190) 90 (45)	(N=818) 346 (40)	(N=1,588) 933 (58)	(N=3,277) 1,924 (60)	0.256
Living arrangements	(N=866) 422 (46)	(N=553) 224 (42)	(N=71) 32 (47)	(N=682) 257 (39)	(N=232) 46 (23)	(N=711) 169 (25)	(N=204) 37 (17)	(N=470) 77 (15)	(N=189) 36 (14)	(N=810) 141 (16)	(N=1,562) 573 (33)	(N=3,226) 868 (26)	<0.001
Infrastructure	(N=858) 396 (46)	(N=551) 255 (48)	(N=70) 32 (45)	(N=680) 276 (44)	(N=229) 44 (23)	(N=706) 168 (24)	(N=203) 55 (30)	(N=469) 108 (23)	(N=189) 35 (18)	(N=807) 160 (21)	(N=1,549) 562 (35)	(N=3,213) 967 (29)	0.004
Social	(N=887) 440 (49)	(N=553) 328 (62)	(N=72) 38 (54)	(N=701) 436 (63)	(N=242) 183 (77)	(N=732) 585 (80)	(N=207) 157 (67)	(N=479) 368 (77)	(N=194) 137 (69)	(N=821) 588 (70)	(N=1,602) 955 (60)	(N=3,286) 2,305 (73)	<0.001
Religious and spiritual	(N=882) 391 (44)	(N=551) 200 (36)	(N=71) 42 (60)	(N=698) 351 (51)	(N=232) 36 (17)	(N=710) 126 (17)	(N=202) 36 (20)	(N=468) 59 (13)	(N=190) 28 (18)	(N=808) 173 (21)	(N=1,577) 533 (35)	(N=3,235) 909 (24)	<0.001

Osterrieder A, *et al. BMJ Open* 2021; 11:e046863. doi: 10.1136/bmjopen-2020-046863

Suppl. Table 13 Breakdown of concerns if advised/not allowed physical contact by country and household size

Values in cells are n (weighted %) of respondents who replied 'yes'.

Variable and categories	Thailand		Malaysia		UK		Italy		Slovenia		Total		
**Household size (number of persons in household)**	**1-4**	**>=5**	**1-4**	**>=5**	**1-4**	**>=5**	**1-4**	**>=5**	**1-4**	**>=5**	**1-4**	**>=5**	**P-value (for total)**
**What are/were your concerns if advised no physical contact/not allowed to go out/allowed to go out only for essential needs?**	N=1,038	N=438	N=494	N=333	N=961	N=48	N=659	N=53	N=827	N=207	N=3,979	N=1,079	
Caring responsibilities	(N=1,019) 618 (62)	(N=435) 272 (61)	(N=461) 246 (59)	(N=311) 210 (56)	(N=900) 299 (30)	(N=46) 26 (44)	(N=630) 286 (46)	(N=51) 26 (52)	(N=806) 329 (33)	(N=200) 94 (44)	(N=3,816) 1,778 (45)	(N=1,043) 628 (56)	0.002
Physical health	(N=1,022) 639 (61)	(N=435) 271 (59)	(N=467) 293 (73)	(N=315) 208 (56)	(N=916) 557 (61)	(N=45) 30 (65)	(N=637) 363 (63)	(N=50) 30 (56)	(N=805) 360 (47)	(N=202) 77 (38)	(N=3,847) 2,212 (60)	(N=1,047) 616 (55)	0.153
Recreational	(N=1,002) 385 (35)	(N=423) 195 (46)	(N=456) 241 (47)	(N=307) 166 (51)	(N=918) 549 (59)	(N=45) 22 (53)	(N=633) 327 (47)	(N=50) 25 (50)	(N=809) 518 (65)	(N=202) 118 (61)	(N=3,818) 2,020 (51)	(N=1,027) 526 (50)	0.896
Sports	(N=984) 379 (38)	(N=416) 167 (38)	(N=447) 169 (33)	(N=308) 133 (45)	(N=900) 207 (22)	(N=43) 7 (8)	(N=625) 155 (23)	(N=50) 19 (42)	(N=798) 262 (35)	(N=199) 69 (41)	(N=3,754) 1,172 (30)	(N=1,016) 395 (39)	0.008
Mental health and wellbeing	(N=1,007) 567 (57)	(N=420) 231 (51)	(N=458) 282 (64)	(N=311) 194 (58)	(N=925) 672 (76)	(N=45) 27 (63)	(N=641) 414 (59)	(N=50) 34 (62)	(N=807) 363 (44)	(N=201) 73 (41)	(N=3,838) 2,298 (60)	(N=1,027) 559 (53)	0.031
Living arrangements	(N=1,000) 465 (47)	(N=419) 181 (42)	(N=448) 164 (40)	(N=305) 125 (53)	(N=899) 199 (23)	(N=44) 16 (45)	(N=624) 107 (16)	(N=50) 7 (16)	(N=798) 143 (14)	(N=201) 34 (18)	(N=3,769) 1,078 (28)	(N=1,019) 363 (41)	<0.001
Infrastructure	(N=995) 455 (46)	(N=414) 196 (47)	(N=445) 170 (42)	(N=305) 138 (48)	(N=892) 204 (23)	(N=43) 8 (35)	(N=622) 154 (28)	(N=50) 9 (19)	(N=796) 165 (20)	(N=200) 30 (15)	(N=3,750) 1,148 (31)	(N=1,012) 381 (40)	0.007
Social	(N=1,012) 534 (51)	(N=428) 234 (53)	(N=461) 277 (50)	(N=312) 197 (62)	(N=928) 736 (80)	(N=46) 32 (66)	(N=636) 491 (70)	(N=50) 34 (70)	(N=811) 584 (69)	(N=204) 141 (72)	(N=3,848) 2,622 (65)	(N=1,040) 638 (60)	0.120
Religious and spiritual	(N=1,008) 405 (42)	(N=425) 186 (44)	(N=457) 211 (58)	(N=312) 182 (58)	(N=898) 151 (16)	(N=44) 11 (30)	(N=621) 86 (18)	(N=49) 9 (24)	(N=797) 134 (17)	(N=201) 67 (27)	(N=3,781) 987 (28)	(N=1,031) 455 (44)	<0.001

Suppl. Table 14 Breakdown of concerns if advised/not allowed physical contact by country and whether or not living with children under 18

Values in cells are n (weighted %) of respondents who replied 'yes'.

Variable and categories	Thailand		Malaysia		UK		Italy		Slovenia		Total		
**Living with children under 18**	**Y**	**N**	**Y**	**N**	**Y**	**N**	**Y**	**N**	**Y**	**N**	**Y**	**N**	**P-value (for total)**

**What are/were your concerns if advised no physical contact/not allowed to go out/allowed to go out only for essential needs?**	N=664	N=812	N=346	N=481	N=186	N=823	N=144	N=568	N=497	N=537	N=1,837	N=3,221	
Caring responsibilities	(N=657)<br>487 (73)	(N=797)<br>403 (51)	(N=318)<br>217 (65)	(N=454)<br>239 (52)	(N=177)<br>109 (49)	(N=769)<br>216 (27)	(N=138)<br>88 (63)	(N=543)<br>224 (43)	(N=484)<br>278 (53)	(N=522)<br>145 (22)	(N=1,774)<br>1,179 (64)	(N=3,085)<br>1,227 (38)	<0.001
Physical health	(N=659)<br>458 (67)	(N=798)<br>452 (55)	(N=321)<br>199 (60)	(N=461)<br>302 (70)	(N=179)<br>103 (61)	(N=782)<br>484 (61)	(N=138)<br>77 (56)	(N=549)<br>316 (64)	(N=484)<br>217 (44)	(N=523)<br>220 (46)	(N=1,781)<br>1,054 (59)	(N=3,113)<br>1,774 (59)	0.984
Recreational	(N=644)<br>220 (36)	(N=781)<br>360 (41)	(N=316)<br>169 (48)	(N=447)<br>238 (49)	(N=179)<br>102 (55)	(N=784)<br>469 (59)	(N=139)<br>66 (40)	(N=544)<br>286 (49)	(N=486)<br>284 (60)	(N=525)<br>352 (68)	(N=1,764)<br>841 (46)	(N=3,081)<br>1,705 (53)	0.013
Sports	(N=633)<br>267 (41)	(N=767)<br>279 (35)	(N=318)<br>137 (45)	(N=437)<br>165 (34)	(N=173)<br>52 (24)	(N=770)<br>162 (21)	(N=135)<br>38 (29)	(N=540)<br>136 (23)	(N=478)<br>175 (41)	(N=519)<br>156 (33)	(N=1,737)<br>669 (39)	(N=3,033)<br>898 (29)	<0.001
Mental health and wellbeing	(N=641)<br>415 (63)	(N=786)<br>383 (48)	(N=318)<br>190 (56)	(N=451)<br>286 (65)	(N=180)<br>139 (80)	(N=790)<br>560 (74)	(N=139)<br>91 (60)	(N=552)<br>357 (60)	(N=481)<br>197 (44)	(N=527)<br>239 (43)	(N=1,759)<br>1,032 (59)	(N=3,106)<br>1,825 (58)	0.841
Living arrangements	(N=641)<br>366 (54)	(N=778)<br>280 (37)	(N=311)<br>118 (55)	(N=442)<br>171 (39)	(N=174)<br>56 (36)	(N=769)<br>159 (21)	(N=134)<br>24 (19)	(N=540)<br>90 (16)	(N=479)<br>93 (21)	(N=520)<br>84 (11)	(N=1,739)<br>657 (42)	(N=3,049)<br>784 (24)	<0.001
Infrastructure	(N=632)<br>322 (50)	(N=777)<br>329 (43)	(N=310)<br>131 (48)	(N=440)<br>177 (42)	(N=172)<br>37 (29)	(N=763)<br>175 (23)	(N=135)<br>30 (18)	(N=537)<br>133 (30)	(N=477)<br>81 (17)	(N=519)<br>114 (20)	(N=1,726)<br>601 (37)	(N=3,036)<br>928 (31)	0.018
Social	(N=651)<br>347 (52)	(N=789)<br>421 (52)	(N=322)<br>194 (53)	(N=451)<br>280 (57)	(N=179)<br>141 (82)	(N=795)<br>627 (78)	(N=140)<br>109 (77)	(N=546)<br>416 (69)	(N=488)<br>341 (69)	(N=527)<br>384 (70)	(N=1,780)<br>1,132 (61)	(N=3,108)<br>2,128 (66)	0.098
Religious and spiritual	(N=641)<br>307 (49)	(N=792)<br>284 (36)	(N=319)<br>174 (58)	(N=450)<br>219 (58)	(N=171)<br>30 (19)	(N=771)<br>132 (16)	(N=133)<br>23 (20)	(N=537)<br>72 (18)	(N=479)<br>118 (20)	(N=519)<br>83 (18)	(N=1,743)<br>652 (39)	(N=3,069)<br>790 (28)	<0.001

Suppl. Table 15 Breakdown of concerns if advised/not allowed physical contact by country and <u>income type</u>

FBP = fixed salary, benefits/pension; CF = contract and freelance; O = other/no income. Values in cells are n (weighted %) of respondents who replied 'yes'.

**Variable and categories**	**Thailand**			**Malaysia**			**UK**			**Italy**			**Slovenia**			**Total**			
**Type of income**	**FBP**	**CF**	**O**	**FBP**	**CF**	**O**	**FBP**	**CF**	**O**	**FBP**	**CF**	**O**	**FBP**	**CF**	**O**	**FBP**	**CF**	**O**	**P-value (for total)**
**What are/were your concerns if advised no physical contact/not allowed to go out/allowed to go out only for essential needs?**	N=546	N=849	N=81	N=524	N=158	N=145	N=705	N=227	N=77	N=347	N=244	N=121	N=847	N=103	N=84	N=2,969	N=1,581	N=508	
Caring responsibilities	(N=540) 372 (72)	(N=836) 481 (57)	(N=78) 37 (39)	(N=490) 307 (58)	(N=145) 78 (64)	(N=137) 71 (47)	(N=661) 223 (32)	(N=213) 83 (32)	(N=72) 19 (26)	(N=328) 167 (49)	(N=236) 101 (41)	(N=117) 44 (44)	(N=826) 362 (36)	(N=97) 42 (31)	(N=83) 19 (23)	(N=2,845) 1,431 (47)	(N=1,527) 785 (51)	(N=487) 190 (38)	0.028
Physical health	(N=543) 381 (70)	(N=835) 482 (56)	(N=79) 47 (49)	(N=497) 324 (63)	(N=146) 89 (71)	(N=139) 88 (66)	(N=672) 415 (62)	(N=216) 124 (60)	(N=73) 48 (63)	(N=333) 204 (68)	(N=236) 122 (51)	(N=118) 67 (59)	(N=826) 345 (44)	(N=98) 56 (58)	(N=83) 36 (42)	(N=2,871) 1,669 (59)	(N=1,531) 873 (58)	(N=492) 286 (57)	0.826
Recreational	(N=535) 243 (43)	(N=812) 296 (35)	(N=78) 41 (42)	(N=483) 253 (46)	(N=143) 78 (48)	(N=137) 76 (56)	(N=671) 386 (54)	(N=218) 134 (65)	(N=74) 51 (71)	(N=331) 153 (46)	(N=236) 136 (50)	(N=116) 63 (47)	(N=828) 511 (62)	(N=101) 63 (75)	(N=82) 62 (75)	(N=2,848) 1,546 (52)	(N=1,510) 707 (46)	(N=487) 293 (58)	0.024
Sports	(N=531) 264 (53)	(N=791) 249 (29)	(N=78) 33 (32)	(N=474) 190 (35)	(N=145) 63 (47)	(N=136) 49 (39)	(N=660) 133 (18)	(N=213) 57 (28)	(N=70) 24 (30)	(N=325) 72 (22)	(N=234) 70 (26)	(N=116) 32 (28)	(N=818) 265 (34)	(N=96) 34 (46)	(N=83) 32 (45)	(N=2,808) 924 (32)	(N=1,479) 473 (32)	(N=483) 170 (36)	0.582
Mental health and wellbeing	(N=533) 339 (65)	(N=816) 410 (50)	(N=78) 49 (50)	(N=485) 297 (61)	(N=146) 86 (58)	(N=138) 93 (66)	(N=676) 485 (75)	(N=221) 157 (74)	(N=73) 57 (80)	(N=335) 213 (60)	(N=238) 147 (55)	(N=118) 88 (68)	(N=826) 346 (43)	(N=99) 42 (38)	(N=83) 48 (53)	(N=2,855) 1,680 (59)	(N=1,520) 842 (55)	(N=490) 335 (63)	0.125
Living arrangements	(N=533) 268 (51)	(N=808) 352 (43)	(N=78) 26 (27)	(N=474) 181 (48)	(N=142) 54 (55)	(N=137) 54 (27)	(N=655) 128 (19)	(N=216) 65 (34)	(N=72) 22 (30)	(N=325) 57 (17)	(N=233) 38 (16)	(N=116) 19 (14)	(N=821) 138 (14)	(N=95) 15 (13)	(N=83) 24 (29)	(N=2,808) 772 (27)	(N=1,494) 524 (38)	(N=486) 145 (26)	<0.001
Infrastructure	(N=530) 279 (56)	(N=800) 335 (42)	(N=79) 37 (35)	(N=473) 179 (46)	(N=141) 55 (39)	(N=136) 74 (48)	(N=654) 134 (21)	(N=210) 56 (30)	(N=71) 22 (29)	(N=325) 74 (30)	(N=230) 56 (23)	(N=117) 33 (26)	(N=819) 157 (19)	(N=94) 15 (13)	(N=83) 23 (25)	(N=2,801) 823 (32)	(N=1,475) 517 (36)	(N=486) 189 (35)	0.370
Social	(N=537) 322 (58)	(N=824) 398 (48)	(N=79) 48 (51)	(N=491) 303 (55)	(N=146) 81 (59)	(N=136) 90 (52)	(N=681) 531 (78)	(N=219) 177 (79)	(N=74) 60 (81)	(N=335) 256 (72)	(N=233) 173 (63)	(N=118) 96 (78)	(N=834) 589 (68)	(N=98) 66 (67)	(N=83) 70 (86)	(N=2,878) 2,001 (67)	(N=1,520) 895 (58)	(N=490) 364 (67)	0.004
Religious and spiritual	(N=532) 235 (49)	(N=823) 326 (39)	(N=78) 30 (35)	(N=486) 254 (57)	(N=145) 68 (57)	(N=138) 71 (62)	(N=659) 121 (17)	(N=210) 31 (16)	(N=73) 10 (12)	(N=322) 43 (20)	(N=231) 36 (14)	(N=117) 16 (17)	(N=821) 168 (18)	(N=94) 22 (31)	(N=83) 11 (14)	(N=2,820) 821 (29)	(N=1,503) 483 (34)	(N=489) 138 (33)	0.195

Suppl. Table 16 Breakdown of maximum number of days that people thought they could cope by country

Values in cells are n (weighted %) of respondents who replied 'yes'.

Variable and categories	Thailand	Malaysia	UK	Italy	Slovenia	Total	P-value
**What is the maximum number of days you think you could cope without meeting family or friends not living in your household in person?**	N=1,476	N=827	N=1,009	N=712	N=1,034	N=5,058	<0.001
1 to 14 days	957 (66)	201 (31)	192 (21)	127 (23)	261 (34)	1,738 (39)	
>14 to 28 days	223 (13)	110 (16)	98 (11)	95 (14)	169 (16)	695 (14)	
29 days+	296 (21)	516 (52)	719 (68)	490 (63)	604 (50)	2,625 (47)	
**What is the maximum number of days you think you could cope with not going out in public, assuming that you have sufficient supplies of food, medicines and other essential items?**	N=1,476	N=827	N=1,009	N=712	N=1,034	N=5,058	<0.001
1 to 14 days	805 (54)	270 (41)	393 (40)	304 (45)	601 (61)	2,373 (49)	
>14 to 28 days	249 (17)	114 (16)	124 (14)	161 (21)	151 (13)	799 (16)	
29 days+	422 (29)	443 (43)	492 (46)	247 (34)	282 (26)	1,886 (35)	
**What is the maximum number of days you think you could cope with going out only for essential needs/work?**	N=1,476	N=827	N=1,009	N=712	N=1,034	N=5,058	<0.001
1 to 14 days	808 (56)	268 (40)	272 (29)	205 (33)	310 (37)	1,863 (41)	
>14 to 28 days	258 (17)	98 (14)	100 (10)	110 (17)	182 (18)	748 (15)	
29 days+	410 (26)	461 (46)	637 (60)	397 (51)	542 (45)	2,447 (44)	

Suppl. Table 17 Breakdown of maximum number of days that people thought they could cope by country and gender

M = male; F = female; O = other/prefer not to say. Values in cells are n (weighted %) of respondents who replied 'yes'.

**Variable and categories**	**Thailand**			**Malaysia**			**UK**			**Italy**			**Slovenia**			**Total**			
**Gender**	**M**	**F**	**O**	**M**	**F**	**O**	**M**	**F**	**O**	**M**	**F**	**O**	**M**	**F**	**O**	**M**	**F**	**O**	**P-value (for total M vs F)**
**What is the maximum number of days you think you could cope without meeting family or friends not living in your household in person?**	N=704	N=766	N=6	N=298	N=525	N=4	N=426	N=572	N=11	N=222	N=490	N=0	N=366	N=662	N=6	N=2,016	N=3,015	N=27	0.381
1 to 14 days	479 (66)	476 (66)	2 (33)	68 (29)	132 (34)	1 (25)	87 (23)	102 (19)	3 (27)	46 (28)	81 (18)		113 (38)	147 (31)	1 (17)	793 (40)	938 (37)	7 (26)	
>14 to 28 days	99 (12)	123 (15)	1 (17)	40 (14)	69 (18)	1 (25)	43 (13)	54 (9)	1 (9)	28 (11)	67 (17)		49 (14)	120 (18)	0 (0)	259 (13)	433 (15)	3 (11)	
29 days+	126 (23)	167 (19)	3 (50)	190 (57)	324 (48)	2 (50)	296 (64)	416 (72)	7 (64)	148 (61)	342 (65)		204 (48)	395 (51)	5 (83)	964 (47)	1,644 (47)	17 (63)	
**What is the maximum number of days you think you could cope with not going out in public, assuming that you have sufficient supplies of food, medicines and other essential items?**	N=704	N=766	N=6	N=298	N=525	N=4	N=426	N=572	N=11	N=222	N=490		N=366	N=662	N=6	N=2,016	N=3,015	N=27	0.890
1 to 14 days	398 (53)	405 (55)	2 (33)	96 (41)	173 (40)	1 (25)	170 (42)	219 (38)	4 (36)	100 (48)	204 (42)		217 (57)	382 (65)	2 (33)	981 (49)	1,383 (50)	9 (33)	
>14 to 28 days	116 (18)	132 (16)	1 (17)	47 (18)	66 (14)	1 (25)	53 (14)	71 (13)	0 (0)	46 (18)	115 (24)		40 (14)	111 (12)	0 (0)	302 (16)	495 (16)	2 (7)	
29 days+	190 (30)	229 (29)	3 (50)	155 (41)	286 (46)	2 (50)	203 (43)	282 (49)	7 (64)	76 (34)	171 (34)		109 (29)	169 (23)	4 (67)	733 (35)	1,137 (35)	16 (59)	
**What is the maximum number of days you think you could cope with going out only for essential needs/work?**	N=704	N=766	N=6	N=298	N=525	N=4	N=426	N=572	N=11	N=222	N=490		N=366	N=662	N=6	N=2,016	N=3,015	N=27	0.680
1 to 14 days	418 (57)	388 (55)	2 (33)	94 (41)	173 (38)	1 (25)	127 (32)	141 (27)	4 (36)	72 (35)	133 (31)		125 (35)	183 (40)	2 (33)	836 (42)	1,018 (40)	9 (33)	
>14 to 28 days	114 (17)	142 (17)	2 (33)	35 (11)	62 (17)	1 (25)	40 (10)	60 (10)	0 (0)	31 (17)	79 (17)		73 (23)	109 (13)	0 (0)	293 (16)	452 (15)	3 (11)	
29 days+	172 (25)	236 (27)	2 (33)	169 (47)	290 (45)	2 (50)	259 (58)	371 (62)	7 (64)	119 (49)	278 (52)		168 (43)	370 (47)	4 (67)	887 (42)	1,545 (45)	15 (56)	

Osterrieder A, *et al. BMJ Open* 2021; 11:e046863. doi: 10.1136/bmjopen-2020-046863

Suppl. Table 18 Breakdown of maximum number of days that people thought they could cope by country and age group

Values in cells are n (weighted %) of respondents who replied 'yes'.

Variable and categories	Thailand			Malaysia			UK			Italy			Slovenia			Total			
**Age group**	**18-34**	**35-64**	**65+**	**18-34**	**35-64**	**65+**	**18-34**	**35-64**	**65+**	**18-34**	**35-64**	**65+**	**18-34**	**35-64**	**65+**	**18-34**	**35-64**	**65+**	**P-value (for total)**
**What is the maximum number of days you think you could cope without meeting family or friends not living in your household in person?**	N=223	N=1,152	N=101	N=350	N=442	N=35	N=140	N=616	N=253	N=272	N=383	N=57	N=308	N=676	N=50	N=1,293	N=3,269	N=496	0.409
1 to 14 days	115 (57)	774 (70)	68 (67)	96 (32)	96 (25)	9 (55)	22 (22)	112 (18)	58 (24)	37 (19)	81 (26)	9 (19)	78 (29)	167 (31)	16 (49)	348 (36)	1,230 (39)	160 (42)	
>14 to 28 days	29 (10)	179 (15)	15 (15)	51 (19)	53 (13)	6 (22)	16 (13)	55 (10)	27 (12)	42 (20)	42 (11)	11 (17)	49 (17)	112 (15)	8 (18)	187 (10)	441 (13)	67 (16)	
29 days+	79 (33)	199 (15)	18 (18)	203 (49)	293 (62)	20 (23)	102 (65)	449 (72)	168 (64)	193 (62)	260 (63)	37 (64)	181 (54)	397 (54)	26 (34)	758 (50)	1,598 (48)	269 (42)	
**What is the maximum number of days you think you could cope with not going out in public, assuming that you have sufficient supplies of food, medicines and other essential items?**	N=223	N=1,152	N=101	N=350	N=442	N=35	N=140	N=616	N=253	N=272	N=383	N=57	N=308	N=676	N=50	N=1,293	N=3,269	N=496	0.335
1 to 14 days	113 (48)	643 (58)	49 (50)	116 (42)	141 (36)	13 (56)	62 (42)	222 (37)	109 (47)	111 (45)	170 (44)	23 (47)	192 (61)	382 (59)	27 (67)	594 (47)	1,558 (49)	221 (53)	
>14 to 28 days	33 (17)	192 (16)	24 (20)	43 (13)	65 (17)	6 (28)	19 (17)	85 (14)	20 (9)	65 (19)	82 (19)	14 (27)	36 (11)	107 (14)	8 (15)	196 (15)	531 (16)	72 (18)	
29 days+	77 (35)	317 (26)	28 (30)	191 (45)	236 (47)	16 (16)	59 (40)	309 (50)	124 (45)	96 (36)	131 (37)	20 (26)	80 (28)	187 (28)	15 (19)	503 (37)	1,180 (36)	203 (29)	
**What is the maximum number of days you think you could cope with going out only for essential needs/work?**	N=223	N=1,152	N=101	N=350	N=442	N=35	N=140	N=616	N=253	N=272	N=383	N=57	N=308	N=676	N=50	N=1,293	N=3,269	N=496	0.255
1 to 14 days	107 (52)	648 (59)	53 (56)	91 (32)	163 (43)	14 (62)	33 (28)	161 (27)	78 (36)	62 (27)	126 (36)	17 (32)	98 (34)	189 (33)	23 (51)	391 (37)	1,287 (42)	185 (46)	
>14 to 28 days	43 (18)	195 (17)	20 (17)	40 (13)	54 (14)	4 (15)	17 (12)	58 (10)	25 (8)	48 (20)	52 (14)	10 (20)	53 (17)	121 (17)	8 (19)	201 (16)	480 (15)	67 (16)	
29 days+	73 (30)	309 (24)	28 (27)	219 (55)	225 (43)	17 (22)	90 (60)	397 (63)	150 (56)	162 (53)	205 (51)	30 (48)	157 (49)	366 (50)	19 (29)	701 (48)	1,502 (43)	244 (38)	

Suppl. Table 19 Breakdown of maximum number of days that people thought they could cope by country and household size

Values in cells are n (weighted %) of respondents who replied 'yes'.

Variable and categories	Thailand		Malaysia		UK		Italy		Slovenia		Total		
**Household size (number of persons in household)**	**1-4**	**≥5**	**1-4**	**≥5**	**1-4**	**≥5**	**1-4**	**≥5**	**1-4**	**≥5**	**1-4**	**≥5**	**P-value (for total)**
**What is the maximum number of days you think you could cope without meeting family or friends not living in your household in person?**	N=1,038	N=438	N=494	N=333	N=961	N=48	N=659	N=53	N=827	N=207	N=3,979	N=1,079	0.023
1 to 14 days	674 (68)	283 (61)	105 (26)	96 (38)	185 (21)	7 (12)	118 (23)	9 (24)	216 (36)	45 (28)	1,298 (37)	440 (44)	
>14 to 28 days	150 (13)	73 (15)	67 (12)	43 (22)	95 (12)	3 (3)	93 (15)	2 (2)	139 (17)	30 (12)	544 (14)	151 (16)	
29 days+	214 (19)	82 (24)	322 (62)	194 (40)	681 (67)	38 (85)	448 (62)	42 (75)	472 (47)	132 (59)	2,137 (49)	488 (40)	
**What is the maximum number of days you think you could cope with not going out in public, assuming that you have sufficient supplies of food, medicines and other essential items?**	N=1,038	N=438	N=494	N=333	N=961	N=48	N=659	N=53	N=827	N=207	N=3,979	N=1,079	0.492
1 to 14 days	594 (59)	211 (44)	160 (29)	110 (56)	375 (40)	18 (49)	285 (45)	19 (46)	487 (61)	114 (63)	1,901 (49)	472 (51)	
>14 to 28 days	158 (14)	91 (22)	68 (19)	46 (12)	114 (14)	10 (14)	146 (21)	15 (24)	123 (14)	28 (11)	609 (16)	190 (17)	
29 days+	286 (27)	136 (34)	266 (52)	177 (32)	472 (47)	20 (37)	228 (34)	19 (30)	217 (26)	65 (26)	1,469 (36)	417 (32)	
**What is the maximum number of days you think you could cope with going out only for essential needs/work?**	N=1,038	N=438	N=494	N=333	N=961	N=48	N=659	N=53	N=827	N=207	N=3,979	N=1,079	0.079
1 to 14 days	579 (58)	229 (54)	165 (35)	103 (47)	262 (30)	10 (24)	197 (33)	8 (21)	255 (37)	55 (40)	1,458 (39)	405 (47)	
>14 to 28 days	172 (15)	86 (21)	63 (20)	35 (6)	96 (11)	4 (5)	104 (17)	6 (8)	146 (18)	36 (14)	581 (16)	167 (14)	
29 days+	287 (27)	123 (25)	266 (46)	195 (46)	603 (60)	34 (72)	358 (50)	39 (72)	426 (45)	116 (46)	1,940 (45)	507 (39)	

Suppl. Table 20 Breakdown of maximum number of days that people thought they could cope by country and whether or not living with children under 18

Y = living with children under 18; N = not living with children under 18. Values in cells are n (weighted %) of respondents who replied 'yes'.

**Variable and categories**	**Thailand**		**Malaysia**		**UK**		**Italy**		**Slovenia**		**Total**		
**Living with children under 18**	**Y**	**N**	**Y**	**N**	**Y**	**N**	**Y**	**N**	**Y**	**N**	**Y**	**N**	**P-value (for total)**
**What is the maximum number of days you think you could cope without meeting family or friends not living in your household in person?**	N=664	N=812	N=346	N=481	N=186	N=823	N=144	N=568	N=497	N=537	N=1,837	N=3,221	<0.001
1 to 14 days	490 (72)	467 (60)	97 (40)	104 (25)	24 (14)	168 (22)	24 (18)	103 (24)	115 (30)	146 (38)	750 (46)	988 (35)	
>14 to 28 days	80 (10)	143 (17)	37 (12)	73 (19)	18 (12)	80 (11)	13 (9)	82 (16)	79 (14)	90 (18)	227 (12)	468 (16)	
29 days+	94 (18)	202 (23)	212 (47)	304 (56)	144 (74)	575 (67)	107 (73)	383 (61)	303 (57)	301 (45)	860 (42)	1,765 (50)	
**What is the maximum number of days you think you could cope with not going out in public, assuming that you have sufficient supplies of food, medicines and other essential items?**	N=664	N=812	N=346	N=481	N=186	N=823	N=144	N=568	N=497	N=537	N=1,837	N=3,221	<0.001
1 to 14 days	412 (59)	393 (49)	120 (57)	150 (29)	60 (36)	333 (41)	62 (44)	242 (45)	290 (62)	311 (60)	944 (56)	1,429 (46)	
>14 to 28 days	100 (16)	149 (18)	45 (11)	69 (20)	34 (19)	90 (12)	33 (26)	128 (20)	73 (13)	78 (14)	285 (15)	514 (17)	
29 days+	152 (25)	270 (33)	181 (33)	262 (51)	92 (46)	400 (46)	49 (31)	198 (34)	134 (25)	148 (26)	608 (29)	1,278 (38)	
**What is the maximum number of days you think you could cope with going out only for essential needs/work?**	N=664	N=812	N=346	N=481	N=186	N=823	N=144	N=568	N=497	N=537	N=1,837	N=3,221	0.004
1 to 14 days	407 (63)	401 (51)	117 (47)	151 (35)	33 (21)	239 (31)	42 (35)	163 (32)	139 (35)	171 (39)	738 (47)	1,125 (38)	
>14 to 28 days	112 (16)	146 (18)	37 (8)	61 (18)	17 (8)	83 (11)	20 (11)	90 (18)	90 (16)	92 (18)	276 (14)	472 (16)	
29 days+	145 (21)	265 (31)	192 (45)	269 (47)	136 (71)	501 (58)	82 (53)	315 (50)	268 (49)	274 (42)	823 (40)	1,624 (46)	

Suppl. Table 21 Breakdown of maximum number of days that people thought they could cope by country and education level

P/S = primary or lower/secondary education; T = tertiary education. Values in cells are n (weighted %) of respondents who replied 'yes'.

**Variable and categories**	**Thailand**		**Malaysia**		**UK**		**Italy**		**Slovenia**		**Total**		
**Education level**	**P/S**	**T**	**P/S**	**T**	**P/S**	**T**	**P/S**	**T**	**P/S**	**T**	**P/S**	**T**	**P-value (for total)**
**What is the maximum number of days you think you could cope without meeting family or friends not living in your household in person?**	N=909	N=567	N=82	N=745	N=247	N=762	N=217	N=495	N=202	N=832	N=1,657	N=3,401	<0.001
1 to 14 days	659 (69)	298 (51)	27 (33)	174 (23)	55 (24)	137 (18)	53 (26)	74 (16)	69 (41)	192 (24)	863 (45)	875 (25)	
>14 to 28 days	122 (12)	101 (17)	15 (17)	95 (13)	30 (13)	68 (9)	31 (15)	64 (13)	33 (16)	136 (16)	231 (15)	464 (13)	
29 days+	128 (18)	168 (32)	40 (50)	476 (64)	162 (63)	557 (73)	133 (59)	357 (72)	100 (43)	504 (60)	563 (41)	2,062 (62)	
**What is the maximum number of days you think you could cope with not going out in public, assuming that you have sufficient supplies of food, medicines and other essential items?**	N=909	N=567	N=82	N=745	N=247	N=762	N=217	N=495	N=202	N=832	N=1,657	N=3,401	0.004
1 to 14 days	541 (56)	264 (47)	34 (43)	236 (32)	101 (41)	292 (40)	95 (46)	209 (43)	119 (63)	482 (58)	890 (51)	1,483 (45)	
>14 to 28 days	144 (17)	105 (18)	15 (17)	99 (13)	31 (15)	93 (13)	41 (20)	120 (24)	23 (12)	128 (15)	254 (16)	545 (16)	
29 days+	224 (28)	198 (35)	33 (40)	410 (55)	115 (44)	377 (48)	81 (34)	166 (33)	60 (25)	222 (27)	513 (33)	1,373 (39)	
**What is the maximum number of days you think you could cope with going out only for essential needs/work?**	N=909	N=567	N=82	N=745	N=247	N=762	N=217	N=495	N=202	N=832	N=1,657	N=3,401	<0.001
1 to 14 days	564 (59)	244 (43)	35 (43)	233 (29)	87 (35)	185 (24)	70 (35)	135 (29)	75 (42)	235 (31)	831 (46)	1,032 (30)	
>14 to 28 days	156 (17)	102 (19)	12 (14)	86 (11)	26 (10)	74 (10)	39 (18)	71 (14)	33 (17)	149 (18)	266 (16)	482 (14)	
29 days+	189 (24)	221 (38)	35 (43)	426 (59)	134 (54)	503 (66)	108 (48)	289 (57)	94 (41)	448 (51)	560 (38)	1,887 (56)	

Suppl. Table 22 Breakdown of maximum number of days that people thought they could cope by country and type of income

FBP = fixed salary, benefits/pension; CF = contract and freelance; O = other. Values in cells are n (weighted %) of respondents who replied 'yes'.

Variable and categories	Thailand			Malaysia			UK			Italy			Slovenia			Total			
**Type of income**	**FBP**	**CF**	**O**	**FBP**	**CF**	**O**	**FBP**	**CF**	**O**	**FBP**	**CF**	**O**	**FBP**	**CF**	**O**	**FBP**	**CF**	**O**	**P-value (for total)**
**What is the maximum number of days you think you could cope without meeting family or friends not living in your household in person?**	N=546	N=849	N=81	N=524	N=158	N=145	N=705	N=227	N=77	N=347	N=244	N=121	N=847	N=103	N=84	N=2,969	N=1,581	N=508	<0.001
1 to 14 days	344 (64)	577 (69)	36 (43)	135 (23)	35 (37)	31 (48)	134 (22)	36 (17)	22 (24)	58 (22)	47 (27)	22 (18)	208 (34)	35 (44)	18 (26)	879 (33)	730 (50)	129 (34)	
>14 to 28 days	74 (11)	134 (14)	15 (17)	57 (15)	24 (16)	29 (19)	69 (11)	25 (14)	4 (7)	46 (15)	30 (12)	19 (15)	141 (17)	19 (16)	9 (9)	387 (14)	232 (14)	76 (14)	
29 days+	128 (25)	138 (16)	30 (41)	332 (62)	99 (47)	85 (33)	502 (68)	166 (69)	51 (69)	243 (63)	167 (60)	80 (66)	498 (49)	49 (40)	57 (65)	1,703 (53)	619 (35)	303 (51)	
**What is the maximum number of days you think you could cope with not going out in public, assuming that you have sufficient supplies of food, medicines and other essential items?**	N=546	N=849	N=81	N=524	N=158	N=145	N=705	N=227	N=77	N=347	N=244	N=121	N=847	N=103	N=84	N=2,969	N=1,581	N=508	0.471
1 to 14 days	313 (55)	461 (55)	31 (39)	183 (38)	46 (39)	41 (49)	273 (40)	87 (41)	33 (42)	147 (45)	108 (47)	49 (40)	485 (560	66 (75)	50 (59)	1,401 (49)	768 (51)	204 (46)	
>14 to 28 days	85 (16)	148 (17)	16 (20)	70 (18)	22 (17)	22 (10)	90 (13)	28 (17)	6 (9)	84 (24)	55 (17)	22 (14)	129 (14)	12 (7)	10 (14)	458 (16)	265 (16)	76 (13)	
29 days+	148 (29)	240 (28)	34 (40)	271 (44)	90 (44)	82 (41)	342 (47)	112 (43)	38 (49)	116 (30)	81 (36)	50 (46)	233 (27)	25 (18)	24 (27)	1,110 (35)	548 (33)	228 (41)	
**What is the maximum number of days you think you could cope with going out only for essential needs/work?**	N=546	N=849	N=81	N=524	N=158	N=145	N=705	N=227	N=77	N=347	N=244	N=121	N=847	N=103	N=84	N=2,969	N=1,581	N=508	<0.001
1 to 14 days	297 (59)	478 (56)	33 (43)	181 (38)	56 (53)	31 (29)	186 (29)	64 (31)	22 (22)	99 (33)	78 (34)	28 (27)	250 (38)	41 (45)	19 (27)	1,013 (39)	717 (49)	133 (30)	
>14 to 28 days	81 (16)	159 (18)	18 (23)	54 (14)	23 (4)	21 (25)	68 (10)	20 (10)	12 (16)	55 (18)	30 (12)	25 (19)	150 (17)	17 (21)	15 (17)	408 (15)	249 (14)	91 (21)	
29 days+	168 (25)	212 (26)	30 (34)	289 (48)	79 (43)	93 (46)	451 (61)	143 (58)	43 (62)	193 (49)	136 (53)	68 (54)	447 (45)	45 (34)	50 (57)	1,548 (46)	615 (37)	284 (50)	

Suppl. Table 23 Breakdown of behavioural changes and acceptance of government public health measures by country

Values in cells are n (weighted %) of respondents who replied 'yes'.

Variable and categories	Thailand	Malaysia	UK	Italy	Slovenia	Total	P-value
	N=1,476	N=827	N=1,009	N=712	N=1,034	N=5,058	
**Did you change your social behaviour before the implementation of government restrictions?**	1,374 (93)	538 (64)	712 (68)	356 (47)	584 (47)	3,564 (67)	<0.001
**If you answered 'yes' to the previous question: how did you change your social behaviour?**							
No physical contact with anyone	(N=1,374) 1,302 (94)	(N=506) 362 (82)	(N=657) 325 (51)	(N=342) 243 (74)	(N=576) 516 (93)	(N=3,455) 2,748 (82)	<0.001
No physical contact only with elderly and those with serious underlying medical conditions	(N=1,374) 1,200 (88)	(N=494) 292 (63)	(N=644) 393 (60)	(N=332) 272 (79)	(N=566) 516 (91)	(N=3,410) 2,673 (79)	<0.001
Going out only for essential needs	(N=1,374) 1,291 (94)	(N=525) 489 (95)	(N=681) 571 (83)	(N=346) 263 (82)	(N=562) 381 (71)	(N=3,488) 2,995 (87)	<0.001
Moving home to stay with parents/relatives	(N=1,374) 677 (54)	(N=489) 99 (26)	(N=627) 30 (8)	(N=326) 27 (6)	(N=552) 33 (5)	(N=3,368) 866 (30)	<0.001
Use of personal protection equipment (e.g. masks and gloves)	(N=1,374) 1,334 (96)	(N=527) 488 (95)	(N=651) 225 (33)	(N=339) 165 (55)	(N=564) 366 (67)	(N=3,455) 2,578 (76)	<0.001
Use of sanitizer products and alcohol	(N=1,374) 1,321 (95)	(N=529) 504 (96)	(N=685) 559 (83)	(N=350) 307 (91)	(N=569) 521 (94)	(N=3,507) 3,212 (92)	<0.001
**"I would comply with government enforced quarantine/ isolation/social distancing."**	N=1,476	N=827	N=1,009	N=712	N=1,034	N=5,058	<0.001
Agree	1,344 (92)	708 (86)	822 (80)	606 (78)	871 (75)	4,351 (83)	
Neither agree nor disagree	92 (5)	18 (0)	48 (4)	36 (7)	68 (14)	262 (6)	
Disagree	40 (3)	101 (14)	139 (15)	70 (15)	95 (11)	445 (10)	
**"I would enter voluntary quarantine/isolation/social distancing for social/self-responsibility."**	N=1,476	N=827	N=1,009	N=712	N=1,034	N=5,058	<0.001
Agree	1,354 (92)	674 (81)	815 (78)	566 (76)	838 (76)	4,247 (82)	
Neither agree nor disagree	100 (7)	48 (4)	50 (5)	59 (10)	91 (13)	348 (8)	
Disagree	22 (1)	105 (15)	144 (17)	87 (14)	105 (11)	463 (10)	
**How much do you agree with quarantine/isolation/social distancing? "It is a necessary strategy to help control COVID-19."**	N=1,476	N=827	N=1,009	N=712	N=1,034	N=5,058	<0.001
Agree	1,383 (94)	739 (88)	853 (83)	608 (80)	846 (74)	4,429 (85)	
Neither agree nor disagree	65 (4)	12 (0)	27 (3)	28 (5)	76 (11)	208 (5)	
Disagree	28 (2)	76 (12)	129 (14)	76 (15)	112 (15)	421 (10)	

Suppl. Table 24 Breakdown of behavioural changes and acceptance of government public health measures by country and gender

M = male; F = female; O = other/prefer not to say. Values in cells are n (weighted %) of respondents who replied 'yes'.

**Variable and categories**	**Thailand**			**Malaysia**			**UK**			**Italy**			**Slovenia**			**Total**			
**Gender**	**M**	**F**	**O**	**M**	**F**	**O**	**M**	**F**	**O**	**M**	**F**	**O**	**M**	**F**	**O**	**M**	**F**	**O**	**P-value (for total M vs F)**
	N=704	N=766	N=6	N=298	N=525	N=4	N=426	N=572	N=11	N=222	N=490	N=0	N=366	N=662	N=6	N=2,016	N=3,015	N=27	
**Did you change your social behaviour before the implementation of government restrictions?**	660 (94)	709 (92)	5 (83)	184 (60)	351 (68)	3 (75)	288 (64)	415 (71)	9 (82)	99 (43)	257 (52)		179 (42)	402 (51)	3 (50)	1,410 (65)	2,134 (70)	20 (74)	0.039
**If you answered 'yes' to the previous question: how did you change your social behaviour?**																			
No physical contact with anyone	(N=660) 626 (93)	(N=709) 671 (95)	(N=5) 5 (100)	(N=173) 122 (75)	(N=330) 237 (87)	(N=3) 3 (100)	(N=271) 141 (51)	(N=379) 181 (50)	(N=7) 3 (43)	(N=94) 63 (68)	(N=248) 180 (78)		(N=175) 162 (94)	(N=398) 351 (892	(N=3) 3 (100)	(N=1,373) 1,114 (80)	(N=2,064) 1,620 (83)	(N=18) 14 (78)	0.227
No physical contact only with elderly and those with serious underlying medical conditions	(N=660) 584 (88)	(N=709) 611 (89)	(N=5) 5 (100)	(N=170) 104 (59)	(N=321) 186 (67)	(N=3) 2 (67)	(N=268) 148 (58)	(N=370) 243 (62)	(N=6) 2 (33)	(N=90) 75 (75)	(N=242) 197 (81)		(N=171) 152 (88)	(N=392) 361 (94)	(N=3) 3 (100)	(N=1,359) 1,063 (77)	(N=2,034) 1,598 (81)	(N=17) 12 (71)	0.124
Going out only for essential needs	(N=660) 612 (93)	(N=709) 674 (94)	(N=5) 5 (100)	(N=177) 164 (91)	(N=345) 322 (99)	(N=3) 3 (100)	(N=277) 234 (84)	(N=396) 330 (82)	(N=8) 7 (88)	(N=95) 71 (84)	(N=251) 192 (81)		(N=172) 113 (65)	(N=387) 265 (76)	(N=3) 3 (100)	(N=1,381) 1,194 (87)	(N=2,088) 1,783 (88)	(N=19) 18 (95)	0.327
Moving home to stay with parents/relatives	(N=660) 359 (59)	(N=709) 316 (49)	(N=5) 2 (40)	(N=167) 39 (27)	(N=319) 59 (24)	(N=3) 1 (33)	(N=267) 8 (3)	(N=354) 22 (11)	(N=6) 0 (0)	(N=91) 7 (3)	(N=235) 20 (9)		(N=167) 11 (3)	(N=382) 21 (6)	(N=3) 1 (33)	(N=1,352) 424 (32)	(N=1,999) 438 (28)	(N=17) 4 (24)	0.207
Use of personal protection equipment (e.g. masks and gloves)	(N=660) 639 (97)	(N=709) 690 (95)	(N=5) 5 (100)	(N=178) 160 (96)	(N=346) 325 (95)	(N=3) 3 (100)	(N=272) 101 (33)	(N=371) 121 (33)	(N=8) 3 (38)	(N=93) 38 (59)	(N=246) 127 (52)		(N=173) 122 (73)	(N=388) 241 (63)	(N=3) 3 (100)	(N=1,376) 1,060 (78)	(N=2,060) 1,504 (74)	(N=19) 14 (74)	0.079
Use of sanitizer products and alcohol	(N=660) 628 (95)	(N=709) 688 (95)	(N=5) 5 (100)	(N=178) 167 (96)	(N=348) 334 (96)	(N=3) 3 (100)	(N=278) 223 (80)	(N=398) 329 (85)	(N=9) 7 (78)	(N=96) 80 (92)	(N=254) 227 (91)		(N=173) 164 (94)	(N=393) 354 (94)	(N=3) 3 (100)	(N=1,385) 1,262 (92)	(N=2,102) 1,932 (93)	(N=20) 18 (90)	0.474
**"I would comply with government enforced quarantine/ isolation/social distancing."**	N=704	N=766	N=6	N=298	N=525	N=4	N=426	N=572	N=11	N=222	N=490		N=366	N=662	N=6	N=2,016	N=3,015	N=27	0.631
Agree	636 (92)	705 (93)	3 (50)	262 (93)	442 (78)	4 (100)	334 (76)	480 (85)	8 (73)	176 (69)	430 (86)		295 (75)	571 (75)	5 (83)	1,703 (82)	2,628 (84)	20 (74)	
Neither agree nor disagree	49 (6)	40 (4)	3 (50)	9 (1)	9 (0)	0 (0)	26 (6)	19 (3)	3 (27)	14 (10)	22 (5)		24 (10)	44 (17)	0 (0)	122 (6)	134 (6)	6 (22)	
Disagree	19 (2)	21 (3)	0 (0)	27 (7)	74 (22)	0 (0)	66 (18)	73 (12)	0 (0)	32 (21)	38 (9)		47 (15)	47 (8)	1 (17)	191 (11)	253 (10)	1 (4)	

**"I would enter voluntary quarantine/isolation/social distancing for social/self-responsibility."**	N=704	N=766	N=6	N=298	N=525	N=4	N=426	N=572	N=11	N=222	N=490		N=366	N=662	N=6	N=2,016	N=3,015	N=27	0.761
Agree	644 (91)	707 (92)	3 (50)	258 (93)	412 (68)	4 (100)	340 (78)	465 (78)	10 (91)	163 (67)	403 (85)		285 (76)	548 (77)	5 (83)	1,690 (83)	2,535 (81)	22 (81)	
Neither agree nor disagree	50 (8)	47 (7)	3 (50)	14 (1)	34 (8)	0 (0)	22 (5)	27 (5)	1 (9)	21 (14)	38 (6)		36 (9)	55 (15)	0 (0)	143 (7)	201 (8)	4 (15)	
Disagree	10 (1)	12 (1)	0 (0)	26 (6)	79 (25)	0 (0)	64 (17)	80 (16)	0 (0)	38 (19)	49 (9)		45 (15)	59 (8)	1 (17)	183 (10)	279 (10)	1 (4)	
**How much do you agree with quarantine/isolation/social distancing? "It is a necessary strategy to help control COVID-19."**	N=704	N=766	N=6	N=298	N=525	N=4	N=426	N=572	N=11	N=222	N=490		N=366	N=662	N=6	N=2,016	N=3,015	N=27	0.191
Agree	653 (93)	725 (95)	5 (83)	272 (93)	463 (83)	4 (100)	342 (77)	502 (88)	9 (82)	169 (68)	439 (91)		285 (75)	557 (74)	4 (67)	1,721 (83)	2,686 (87)	22 (81)	
Neither agree nor disagree	38 (5)	26 (3)	1 (17)	6 (0)	6 (0)	0 (0)	16 (4)	11 (3)	0 (0)	15 (9)	13 (2)		28 (7)	47 (15)	1 (17)	103 (5)	103 (5)	2 (7)	
Disagree	13 (1)	15 (2)	0 (0)	20 (6)	56 (17)	0 (0)	68 (19)	59 (10)	2 (18)	38 (23)	38 (8)		53 (18)	58 (12)	1 (17)	192 (12)	226 (9)	3 (11)	

Osterrieder A, *et al. BMJ Open* 2021; 11:e046863. doi: 10.1136/bmjopen-2020-046863

Suppl. Table 25 Breakdown of behavioural changes and acceptance of government public health measures by country and <u>education level</u>

P/S = primary or lower/secondary education; T = tertiary education. Values in cells are n (weighted %) of respondents who replied ‘yes’.

**Variable and Categories**	**Thailand**		**Malaysia**		**UK**		**Italy**		**Slovenia**		**Total**		
**Education level**	**P/S**	**T**	**P/S**	**T**	**P/S**	**T**	**P/S**	**T**	**P/S**	**T**	**P/S**	**T**	**P-value (for total)**
	N=909	N=567	N=82	N=745	N=247	N=762	N=217	N=495	N=202	N=832	N=1,657	N=3,401	
**Did you change your social behaviour before the implementation of government restrictions?**	849 (93)	525 (92)	52 (64)	486 (65)	147 (60)	565 (74)	99 (46)	257 (52)	99 (41)	485 (56)	1,246 (67)	2,318 (69)	0.369
**If you answered 'yes' to the previous question: how did you change your social behaviour?**													
No physical contact with anyone	(N=849) 816 (95)	(N=525) 486 (91)	(N=47) 41 (85)	(N=459) 321 (70)	(N=138) 80 (59)	(N=519) 245 (45)	(N=90) 67 (76)	(N=252) 176 (71)	(N=97) 92 (96)	(N=479) 424 (90)	(N=1,221) 1,096 (87)	(N=2,234) 1,652 (70)	<0.001
No physical contact only with elderly and those with serious underlying medical conditions	(N=849) 771 (90)	(N=525) 429 (81)	(N=43) 29 (64)	(N=451) 263 (59)	(N=131) 76 (58)	(N=513) 317 (61)	(N=87) 73 (77)	(N=245) 199 (82)	(N=91) 83 (93)	(N=475) 433 (90)	(N=1,201) 1,032 (81)	(N=2,209) 1,641 (74)	0.003
Going out only for essential needs	(N=849) 798 (94)	(N=525) 493 (92)	(N=49) 47 (96)	(N=476) 442 (93)	(N=143) 122 (84)	(N=538) 449 (82)	(N=93) 69 (84)	(N=253) 194 (79)	(N=93) 66 (75)	(N=469) 315 (67)	(N=1,227) 1,102 (90)	(N=2,261) 1,893 (82)	<0.001
Moving home to stay with parents/relatives	(N=849) 515 (58)	(N=525) 162 (32)	(N=42) 11 (26)	(N=447) 88 (23)	(N=131) 5 (8)	(N=496) 25 (8)	(N=84) 10 (6)	(N=242) 17 (6)	(N=91) 4 (3)	(N=461) 29 (6)	(N=1,197) 545 (37)	(N=2,171) 321 (15)	<0.001
Use of personal protection equipment (e.g. masks and gloves)	(N=849) 819 (96)	(N=525) 515 (98)	(N=49) 47 (96)	(N=478) 441 (91)	(N=136) 55 (35)	(N=515) 170 (32)	(N=89) 49 (59)	(N=250) 116 (47)	(N=94) 57 (67)	(N=470) 309 (68)	(N=1,217) 1,027 (82)	(N=2,238) 1,551 (62)	<0.001
Use of sanitizer products and alcohol	(N=849) 813 (95)	(N=525) 508 (97)	(N=48) 46 (96)	(N=481) 458 (95)	(N=142) 120 (83)	(N=543) 439 (81)	(N=94) 84 (94)	(N=256) 223 (87)	(N=96) 92 (96)	(N=473) 429 (92)	(N=1,229) 1,155 (94)	(N=2,278) 2,057 (89)	<0.001
**“I would comply with government enforced quarantine/ isolation/social distancing.”**	N=909	N=567	N=82	N=745	N=247	N=762	N=217	N=495	N=202	N=832	N=1,657	N=3,401	0.315
Agree	843 (93)	501 (87)	70 (85)	638 (87)	190 (77)	632 (83)	178 (75)	428 (84)	148 (68)	723 (87)	1,429 (82)	2,922 (85)	
Neither agree nor disagree	43 (4)	49 (10)	0 (0)	18 (3)	14 (5)	34 (4)	9 (7)	27 (7)	22 (19)	46 (6)	88 (7)	174 (6)	
Disagree	23 (3)	17 (3)	12 (15)	89 (11)	43 (18)	96 (13)	30 (17)	40 (9)	32 (14)	63 (7)	140 (11)	305 (9)	
**“I would enter voluntary quarantine/isolation/social distancing for social/self-responsibility.”**	N=909	N=567	N=82	N=745	N=247	N=762	N=217	N=495	N=202	N=832	N=1,657	N=3,401	0.370
Agree	842 (92)	512 (89)	65 (80)	609 (83)	180 (73)	635 (83)	165 (75)	401 (80)	151 (72)	687 (82)	1,403 (81)	2,844 (84)	
Neither agree nor disagree	55 (7)	45 (10)	3 (4)	45 (6)	17 (6)	33 (4)	24 (11)	35 (7)	24 (15)	67 (9)	123 (8)	225 (7)	
Disagree	12 (1)	10 (2)	14 (16)	91 (11)	50 (21)	94 (13)	28 (14)	59 (13)	27 (13)	78 (9)	131 (11)	332 (10)	
**How much do you agree with quarantine/isolation/social distancing? “It is a necessary strategy to help control COVID-19.”**	N=909	N=567	N=82	N=745	N=247	N=762	N=217	N=495	N=202	N=832	N=1,657	N=3,401	0.304
Agree	858 (95)	525 (91)	72 (88)	667 (90)	201 (80)	652 (85)	179 (78)	429 (84)	145 (768	701 (85)	1,455 (84)	2,974 (87)	
Neither agree nor disagree	34 (4)	31 (7)	0 (0)	12 (2)	8 (4)	19 (3)	6 (5)	22 (5)	23 (14)	53 (6)	71 (5)	137 (5)	
Disagree	17 (2)	11 (2)	10 (12)	66 (8)	38 (17)	91 (12)	32 (17)	44 (10)	34 (19)	78 (9)	131 (11)	290 (9)	

Suppl. Table 26 Breakdown of behavioural changes and acceptance of government public health measures by age group

Values in cells are n (weighted %) of respondents who replied 'yes'.

**Variable and categories**	**Thailand**			**Malaysia**			**UK**			**Italy**			**Slovenia**			**Total**			
**Age group**	**18-34**	**35-64**	**65+**	**18-34**	**35-64**	**65+**	**18-34**	**35-64**	**65+**	**18-34**	**35-64**	**65+**	**18-34**	**35-64**	**65+**	**18-34**	**35-64**	**65+**	**P-value (for total)**
	N=223	N=1,152	N=101	N=350	N=442	N=35	N=140	N=616	N=253	N=272	N=383	N=57	N=308	N=676	N=50	N=1,293	N=3,269	N=496	
**Did you change your social behaviour before the implementation of government restrictions?**	202 (92)	1,079 (94)	93 (93)	233 (63)	287 (71)	18 (37)	104 (71)	448 (69)	160 (61)	124 (44)	202 (44)	30 (57)	178 (54)	386 (53)	20 (25)	841 (70)	2,402 (70)	321 (57)	0.004
**If you answered 'yes' to the previous question: how did you change your social behaviour?**																			
No physical contact with anyone	(N=202) 180 (91)	(N=1,079) 1,037 (96)	(N=93) 85 (90)	(N=225) 156 (84)	(N=265) 193 (80)	(N=16) 13 (81)	(N=99) 35 (43)	(N=412) 200 (51)	(N=146) 90 (61)	(N=120) 79 (72)	(N=196) 143 (74)	(N=26) 21 (75)	(N=176) 151 (87)	(N=380) 345 (94)	(N=20) 20 (100)	(N=822) 601 (78)	(N=2,332) 1,918 (84)	(N=301) 229 (82)	0.204
No physical contact only with elderly and those with serious underlying medical conditions	(N=202) 168 (88)	(N=1,079) 956 (90)	(N=93) 76 (83)	(N=218) 127 (65)	(N=261) 158 (61)	(N=15) 7 (73)	(N=98) 60 (60)	(N=416) 271 (65)	(N=130) 62 (46)	(N=120) 100 (89)	(N=187) 150 (80)	(N=25) 22 (69)	(N=174) 163 (90)	(N=374) 340 (92)	(N=18) 13 (87)	(N=812) 618 (78)	(N=2,317) 1,875 (81)	(N=281) 180 (73)	0.152
Going out only for essential needs	(N=202) 186 (94)	(N=1,079) 1,022 (95)	(N=93) 83 (89)	(N=230) 212 (98)	(N=278) 262 (94)	(N=17) 15 (82)	(N=102) 79 (76)	(N=427) 362 (86)	(N=152) 130 (86)	(N=121) 79 (68)	(N=198) 159 (79)	(N=27) 25 (99)	(N=174) 102 (55)	(N=370) 266 (75)	(N=18) 13 (87)	(N=829) 658 (85)	(N=2,352) 2,071 (88)	(N=307) 266 (89)	0.153
Moving home to stay with parents/relatives	(N=202) 88 (59)	(N=1,079) 556 (56)	(N=93) 33 (34)	(N=219) 65 (38)	(N=256) 32 (16)	(N=14) 2 (22)	(N=98) 21 (21)	(N=398) 8 (2)	(N=131) 1 (2)	(N=120) 16 (11)	(N=184) 11 (7)	(N=22) 0 (0)	(N=172) 16 (8)	(N=363) 17 (4)	(N=17) 0 (0)	(N=811) 206 (37)	(N=2,280) 624 (29)	(N=277) 36 (17)	<0.001
Use of personal protection equipment (e.g. masks and gloves)	(N=202) 198 (98)	(N=1,079) 1,050 (97)	(N=93) 86 (90)	(N=230) 212 (93)	(N=279) 262 (99)	(N=18) 14 (80)	(N=100) 23 (20)	(N=417) 157 (40)	(N=134) 45 (35)	(N=121) 48 (39)	(N=191) 100 (54)	(N=27) 17 (69)	(N=174) 88 (52)	(N=371) 260 (68)	(N=19) 18 (97)	(N=827) 569 (72)	(N=2,337) 1,829 (79)	(N=291) 180 (74)	0.067
Use of sanitizer products and alcohol	(N=202) 197 (96)	(N=1,079) 1,037 (96)	(N=93) 87 (91)	(N=230) 218 (94)	(N=281) 271 (99)	(N=18) 15 (81)	(N=102) 88 (84)	(N=436) 352 (82)	(N=147) 119 (84)	(N=122) 103 (84)	(N=199) 177 (90)	(N=29) 27 (99)	(N=174) 157 (92)	(N=377) 346 (94)	(N=18) 18 (100)	(N=830) 763 (92)	(N=2,372) 2,183 (93)	(N=305) 266 (91)	0.613
**"I would comply with government enforced quarantine/ isolation/social distancing."**	N=223	N=1,152	N=101	N=350	N=442	N=35	N=140	N=616	N=253	N=272	N=383	N=57	N=308	N=676	N=50	N=1,293	N=3,269	N=496	0.003
Agree	189 (90)	1,058 (92)	97 (96)	307 (82)	371 (88)	30 (91)	120 (85)	493 (78)	209 (80)	247 (88)	311 (77)	48 (72)	272 (85)	559 (75)	40 (65)	1,135 (86)	2,792 (83)	424 (80)	
Neither agree nor disagree	28 (8)	63 (5)	1 (1)	7 (1)	11 (1)	0 (0)	3 (1)	33 (6)	12 (5)	7 (2)	24 (5)	5 (14)	16 (7)	44 (8)	8 (34)	61 (4)	175 (5)	26 (13)	
Disagree	6 (2)	31 (3)	3 (3)	36 (18)	60 (11)	5 (9)	17 (14)	90 (17)	32 (14)	18 (10)	48 (17)	4 (14)	20 (8)	73 (17)	2 (1)	97 (10)	302 (12)	46 (8)	

**"I would enter voluntary quarantine/isolation/social distancing for social/self-responsibility."**	N=223	N=1,152	N=101	N=350	N=442	N=35	N=140	N=616	N=253	N=272	N=383	N=57	N=308	N=676	N=50	N=1,293	N=3,269	N=496	0.327
Agree	188 (86)	1,068 (93)	98 (96)	294 (79)	353 (86)	27 (68)	114 (79)	497 (78)	204 (78)	211 (70)	306 (75)	49 (84)	247 (80)	550 (75)	41 (74)	1,054 (80)	2,774 (83)	419 (82)	
Neither agree nor disagree	33 (13)	64 (5)	3 (4)	23 (7)	23 (1)	2 (9)	6 (4)	30 (5)	14 (7)	28 (15)	28 (8)	3 (10)	28 (9)	57 (11)	6 (20)	118 (9)	202 (6)	28 (10)	
Disagree	2 (1)	20 (2)	0 (0)	33 (15)	66 (13)	6 (24)	20 (17)	89 (17)	35 (15)	33 (16)	49 (17)	5 (6)	33 (11)	69 (13)	3 (7)	121 (11)	293 (11)	49 (8)	
**How much do you agree with quarantine/isolation/social distancing? "It is a necessary strategy to help control COVID-19."**	N=223	N=1,152	N=101	N=350	N=442	N=35	N=140	N=616	N=253	N=272	N=383	N=57	N=308	N=676	N=50	N=1,293	N=3,269	N=496	0.271
Agree	203 (93)	1,083 (94)	97 (96)	313 (85)	393 (89)	33 (100)	120 (83)	521 (83)	212 (82)	243 (86)	315 (78)	50 (79)	254 (79)	549 (76)	43 (67)	1,133 (86)	2,861 (85)	435 (82)	
Neither agree nor disagree	18 (7)	45 (4)	2 (2)	5 (0)	6 (0)	1 (0)	3 (3)	16 (3)	8 (4)	10 (4)	14 (3)	4 (11)	28 (12)	45 (7)	3 (18)	64 (5)	126 (4)	18 (8)	
Disagree	2 (0)	24 (2)	2 (2)	32 (15)	43 (11)	1 (0)	17 (14)	79 (15)	33 (14)	19 (10)	54 (19)	3 (10)	26 (10)	82 (17)	4 (15)	96 (9)	282 (11)	43 (10)	

Osterrieder A, *et al. BMJ Open* 2021; 11:e046863. doi: 10.1136/bmjopen-2020-046863

Suppl. Table 27 Breakdown of behavioural changes and acceptance of government public health measures by self-reported level of understanding of COVID-19

H = high/very high/expert level; S = some; N = a little/none at all. Values in cells are n (weighted %) of respondents who replied ‘yes’.

**Variable and categories**	**Thailand**			**Malaysia**			**UK**			**Italy**			**Slovenia**			**Total**			
**Self-reported level of understanding of COVID-19**	**H**	**S**	**N**	**H**	**S**	**N**	**H**	**S**	**N**	**H**	**S**	**N**	**H**	**S**	**N**	**H**	**S**	**N**	**P-value (for total)**
	N=965	N=459	N=52	N=435	N=359	N=33	N=647	N=336	N=26	N=368	N=324	N=20	N=713	N=279	N=42	N=3,128	N=1,757	N=173	
**Did you change your social behaviour before the implementation of government restrictions?**	898 (94)	430 (92)	46 (91)	285 (64)	232 (66)	21 (58)	468 (69)	232 (66)	12 (68)	200 (52)	146 (43)	10 (60)	429 (52)	137 (37)	18 (46)	2,280 (70)	1,177 (64)	107 (65)	0.091
**If you answered 'yes' to the previous question: how did you change your social behaviour?**																			
No physical contact with anyone	(N=898) 849 (94)	(N=430) 411 (95)	(N=46) 42 (9187)	(N=272) 204 (90)	(N=214) 143 (73)	(N=20) 15 (69)	(N=428) 221 (53)	(N=217) 99 (47)	(N=12) 5 (52)	(N=194) 137 (78)	(N=138) 99 (67)	(N=10) 7 (88)	(N=423) 380 (95)	(N=135) 119 (87)	(N=18) 17 (96)	(N=2,215) 1,791 (85)	(N=1,134) 871 (77)	(N=106) 86 (78)	0.033
No physical contact only with elderly and those with serious underlying medical conditions	(N=898) 765 (87)	(N=430) 394 (92)	(N=46) 41 (87)	(N=266) 162 (63)	(N=209) 119 (60)	(N=19) 11 (74)	(N=417) 261 (61)	(N=215) 128 (59)	(N=12) 4 (49)	(N=192) 163 (85)	(N=130) 101 (67)	(N=10) 8 (94)	(N=418) 379 (91)	(N=131) 122 (92)	(N=17) 15 (95)	(N=2,191) 1,730 (80)	(N=1,115) 864 (77)	(N=104) 79 (79)	0.744
Going out only for essential needs	(N=898) 844 (93)	(N=430) 405 (95)	(N=46) 42 (87)	(N=280) 266 (99)	(N=225) 205 (89)	(N=20) 18 (99)	(N=444) 381 (86)	(N=225) 182 (80)	(N=12) 8 (66)	(N=196) 145 (80)	(N=140) 109 (83)	(N=10) 9 (95)	(N=415) 283 (72)	(N=129) 87 (73)	(N=18) 11 (60)	(N=2,233) 1,919 (88)	(N=1,149) 988 (87)	(N=106) 88 (84)	0.711
Moving home to stay with parents/relatives	(N=898) 345 (45)	(N=430) 298 (67)	(N=46) 34 (73)	(N=261) 45 (24)	(N=209) 48 (25)	(N=19) 6 (40)	(N=404) 17 (5)	(N=212) 12 (10)	(N=11) 1 (24)	(N=189) 17 (6)	(N=127) 9 (7)	(N=10) 1 (10)	(N=405) 19 (3)	(N=129) 14 (9)	(N=18) 0 (0)	(N=2,157) 443 (25)	(N=1,107) 381 (36)	(N=104) 42 (42)	<0.001
Use of personal protection equipment (e.g. masks and gloves)	(N=898) 874 (97)	(N=430) 418 (96)	(N=46) 42 (81)	(N=280) 266 (99)	(N=227) 203 (90)	(N=20) 19 (99)	(N=421) 153 (38)	(N=218) 68 (28)	(N=12) 4 (17)	(N=194) 90 (46)	(N=135) 69 (66)	(N=10) 6 (66)	(N=416) 289 (71)	(N=130) 71 (59)	(N=18) 6 (38)	(N=2,209) 1,672 (78)	(N=1,140) 829 (74)	(N=106) 77 (69)	0.172
Use of sanitizer products and alcohol	(N=898) 863 (96)	(N=430) 416 (95)	(N=46) 42 (81)	(N=281) 270 (99)	(N=228) 215 (91)	(N=20) 19 (100)	(N=447) 374 (85)	(N=226) 179 (85)	(N=12) 6 (30)	(N=198) 170 (90)	(N=142) 129 (93)	(N=10) 8 (94)	(N=418) 385 (95)	(N=133) 125 (95)	(N=18) 11 (70)	(N=2,242) 2,062 (94)	(N=1,159) 1,064 (92)	(N=106) 86 (78)	<0.001

**"I would comply with government enforced quarantine/ isolation/social distancing."**	N=965	N=459	N=52	N=435	N=359	N=33	N=647	N=336	N=26	N=368	N=324	N=20	N=713	N=279	N=42	N=3,128	N=1,757	N=173	0.370
Agree	903 (95)	402 (88)	39 (81)	378 (93)	305 (79)	25 (76)	511 (79)	291 (83)	20 (87)	303 (76)	284 (79)	19 (97)	607 (75)	232 (75)	32 (70)	2,702 (85)	1,514 (82)	135 (80)	
Neither agree nor disagree	39 (3)	44 (9)	9 (10)	5 (0)	9 (1)	4 (1)	29 (3)	18 (6)	1 (2)	17 (4)	18 (11)	1 (3)	45 (16)	19 (10)	4 (7)	135 (6)	108 (7)	19 (4)	
Disagree	23 (2)	13 (3)	4 (9)	52 (7)	45 (20)	4 (23)	107 (18)	27 (12)	5 (11)	48 (21)	22 (10)	0 (0)	61 (9)	28 (15)	6 (24)	291 (10)	135 (11)	19 (16)	
**"I would enter voluntary quarantine/isolation/social distancing for social/self-responsibility."**	N=965	N=459	N=52	N=435	N=359	N=33	N=647	N=336	N=26	N=368	N=324	N=20	N=713	N=279	N=42	N=3,128	N=1,757	N=173	0.091
Agree	909 (95)	401 (85)	44 (90)	357 (86)	294 (76)	23 (75)	516 (78)	284 (80)	15 (60)	293 (78)	258 (74)	15 (91)	587 (78)	219 (74)	32 (69)	2,662 (84)	1,456 (79)	129 (77)	
Neither agree nor disagree	41 (4)	51 (13)	8 (10)	21 (1)	21 (10)	6 (1)	29 (5)	18 (5)	3 (8)	27 (8)	30 (12)	2 (6)	58 (14)	26 (9)	7 (23)	176 (6)	146 (10)	26 (8)	
Disagree	15 (1)	7 (1)	0 (0)	57 (13)	44 (14)	4 (23)	102 (17)	34 (15)	8 (32)	48 (15)	36 (13)	3 (4)	68 (9)	34 (17)	3 (7)	290 (9)	155 (11)	18 (15)	
**How much do you agree with quarantine/isolation/social distancing? "It is a necessary strategy to help control COVID-19."**	N=965	N=459	N=52	N=435	N=359	N=33	N=647	N=336	N=26	N=368	N=324	N=20	N=713	N=279	N=42	N=3,128	N=1,757	N=173	0.688
Agree	920 (96)	418 (91)	45 (90)	392 (91)	319 (85)	28 (86)	540 (82)	293 (83)	20 (85)	304 (77)	285 (82)	19 (82)	589 (73)	226 (78)	31 (72)	2,745 (85)	1,541 (85)	143 (84)	
Neither agree nor disagree	26 (2)	33 (8)	6 (8)	5 (0)	5 (0)	2 (1)	16 (3)	10 (3)	1 (2)	10 (2)	18 (9)	0 (0)	45 (12)	27 (9)	4 (7)	102 (4)	93 (6)	13 (4)	
Disagree	19 (1)	8 (2)	1 (2)	38 (9)	35 (15)	3 (13)	91 (15)	33 (13)	5 (13)	54 (21)	21 (9)	1 (18)	79 (16)	26 (13)	7 (21)	281 (11)	123 (10)	17 (12)	

Osterrieder A, *et al. BMJ Open* 2021; 11:e046863. doi: 10.1136/bmjopen-2020-046863

Suppl. Table 28 Breakdown of self-reported level of understanding of COVID-19 by country

Values in cells are n (weighted %) of respondents who replied ‘yes’.

Variable and categories	Thailand	Malaysia	UK	Italy	Slovenia	Total	P-value
	N=1,476	N=827	N=1,009	N=712	N=1,034	N=5,058	<0.001
**High/very high/expert level understanding**	965 (63)	435 (51)	647 (59)	368 (47)	713 (66)	3,128 (59)	
**Some understanding**	459 (33)	359 (38)	336 (38)	324 (50)	279 (30)	1,757 (36)	
**A little/none at all**	52 (4)	33 (11)	26 (4)	20 (3)	42 (4)	173 (5)	

Suppl. Table 29 Breakdown of self-reported level of understanding of COVID-19 by demographic characteristics

H = high/very high/expert level; S = some; N = a little/none at all. Values in cells are n (weighted %) of respondents who replied 'yes'.

**Variable and categories**	**Thailand**			**Malaysia**			**UK**			**Italy**			**Slovenia**			**Total**			
**Self-reported understanding of COVID-19**	**H**	**S**	**N**	**H**	**S**	**N**	**H**	**S**	**N**	**H**	**S**	**N**	**H**	**S**	**N**	**H**	**S**	**N**	**P-value (for total)**
**Gender**																			0.058
Male	458 (65)	224 (31)	22 (4)	153 (55)	130 (30)	15 (15)	280 (61)	134 (35)	12 (4)	130 (51)	87 (46)	5 (3)	269 (64)	84 (31)	13 (5)	1,290 (60)	659 (34)	67 (6)	
Female	504 (61)	232 (35)	30 (4)	280 (47)	228 (46)	17 (7)	358 (56)	200 (40)	14 (3)	238 (44)	237 (53)	15 (3)	439 (68)	194 (29)	29 (3)	1,819 (57)	1,091 (39)	105 (4)	
Other/prefer not to say	3 (50)	3 (50)	0 (0)	2 (50)	1 (25)	1 (25)	9 (82)	2 (18)	0 (0)				5 (83)	1 (17)	0 (0)	19 (70)	7 (26)	1 (4)	
**Age group**																			0.033
18-34	143 (62)	69 (34)	11 (4)	170 (48)	167 (48)	13 (9)	74 (44)	58 (48)	8 (8)	119 (39)	143 (57)	10 (5)	186 (59)	106 (35)	16 (6)	692 (52)	543 (41)	58 (6)	
35-64	746 (62)	371 (35)	35 (3)	244 (54)	179 (32)	19 (14)	411 (67)	193 (32)	12 (2)	220 (54)	153 (42)	10 (4)	492 (69)	158 (27)	26 (5)	2,113 (62)	1,054 (33)	102 (5)	
65+	76 (68)	19 (25)	6 (7)	21 (52)	13 (42)	1 (6)	162 (59)	85 (39)	6 (2)	29 (42)	28 (58)	0 (0)	35 (68)	15 (32)	0 (0)	323 (60)	160 (38)	13 (3)	
**Education level**																			<0.001
Primary or lower/secondary	537 (60)	341 (36)	31 (4)	42 (51)	30 (36)	10 (13)	140 (52)	101 (44)	6 (4)	92 (43)	114 (53)	11 (4)	124 (63)	67 (33)	11 (4)	935 (56)	653 (39)	69 (6)	
Tertiary	428 (74)	118 (22)	21 (4)	393 (51)	329 (46)	23 (3)	507 (64)	235 (32)	20 (3)	276 (58)	210 (41)	9 (2)	589 (71)	212 (26)	31 (3)	2,193 (66)	1,104 (31)	104 (3)	
**Healthcare worker status**																			0.001
Healthcare worker	172 (72)	59 (26)	8 (3)	128 (49)	79 (50)	6 (1)	90 (76)	24 (21)	4 (3)	45 (67)	18 (29)	1 (4)	291 (78)	44 (21)	6 (1)	726 (70)	224 (28)	25 (2)	
Non-healthcare worker	793 (61)	400 (33)	44 (4)	307 (52)	280 (35)	27 (13)	557 (57)	312 (39)	22 (4)	323 (46)	306 (50)	19 (3)	422 (63)	235 (32)	36 (5)	2,402 (57)	1,533 (38)	148 (5)	

Osterrieder A, *et al. BMJ Open* 2021; 11:e046863. doi: 10.1136/bmjopen-2020-046863

Suppl. Table 30 Breakdown of self-reported understanding of public health measures by self-reported level of understanding of COVID-19

(H = high/very high/expert level; S = some; N = a little/none at all). Values in cells are n (weighted %) of respondents who replied 'yes'.

**Variable and categories**	**Thailand**			**Malaysia**			**UK**			**Italy**			**Slovenia**			**Total**			
**Self-reported level of understanding of COVID-19**	**H**	**S**	**N**	**H**	**S**	**N**	**H**	**S**	**N**	**H**	**S**	**N**	**H**	**S**	**N**	**H**	**S**	**N**	**P-value**
**How would you rate your level of understanding of the current quarantine/isolation/social distancing requirements for COVID-19?**	N=965	N=459	N=52	N=435	N=359	N=33	N=647	N=336	N=26	N=368	N=324	N=20	N=713	N=279	N=42	N=3,128	N=1,757	N=173	<0.001
H	855 (89)	116 (23)	19 (24)	399 (89)	193 (52)	9 (21)	532 (81)	182 (57)	8 (21)	338 (93)	213 (71)	7 (36)	652 (89)	212 (59)	24 (46)	2,776 (88)	916 (50)	67 (27)	
S	102 (10)	323 (71)	11 (12)	31 (7)	157 (39)	15 (52)	98 (15)	129 (35)	11 (46)	22 (5)	106 (28)	10 (38)	50 (10)	55 (32)	12 (44)	303 (10)	770 (43)	59 (39)	
N	8 (1)	20 (6)	22 (64)	5 (4)	9 (9)	9 (27)	17 (4)	25 (8)	7 (33)	8 (2)	5 (1)	3 (26)	11 (1)	12 (9)	6 (10)	49 (2)	71 (6)	47 (34)	

Suppl. Table 31 What were the three most common ways people received communication on COVID-19, and what are the three most preferred ways to receive COVID-19 communications? Breakdown by country

Values in cells are n (weighted %) of respondents who replied 'yes'.

Variable and categories	Thailand	Malaysia	UK	Italy	Slovenia	Total	P-value
	N=1,476	N=827	N=1,009	N=712	N=1,034	N=5,058	
**How do/did you receive information about COVID-19?**							
Face-to-face (e.g. doctors or health workers)	1,096 (78)	275 (19)	155 (15)	276 (32)	413 (34)	2,215 (40)	<0.001
Traditional media (TV, radio, newspapers)	1,407 (95)	795 (93)	940 (93)	650 (85)	994 (95)	4,786 (93)	0.012
Print materials (leaflets, brochures)	803 (55)	256 (32)	403 (36)	119 (23)	479 (43)	2,060 (40)	<0.001
Online (websites, email)	1,101 (69)	779 (90)	918 (89)	651 (88)	964 (87)	4,413 (83)	<0.001
Social media and messenger apps	1,279 (83)	786 (95)	773 (77)	528 (75)	731 (66)	4,097 (79)	<0.001
Government/institution's web page	1,134 (74)	682 (75)	698 (70)	580 (79)	784 (60)	3,878 (71)	<0.001
WHO web page	367 (20)	550 (56)	380 (36)	334 (39)	397 (30)	2,028 (34)	<0.001
**How would you prefer to receive information about COVID-19?**							
Face-to-face (e.g doctors or health workers)	1,200 (83)	417 (44)	361 (36)	584 (77)	577 (55)	3,139 (61)	<0.001
Traditional media (TV, radio, newspapers)	1,347 (90)	759 (91)	648 (64)	467 (62)	806 (76)	4,027 (78)	<0.001
Print materials	893 (63)	340 (40)	418 (41)	149 (29)	481 (52)	2,281 (48)	<0.001
Online (websites, email)	1,105 (71)	742 (88)	812 (75)	473 (71)	856 (79)	3,988 (76)	<0.001
Social media and messenger apps	1,245 (82)	659 (85)	330 (31)	292 (50)	470 (50)	2,996 (61)	<0.001
Government/institution's web page	1,181 (77)	731 (86)	741 (74)	605 (77)	845 (71)	4,103 (77)	0.009
WHO web page	586 (36)	703 (82)	609 (58)	531 (64)	678 (55)	3,107 (56)	<0.001

Suppl. Table 32 What were the three most common ways people received communications on COVID-19, and what are the three most preferred ways to receive COVID-19 communications? Breakdown by country and gender

M = male; F = female; O = other/prefer not to say. Values in cells are n (weighted %) of respondents who replied 'yes'.

**Variable and categories**	**Thailand**			**Malaysia**			**UK**			**Italy**			**Slovenia**			**Total**			
**Gender**	**M**	**F**	**O**	**M**	**F**	**O**	**M**	**F**	**O**	**M**	**F**	**O**	**M**	**F**	**O**	**M**	**F**	**O**	**P-value (for total M vs F)**
	N=704	N=766	N=6	N=298	N=525	N=4	N=426	N=572	N=11	N=222	N=490	N=0	N=366	N=662	N=6	N=2,016	N=3,015	N=27	
**How do/did you receive information about COVID-19?**																			
Face-to-face	563 (81)	529 (75)	4 (67)	93 (17)	180 (21)	2 (50)	68 (16)	84 (14)	3 (27)	82 (29)	194 (34)		126 (31)	285 (37)	2 (33)	932 (40)	1,272 (41)	11 (41)	0.591
Traditional media (TV, radio, newspapers)	669 (94)	732 (96)	6 (100)	284 (92)	507 (93)	4 (100)	390 (92)	539 (95)	11 (100)	199 (82)	451 (88)		353 (98)	635 (93)	6 (100)	1,895 (92)	2,864 (94)	27 (100)	0.468
Print materials (leaflets, brochures)	398 (54)	402 (56)	3 (50)	94 (37)	162 (26)	0 (0)	171 (37)	227 (36)	5 (45)	31 (27)	88 (20)		168 (44)	307 (41)	4 (67)	862 (42)	1,186 (39)	12 (44)	0.265
Online (websites, email)	509 (69)	586 (69)	6 (100)	281 (92)	495 (89)	3 (75)	379 (87)	528 (91)	11 (100)	201 (85)	450 (90)		336 (84)	622 (90)	6 (100)	1,706 (82)	2,681 (84)	26 (96)	0.332
Social media and messenger apps	595 (84)	678 (82)	6 (100)	281 (96)	502 (94)	3 (75)	312 (74)	450 (79)	11 (100)	154 (70)	374 (80)		256 (66)	470 (67)	5 (83)	1,598 (78)	2,474 (80)	25 (93)	0.589
Government/institution's web page	540 (73)	589 (74)	5 (83)	246 (80)	432 (69)	4 (100)	282 (69)	409 (71)	7 (64)	170 (74)	410 (83)		260 (59)	518 (61)	6 (100)	1,498 (71)	2,358 (71)	22 (81)	0.881
WHO web page	150 (18)	214 (22)	3 (50)	173 (52)	374 (60)	3 (75)	136 (34)	239 (39)	5 (45)	81 (27)	253 (50)		108 (26)	286 (33)	3 (50)	648 (30)	1,366 (38)	14 (52)	0.003
**How would you prefer to receive information about COVID-19?**																			
Face-to-face	594 (85)	603 (82)	3 (50)	146 (39)	270 (50)	1 (25)	163 (36)	195 (37)	3 (27)	171 (75)	413 (79)		182 (53)	389 (57)	6 (100)	1,256 (59)	1,870 (63)	13 (48)	0.209
Traditional media (TV, radio, newspapers)	644 (89)	697 (91)	6 (100)	267 (91)	488 (92)	4 (100)	278 (66)	365 (63)	5 (45)	134 (57)	333 (67)		274 (76)	530 (77)	2 (33)	1,597 (77)	2,413 (79)	17 (63)	0.395
Print materials	446 (65)	442 (61)	5 (83)	115 (39)	223 (41)	2 (50)	177 (41)	237 (41)	4 (36)	46 (33)	103 (25)		165 (53)	314 (51)	2 (33)	949 (49)	1,319 (47)	13 (48)	0.408
Online (websites, email)	516 (70)	583 (71)	6 (100)	269 (92)	469 (83)	4 (100)	334 (71)	470 (78)	8 (73)	151 (72)	322 (70)		290 (74)	561 (84)	5 (83)	1,560 (75)	2,405 (77)	23 (85)	0.403
Social media and messenger apps	589 (84)	650 (80)	6 (100)	239 (85)	416 (87)	4 (100)	134 (29)	195 (34)	1 (9)	88 (52)	204 (48)		161 (43)	307 (57)	2 (33)	1,211 (60)	1,772 (63)	13 (48)	0.364
Government/institution's web page	575 (78)	601 (75)	5 (83)	270 (93)	457 (79)	4 (100)	293 (69)	440 (78)	8 (73)	181 (73)	424 (82)		278 (64)	561 (77)	6 (100)	1,597 (75)	2,483 (78)	23 (85)	0.335
WHO web page	248 (36)	334 (36)	4 (67)	242 (80)	457 (83)	4 (100)	234 (54)	370 (62)	5 (45)	143 (54)	388 (74)		209 (49)	466 (60)	3 (50)	1,076 (52)	2,015 (59)	16 (59)	0.020

Suppl. Table 33 What were the three most common ways people received communications on COVID-19, and what are the three most preferred ways to receive COVID-19 communications? Breakdown by country and age group

Values in cells are n (weighted %) of respondents who replied ‘yes’.

**Variable and categories**	**Thailand**			**Malaysia**			**UK**			**Italy**			**Slovenia**			**Total**			
**Age group**	**18-34**	**35-64**	**65+**	**18-34**	**35-64**	**65+**	**18-34**	**35-64**	**65+**	**18-34**	**35-64**	**65+**	**18-34**	**35-64**	**65+**	**18-34**	**35-64**	**65+**	**P-value (for total)**
	N=223	N=1,152	N=101	N=350	N=442	N=35	N=140	N=616	N=253	N=272	N=383	N=57	N=308	N=676	N=50	N=1,293	N=3,269	N=496	
**How do/did you receive information about COVID-19?**																			
Face-to-face	125 (68)	892 (82)	79 (82)	141 (20)	124 (16)	10 (23)	25 (17)	107 (17)	23 (8)	112 (37)	152 (34)	12 (23)	111 (32)	282 (30)	20 (48)	514 (37)	1,557 (42)	144 (40)	0.424
Traditional media (TV, radio, newspapers)	210 (94)	1,099 (95)	98 (96)	337 (89)	424 (95)	34 (100)	130 (93)	567 (92)	243 (97)	247 (92)	352 (90)	51 (70)	299 (98)	647 (96)	48 (91)	1,223 (93)	3,089 (94)	474 (90)	0.336
Print materials (leaflets, brochures)	107 (54)	652 (59)	44 (44)	104 (31)	146 (35)	6 (20)	34 (22)	258 (40)	111 (43)	34 (12)	71 (19)	14 (41)	140 (45)	319 (46)	20 (31)	419 (37)	1,446 (43)	195 (38)	0.106
Online (websites, email)	199 (84)	853 (71)	49 (35)	328 (86)	418 (94)	33 (91)	129 (89)	575 (92)	214 (82)	242 (90)	358 (89)	51 (82)	289 (93)	632 (91)	43 (74)	1,187 (87)	2,836 (85)	390 (69)	<0.001
Social media and messenger apps	206 (91)	1,008 (86)	65 (55)	329 (93)	424 (98)	33 (91)	104 (76)	485 (78)	184 (74)	214 (79)	274 (73)	40 (77)	243 (80)	462 (70)	26 (42)	1,096 (86)	2,653 (81)	348 (63)	<0.001
Government/institution’s web page	166 (73)	902 (78)	66 (61)	298 (71)	360 (81)	24 (61)	108 (77)	459 (74)	131 (53)	219 (73)	318 (81)	43 (78)	226 (68)	528 (71)	30 (29)	1,017 (72)	2,567 (77)	294 (54)	<0.001
WHO web page	100 (31)	256 (19)	11 (6)	260 (62	274 (53)	16 (39)	60 (45)	271 (40)	49 (18)	129 (39)	176 (38)	29 (42)	127 (39)	255 (30)	15 (19)	676 (44)	1,232 (33)	120 (22)	<0.001
**How would you prefer to receive information about COVID-19?**																			
Face-to-face	152 (77)	965 (87)	83 (84)	198 (53)	203 (34)	16 (53)	48 (33)	218 (37)	95 (39)	230 (78)	313 (80)	41 (71)	187 (57)	365 (53)	25 (59)	815 (59)	2,064 (61)	260 (62)	0.785
Traditional media (TV, radio, newspapers)	194 (85)	1,056 (91)	97 (93)	327 (90)	402 (91)	30 (99)	89 (65)	396 (64)	163 (64)	179 (60)	247 (58)	41 (72)	228 (73)	534 (75)	44 (83)	1,017 (78)	2,635 (78)	375 (80)	0.712
Print materials	118 (64)	720 (65)	55 (54)	143 (41)	179 (37)	18 (45)	40 (27)	256 (44)	122 (52)	43 (15)	88 (24)	18 (50)	149 (50)	308 (48)	24 (63)	493 (44)	1,551 (48)	237 (54)	0.073
Online (websites, email)	187 (83)	867 (73)	51 (41)	312 (87)	399 (91)	31 (77)	98 (59)	522 (84)	192 (74)	180 (74)	253 (68)	40 (75)	250 (79)	567 (83)	39 (71)	1,027 (78)	2,608 (79)	353 (66)	<0.001
Social media and messenger apps	196 (91)	986 (85)	63 (55)	285 (88)	349 (86)	25 (75)	34 (21)	219 (37)	77 (31)	105 (38)	156 (48)	31 (65)	134 (48)	317 (51)	19 (49)	754 (64)	2,027 (64)	215 (52)	0.005
Government/institution’s web page	177 (79)	936 (80)	68 (60)	323 (93)	381 (81)	27 (82)	108 (71)	468 (77)	165 (71)	235 (83)	325 (82)	45 (65)	252 (75)	557 (76)	36 (56)	1,095 (81)	2,667 (79)	341 (64)	<0.001
WHO web page	145 (55)	415 (31)	26 (20)	320 (92)	357 (72)	26 (77)	98 (65)	387 (60)	124 (46)	226 (79)	266 (64)	39 (53)	231 (73)	427 (59)	20 (26)	1,020 (72)	1,852 (53)	235 (39)	<0.001

Suppl. Table 34 What were the three most common ways people received communications on COVID-19, and what are the three most preferred ways to receive COVID-19 communications? Breakdown by country and education level

P/S = primary or lower/secondary education; T = tertiary education. Values in cells are n (weighted %) of respondents who replied ‘yes’.

Variable and categories	Thailand		Malaysia		UK		Italy		Slovenia		Total		
**Education level**	**P/S**	**T**	**P/S**	**T**	**P/S**	**T**	**P/S**	**T**	**P/S**	**T**	**P/S**	**T**	**P-value (for total)**
	N=909	N=567	N=82	N=745	N=247	N=762	N=217	N=495	N=202	N=832	N=1,657	N=3,401	
**How do/did you receive information about COVID-19?**													
Face-to-face	781 (83)	315 (55)	13 (14)	262 (37)	32 (14)	123 (16)	72 (28)	204 (39)	48 (29)	365 (43)	946 (43)	1,269 (35)	<0.001
Traditional media (TV, radio, newspapers)	865 (95)	542 (95)	76 (92)	719 (97)	234 (95)	706 (92)	192 (82)	458 (93)	196 (95)	798 (96)	1,563 (92)	3,223 (94)	0.155
Print materials (leaflets, brochures)	547 (57)	256 (45)	26 (32)	230 (31)	90 (34)	313 (38)	39 (26)	80 (16)	91 (40)	388 (47)	793 (42)	1,267 (38)	0.062
Online (websites, email)	605 (65)	496 (87)	74 (89)	705 (95)	212 (85)	706 (93)	190 (85)	461 (93)	179 (83)	785 (94)	1,260 (79)	3,153 (92)	<0.001
Social media and messenger apps	757 (81)	522 (91)	78 (95)	708 (94)	196 (79)	577 (75)	173 (78)	355 (70)	150 (65)	581 (68)	1,354 (80)	2,743 (77)	0.146
Government/institution’s web page	689 (73)	445 (78)	59 (73)	623 (85)	171 (70)	527 (71)	166 (77)	414 (81)	123 (49)	661 (78)	1,208 (69)	2,670 (77)	<0.001
WHO web page	139 (15)	228 (42)	44 (53)	506 (67)	68 (30)	312 (42)	84 (35)	250 (49)	59 (24)	338 (39)	394 (29)	1,634 (44)	<0.001
**How would you prefer to receive information about COVID-19?**													
Face-to-face	806 (87)	394 (68)	36 (42)	381 (53)	104 (39)	257 (34)	170 (75)	414 (81)	111 (56)	466 (54)	1,227 (65)	1,912 (53)	<0.001
Traditional media (TV, radio, newspapers)	830 (90)	517 (90)	75 (91)	684 (92)	149 (63)	499 (66)	133 (60)	334 (68)	145 (74)	661 (80)	1,332 (79)	2,695 (76)	0.100
Print materials	608 (66)	285 (49)	35 (40)	305 (40)	126 (47)	292 (37)	48 (32)	101 (21)	105 (57)	376 (45)	922 (52)	1,359 (39)	<0.001
Online (websites, email)	632 (68)	473 (82)	71 (87)	671 (90)	186 (68)	626 (81)	156 (74)	317 (64)	160 (77)	696 (83)	1,205 (74)	2,783 (80)	<0.001
Social media and messenger apps	753 (81)	492 (86)	72 (87)	587 (79)	90 (32)	240 (31)	106 (55)	186 (38)	111 (55)	359 (42)	1,132 (67)	1,864 (49)	<0.001
Government/institution’s web page	711 (75)	470 (83)	69 (86)	662 (90)	194 (75)	547 (72)	173 (74)	432 (86)	138 (63)	707 (84)	1,285 (75)	2,818 (81)	0.001
WHO web page	246 (30)	340 (61)	66 (81)	637 (85)	122 (50)	487 (65)	149 (60)	382 (74)	123 (49)	555 (64)	706 (50)	2,401 (67)	<0.001

Osterrieder A, et al. BMJ Open 2021; 11:e046863. doi: 10.1136/bmjopen-2020-046863

Suppl. Table 35 Most prevalent topic areas with unclear or conflicting COVID-19 information, and most prevalent 'fake news', breakdown by country

Values in cells are n (weighted %) of respondents who replied 'yes'.

**Variable and categories**	**Thailand**	**Malaysia**	**UK**	**Italy**	**Slovenia**	**Total**	**P-value**
	N=1,476	N=827	N=1,009	N=712	N=1,034	N=5,058	
**Have you seen any unclear or conflicting information about COVID-19 in the last month?**							
Ways to avoid the infection	564 (36)	409 (47)	679 (68)	410 (64)	682 (64)	2,744 (54)	<0.001
Symptoms of COVID-19	568 (36)	353 (42)	590 (62)	328 (44)	494 (44)	2,333 (45)	<0.001
What to do in case of symptoms	506 (34)	295 (37)	438 (43)	293 (45)	435 (42)	1,967 (40)	0.058
Social distancing guidance	490 (33)	292 (42)	568 (56)	314 (42)	559 (51)	2,223 (44)	<0.001
Quarantine/isolation	529 (36)	314 (39)	547 (54)	292 (41)	559 (52)	2,241 (44)	<0.001
Penalties if disobey restrictions	614 (41)	384 (42)	620 (60)	378 (52)	508 (45)	2,504 (47)	<0.001
Risks in case of infection	527 (34)	327 (37)	542 (54)	330 (49)	493 (46)	2,219 (43)	<0.001
Numbers of coronavirus cases/deaths related to COVID-19	563 (37)	284 (47)	741 (72)	457 (66)	463 (46)	2,508 (52)	<0.001
Government support schemes (e.g. financial)	779 (51)	432 (53)	438 (46)	492 (69)	572 (51)	2,713 (53)	<0.001
Testing	531 (34)	376 (39)	734 (72)	520 (72)	534 (49)	2,695 (51)	<0.001
Travel restrictions (e.g. curfew, restricted hours of movement)	520 (33)	407 (43)	641 (62)	382 (55)	533 (45)	2,483 (46)	<0.001
**Have you come across news about the following COVID-19 topics that seemed fake to you?**							
General spread of fear	668 (42)	606 (70)	693 (72)	382 (58)	771 (69)	3,120 (60)	<0.001
Coronavirus as an engineered modified virus	543 (32)	613 (65)	819 (81)	613 (82)	864 (75)	3,452 (63)	<0.001
Minimisation of risks	440 (27)	416 (39)	579 (55)	540 (69)	731 (62)	2,706 (48)	<0.001
Numbers of infected/deceased people	512 (33)	400 (47)	615 (61)	475 (75)	574 (54)	2,576 (51)	<0.001
Unreasonable health recommendations	517 (32)	545 (55)	574 (57)	385 (50)	650 (60)	2,671 (49)	<0.001
Pharmaceutical conspiracy	490 (32)	440 (50)	525 (54)	489 (63)	673 (61)	2,617 (49)	<0.001
Home-made recipes to make sanitizer products	538 (32)	573 (61)	557 (56)	516 (70)	603 (51)	2,787 (51)	<0.001
Alternative drugs/cure	537 (33)	581 (60)	697 (67)	444 (58)	612 (51)	2,871 (51)	<0.001
Fear toward products coming from infected countries	458 (29)	549 (63)	483 (49)	425 (56)	519 (48)	2,434 (46)	<0.001

Suppl. Table 36 Most prevalent topic areas with unclear or conflicting COVID-19 information, and most prevalent 'fake news', breakdown by country and education level

P/S = primary or lower/secondary education; T = tertiary education. Values in cells are n (weighted %) of respondents who replied 'yes'.

**Variable and categories**	**Thailand**		**Malaysia**		**UK**		**Italy**		**Slovenia**		**Total**		
**Education level**	**P/S**	**T**	**P/S**	**T**	**P/S**	**T**	**P/S**	**T**	**P/S**	**T**	**P/S**	**T**	**P-value (for total)**
	N=909	N=567	N=82	N=745	N=247	N=762	N=217	N=495	N=202	N=832	N=1,657	N=3,401	
**Have you seen any unclear or conflicting information about COVID-19 in the last month?**													
Ways to avoid the infection	276 (33)	288 (51)	37 (46)	372 (49)	153 (66)	526 (69)	119 (65)	291 (60)	125 (63)	557 (67)	710 (50)	2,034 (62)	<0.001
Symptoms	268 (33)	300 (53)	36 (43)	317 (41)	146 (65)	444 (59)	94 (42)	234 (48)	96 (44)	398 (46)	640 (42)	1,693 (51)	<0.001
What to do in case of symptoms	245 (31)	261 (47)	32 (38)	263 (36)	96 (42)	342 (44)	94 (46)	199 (43)	80 (42)	355 (41)	547 (38)	1,420 (43)	0.026
Social distancing guidance	249 (31)	241 (42)	36 (44)	256 (34)	113 (51)	455 (61)	92 (41)	222 (46)	109 (50)	450 (53)	599 (41)	1,624 (51)	<0.001
Quarantine/isolation	278 (34)	251 (45)	32 (40)	282 (38)	123 (51)	424 (56)	84 (41)	208 (43)	102 (50)	457 (55)	619 (41)	1,622 (50)	<0.001
Penalties if disobey restrictions	315 (38)	299 (52)	34 (40)	350 (48)	143 (56)	477 (62)	103 (50)	275 (56)	101 (44)	407 (47)	696 (44)	1,808 (55)	<0.001
Risks in case of infection	257 (31)	270 (49)	32 (36)	295 (39)	127 (54)	415 (55)	105 (50)	225 (46)	93 (45)	400 (47)	614 (40)	1,605 (49)	<0.001
Numbers of coronavirus cases/deaths related to COVID-19	284 (33)	279 (52)	42 (50)	242 (33)	172 (70)	569 (74)	140 (67)	317 (65)	107 (50)	356 (41)	745 (49)	1,763 (56)	0.001
Government support schemes (e.g. financial)	402 (47)	377 (69)	44 (54)	388 (52)	103 (50)	335 (43)	138 (69)	354 (71)	108 (50)	464 (54)	795 (52)	1,918 (55)	0.257
Testing	258 (31)	273 (49)	31 (38)	345 (45)	161 (68)	573 (75)	145 (70)	375 (76)	95 (48)	439 (51)	690 (46)	2,005 (62)	<0.001
Travel restrictions (e.g. curfew, restricted hours of movement)	248 (30)	272 (49)	36 (42)	371 (49)	142 (59)	499 (65)	112 (55)	270 (55)	96 (41)	437 (51)	634 (42)	1,849 (56)	<0.001
**Have you come across news about the following COVID-19 topics that seemed fake to you?**													
General spread of fear	308 (37)	360 (64)	56 (69)	550 (73)	182 (76)	511 (68)	116 (60)	266 (54)	147 (66)	624 (74)	809 (57)	2,311 (67)	<0.001
Coronavirus as an engineered modified virus	209 (26)	334 (61)	52 (62)	561 (76)	193 (80)	626 (82)	174 (80)	439 (89)	156 (70)	708 (84)	784 (56)	2,668 (79)	<0.001
Minimisation of risks	178 (23)	262 (47)	31 (36)	385 (51)	128 (52)	451 (59)	141 (63)	399 (81)	122 (56)	609 (71)	600 (41)	2,106 (62)	<0.001
Numbers of infected/deceased people	231 (29)	281 (51)	40 (47)	360 (49)	152 (62)	463 (61)	153 (719	322 (67)	118 (55)	456 (54)	694 (49)	1,882 (57)	<0.001
Unreasonable health recommendations	204 (27)	313 (57)	45 (52)	500 (66)	131 (55)	443 (59)	101 (46)	284 (60)	122 (58)	528 (64)	603 (44)	2,068 (61)	<0.001
Pharmaceutical conspiracy	239 (29)	251 (45)	41 (49)	399 (54)	131 (56)	394 (52)	138 (60)	351 (71)	125 (58)	548 (64)	674 (46)	1,943 (57)	<0.001
Home-made recipes to make sanitizer products	230 (27)	308 (55)	51 (59)	522 (69)	158 (62)	399 (51)	149 (68)	367 (75)	104 (46)	499 (59)	692 (47)	2,095 (59)	<0.001
Alternative drugs/cure	240 (28)	297 (53)	48 (57)	533 (71)	168 (65)	529 (69)	125 (55)	319 (66)	105 (44)	507 (61)	686 (46)	2,185 (64)	<0.001
Fear toward products coming from infected countries	197 (25)	261 (46)	52 (62)	497 (67)	127 (52)	356 (46)	126 (55)	299 (59)	100 (46)	419 (51)	602 (44)	1,832 (51)	<0.001

Suppl. Table 37 Most prevalent topic areas with unclear or conflicting COVID-19 information, and most prevalent 'fake news', breakdown by country and self-reported level of understanding of COVID-19

H = high/very high/expert level; S = some; N = a little/none at all. Values in cells are n (weighted %) of respondents who replied 'yes'.

**Variable and categories**	**Thailand**			**Malaysia**			**UK**			**Italy**			**Slovenia**			**Total**			
**Self-reported level of understanding of COVID-19**	**H**	**S**	**N**	**H**	**S**	**N**	**H**	**S**	**N**	**H**	**S**	**N**	**H**	**S**	**N**	**H**	**S**	**N**	**P-value (for total)**
	N=965	N=459	N=52	N=435	N=359	N=33	N=647	N=336	N=26	N=368	N=324	N=20	N=713	N=279	N=42	N=3,128	N=1,757	N=173	
**Have you seen any unclear or conflicting information about COVID-19 in the last month?**																			
Ways to avoid the infection	401 (40)	145 (32)	18 (19)	197 (43)	191 (46)	21 (63)	416 (63)	248 (76)	15 (53)	202 (54)	193 (72)	15 (73)	445 (61)	211 (73)	26 (53)	1,661 (51)	988 (58)	95 (51)	0.094
Symptoms of COVID-19	400 (40)	150 (33)	18 (19)	170 (36)	167 (49)	16 (51)	363 (58)	210 (66)	17 (79)	147 (31)	163 (53)	18 (81)	312 (40)	164 (54)	18 (41)	1,392 (42)	854 (50)	87 (49)	0.026
What to do in case of symptoms	361 (37)	129 (30)	16 (17)	134 (34)	145 (41)	16 (39)	272 (39)	156 (49)	10 (59)	138 (34)	144 (55)	11 (49)	285 (37)	130 (52)	20 (40)	1,190 (37)	704 (44)	73 (37)	0.041
Social distancing guidance	349 (37)	124 (27)	17 (19)	132 (36)	144 (43)	16 (62)	355 (52)	199 (62)	14 (70)	163 (38)	140 (45)	11 (65)	362 (47)	170 (58)	27 (64)	1,361 (42)	777 (46)	85 (54)	0.168
Quarantine/isolation	379 (39)	139 (32)	11 (11)	153 (33)	145 (39)	16 (71)	338 (49)	193 (59)	16 (76)	148 (39)	135 (44)	9 (39)	372 (50)	165 (58)	22 (41)	1,390 (43)	777 (46)	74 (50)	0.397
Penalties if disobey restrictions	477 (49)	126 (28)	11 (11)	186 (35)	180 (46)	18 (56)	381 (54)	225 (68)	14 (66)	187 (47)	180 (56)	11 (69)	324 (44)	162 (48)	22 (53)	1,555 (47)	873 (48)	76 (47)	0.906
Risks in case of infection	381 (38)	132 (29)	14 (15)	152 (29)	158 (43)	17 (50)	337 (50)	191 (62)	14 (46)	158 (43)	156 (53)	16 (73)	312 (46)	159 (45)	22 (45)	1,340 (41)	796 (46)	83 (42)	0.343
Numbers of coronavirus cases/deaths related to COVID-19	416 (42)	134 (29)	13 (15)	129 (41)	137 (50)	18 (68)	463 (66)	261 (81)	17 (77)	233 (67)	214 (66)	10 (57)	284 (43)	156 (53)	23 (57)	1,525 (50)	902 (54)	81 (54)	0.276
Government support schemes (e.g. financial)	583 (60)	178 (38)	18 (20)	208 (46)	203 (61)	21 (62)	269 (40)	158 (53)	11 (56)	248 (67)	227 (71)	17 (78)	372 (48)	176 (59)	24 (48)	1,680 (52)	942 (55)	91 (50)	0.590
Testing	392 (39)	124 (29)	15 (15)	181 (36)	179 (46)	16 (32)	467 (70)	249 (74)	18 (77)	266 (71)	239 (71)	15 (86)	357 (48)	154 (55)	23 (31)	1,663 (50)	945 (53)	87 (39)	0.108
Travel restrictions (e.g. curfew, restricted hours of movement)	391 (39)	118 (25)	11 (11)	209 (37)	178 (46)	20 (62)	398 (60)	228 (71)	15 (52)	192 (50)	176 (58)	14 (78)	341 (43)	167 (50)	25 (41)	1,531 (44)	867 (49)	85 (47)	0.356

**Have you come across news about the following COVID-19 topics that seemed fake to you?**																			
General spread of fear	488 (47)	158 (36)	22 (23)	320 (65)	266 (80)	20 (56)	449 (70)	228 (73)	16 (81)	208 (57)	163 (59)	11 (61)	518 (71)	222 (65)	31 (66)	1,983 (61)	1,037 (60)	100 (54)	0.594
Coronavirus as an engineered modified virus	390 (37)	134 (26)	19 (19)	327 (71)	266 (62)	20 (46)	532 (83)	268 (79)	19 (70)	320 (87)	277 (80)	16 (60)	598 (80)	231 (65)	35 (75)	2,167 (66)	1,176 (60)	109 (49)	0.007
Minimisation of risks	305 (30)	120 (24)	15 (13)	222 (38)	176 (41)	18 (32)	377 (56)	191 (56)	11 (39)	277 (64)	249 (74)	14 (54)	510 (64)	196 (57)	25 (47)	1,691 (48)	932 (49)	83 (33)	0.063
Numbers of infected/deceased people	345 (34)	148 (33)	19 (18)	206 (49)	174 (48)	20 (39)	392 (58)	207 (66)	16 (75)	252 (76)	214 (75)	9 (63)	377 (51)	172 (62)	25 (61)	1,572 (49)	915 (55)	89 (45)	0.105
Unreasonable health recommendations	387 (36)	113 (26)	17 (17)	286 (54)	237 (53)	22 (63)	375 (55)	186 (58)	13 (71)	211 (57)	163 (44)	11 (54)	440 (59)	186 (65)	24 (48)	1,699 (50)	885 (47)	87 (50)	0.538
Pharmaceutical conspiracy	358 (36)	112 (25)	20 (21)	238 (53)	188 (48)	14 (38)	355 (55)	158 (51)	12 (56)	266 (69)	209 (57)	14 (65)	453 (61)	192 (61)	28 (45)	1,670 (52)	859 (46)	88 (40)	0.059
Home-made recipes to make sanitizer products	400 (38)	122 (24)	16 (15)	309 (62)	241 (62)	23 (57)	366 (56)	179 (55)	12 (68)	274 (78)	227 (62)	15 (71)	411 (52)	170 (51)	22 (45)	1,760 (52)	939 (49)	88 (48)	0.390
Alternative drugs/cure	409 (39)	112 (24)	16 (16)	305 (57)	257 (75)	19 (20)	468 (72)	214 (62)	15 (50)	243 (64)	188 (52)	13 (66)	430 (53)	159 (45)	23 (58)	1,855 (54)	930 (49)	86 (33)	0.004
Fear toward products coming from infected countries	330 (33)	109 (23)	19 (20)	297 (65)	234 (68)	18 (39)	317 (50)	155 (48)	11 (44)	226 (58)	187 (55)	12 (64)	352 (47)	145 (49)	22 (46)	1,522 (47)	830 (46)	82 (39)	0.456
