# Supplementary material for: Economic and social impacts of COVID-19 and public health measures: results from an anonymous online survey in Thailand, Malaysia, the UK, Italy and Slovenia
Source: BMJ Open. 2021 Jul 20;11(7):e046863. doi: 10.1136/bmjopen-2020-046863 (PMC8295020; doi:10.1136/bmjopen-2020-046863)

Supplementary figure for "Economic and social impacts of COVID-19 and public health measures: results from an anonymous online survey in Thailand, Malaysia, the United Kingdom, Italy and Slovenia"

Anne Osterrieder[1,2], Giulia Cuman[3], Wirichada Pan-ngum[1,4], Phaik Kin Cheah[5], Phee-Kheng Cheah[6], Pimnara Peerawaranun[1], Margherita Silan[7], Miha Orazem[8,9], Ksenija Perkovic[10], Urh Groselj[8,11], Mira Leonie Schneiders[1,2,12], Tassawan Poomchaichote[1,13], Naomi Waithira[1,2], Supa-at Asarath[1], Bhensri Naemiratch[1], Supanat Ruangkajorn[1], Lenart Skof[14], Natinee Kulpijit[1], Constance R.S. Mackworth-Young[15], Darlene Ongkili[16], Rita Chanviriyavuth[1], Mavuto Mukaka[1,2], Phaik Yeong Cheah[1,2,12,,13]

[1]Mahidol Oxford Tropical Medicine Research Unit, Faculty of Tropical Medicine, Mahidol University, Bangkok, 10400, Thailand
[2]Centre for Tropical Medicine & Global Health, Nuffield Department of Medicine, University of Oxford, Oxford, UK
[3]Paediatric Ethics Committee; Research Ethics Committee, University Hospital of Padua, Padua, Italy
[4]Department of Tropical Hygiene, Faculty of Tropical Medicine, Mahidol University, Bangkok, 10400, Thailand
[5]Faculty of Arts and Social Science, Universiti Tunku Abdul Rahman, Kampar, Malaysia
[6]Emergency and Trauma Department, Sabah Women and Children's Hospital, Ministry of Health Malaysia, Kota Kinabalu, Malaysia
[7]Department of Statistical Sciences, University of Padua, Padua, Italy
[8]Faculty of Medicine, University of Ljubljana, Ljubljana, Slovenia
[9]Department of Radiation Oncology, Institute of Oncology Ljubljana, Ljubljana, Slovenia
[10]Institute for Social Studies, Science and Research Centre Koper, Koper, Slovenia
[11]Department of Endocrinology, Diabetes and Metabolic Diseases, University Children's Hospital, University Medical Center, University Children's Hospital Ljubljana, Ljubljana, Slovenia
[12]Ethox Centre, Nuffield Department of Population Health, University of Oxford, Oxford, UK
[13]The SoNAR-Global Network
[14]Institute for Philosophical Studies, Science and Research Centre Koper, Koper, Slovenia
[15]Department of Global Health and Development, London School of Hygiene and Tropical Medicine, London, UK
[16]Emergency and Trauma Department, Queen Elizabeth Hospital, Ministry of Health Malaysia, Kota Kinabalu, Malaysia

Corresponding author: Phaik Yeong Cheah, 420/6 Mahidol-Oxford Tropical Medicine 24 Research Unit, Faculty of Tropical Medicine, Rajvithi Road, Bangkok, Thailand, 10400. Email: phaikyeong@tropmedres.ac

Supplementary Figure 1: Diagram showing the level of coordination of public information campaigns on COVID-19 in the five study countries.

Levels of coordination: 0 = no COVID-19 public information campaign; 1 = public officials urging caution about COVID-19; 2 = coordinated public information campaign (e.g. across traditional and social media). All countries ran public information campaigns at level 2 during the study period from 1st May to 30th June 2020. Data was provided by the Oxford COVID-19 Government Response Tracker[32] and downloaded from 'Our World in Data'[33].

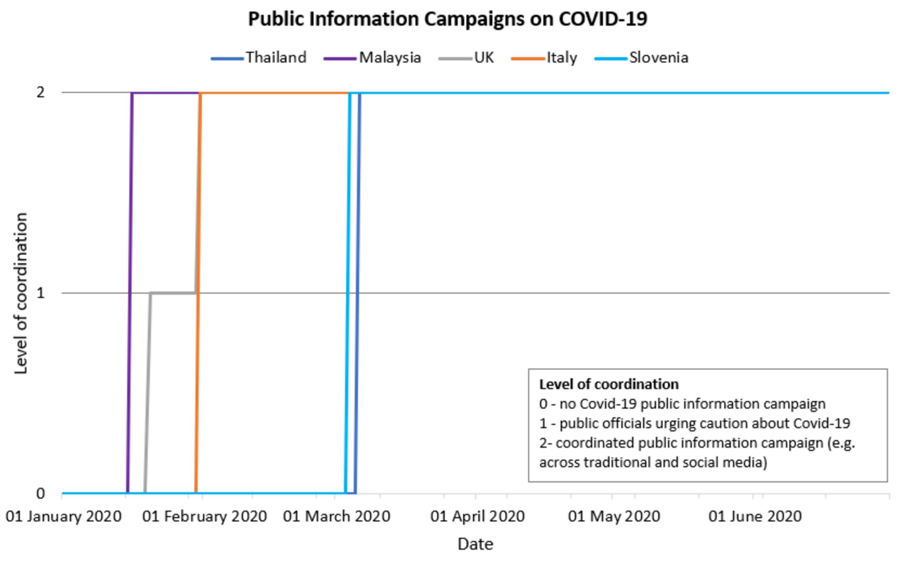

Supplement: Supplementary data [file bmjopen-2020-046863supp002.pdf]
