# Supplementary material for: Economic and social impacts of COVID-19 and public health measures: results from an anonymous online survey in Thailand, Malaysia, the UK, Italy and Slovenia
Source: BMJ Open. 2021 Jul 20;11(7):e046863. doi: 10.1136/bmjopen-2020-046863 (PMC8295020; doi:10.1136/bmjopen-2020-046863)
Supplement: Author's manuscript [file bmjopen-2020-046863.draft_revisions.pdf]

BMJ Open

BMJ Open is committed to open peer review. As part of this commitment we make the peer review history of every article we publish publicly available.

When an article is published we post the peer reviewers' comments and the authors' responses online. We also post the versions of the paper that were used during peer review. These are the versions that the peer review comments apply to.

The versions of the paper that follow are the versions that were submitted during the peer review process. They are not the versions of record or the final published versions. They should not be cited or distributed as the published version of this manuscript.

BMJ Open is an open access journal and the full, final, typeset and author-corrected version of record of the manuscript is available on our site with no access controls, subscription charges or pay-per-view fees (http://bmjopen.bmj.com).

If you have any questions on BMJ Open's open peer review process please email info.bmjopen@bmj.com

BMJ Open

Economic and social impacts of COVID-19 and public health measures: results from an anonymous online survey in Thailand, Malaysia, the United Kingdom, Italy and Slovenia

Journal:	*BMJ Open*
Manuscript ID	bmjopen-2020-046863
Article Type:	Original research
Date Submitted by the Author:	15-Nov-2020
Complete List of Authors:	Osterrieder, Anne; Mahidol University Rajvithi Campus, Mahidol Oxford Tropical Medicine Research Unit, Faculty of Tropical Medicine; University of Oxford, Centre for Tropical Medicine and Global Health, Nuffield Department of Medicine<br>Cuman, Giulia; University Hospital of Padova, Paediatric Ethics Committee; Research Ethics Committee<br>Pan-Ngum, Wirichada; Mahidol University Rajvithi Campus, Department of Tropical Hygiene Faculty of Tropical Medicine; Mahidol University Rajvithi Campus, Mahidol Oxford Tropical Medicine Research Unit, Faculty of Tropical Medicine<br>Cheah, Phaik Kin; Universiti Tunku Abdul Rahman, Faculty of Arts and Social Science<br>Cheah, Phee-Kheng; Ministry of Health, Malaysia, Emergency & Trauma Department, Sabah Women & Children's Hospital<br>Peerawaranun, Pimnara; Mahidol University Rajvithi Campus, Mahidol Oxford Tropical Medicine Research Unit, Faculty of Tropical Medicine<br>Silan, Margherita; University of Padua, Department of Statistical Sciences<br>Orazem, Miha; University of Ljubljana, Faculty of Medicine; Institute of Oncology Ljubljana, Department of Radiation Oncology<br>Perkovic, Ksenija; Science and Research Centre Koper, Institute for Social Studies<br>Groselj, Urh; University of Ljubljana, Faculty of Medicine; University Children’s Hospital, University 26 Medical Center – University Children’s Hospital Ljubljana,, Department of Endocrinology, Diabetes and Metabolic Diseases<br>Schneiders, Mira Leonie; Mahidol University Rajvithi Campus, Mahidol Oxford Tropical Medicine Research Unit, Faculty of Tropical Medicine; Mahidol University Rajvithi Campus, Mahidol Oxford Tropical Medicine Research Unit, Faculty of Tropical Medicine<br>Poomchaichote, Tassawan; Mahidol University Rajvithi Campus, Mahidol Oxford Tropical Medicine Research Unit, Faculty of Tropical Medicine<br>Waithira, Naomi; Mahidol University Rajvithi Campus, Mahidol Oxford Tropical Medicine Research Unit, Faculty of Tropical Medicine; University of Oxford, Centre for Tropical Medicine and Global Health, Nuffield Department of Medicine<br>Asarath, Supa-at; Mahidol University Rajvithi Campus, Mahidol Oxford Tropical Medicine Research Unit, Faculty of Tropical Medicine<br>Naemiratch, Bhensri; Mahidol University Rajvithi Campus, Mahidol

	Oxford Tropical Medicine Research Unit, Faculty of Tropical Medicine<br>Ruangkajorn, Supanat; Mahidol University Rajvithi Campus, Mahidol Oxford Tropical Medicine Research Unit, Faculty of Tropical Medicine<br>Skof, Lenart; Science and Research Centre Koper, Institute for Philosophical Studies<br>Kulpijit, Natinee; Mahidol University Rajvithi Campus, Mahidol Oxford Tropical Medicine Research Unit, Faculty of Tropical Medicine<br>Mackworth-Young, Constance; London School of Hygiene & Tropical Medicine, Department of Global Health & Development<br>Ongkili, Darlene; Ministry of Health, Malaysia, Emergency & Trauma Department, Queen Elizabeth Hospital<br>Chanviriyavuth, Rita; Mahidol University Rajvithi Campus, Mahidol Oxford Tropical Medicine Research Unit, Faculty of Tropical Medicine<br>Mukaka, Mavuto; Mahidol University Rajvithi Campus, Mahidol Oxford Tropical Medicine Research Unit, Faculty of Tropical Medicine; University of Oxford, Centre for Tropical Medicine and Global Health, Nuffield Department of Medicine<br>Cheah, Phaikyeong; Mahidol University Rajvithi Campus, Mahidol Oxford Tropical Medicine Research Unit, Faculty of Tropical Medicine; University of Oxford, Centre for Tropical Medicine and Global Health, Nuffield Department of Medicine

Keywords:	Public health < INFECTIOUS DISEASES, Epidemiology < TROPICAL MEDICINE, PUBLIC HEALTH

SCHOLARONE™ Manuscripts

I, the Submitting Author has the right to grant and does grant on behalf of all authors of the Work (as defined in the below author licence), an exclusive licence and/or a non-exclusive licence for contributions from authors who are: i) UK Crown employees; ii) where BMJ has agreed a CC-BY licence shall apply, and/or iii) in accordance with the terms applicable for US Federal Government officers or employees acting as part of their official duties; on a worldwide, perpetual, irrevocable, royalty-free basis to BMJ Publishing Group Ltd ("BMJ") its licensees and where the relevant Journal is co-owned by BMJ to the co-owners of the Journal, to publish the Work in this journal and any other BMJ products and to exploit all rights, as set out in our licence.

The Submitting Author accepts and understands that any supply made under these terms is made by BMJ to the Submitting Author unless you are acting as an employee on behalf of your employer or a postgraduate student of an affiliated institution which is paying any applicable article publishing charge ("APC") for Open Access articles. Where the Submitting Author wishes to make the Work available on an Open Access basis (and intends to pay the relevant APC), the terms of reuse of such Open Access shall be governed by a Creative Commons licence – details of these licences and which Creative Commons licence will apply to this Work are set out in our licence referred to above.

Other than as permitted in any relevant BMJ Author's Self Archiving Policies, I confirm this Work has not been accepted for publication elsewhere, is not being considered for publication elsewhere and does not duplicate material already published. I confirm all authors consent to publication of this Work and authorise the granting of this licence.

For peer review only

Economic and social impacts of COVID-19 and public health measures: results from an anonymous online survey in Thailand, Malaysia, the United Kingdom, Italy and Slovenia

Anne Osterrieder[1,2], Giulia Cuman[3], Wirichada Pan-ngum[1,4], Phaik Kin Cheah[5], Phee-Kheng Cheah[6], Pimnara Peerawaranun[1], Margherita Silan[7], Miha Orazem[8,9], Ksenija Perkovic[10], Urh Groselj[8,11], Mira Leonie Schneiders[1,2,12], Tassawan Poomchaichote[1,13], Naomi Waithira[1,2], Supa-at Asarath[1], Bhensri Naemiratch[1], Supanat Ruangkajorn[1], Lenart Skof[14], Natinee Kulpijit[1], Constance R.S. Mackworth-Young[15], Darlene Ongkili[16], Rita Chanviriyavuth[1], Mavuto Mukaka[1,2], Phaik Yeong Cheah[1,2,12,13]

[1]Mahidol Oxford Tropical Medicine Research Unit, Faculty of Tropical Medicine, Mahidol University, Bangkok, 10400, Thailand

[2]Centre for Tropical Medicine & Global Health, Nuffield Department of Medicine, University of Oxford, Oxford, UK

[3]Paediatric Ethics Committee; Research Ethics Committee, University Hospital of Padua, Padua, Italy

[4]Department of Tropical Hygiene, Faculty of Tropical Medicine, Mahidol University, Bangkok, 10400, Thailand

[5]Faculty of Arts and Social Science, Universiti Tunku Abdul Rahman, Kampar, Malaysia

[6]Emergency and Trauma Department, Sabah Women and Children's Hospital, Ministry of Health Malaysia, Kota Kinabalu, Malaysia

[7]Department of Statistical Sciences, University of Padua, Padua, Italy

[8]Faculty of Medicine, University of Ljubljana, Ljubljana, Slovenia

[9]Department of Radiation Oncology, Institute of Oncology Ljubljana, Ljubljana, Slovenia

[10]Institute for Social Studies, Science and Research Centre Koper, Koper, Slovenia

[11]Department of Endocrinology, Diabetes and Metabolic Diseases, University Children's Hospital, University Medical Center – University Children's Hospital Ljubljana, Ljubljana, Slovenia

[12]Ethox Centre, Nuffield Department of Population Health, University of Oxford, Oxford, UK

[13]The SoNAR-Global Network

[14]Institute for Philosophical Studies, Science and Research Centre Koper, Koper, Slovenia

[15]Department of Global Health and Development, London School of Hygiene and Tropical Medicine, London, UK

[16]Emergency and Trauma Department, Queen Elizabeth Hospital, Ministry of Health Malaysia, Kota Kinabalu, Malaysia

Corresponding author: Phaik Yeong Cheah, 420/6 Mahidol-Oxford Tropical Medicine 24 Research Unit, Faculty of Tropical Medicine, Rajvithi Road, Bangkok, Thailand, 10400. Email: phaikyeong@tropmedres.ac

Word count: abstract 299 words, main text 5335 words

For peer review only - http://bmjopen.bmj.com/site/about/guidelines.xhtml

Keywords: COVID-19, SARS-CoV-2, non-pharmaceutical interventions, restrictions, social distancing, quarantine, isolation, compliance, information, fake news, social, ethical, behavioural, survey

Abstract

Objectives

In the absence of a vaccine and widely available treatments for COVID-19, governments have relied primarily on non-pharmaceutical interventions to curb the pandemic. To aid understanding of the impact of these public health measures on different social groups we conducted a mixed-methods study in five countries ('SEBCOV - Social, ethical and behavioural aspects of COVID-19'). Here we report the results of the SEBCOV online survey.

Design

Overall, 5,058 respondents from Thailand, Malaysia, the United Kingdom, Italy and Slovenia completed the self-administered survey between May and June 2020. Post-stratification weighting was applied, and associations between categorical variables assessed.

Results

Among the five countries, Thai respondents appeared to have been most, and Slovenian respondents least, affected economically. Overall, lower education levels, larger households, having children under 18 in the household, being 65 years or older and having flexible/no income were associated with worse economic impact. Regarding social impact, respondents expressed most concern about their social life, physical health, and mental health and wellbeing.

There were large differences between countries in terms of voluntary behavioural change, and in compliance and agreement with COVID-19 restrictions. Overall, self-reported compliance was higher among respondents reporting a high understanding of COVID-19. UK respondents felt able to cope the longest and Thai respondents the shortest with only going out for essential needs or work, with 60% and 26% respectively able to cope with 29 days or longer. Many respondents reported seeing news that seemed fake to them, the proportion varying between countries, and with education level and self-reported levels of understanding of COVID-19.

Conclusions

Our data showed that COVID-19 public health measures have uneven economic and social impacts on people from different countries and social groups. Understanding the factors associated with these impacts can help to inform future public health interventions and mitigate their negative consequences.

Registration: TCTR20200401002

For peer review only - http://bmjopen.bmj.com/site/about/guidelines.xhtml

Summary

Strengths

- Our research findings help to address an evidence gap as identified by the global research community in a recent study on COVID-19 research priorities, which identified public health messaging, compliance and trust in public health interventions, and evaluation of these interventions in varied settings as areas of high priority (BMJ Global Health Vol 5, Issue 7 (https://gh.bmj.com/content/5/7/e003306).
- Because we recruited a reasonably large sample size in each country (between 700-1400), we were able to compare population segments (e.g. men versus women, younger versus older people, those with lower versus higher levels of education) in the whole cohort, and between countries.
- Our online survey enabled us to capture people's experiences and concerns in multiple domains, in five countries, all of which had restrictions in place, during the relatively early stage of the COVID-19 pandemic.
- Our study and survey questions were discussed with the Bangkok Health Research Ethics Interest Group, a public involvement group set in a dedicated virtual meeting.

Limitations

- We did not aim to obtain nationally representative samples and acknowledge that although we used weighting strategies in our analysis, our results may not be fully representative of the populations in the respective countries.

Introduction

COVID-19 is a respiratory disease caused by the novel coronavirus 'severe acute respiratory syndrome coronavirus 2' (SARS-CoV2), which is transmitted through droplets, close contact, and aerosols[1,2]. The SARS-CoV2 outbreak was first reported in December 2019 in Wuhan, China[3], with the World Health Organization declaring it Public Health Emergency of International Concern on 30th January 2020 and a global pandemic on 11th March 2020[1].

In the absence of a vaccine or widely available and effective pharmaceutical treatments, the impact of COVID-19 is being mitigated using non-pharmaceutical interventions (NPIs)[4,5]. Examples of NPIs include: social distancing (or 'physical distancing') measures, such as isolation of sick individuals, quarantine of exposed individuals, contact tracing, voluntary shielding, travel-related restrictions; and personal protective measures, such as hand hygiene and wearing face masks[4,6,7]. Scientific evidence indicates that NPIs are effective measures to contain a pandemic and ease pressures on health care systems[6-12]. However, authorities and policy makers need to consider the societal, economic and

For peer review only

ethical impacts of these public health measures, in particular on vulnerable groups[13,14]. Such groups might be disproportionally affected by NPIs and/or might be unable to comply with them[15], e.g. due to loss of income when having to isolate at home, crowded living conditions[14], or not being able to afford masks[16].

As the COVID-19 pandemic continues, evidence is urgently needed to understand how people perceive and experience NPIs, which groups are disproportionally negatively affected by NPIs, and how communication is perceived by various social groups[17]. This understanding is important so that the policies can be improved to minimize the negative impact of COVID-19 on people's lives, and to improve communications.

Here we report the highlights of an online survey conducted in Southeast Asia (Thailand and Malaysia, both upper middle-income countries), and Europe (the United Kingdom, Italy and Slovenia, all high-income countries) between May 1 to June 30, 2020 as part of the mixed-methods study 'Social, ethical and behavioural aspects of COVID-19' (SEBCOV)[18]. These findings help to address an evidence gap as identified by the global research community in a recent study on COVID-19 research priorities[19], which identified public health messaging, compliance and trust in public health interventions, and evaluation of these interventions in varied settings as areas of high priority[19].

Methods

Survey development

The survey contained five sections with 36 questions (single-answer multiple choice and five-point Likert scales) on (1) socio-demographic information; (2) income, occupation status and economic impacts of COVID-19 restrictions; (3) sources of, preferences and perceptions regarding COVID-19 related communication, and the occurrence of 'fake news' (untrue information presented as news); and (4) perceived levels of understanding of COVID-19 and NPIs, agreement with NPIs, voluntary behavioural changes, and concerns and coping strategies relating to restrictions[20]. The Malaysia and UK surveys were administered in English, with the other surveys translated into the respective country languages. The self-administered online survey was set up using the 'JISC Online surveys' platform[21].

Patient and public involvement

The survey questions were pilot-tested with 25 people from participating countries, and revised accordingly based on feedback. In addition, the Bangkok Health Research Ethics Interest Group, a public involvement group set up by the Mahidol Oxford Tropical Medicine Research Unit (MORU)[22], discussed the study and the survey questions in a dedicated virtual meeting. Selected questions were

For peer review only - http://bmjopen.bmj.com/site/about/guidelines.xhtml

tested using an adapted cognitive testing technique using the "thinking out loud" approach[23], and the collaborative virtual sticky notes board 'Padlet'[24].

Participant selection and recruitment

Adults of any age residing in Thailand, Italy, Malaysia, United Kingdom (UK) or Slovenia at the time of the study were eligible to take part. Participants needed to be able to use a computer or smart phone to access the survey and provide online consent to participate.

The survey was open from 1st May to 30th June 2020 (1st-30th June for Slovenia due to late start). Participants were recruited using a combination of approaches: snowball sampling through personal and professional networks (via email, social media and messenger apps, mailing lists, and organisations such as the Medical Chamber[25] in Slovenia); a polling company[26] in Thailand; and through promoted posts on Facebook. Facebook allows users to 'boost' posts to selected demographic audiences for a small fee, so that the post appears on their Facebook newsfeed[27]. To achieve more balanced responses in the categories of gender, education level and geographic distribution, promoted Facebook posts were targeted at people with primary or lower/secondary education in UK and Malaysia; potential participants in Wales, Scotland and Northern Ireland in the UK; and at men in the UK and Italy.

Sample size

Each country aimed to recruit a minimum sample of 600 respondents, exceeding the 40-200 respondents recommended for a mixed-methods study[28]. A minimum sample size of 600 respondents is adequate to estimate the prevalence of a response assuming a 50% prevalence rate, with 95% confidence and with a precision of 4%. The 50% prevalence is the standard assumption for precision sample size calculations when the true prevalence is not available, as this gives the highest sample size for a binomial distribution for a desired level of precision.

Statistical analysis

To simplify analysis, answers in the following categories were combined as follows: "slightly agree/highly agree" were combined into one "agree", category, and "slightly/strongly disagree" responses into one "disagree" category (Suppl. Tables 23-27). To understand the distribution of the basic demographic variables in the respondent sample, the observed frequencies and sample characteristics are reported using unweighted percentages (Suppl. Table 1). The characteristics for the rest of the variables are presented using the observed survey frequency counts followed by weighted percentages (Suppl. Tables 2-37). Post-stratification weighting was used to align the composition of the respondents' sample with the known distribution of the whole population's characteristics,

For peer review only - http://bmjopen.bmj.com/site/about/guidelines.xhtml

reducing sampling error. Weights were computed considering three stratifying variables that were available from population census data from each country[29], namely, gender, age and education level. Weights were obtained as the ratio between the proportion of each possible combination of the three variables in the whole country population and the correspondent proportion in the respondent sample.

Survey data was analysed using Stata 15.0 software[30]. Frequency counts and percentages were used to summarise categorical data. Associations between categorical variables were assessed using Pearson's Chi-squared test. P-values have been provided in the tables and considered statistically significant below the two-sided alpha=0.05 level. All p-values presented in the tables are for global tests of significance. Practical significance was taken into account when interpreting differences in the results.

Results

At the time of the inception of this study, governments in Thailand, Malaysia, Italy, the UK and Slovenia had initiated public health measures, using varying degrees of "lockdowns" to curb the pandemic. Figure 1 shows a visualization of the 'Stringency Index' (SI) of the public health responses of the five government over the study period, drawing upon data provided by the Oxford COVID-19 Government Response Tracker (OxCGRT)[31]. The OxCGRT tool tracks government policies and interventions from more than 180 countries on standardized indicators, and aggregates the data into a 'Stringency Index' for each country on a scale from 0-100, with 100 indicating the strictest response[31]. For example, Italy had the strictest public health measures in early May (SI = 93) and then gradually lifted and reintroduced restrictions, whereas restrictions in the UK remained at around the same level (SI = 69-76) throughout the study period. Restrictions in Slovenia were substantially eased from June onwards (SI = 33).

Characteristics of survey respondents

A total of 5,058 participants took part in the survey: 1,476 respondents from Thailand, 827 from Malaysia, 1,009 from the UK, 712 from Italy, and 1,034 from Slovenia (Suppl. Table 1, unweighted data). Overall, around 40% identified as male, around 60% as female, and around 1% as 'other/prefer not to say'. Of all respondents, 26% were aged 18-34 years old, 65% were 35-64 years old, and 10% fell into the 65+ age group. Thirty three percent had primary or lower (from here on referred to as 'primary') or secondary education, whereas 67% had tertiary education. Overall, 10% of respondents lived in large households with six or more people. Fifty nine percent of respondents received a fixed income (salary/benefits/pension), 31% had flexible income (contract and freelance), and 10% received no or 'other income'. Thirty six percent lived with children under 18 years in their household, and 29% reported that they or a household member belonged to a "vulnerable group" (persons aged 70 or older, pregnant women, or people with serious health conditions). Nineteen

For peer review only - http://bmjopen.bmj.com/site/about/guidelines.xhtml

percent of respondents were healthcare provider/workers. Supplementary Table 1 provides the breakdown by country. All results in the following subsections are presented as weighted percentages.

Economic impacts of COVID-19 and public health measures

In order to understand the economic impacts of COVID-19, respondents who had been working before the pandemic (paid or unpaid work) were asked whether COVID-19 had created any work-related inconvenience for them. Overall, 56% of respondents said that they experienced loss of earnings, 44% reduction of working hours, 36% closure of workplace and 14% job loss (Fig. 2, Suppl. Table 2). Seventy five percent reported that they continued to work during COVID-19. Of all respondents, 53% expressed financial concerns, and 32% worried about professional/career progression. Our results indicated that the most affected country was Thailand, with 85% of respondents reporting loss of earnings, 23% loss of job, and 86% expressing financial concerns (Suppl. Table 2). Slovenian respondents reported the least severe economic impacts e.g. 30% reported loss of earnings, 3% reported loss of job, and 28% had financial concerns.

To investigate the impact of public health measures on different social groups, we analyzed responses based on gender, level of education, age group, household size, whether respondents lived with children under 18 years old, and income type.

Overall, there were no significant differences between male, female and respondents who identified as 'other/prefer not to say', and who had been working before COVID-19, in terms of loss of earnings, loss of job, reduction of working hours and closure of workplace (Fig. 2, Suppl. Table 3). Overall, fewer women continued to work during COVID-19 (71% women vs 78% men; $p=0.010$). The trend was similar at country level, except for Malaysia (73% women versus 67% men; Suppl. Table 3).

Overall, 65% of respondents with primary and secondary education who had been working before COVID-19 reported a loss of earnings, compared to 38% of respondents with tertiary education ($p<0.001$; Fig. 2, Suppl. Table 4). More respondents with primary/secondary education lost their job (17% versus 8%; $p<0.001$), and had their working hours reduced (47% versus 37%; $p<0.001$). Fewer respondents with primary/secondary education continued to work (71%, versus 83%, $p<0.001$), and 59% reported financial concerns (versus 41%; $p<0.001$). This trend was mirrored at country level. Respondents with primary/secondary education were most affected in Thailand, where 90% reported loss of earnings, 24% reported loss of job, and 89% reported financial concerns (Suppl. Table 4). Only 65% of respondents with primary/secondary education in Malaysia (versus 90% with tertiary education) and 59% in Italy (versus 79%) continued to work during COVID-19.

In order to assess whether age was a factor associated with economic impact, respondents were divided into three age groups in the analysis: 18-34 year olds, 35-64 year olds, and over 65 year olds (Fig. 2, Suppl. Table 5). There were no significant differences between age groups regarding loss of

For peer review only - http://bmjopen.bmj.com/site/about/guidelines.xhtml

earnings (p=0.102) or loss of job (p=0.054). However, the 18-34 year olds appeared to be most affected through reduction of working hours (p=0.005) and closure of workplace (p=0.003). Only 71% of 18-34 year olds and 68% of 65+ year olds continued to work during COVID-19, compared to 78% of 35-64 year olds (p=0.025). Analysing by country, the 65+ year olds reported highest loss of earnings in Malaysia (57%) and Slovenia (39%). This age group was particularly affected in Italy, where 87% of 65+ year olds reported loss of earnings and 42% reported loss of job. In all countries except for Thailand, fewer 65+ year olds continued to work during COVID-19.

Overall, larger households and having children under 18 in the household appeared to be associated with worse economic impacts (Fig. 2, Suppl. Tables 6 and 7). Overall, 67% of respondents whose household included 6 people or more reported loss of earnings (compared to 54% of households with 1-5 people; p=0.013), and 23% reported loss of job (compared to 13%; p=0.009; Suppl. Table 6). Respondents with children reported a higher loss of earnings compared to respondents without children (62% versus 53%; p=0.005), and higher job loss (18% versus 12%; p=0.008; Suppl. Table 7). Analysing by country, respondents living with children appeared to be particularly affected in Thailand and Malaysia.

We also analysed responses according to three types of income: fixed income (e.g. fixed salary, benefits or pension), flexible income (e.g. contract, freelance), and other/no income (Fig. 2; Suppl. Table 8). We did not ask for amount of income. Overall, respondents with fixed income were less affected economically than those with flexible or other/no income. Of the latter only 38% reported loss of earnings, compared to 81% of respondents with flexible income and 69% of respondents with other/no income (p<0.001). Only 8% of people with fixed income had lost their job, compared to 22% with flexible income and 27% with other/no income (p<0.001). At country level, the trends were similar (Suppl. Table 8). Fewer people with flexible or other/no income continued to work in Malaysia (42% with flexible/25% with no/other income, compared to 83% with fixed income; p<0.001), UK (57%/62%, compared to 79%; p<0.001), Italy (51%/15%, compared to 81%; p<0.001) and Slovenia (57%/59%, compared to 84%; p<0.001).

Social impacts of COVID-19 and public health measures

We asked respondents if they were concerned about the following areas of life if advised no physical contact/not allowed to go out/allowed to go out only for essential needs: caring responsibilities, physical health, recreational pursuits, sports, mental health and wellbeing, living arrangements, infrastructure (e.g. access to transport, internet), social, and religious and spiritual needs/aspects (Suppl. Table 9). Overall, respondents expressed most concern about their social life (64%), their physical health (59%), and their mental health and wellbeing (58%). This trend was largely similar in individual countries, except for Thailand, where caring responsibilities attracted the most concern

For peer review only - http://bmjopen.bmj.com/site/about/guidelines.xhtml

(62%); Malaysia, where 58% were concerned about religion and spirituality; and Slovenia, where 65% of people worried about recreational aspects. In general, there were no major differences between gender, age groups, education level, household size, living with children or income type (Suppl. Tables 10-15). Overall, those who were most worried about caring responsibilities were women (52%, versus 42% men, p<0.001; Suppl. Table 10), 35-64 year olds (53%, versus 46% of 18-34 year olds and 32% of 65+ year olds, p<0.001; Suppl. Table 11), people with primary/secondary education (49%, versus 43% with tertiary education, p=0.002; Suppl. Table 12), and people with children (64%, versus 38% of those without children, p<0.001; Suppl. Table 14).

We asked respondents how many days they could cope with not going out except for essential needs/work, with answer options ranging from one to 59 days or more. In total, 44% of respondents said that they could cope for 29 days or longer (Suppl. Table 16). However, coping time varied significantly between countries (p<0.001): in the UK, 60% of people felt they would be able to cope for 29 days or longer, whereas in Thailand, only 26% of respondents said that they could cope this long. Overall, gender, age, and household size did not appear to be associated with coping time (Suppl. Tables 17-19). Factors that appeared to be associated with lower coping times were living with children under 18 years (p=0.004, Suppl. Table 20), having primary/secondary education (p<0.001, Suppl. Table 21), and receiving flexible income (p<0.001; Suppl. Table 22). Indicators varied at country level.

Compliance and acceptance of public health measures

Next, we explored which factors were associated with compliance and agreement with public health measures. Of all respondents, 67% reported that they had changed their social behaviour *before* government restrictions were implemented (Fig. 3; Suppl. Table 23). There were significant differences at country level (p<0.001): 93% of Thai respondents reported voluntary pre-restriction behaviour change, followed by the UK (68%) and Malaysia (64%). Slovenian (47%) and Italian respondents (47%) reported the lowest levels of voluntary pre-restriction behaviour change. Overall, 92% of respondents had used sanitizer products and alcohol , 82% avoided physical contact with anyone, and 79% avoided physical contact with only vulnerable groups. In Thailand and Malaysia, 96% and 95% of respondents indicated that they had been using personal protective equipment (PPE; e.g. face masks and gloves), compared to only 33% in UK, 55% in Italy, and 67% in Slovenia (p<0.001). We also asked respondents how much they agreed with quarantine/isolation/social distancing measures and the statement that these are a necessary strategy to help control COVID-19 (Suppl. Table 23). There was a significant difference between countries (p<0.001): agreement with public health measures was highest amongst respondents from Thailand (94%) and lowest amongst those from Slovenia (around 75%).

For peer review only - http://bmjopen.bmj.com/site/about/guidelines.xhtml

Overall, fewer male than female respondents changed their social behaviour before the government implemented official restrictions (65% and 70%, respectively, p=0.039; Fig. 3, Suppl. Table 24). At country level, fewer men than women reported changing their social behaviour voluntarily, except in Thailand, where reported changes among men and women were similar (94%/92%, p=0.426). Overall, there were no significant differences between men and women when asked about how much they agreed with public health measures and the statement that these are a necessary strategy to help control COVID-19 (p=0.191; Suppl. Table 24).

When it came to education level, there were no significant differences between respondents with primary/secondary and those with tertiary education regarding voluntary behaviour change before government-imposed restrictions (p=0.369), and agreement with public health measures and the statement that these are a necessary strategy to help control COVID-19 (p=0.304; Fig. 3, Suppl. Table 25). Indicators varied at country level.

Overall, 70% of 18-34 year olds and 70% of 35-64 year olds indicated that they had voluntarily changed their behaviour before government restrictions, compared to only 57% of 65+ year olds (p=0.004; Fig. 3, Suppl. Table 26). This trend was similar at country level, except in Italy where 57% of 65+ year olds were most likely to change their behaviour, compared with 44% of 18-34 and 44% of 35-64 year olds. Overall, agreement with voluntary restrictions was similar across age groups (p=0.271; Suppl. Table 26), but fewer 65+ year expressed agreement with restrictions that were government-enforced (p=0.003). Respondents over 65 years old in Slovenia reported the lowest agreement with the statement that quarantine/isolation/social distancing are a necessary strategy to help control COVID-19 (67%), compared to 96% in Thailand and 100% in Malaysia.

Lastly, self-reported levels of understanding of COVID-19 did not significantly affect voluntary change of behaviour (p=0.091), or agreement with public health measures (p=0.688; Suppl. Table 27).

Level of understanding of COVID-19

We asked respondents to indicate their perceived level of understanding of COVID-19. Overall, 59% of respondents indicated a 'high/very high' level of understanding, 36% reported 'some' understanding, and only 5% reported 'very little/none' (Fig. 4, Suppl. Table 28). There were significant differences at country level ($p<0.001$): perceived levels of understanding were highest in Slovenia (66% reported 'high/very high', and 30% 'some' understanding) and Thailand (63% 'high/very high' and 33% 'some'), and lowest in Italy, with 47% reporting 'high/very high', and 50% reporting 'some' level of understanding.

To probe for factors associated with perceived level of understanding of COVID-19, we broke down responses by gender, age, education and healthcare worker status (Fig. 4, Suppl. Table 29). Overall, there was no significant difference between men, women and people who identified as other or

For peer review only - http://bmjopen.bmj.com/site/about/guidelines.xhtml

preferred not to say (p=0.058; Fig. 4, Suppl. Table 29). Age appeared to be a factor, as only 52% of 18-34 year old respondents self-reported 'high/very high' understanding compared to 62% of 35-64 year olds and 60% of 65+ year olds (p=0.033). Overall, fewer respondents with primary and secondary education self-reported 'high/very high' understanding (56% indicated 'high/very high' compared to 66% with tertiary education, p<0.001). Lastly, healthcare worker status was associated with perceived higher understanding (p=0.001). This trend was similar at country level, except for Malaysia, where 49% of healthcare workers reported 'high/very high' understanding compared to 52% of non-healthcare workers (p=0.805) (Suppl. Table 29).

Overall, higher levels of perceived understanding of COVID-19 were associated with higher levels of perceived understanding of public health measures (p<0.001; Suppl. Table 30). For example, 88% of respondents who self-reported 'high/very high' understanding of COVID-19 and 50% who reported 'some' understanding felt that they had a 'high/very high' level of understanding of public health measures. In contrast, only 27% of respondents who reported 'very little/no' understanding of COVID-19 indicated a high understanding of public health measures.

Information about COVID-19, unclear information and fake news

When respondents were asked how they receive/received information about COVID-19 (Suppl. Table 31), most reported traditional mass media (TV, radio, newspapers; 93%), followed by online methods (websites, email; 83%) and social media and messenger apps (79%). When asked about their preferences for receiving information, the top three responses were traditional mass media (78%), government or institution's website (77%), and online (76%). There were no significant differences based on gender (Suppl. Table 32). Fewer respondents over 65 years said that they had used online channels or social media and messenger apps, and they expressed significantly lower preference for these channels too. For example, only 66% of over 65 year olds wanted to receive information online, compared to 78%/79% of the other age groups (p<0.001), and only 52% of over 65 year olds expressed preference for social media and messenger apps, compared to 64%/64% (p=0.005; Suppl. Table 33). Overall, most respondents with primary/secondary education and those with tertiary education had received information through traditional mass media, and social media/messenger apps (Suppl. Table 34). Fewer respondents with primary/secondary education had used online channels in the form of websites and emails (79% versus 92%, p<0.001), and more had received face-to-face information compared to those with tertiary education (43% versus 35%, p<0.001; Suppl. Table 34). However, both education level groups indicated that their preferred methods of communication were mass media channels, online methods and government/institutions' websites.

We asked respondents if they had seen unclear or conflicting information about COVID-19 in nine categories relating to infection, symptoms and various public health measures. Overall, between 36-

For peer review only - http://bmjopen.bmj.com/site/about/guidelines.xhtml

54% of respondents indicated that they had seen such information. Ways to avoid the infection (54%), government support schemes (52%) and testing (51%) were identified as the most unclear areas (Suppl. Table 35). Thailand reported the lowest levels of seeing unclear or conflicting information in most categories (around 35-40%), while respondents in the UK reported the highest levels in most categories (around 55-70%). Overall, those with tertiary education reported significantly higher levels of seeing unclear information than those with primary/secondary education in almost all categories ($p<0.001$) except government support schemes (Suppl. Table 36).

When asked “Have you come across news about the following COVID-19 topics that seemed fake to you?”, overall 63% of respondents had encountered news on “Coronavirus as an engineered modified virus”, 60% reported seeing “general spread of fear”, and 51% had come across seemingly fake news about “numbers of infected/deceased people”, “home-made recipes to make sanitizer products” and “alternative drugs/cure” (Fig. 5, Suppl. Table 35). Thailand reported the lowest percentages in all ‘fake news’ categories, with a range of 27-42% (Suppl. Table 35). Overall, respondents with tertiary education reported significantly higher levels of seeing ‘fake news’ in all categories compared to those with primary/secondary education ($p<0.001$; Fig. 5, Suppl. Table 36). For example, only 56% of people with primary/secondary education reported coming across fake news about “coronavirus as an engineered modified virus” versus 79% of those with tertiary education ($p<0.001$). There did not appear to be an association between self-reported levels of understanding of COVID-19 and seeing unclear/conflicting information or ‘fake news’ (Suppl. Table 37).

Discussion

Our results indicate how public health measures that were in place between 1st May and 30th June 2020 affected a cohort of over 5,000 respondents across five countries, and thus contribute new data and insights to these research areas.

Who was most affected by COVID-19 public health measures?

Overall, lower education levels, larger households, having children under 18 in the household, being 65 years or older, and having flexible/no income were associated with worse economic impact. This confirms that COVID-19 public health measures have greater negative impacts on already disadvantaged groups. Overall, it appeared that the 35-64 year old age group was less affected than 18-34 year olds and people older than 65 years. Possible explanations for this could be the types of sectors that younger and older people work in (e.g low paid or service industries)[32,33], or for older workers, shielding guidance issued by governments, lower levels of digital skills for remote working[34], or discrimination in the form of ageism[32,35]. There were no significant differences between gender groups in our overall analysis. However, other studies have shown that COVID-19 has had a greater impact on women (e.g. women are more likely to have temporary contracts[36,37] and

For peer review only - http://bmjopen.bmj.com/site/about/guidelines.xhtml

disproportionally carry the burden of unpaid care[38,39]). A more detailed gender analysis to further break down our survey results is currently underway.

Our results showed that among the countries surveyed, respondents from Thailand were most affected. Thailand is a middle-income country with a large informal economy, and relies heavily on the tourism industry (15% GDP)[40]. Thailand also had a high government stringency index during the period of the study (Fig. 1), which included closure of borders, businesses and nighttime curfews[41]. This meant that many informal street vendors and those working in the tourism industry (e.g. tour operators) had no income or lost their jobs.

Overall, about two thirds of respondents were most concerned about the effects of public health measures on their social life, their physical health, and their mental health and wellbeing. These findings resonate with other studies showing the substantial negative impact of COVID-19 restrictions on mental health, wellbeing and social life[42-44].

Self-reported compliance and behavioural changes

A number of quantitative online surveys have examined experiences, knowledge, attitude and perceptions towards COVID-19 and public health measures, at country level[36,45-54], and among different social groups[55-58]. Our findings show that self-reported compliance and behavioural change seemed to differ between countries. For example, respondents in Thailand indicated significantly higher levels of compliance, acceptance of public health measures and voluntary behavioural change compared to other countries. Although our survey was unable to implicate causality, it may contribute to better understanding of why Thailand has the lowest number of COVID cases relative to its population among the countries who took part in the survey[59]. Some of our results with regards to gender and age were similar to trends reported in other studies. For example, results from a Hong Kong study showed that female respondents, and those who reported higher levels of understanding of COVID-19, were more likely to adopt social distancing measures[60]. Similarly, a Chinese study found that men and those with a lower COVID-19 knowledge score were less likely to avoid crowded places or wear a mask outside. Using survey data from 27 countries, Daoust[55] observed that compliance was not higher in older people even though they might be expected to comply more due to being a risk group. Similarly, our data showed that overall and in Malaysia, UK and Slovenia, far fewer respondents over 65 years reported changing their behaviour voluntarily before official restrictions came into place. However, overall, over 80% of respondents in all three age groups expressed agreement when asked if they would comply voluntarily or with government-mandated restrictions (Suppl. Table 26).

For peer review only - http://bmjopen.bmj.com/site/about/guidelines.xhtml

Improving COVID-19 communication

Our findings indicated that younger age and lower education levels appeared to be associated with lower self-perceived/subjective levels of understanding of COVID-19. Also, higher self-reported levels of understanding of COVID-19 seemed to be associated with higher levels of understanding of public health measures. A recent modelling study suggests that self-imposed public health measures combined with fast spreading of disease awareness in the population can help reduce transmission of the virus[11]. Our findings suggest that specific groups of people, such as those with primary/secondary education levels and those 18-34 year old, may benefit most from targeted COVID-19 communication initiatives.

In terms of channels of communications, the three most popular channels across countries were traditional mass media, government or institutional websites, and online media. Similar results emerged from a recent survey carried out in the Netherlands, Germany and Italy[52]. However, respondents in Thailand reported that they preferred to receive information face-to-face, especially those with primary/secondary education. This suggests that in order for communication strategies to be effective, they need to be sensitive to population preferences and tailored to local contextual factors (e.g. levels of connectivity, literacy[61]).

Our survey showed that a significant proportion of the population received conflicting information and news that seemed fake to them, in particular about coronavirus being an engineered modified virus. These findings confirm other reports that misinformation and what has been termed the COVID-19 ‘infodemic’ is widespread[56,62,63]. More efforts should be made to curb misinformation and disinformation, taking into account the needs of different groups[44].

Strengths and limitations

Our online survey enabled us to capture people’s experiences and concerns in multiple domains, in five countries, all of which had restrictions in place, during the relatively early stage of the COVID-19 pandemic. To our knowledge, the SEBCOV study was one of the largest international mixed-methods studies conducted on the impact of COVID-19. To maximise the number of respondents and the likelihood of getting honest answers, the survey was completely anonymous. Due to the relatively large sample of respondents in each country, we were able to compare population segments (e.g. men versus women or younger versus older people) in our overall cohort and at country level. We did not aim to obtain nationally representative samples and acknowledge that although we used weighting strategies in our analysis, our results may not be fully representative of the populations in the respective countries. Overall, there was a high proportion of respondents who were healthcare workers (19%), and some variation in this proportion between countries. This may have influenced

For peer review only - http://bmjopen.bmj.com/site/about/guidelines.xhtml

the country level analysis, in particular in the areas of perceived understanding, compliance/agreement and communication preferences.

Because the survey was online, only people who were literate, had internet access, and had access to computers or smartphones could take part. Due to COVID-19 related restrictions, it was not possible to conduct face-to-face data collection to reach groups who were illiterate in the language of the survey, or who did not have access to online technology. This is likely to have biased our data towards more educated and economically advantaged populations. Our study was also subject to response bias and other biases arising from self-reporting and recall. Lastly, as a cross-sectional survey, our data only sheds light on the prevalence of certain phenomena and opinions of respondents but does not imply causality.

The results of the survey reported here form part of a mixed-methods study, which also includes an in-depth qualitative study, the findings of which are currently being analysed and will be published separately. Combined, our results may help explain some of the trends reported in this survey, as well as the differences between countries and social groups. We have also conducted a preliminary analysis of unweighted Thai survey responses during May 2020, which includes more detailed breakdowns by regions within Thailand[64].

Conclusion

NPIs such as lockdowns and social distancing measures to mitigate transmission of COVID-19 exert substantial negative economic and social impacts[44]. Our data confirmed that NPIs have unequal effects on different countries and different social groups within countries, and contributes to an important body of research showing that lockdowns most negatively affect those who are socio-economically disadvantaged[50,53]. As such, this study helps to expose some of the social and economic inequalities resulting from COVID-19 and public health measures. Our findings provide an indication of the social groups who may be most in need of support during pandemics, so that existing social inequalities are not perpetuated and worsened. Lastly, in order to mitigate the impacts of COVID-19, we need effective communication[19], and our data can help to inform future strategies.

Ethics approval

Ethics approval was granted by Oxford Tropical Research Ethics Committee (OxTREC, reference no.520-20), covering all countries; the Faculty of Tropical Medicine Ethics Committee, Thailand (FTMEC, ref: MUTM 2020-031-01); the Medical Research and Ethics Committee (MREC), Ministry of Health Malaysia (MOH), Malaysia, ref: NMRR-20-595-54437 (IIR), and the Universiti Tunku Abdul Rahman (Utar) Scientific and Ethical Review Committee (SERC, ref: (U/SERC/63/2020), Malaysia; and the National Medical Ethics Committee of the Republic of Slovenia (0120-

For peer review only - http://bmjopen.bmj.com/site/about/guidelines.xhtml

237/2020/7). Additional ethics committee approval from Italy was not required for the study to be conducted there.

Acknowledgements

We would like to thank all participants who took part in our study. We also thank the people who helped to test and promote the survey, and the members of the Bangkok Health Research and Ethics Group for their input into the study. We would like to thank the SoNAR-Global Network for the social science expertise in the development of this project and the ODK core team for their input in design of the quantitative survey. Lastly, we would like to thank Prof. Nicholas PJ Day, Prof Richard Maude and Dr Tamara Giles-Vernick for critical reading of the manuscript.

Data availability statement

The Mahidol Oxford Tropical Medicine Research Unit recognizes the value of sharing individual level data. We aim to ensure that data generated from all our research are collected, curated, managed and shared in a way that maximizes their benefit. Data underlying this publication are available upon request to the Mahidol Oxford Tropical Medicine Research Uni Data Access Committee at https://www.tropmedres.ac/units/moru-bangkok/bioethics-engagement/data-sharing.

Funding

This research was funded by a Wellcome Trust Institutional Translational Partnership Award (iTPA), Thailand [210559], and a Wellcome Trust Strategic Award [096527]. The Mahidol Oxford Tropical Medicine Research Unit is funded by the Wellcome Trust [106698]. This study was also supported by the Sonar-Global project which has received funding from the European Union Horizon 2020 Research and Innovation Program [825671]. The research programme in Slovenia is funded by the Slovenian Research Agency (Javna agencija za raziskovalno dejavnost RS) [P6-0279]

Conflicts of Interest

The authors declare no conflict of interest. The funders had no role in the design of the study; in the collection, analyses, or interpretation of data; in the writing of the manuscript, or in the decision to publish the results.

Contributorship statement

AO and PYC oversaw the whole project and wrote the initial draft of the manuscript. AO, GC, WP, PKC, PC, MS, MLS, TS, NW, SA, BN, SR, NK, DO, RC and PYC developed the survey and translations. AO, GC, WP, PC, LS led the project in the UK, Italy, Thailand, Malaysia and Slovenia,

For peer review only - http://bmjopen.bmj.com/site/about/guidelines.xhtml

respectively. MM and PP conducted the statistical analysis, figures and tables, with critical input from MS, AO and PYC. MLS critically reviewed the manuscript, figures and tables. All authors implemented the survey, contributed to the draft paper, and approved the final version of the paper. PYC conceived the project and is the guarantor of the paper.

Transparency declaration

The corresponding author (manuscript guarantor) affirms that this manuscript is an honest, accurate, and transparent account of the study being reported; that no important aspects of the study have been omitted; and that any discrepancies from the study as planned (and, if relevant, registered) have been explained.

For peer review only

Figure legends

Figure 1: Government stringency indices in Thailand, Malaysia, UK, Italy and Slovenia between 1st May – 30th June 2020. A higher score indicates a stricter government response, i.e. 100 = strictest[31].

Figure 2: Bar chart showing how respondents from the following demographic groups were affected economically by COVID-19: at country level (TH = Thailand, MY = Malaysia, UK = United Kingdom, IT = Italy, SI = Slovenia), gender (M = male, F = female, O = Other/prefer not to say); education level (P/S = primary or lower/secondary, T = tertiary); age (18-34 years old, 35-64 years old, 65+ years old); household size (1-5 people, ≥6 people); living with children under 18 years (Y = yes, N = no); and type of income (FBP = fixed/benefits/pension, CF = contract/freelance, O = other/no income).

Figure 3: Breakdown of responses to the question "Did you change your social behaviour before the implementation of government restrictions?" by country (TH = Thailand, MY = Malaysia, UK = United Kingdom, IT = Italy, SI = Slovenia) and demographic groups: gender (M = male, F = female, O = other/prefer not to say); education level (P/S = primary or lower/secondary, T = tertiary); age (18-34 years old, 35-64 years old, 65+ years old); self-reported/perceived level of understanding of COVID-19 (H = high/very high/expert level, S = some, N = a little/none at all).

Figure 4: Breakdown of responses to the question "How would you rate your level understanding of the current quarantine/isolation/social distancing requirements for COVID-19?" Self-

For peer review only - http://bmjopen.bmj.com/site/about/guidelines.xhtml

reported/perceived level of understanding of COVID-19 ((H = high/very high/expert level, S = some, N = a little/none at all) shown by country (TH = Thailand, MY = Malaysia, UK = United Kingdom, IT = Italy, SI = Slovenia) and demographic groups: gender (M = male, F = female, O = other/prefer not to say); age (18-34 years old, 35-64 years old, 65+ years old); education level (P/S = primary/secondary, T = tertiary); healthcare worker status (HCW = healthcare worker, Non-HCW = non-healthcare worker).

Figure 5: Diagram showing how many survey respondents had come across five 'fake news' categories, in response to the question "Have you come across news about the following COVID-19 topics that seemed fake to you?". Breakdown by country (TH = Thailand, MY = Malaysia, UK = United Kingdom, IT = Italy, SI = Slovenia), gender (M = male, F = female, O = other/prefer not to say), age (18-34 years old, 35-64 years old, 65+ years old), education level (P/S = primary or lower/secondary, T = tertiary), and perceived level of understanding of COVID-19 (H = high/very high/expert level, S = some, N = a little/none at all).

References

1. Guo G., Ye L., Pan K., *et al.* New insights of emerging SARS-CoV-2: epidemiology, etiology, clinical features, clinical treatment, and prevention. *Front Cell Dev Biol* 2020;8(410) doi: https://doi.org/10.3389/fcell.2020.00410

2. Wang L., Wang Y., Ye D., *et al.* Review of the 2019 novel coronavirus (SARS-CoV-2) based on current evidence. *Int J Antimicrob Agents* 2020;55(6):105948. doi: https://doi.org/10.1016/j.ijantimicag.2020.105948

3. Yan Y., Shin W. I., Pang Y. X., *et al.* The first 75 days of novel coronavirus (SARS-CoV-2) outbreak: recent advances, prevention, and treatment. *Int J Environ Res Public Health* 2020;17(7) doi: https://doi.org/10.3390/ijerph17072323

4. World Health Organization. Non-pharmaceutical public health measures for mitigating the risk and impact of epidemic and pandemic influenza. 2019. [Available from: https://www.who.int/influenza/publications/public_health_measures/publication/en/ accessed 9 October 2020].

5. Centers for Disease Control and Prevention. Non-pharmaceutical interventions (NPIs). 2020. [Available from: https://www.cdc.gov/nonpharmaceutical-interventions/index.html accessed 17th July 2020].

6. Aledort J. E., Lurie N., Wasserman J., *et al.* Non-pharmaceutical public health interventions for pandemic influenza: an evaluation of the evidence base. *BMC Public Health* 2007;7(1):208. doi: https://doi.org/10.1186/1471-2458-7-208

For peer review only - http://bmjopen.bmj.com/site/about/guidelines.xhtml

7. Martinez D. L., Das T. K. Design of non-pharmaceutical intervention strategies for pandemic influenza outbreaks. *BMC Public Health* 2014;14(1):1328. doi: https://doi.org/10.1186/1471-2458-14-1328

8. Ferguson N., Laydon D., Nedjati Gilani G.*, et al.* Report 9: Impact of non-pharmaceutical interventions (NPIs) to reduce COVID19 mortality and healthcare demand. 2020. doi: 10.25561/77482 [Available from: http://hdl.handle.net/10044/1/77482 accessed 9 October 2020].

9. Koo J. R., Cook A. R., Park M.*, et al.* Interventions to mitigate early spread of SARS-CoV-2 in Singapore: a modelling study. *Lancet Infect Dis* 2020;20(6):678-88. doi: https://doi.org/10.1016/S1473-3099(20)30162-6

10. Flaxman S., Mishra S., Gandy A.*, et al.* Estimating the effects of non-pharmaceutical interventions on COVID-19 in Europe. *Nature* 2020;584(7820):257-61. doi: https://doi.org/10.1038/s41586-020-2405-7

11. Teslya A., Pham T. M., Godijk N. G.*, et al.* Impact of self-imposed prevention measures and short-term government-imposed social distancing on mitigating and delaying a COVID-19 epidemic: a modelling study. *PLoS Med* 2020;17(7):e1003166. doi: https://doi.org/10.1371/journal.pmed.1003166

12. Doung-Ngern P., Suphanchaimat R., Panjangampatthana A.*, et al.* Case-control study of use of personal protective measures and risk for severe acute respiratory syndrome coronavirus 2 Infection, Thailand. *Emerging Infect Dis* 2020;26(11) doi: https://doi.org/10.3201/eid2611.203003

13. Lewnard J. A., Lo N. C. Scientific and ethical basis for social-distancing interventions against COVID-19. *Lancet Infect Dis* 2020;20(6):631-33. doi: https://doi.org/10.1016/S1473-3099(20)30190-0

14. Xafis V. 'What is Inconvenient for You is Life-saving for Me': How Health Inequities are playing out during the COVID-19 Pandemic. *Asian Bioeth Rev* 2020;12(2):223-34. doi: https://doi.org/10.1007/s41649-020-00119-1

15. Bavel J. J. V., Baicker K., Boggio P. S.*, et al.* Using social and behavioural science to support COVID-19 pandemic response. *Nat Hum Behav* 2020;4(5):460-71. doi: https://doi.org/10.1038/s41562-020-0884-z

16. Seale H., Dyer C. E. F., Abdi I.*, et al.* Improving the impact of non-pharmaceutical interventions during COVID-19: examining the factors that influence engagement and the impact on individuals. *BMC Infect Dis* 2020;20(1):607. doi: https://doi.org/10.1186/s12879-020-05340-9

17. World Health Organisation. A coordinated global research roadmap: 2019 novel coronavirus. 2020. [Available from: https://www.who.int/blueprint/priority-diseases/key-action/Coronavirus_Roadmap_V9.pdf accessed 9 October 2020].

18. Pan-Ngum W., Poomchaichote T., Cuman G.*, et al.* Social, ethical and behavioural aspects of COVID-19 [version 2; peer review: 2 approved]. *Wellcome Open Res* 2020;5(90) doi: https://doi.org/10.12688/wellcomeopenres.15813.2

19. Norton A., De La Horra Gozalo A., Feune De Colombi N.*, et al.* The remaining unknowns: a mixed methods study of the current and global health research priorities for COVID-19. *BMJ Glob Health* 2020;5(7):e003306. doi: http://dx.doi.org/10.1136/bmjgh-2020-003306

For peer review only - http://bmjopen.bmj.com/site/about/guidelines.xhtml

20. Osterrieder A., Poomchaichote T., Cuman G., *et al.* Online survey questions: Social, ethical and behavioural aspects of COVID-19 (Version Version 2.0 7 July 2020). 2020. [Available from: http://doi.org/10.5281/zenodo.4049821 accessed 25 September 2020].

21. JISC. Online surveys (formerly BOS). 2020. [Available from: https://www.onlinesurveys.ac.uk/ accessed 13 July 2020].

22. Cheah P. Y. Thailand "Asia and Africa Programme" Stakeholder Engagement Strategy 2020 - 2025 (Version Version 1, 19 Oct 2019). 2019. doi: http://doi.org/10.5281/zenodo.3510158

23. National Research Council. Cognitive aspects of survey methodology: building a bridge between disciplines. Washington, DC: The National Academies Press 1984.

24. Padlet. 2020. [Available from: http://padlet.com/ accessed 2 October 2020].

25. The Medical Chamber of Slovenia. 2020. [Available from: https://www.zdravniskazbornica.si/en/medical-chamber-of-slovenia accessed 2 October 2020].

26. Super Poll Thailand. Super Poll Thailand. 2020. [Available from: https://www.superpollthailand.net/ accessed 16 September 2020].

27. Facebook. About boosted posts. 2020. [Available from: https://www.facebook.com/business/help/240208966080581?id=352109282177656 accessed 25 September 2020].

28. Castro F. G., Kellison J. G., Boyd S. J., *et al.* A Methodology for Conducting Integrative Mixed Methods Research and Data Analyses. *J Mix Methods Res* 2010;4(4):342-60. doi: 10.1177/1558689810382916

29. Lutz W., Goujon A., Kc S., *et al.* Demographic and human capital scenarios for the 21st century: 2018 assessment for 201 countries. 2018. [Available from: https://ec.europa.eu/jrc/en/publication/demographic-and-human-capital-scenarios-21st-century-2018-assessment-201-countries accessed 9 October 2020].

30. Statacorp. Stata Statistical Software: Release 15. College Station, TX: StataCorp LLC, 2017.

31. Hale T., Webster S., Petherick A., *et al.* Oxford COVID-19 Government Response Tracker, Blavatnik School of Government. 2020. [Available from: https://covidtracker.bsg.ox.ac.uk/].

32. Alwin R. L., Schramm J. Coronavirus' devastating economic impact on workers age 50-plus. 2020. [Available from: https://www.aarp.org/politics-society/advocacy/info-2020/coronavirus-economic-impact-older-workers.html accessed 16 September 2020].

33. Business in the Community. COVID-19: economic impact on age in the workplace. 2020. [Available from: https://www.bitc.org.uk/fact-sheet/covid-19-economic-impact-on-age-in-the-workplace/ accessed 16 September 2020].

34. McIvor C. The risk older workers face in the wake of COVID-19. Nesta Blogs. 2020. [Available from: https://www.nesta.org.uk/blog/risk-older-workers-face-wake-covid-19/ accessed 13 October 2020].

35. Officer A., Schneiders M. L., Wu D., *et al.* Valuing older people: time for a global campaign to combat ageism. *Bull World Health Organ* 2016;94(10):710-10a. doi: https://doi.org/10.2471/blt.16.184960

For peer review only - http://bmjopen.bmj.com/site/about/guidelines.xhtml

36. Eurofound. Living, working and COVID-19: First findings – April 2020. 2020. [Available from: https://www.eurofound.europa.eu/publications/report/2020/living-working-and-covid-19-first-findings-april-2020 accessed 13 October 2020].

37. Burki T. The indirect impact of COVID-19 on women. *Lancet Infect Dis* 2020;20(8):904-05. doi: https://doi.org/10.1016/S1473-3099(20)30568-5

38. Anu M., Olivia W., Mekala K.*, et al.* COVID-19 and gender equality: Countering the regressive effects. 2020. [Available from: https://www.mckinsey.com/featured-insights/future-of-work/covid-19-and-gender-equality-countering-the-regressive-effects accessed 16 October 2020].

39. Power K. The COVID-19 pandemic has increased the care burden of women and families. *Sustainability: Science, Practice and Policy* 2020;16(1):67-73. doi: https://doi.org/10.1080/15487733.2020.1776561

40. World Bank Group. Thailand Economic Monitor: Thailand in the Time of COVID-19 (English). 2020. [Available from: http://documents.worldbank.org/curated/en/456171593190431246/Thailand-Economic-Monitor-Thailand-in-the-Time-of-COVID-19 accessed 13 October 2020].

41. Ministry of Public Health. Thailand's experience in the COVID-19 response. 2020. [Available from: https://ddc.moph.go.th/viralpneumonia/eng/file/pub_doc/LDoc9.pdf accessed 13 October 2020].

42. Brooks S. K., Webster R. K., Smith L. E.*, et al.* The psychological impact of quarantine and how to reduce it: rapid review of the evidence. *The Lancet* 2020;395(10227):912-20. doi: https://doi.org/10.1016/S0140-6736(20)30460-8

43. Pierce M., Hope H., Ford T.*, et al.* Mental health before and during the COVID-19 pandemic: a longitudinal probability sample survey of the UK population. *Lancet Psychiatry* 2020;7(10):883-92. doi: https://doi.org/10.1016/S2215-0366(20)30308-4

44. Social Science in Humanitarian Action Platform. Quarantine in the context of COVID-19. [Available from: https://www.socialscienceinaction.org/resources/february-2020-social-science-humanitarian-action-platform/ accessed 16 September 2020].

45. Azlan A. A., Hamzah M. R., Sern T. J.*, et al.* Public knowledge, attitudes and practices towards COVID-19: A cross-sectional study in Malaysia. *PLoS One* 2020;15(5):e0233668. doi: https://doi.org/10.1371/journal.pone.0233668

46. Lin Y., Hu Z., Alias H.*, et al.* Knowledge, attitudes, impact, and anxiety regarding COVID-19 infection among the public in China. *Front Public Health* 2020;8:236. doi: https://doi.org/10.3389/fpubh.2020.00236

47. Roy D., Tripathy S., Kar S. K.*, et al.* Study of knowledge, attitude, anxiety & perceived mental healthcare need in Indian population during COVID-19 pandemic. *Asian J Psychiatr* 2020;51:102083. doi: https://doi.org/10.1016/j.ajp.2020.102083

48. Geldsetzer P. Use of rapid online surveys to assess people's perceptions during infectious disease outbreaks: A cross-sectional survey on COVID-19. *J Med Internet Res* 2020;22(4):e18790. doi: https://doi.org/10.2196/18790

For peer review only - http://bmjopen.bmj.com/site/about/guidelines.xhtml

49. Zhong B. L., Luo W., Li H. M.*, et al.* Knowledge, attitudes, and practices towards COVID-19 among Chinese residents during the rapid rise period of the COVID-19 outbreak: a quick online cross-sectional survey. *Int J Biol Sci* 2020;16(10):1745-52. doi: 10.7150/ijbs.45221

50. Bonaccorsi G., Pierri F., Cinelli M.*, et al.* Economic and social consequences of human mobility restrictions under COVID-19. *Proc Natl Acad Sci U S A* 2020;117(27):15530-35. doi: https://doi.org/10.1073/pnas.2007658117

51. Murphy K., Williamson H., Sargeant E.*, et al.* Why people comply with COVID-19 social distancing restrictions: Self-interest or duty? *Aust N Z J Criminol* 2020;0(0):0004865820954484. doi: https://doi.org/10.1177/0004865820954484

52. Meier K., Glatz T., Guijt M. C.*, et al.* Public perspectives on protective measures during the COVID-19 pandemic in the Netherlands, Germany and Italy: A survey study. *PLoS One* 2020;15(8):e0236917. doi: https://doi.org/10.1371/journal.pone.0236917

53. Bezerra A. C. V., Silva C., Soares F. R. G.*, et al.* Factors associated with people's behavior in social isolation during the COVID-19 pandemic. *Cien Saude Colet* 2020;25(suppl 1):2411-21. doi: https://doi.org/10.1590/1413-81232020256.1.10792020

54. Daly M., Ebbinghaus B., Lehner L.*, et al.* Oxford Supertracker: The Global Directory for COVID Policy Trackers and Surveys, Department of Social Policy and Intervention. 2020. [Available from: https://supertracker.spi.ox.ac.uk/ accessed 9 October 2020].

55. Daoust J. F. Elderly people and responses to COVID-19 in 27 Countries. *PLoS One* 2020;15(7):e0235590. doi: https://doi.org/10.1371/journal.pone.0235590

56. Cuan-Baltazar J. Y., Muñoz-Perez M. J., Robledo-Vega C.*, et al.* Misinformation of COVID-19 on the internet: infodemiology study. *JMIR Public Health Surveill* 2020;6(2):e18444-e44. doi: https://doi.org/10.2196/18444

57. Biroli P., Bosworth S. J., Della Giusta M.*, et al.* Family Life in Lockdown. 2020. [Available from: https://www.iza.org/publications/dp/13398/family-life-in-lockdown accessed 13 October 2020].

58. Hamadani J. D., Hasan M. I., Baldi A. J.*, et al.* Immediate impact of stay-at-home orders to control COVID-19 transmission on socioeconomic conditions, food insecurity, mental health, and intimate partner violence in Bangladeshi women and their families: an interrupted time series. *Lancet Glob Health* 2020 doi: https://doi.org/10.1016/S2214-109X(20)30366-1

59. World Health Organisation. WHO Coronavirus Disease (COVID-19) Dashboard. 2020. [Available from: https://covid19.who.int/table accessed 16 September 2020].

60. Kwok K. O., Li K. K., Chan H. H. H.*, et al.* Community responses during early phase of COVID-19 epidemic, Hong Kong. *Emerg Infect Dis* 2020;26(7):1575-79. doi: https://dx.doi.org/10.3201/eid2607.200500

61. Vaughan E., Tinker T. Effective health risk communication about pandemic influenza for vulnerable populations. *Am J Public Health* 2009;99 Suppl 2(Suppl 2):S324-S32. doi: https://dx.doi.org/10.2105%2FAJPH.2009.162537

62. The Lancet Infectious Diseases. The COVID-19 infodemic. *Lancet Infect Dis* 2020;20(8):875. doi: 10.1016/S1473-3099(20)30565-X

For peer review only - http://bmjopen.bmj.com/site/about/guidelines.xhtml

762 63. Yusof A. N. M., Muuti M. Z., Ariffin L. A., *et al.* Sharing Information on COVID-19: the ethical challenges in the Malaysian setting. *Asian Bioeth Rev* 2020;12(3):349-61. doi: https://doi.org/10.1007/s41649-020-00132-4

765 64. Pan-Ngum W., Poomchaichote T., Peerawaranun P., *et al.* Perspectives on public health interventions in the management of the COVID-19 pandemic in Thailand [version 1; peer review: 1 approved with reservations]. *Wellcome Open Res* 2020;5(245) doi: https://doi.org/10.12688/wellcomeopenres.16293.1

For peer review only

For peer review only - http://bmjopen.bmj.com/site/about/guidelines.xhtml

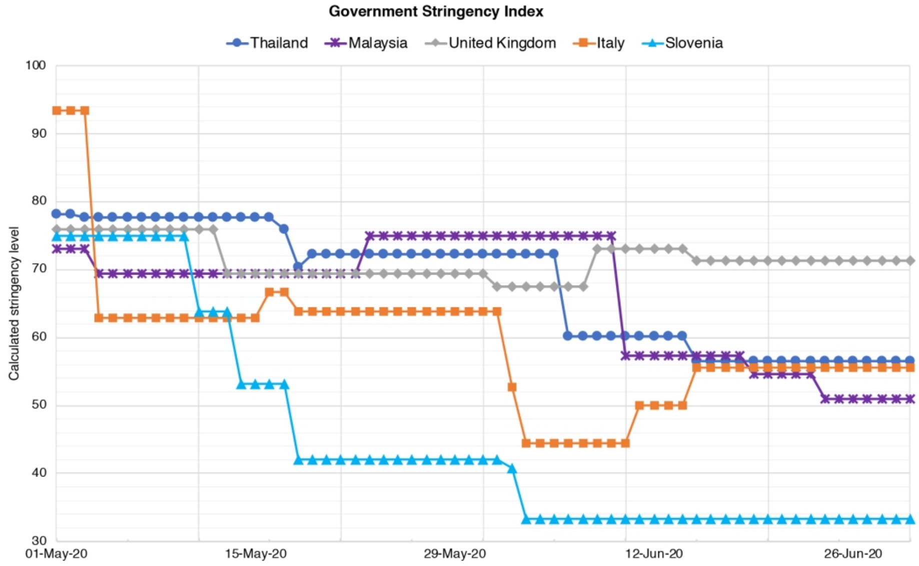

Figure 1: Government stringency indices in Thailand, Malaysia, UK, Italy and Slovenia between 1st May – 30th June 2020. A higher score indicates a stricter government response, i.e. 100 = strictest

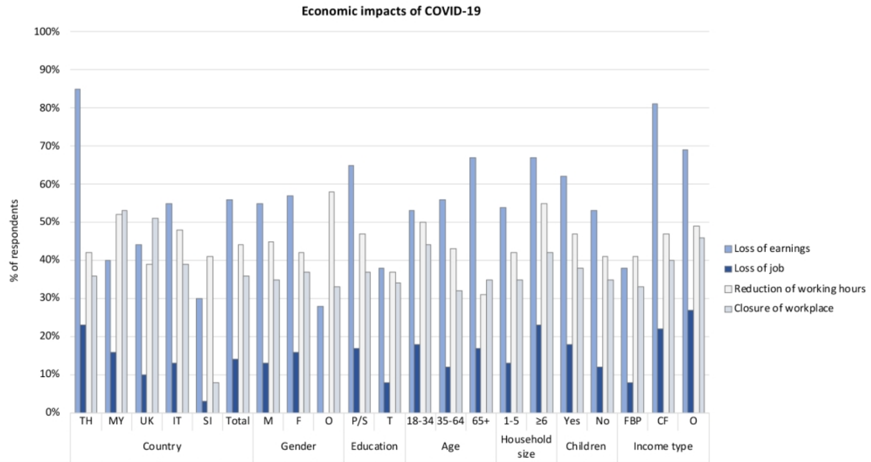

	TH	MY	UK	IT	SI	Total	M	F	O	P/S	T	18-34	35-64	65+	1-5	≥6	Yes	No	FBP	CF	O
	Country						Gender			Education		Age			Household size		Children		Income type		
Loss of earnings	85%	40%	44%	55%	30%	56%	55%	57%	28%	65%	38%	53%	56%	67%	54%	67%	62%	53%	38%	81%	69%
Loss of job	23%	16%	10%	13%	3%	14%	13%	16%	0%	17%	8%	18%	12%	17%	13%	23%	18%	12%	8%	22%	27%
Reduction of working hours	42%	52%	39%	48%	41%	44%	45%	42%	58%	47%	37%	50%	43%	31%	42%	55%	47%	41%	41%	47%	49%
Closure of workplace	36%	53%	51%	39%	8%	36%	35%	37%	33%	37%	34%	44%	32%	35%	35%	42%	38%	35%	33%	40%	46%

Figure 2: Bar chart showing how respondents from the following demographic groups were affected economically by COVID-19: at country level (TH = Thailand, MY = Malaysia, UK = United Kingdom, IT = Italy, SI = Slovenia), gender (M = male, F = female, O = Other/prefer not to say); education level (P/S = primary or lower/secondary, T = tertiary); age (18-34 years old, 35-64 years old, 65+ years old); household size (1-5 people, ≥6 people); living with children under 18 years (Y = yes, N = no); and type of income (FBP = fixed/benefits/pension, CF = contract/freelance, O = other/no income).

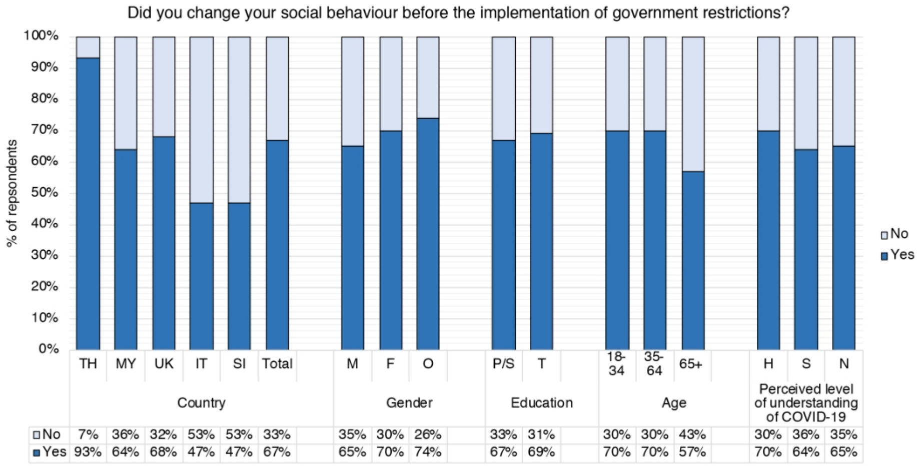

Figure 3: Breakdown of responses to the question "Did you change your social behaviour before the implementation of government restrictions?" by country (TH = Thailand, MY = Malaysia, UK = United Kingdom, IT = Italy, SI = Slovenia) and demographic groups: gender (M = male, F = female, O = other/prefer not to say); education level (P/S = primary or lower/secondary, T = tertiary); age (18-34 years old, 35-64 years old, 65+ years old); self-reported/perceived level of understanding of COVID-19 (H = high/very high/expert level, S = some, N = a little/none at all).

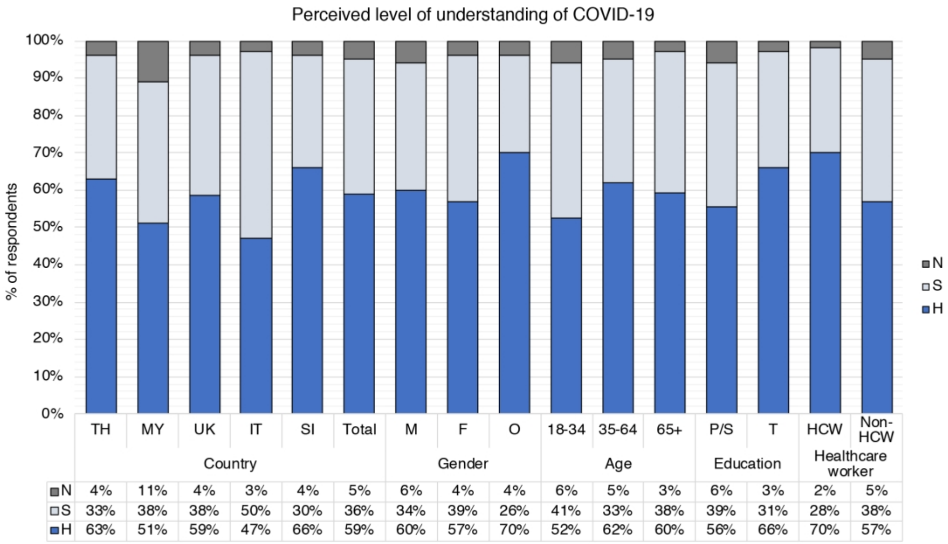

	TH	MY	UK	IT	SI	Total	M	F	O	18-34	35-64	65+	P/S	T	HCW	Non-HCW
N	4%	11%	4%	3%	4%	5%	6%	4%	4%	6%	5%	3%	6%	3%	2%	5%
S	33%	38%	38%	50%	30%	36%	34%	39%	26%	41%	33%	38%	39%	31%	28%	38%
H	63%	51%	59%	47%	66%	59%	60%	57%	70%	52%	62%	60%	56%	66%	70%	57%

Figure 4: Breakdown of responses to the question “How would you rate your level understanding of the current quarantine/isolation/social distancing requirements for COVID-19?” Self-reported/perceived level of understanding of COVID-19 ((H = high/very high/expert level, S = some, N = a little/none at all) shown by country (TH = Thailand, MY = Malaysia, UK = United Kingdom, IT = Italy, SI = Slovenia) and demographic groups: gender (M = male, F = female, O = other/prefer not to say); age (18-34 years old, 35-64 years old, 65+ years old); education level (P/S = primary/secondary, T = tertiary); healthcare worker status (HCW = healthcare worker, Non-HCW = non-healthcare worker).

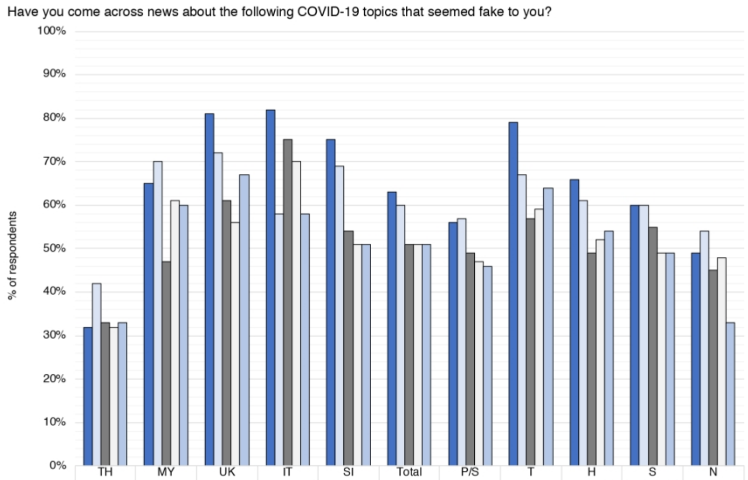

	TH	MY	UK	IT	SI	Total	P/S	T	H	S	N
	Country						Education		Perceived level of understanding of COVID-19		
Coronavirus as an engineered modified virus	32%	65%	81%	82%	75%	63%	56%	79%	66%	60%	49%
General spread of fear	42%	70%	72%	58%	69%	60%	57%	67%	61%	60%	54%
Numbers of infected/deceased people	33%	47%	61%	75%	54%	51%	49%	57%	49%	55%	45%
Home-made recipes to make sanitizer products	32%	61%	56%	70%	51%	51%	47%	59%	52%	49%	48%
Alternative drugs/cure	33%	60%	67%	58%	51%	51%	46%	64%	54%	49%	33%

Figure 5: Diagram showing how many survey respondents had come across five ‘fake news’ categories, in response to the question “Have you come across news about the following COVID-19 topics that seemed fake to you?”. Breakdown by country (TH = Thailand, MY = Malaysia, UK = United Kingdom, IT = Italy, SI = Slovenia), gender (M = male, F = female, O = other/prefer not to say), age (18-34 years old, 35-64 years old, 65+ years old), education level (P/S = primary or lower/secondary, T = tertiary), and perceived level of understanding of COVID-19 (H = high/very high/expert level, S = some, N = a little/none at all).

Supplementary tables for "Economic and social impacts of COVID-19 and public health measures: results from an anonymous online survey in Thailand, Malaysia, the United Kingdom, Italy and Slovenia"

Anne Osterrieder[1,2], Giulia Cuman[3], Wirichada Pan-ngum[1,4], Phaik Kin Cheah[5], Phee-Kheng Cheah[6], Pimnara Peerawaranun[1], Margherita Silan[7], Miha Orazem[8,9], Ksenija Perkovic[10], Urh Groselj[8,11], Mira Leonie Schneiders[1,2,12], Tassawan Poomchaichote[1,13], Naomi Waithira[1,2], Supa-at Asarath[1], Bhensri Naemiratch[1], Supanat Ruangkajorn[1], Lenart Skof[14], Natinee Kulpijit[1], Constance R.S. Mackworth-Young[15], Darlene Ongkili[16], Rita Chanviriyavuth[1], Mavuto Mukaka[1,2], Phaik Yeong Cheah[1,2,12,,13]

[1]Mahidol Oxford Tropical Medicine Research Unit, Faculty of Tropical Medicine, Mahidol University, Bangkok, 10400, Thailand
[2]Centre for Tropical Medicine & Global Health, Nuffield Department of Medicine, University of Oxford, Oxford, UK
[3]Paediatric Ethics Committee; Research Ethics Committee, University Hospital of Padua, Padua, Italy
[4]Department of Tropical Hygiene, Faculty of Tropical Medicine, Mahidol University, Bangkok, 10400, Thailand
[5]Faculty of Arts and Social Science, Universiti Tunku Abdul Rahman, Kampar, Malaysia
[6]Emergency and Trauma Department, Sabah Women and Children's Hospital, Ministry of Health Malaysia, Kota Kinabalu, Malaysia
[7]Department of Statistical Sciences, University of Padua, Padua, Italy
[8]Faculty of Medicine, University of Ljubljana, Ljubljana, Slovenia
[9]Department of Radiation Oncology, Institute of Oncology Ljubljana, Ljubljana, Slovenia
[10]Institute for Social Studies, Science and Research Centre Koper, Koper, Slovenia
[11]Department of Endocrinology, Diabetes and Metabolic Diseases, University Children's Hospital, University Medical Center, University Children's Hospital Ljubljana, Ljubljana, Slovenia
[12]Ethox Centre, Nuffield Department of Population Health, University of Oxford, Oxford, UK
[13]The SoNAR-Global Network
[14]Institute for Philosophical Studies, Science and Research Centre Koper, Koper, Slovenia
[15]Department of Global Health and Development, London School of Hygiene and Tropical Medicine, London, UK
[16]Emergency and Trauma Department, Queen Elizabeth Hospital, Ministry of Health Malaysia, Kota Kinabalu, Malaysia

Corresponding author: Phaik Yeong Cheah, 420/6 Mahidol-Oxford Tropical Medicine 24 Research Unit, Faculty of Tropical Medicine, Rajvithi Road, Bangkok, Thailand, 10400. Email: phaikyeong@tropmedres.ac

Notes

- There are a total of 37 tables in this document. Suppl. Table 1 reports the distribution of the basic demographic variables in the respondent sample (N= number of respondents), followed by unweighted percentages (unweighted %) in brackets. The values displayed in the cells in Suppl. Tables 2-37 show the number of respondents (N) who replied 'yes' to the respective survey categories, followed by weighted percentages (weighted %) in brackets.
- Because of rounding to the nearest integer, percentages do not always add up to 100% exactly.
- For gender, due to small number in the "other/prefer not to say" category, p-values are presented for comparison between the male and female groups only.

For peer review only - http://bmjopen.bmj.com/site/about/guidelines.xhtml

Contents

Supplementary tables for "Economic and social impacts of COVID-19 and public health measures: results from an anonymous online survey in Thailand, Malaysia, the United Kingdom, Italy and Slovenia" 1
Suppl. Table 1 Distribution of respondents by demographic characteristics and country (unweighted data) 4
Suppl. Table 2 Breakdown of economic impacts of COVID-19 and concerns by country 5
Suppl. Table 3 Breakdown of economic impacts of COVID-19 and concerns by country and gender 6
Suppl. Table 4 Breakdown of economic impacts of COVID-19 and concerns by country and education level 7
Suppl. Table 5 Breakdown of economic impacts of COVID-19 and concerns by country and age group 8
Suppl. Table 6 Breakdown of economic impacts of COVID-19 and concerns by country and household size 9
Suppl. Table 7 Breakdown of economic impacts of COVID-19 and concerns by country and whether or not living with children under 18 10
Suppl. Table 8 Breakdown of economic impacts of COVID-19 and concerns by country and type of income 11
Suppl. Table 9 Breakdown of concerns if advised/not allowed physical contact by country 12
Suppl. Table 10 Breakdown of concerns if advised/not allowed physical contact by country and gender 13
Suppl. Table 11 Breakdown of concerns if advised/not allowed physical contact by country and age group 14
Suppl. Table 12 Breakdown of concerns if advised/not allowed physical contact by country and education level 15
Suppl. Table 13 Breakdown of concerns if advised/not allowed physical contact by country and household size 16
Suppl. Table 14 Breakdown of concerns if advised/not allowed physical contact by country and whether or not living with children under 18 17
Suppl. Table 15 Breakdown of concerns if advised/not allowed physical contact by country and income type 18
Suppl. Table 16 Breakdown of maximum number of days that people thought they could cope by country 19
Suppl. Table 17 Breakdown of maximum number of days that people thought they could cope by country and gender 20
Suppl. Table 18 Breakdown of maximum number of days that people thought they could cope by country and age group 21
Suppl. Table 19 Breakdown of maximum number of days that people thought they could cope by country and household size 22
Suppl. Table 20 Breakdown of maximum number of days that people thought they could cope by country and whether or not living with children under 18 23
Suppl. Table 21 Breakdown of maximum number of days that people thought they could cope by country and education level 24
Suppl. Table 22 Breakdown of maximum number of days that people thought they could cope by country and type of income 25
Suppl. Table 23 Breakdown of behavioural changes and acceptance of government public health measures by country 26
Suppl. Table 24 Breakdown of behavioural changes and acceptance of government public health measures by country and gender 27
Suppl. Table 25 Breakdown of behavioural changes and acceptance of government public health measures by country and education level 29
Suppl. Table 26 Breakdown of behavioural changes and acceptance of government public health measures by age group 30

For peer review only

Suppl. Table 27 Breakdown of behavioural changes and acceptance of government public health measures by self-reported level of understanding of COVID-19 32

Suppl. Table 28 Breakdown of self-reported level of understanding of COVID-19 by country 34

Suppl. Table 29 Breakdown of self-reported level of understanding of COVID-19 by demographic characteristics 35

Suppl. Table 30 Breakdown of self-reported understanding of public health measures by self-reported level of understanding of COVID-19 36

Suppl. Table 31 What were the three most common ways people received communication on COVID-19, and what are the three most preferred ways to receive COVID-19 communications? Breakdown by country 37

Suppl. Table 32 What were the three most common ways people received communications on COVID-19, and what are the three most preferred ways to receive COVID-19 communications? Breakdown by country and gender 38

Suppl. Table 33 What were the three most common ways people received communications on COVID-19, and what are the three most preferred ways to receive COVID-19 communications? Breakdown by country and age group 39

Suppl. Table 34 What were the three most common ways people received communications on COVID-19, and what are the three most preferred ways to receive COVID-19 communications? Breakdown by country and education level 40

Suppl. Table 35 Most prevalent topic areas with unclear or conflicting COVID-19 information, and most prevalent 'fake news', breakdown by country 41

Suppl. Table 36 Most prevalent topic areas with unclear or conflicting COVID-19 information, and most prevalent 'fake news', breakdown by country and education level 42

Suppl. Table 37 Most prevalent topic areas with unclear or conflicting COVID-19 information, and most prevalent 'fake news', breakdown by country and self-reported level of understanding of COVID-19 43

For peer review only

Suppl. Table 1 Distribution of respondents by demographic characteristics and country (unweighted data)

Variable and categories	Thailand (N=1,476)	Malaysia (N=827)	UK (N=1,009)	Italy (N=712)	Slovenia (N=1,034)	Total (N=5,058)
**Gender**						
Male	704 (48)	298 (36)	426 (42)	222 (31)	366 (35)	2,016 (40)
Female	766 (52)	525 (63)	572 (57)	490 (69)	662 (64)	3,015 (60)
Other/prefer not to say	6 (0)	4 (0)	11 (1)	0 (0)	6 (1)	27 (1)
**Age (years)**						
18-34	223 (15)	350 (42)	140 (14)	272 (38)	308 (30)	1,293 (26)
35-64	1,152 (78)	442 (53)	616 (61)	383 (54)	676 (65)	3,269 (65)
65+	101 (7)	35 (4)	253 (25)	57 (8)	50 (5)	496 (10)
**Education level**						
Primary or lower/ secondary	909 (62)	82 (10)	247 (24)	217 (30)	202 (20)	1,657 (33)
Tertiary	567 (38)	745 (90)	762 (76)	495 (70)	832 (80)	3,401 (67)
**Household structure**						
Living alone	134 (9)	74 (9)	206 (20)	106 (15)	97 (9)	617 (12)
Living only with partner/spouse	173 (12)	95 (11)	391 (39)	192 (27)	210 (20)	1,061 (21)
Living with partner/spouse and children; living as single parent with children	847 (57)	312 (38)	260 (26)	188 (26)	518 (50)	2,125 (42)
Living with other relatives/non-relatives/other	322 (22)	346 (42)	152 (15)	226 (32)	209 (20)	1,255 (25)
**Household size**						
1	107 (7)	68 (8)	222 (22)	106 (15)	128 (12)	631 (12)
2	171 (12)	121 (15)	439 (44)	230 (32)	220 (21)	1,181 (23)
3-5	995 (67)	457 (55)	333 (33)	360 (51)	605 (59)	2,750 (54)
≥6	203 (14)	181 (22)	15 (1)	16 (2)	81 (8)	496 (10)
**Type of income**						
Fixed salary/benefits/pension	546 (37)	524 (63)	705 (70)	347 (49)	847 (82)	2,969 (59)
Contract and freelance	849 (58)	158 (19)	227 (22)	244 (34)	103 (10)	1,581 (31)
Other/no income	81 (5)	145 (18)	77 (8)	121 (17)	84 (8)	508 (10)
**Living with children under 18**	664 (45)	346 (42)	186 (18)	144 (20)	497 (48)	1,837 (36)
**Living with vulnerable group***	457 (31)	230 (28)	367 (36)	151 (21)	280 (27)	1,485 (29)
**Healthcare provider/worker****	239 (16)	213 (26)	118 (12)	64 (9)	341 (33)	975 (19)

Values in cells are n (%)
* Persons aged 70 or older; pregnant woman; people with serious health conditions
** Included respondents who were not working before COVID-19

For peer review only - http://bmjopen.bmj.com/site/about/guidelines.xhtml

Suppl. Table 2 Breakdown of economic impacts of COVID-19 and concerns by country

Values in cells are n (weighted %) of respondents who replied ‘yes’.

Variable and categories	Thailand	Malaysia	UK	Italy	Slovenia	Total	P-value
**If you were working before COVID-19, has COVID-19 created any inconvenience for you?**	N=1,255	N=613	N=630	N=526	N=929	N=3,953	
Loss of earnings	(N=1,248) 1,012 (85)	(N=556) 155 (40)	(N=584) 226 (44)	(N=496) 260 (55)	(N=867) 219 (30)	(N=3,751) 1,872 (56)	<0.001
Loss of job	(N=1,191) 233 (23)	(N=532) 44 (16)	(N=551) 51 (10)	(N=471) 59 (13)	(N=832) 15 (3)	(N=3,577) 402 (14)	<0.001
Reduction of working hours	(N=1,210) 492 (42)	(N=546) 228 (52)	(N=570) 201 (39)	(N=484) 233 (48)	(N=862) 319 (41)	(N=3,672) 1,473 (44)	0.107
Closure of workplace	(N=1,207) 425 (36)	(N=562) 289 (53)	(N=591) 296 (51)	(N=484) 167 (39)	(N=833) 63 (8)	(N=3,677) 1,240 (36)	<0.001
**Did you continue to work during COVID-19?**	(N=1,255) 1,019 (79)	(N=613) 532 (70)	(N=630) 460 (70)	(N=526) 388 (67)	(N=929) 768 (79)	(N=3,953) 3,167 (75)	0.011
**What are/were your concerns if advised no physical contact/not allowed to go out/allowed to go out only for essential needs?**	N=1,476	N=827	N=1,009	N=712	N=1,034	N=5,058	
Financial (e.g. loss of income, loss of job)	(N=1,466) 1,215 (86)	(N=775) 419 (60)	(N=950) 271 (32)	(N=678) 315 (41)	(N=1,015) 302 (28)	(N=4,884) 2,522 (53)	<0.001
Professional/ career progression	(N=1,414) 607 (42)	(N=759) 418 (52)	(N=942) 198 (24)	(N=670) 224 (22)	(N=1,001) 219 (17)	(N=4,786) 1,666 (32)	<0.001

Suppl. Table 3 Breakdown of economic impacts of COVID-19 and concerns by country and gender

M = male; F = female; O = other/prefer not to say. Values in cells are n (weighted %) of respondents who replied 'yes'.

**Variable and categories**	**Thailand**			**Malaysia**			**UK**			**Italy**			**Slovenia**			**Total**			
**Gender**	**M**	**F**	**O**	**M**	**F**	**O**	**M**	**F**	**O**	**M**	**F**	**O**	**M**	**F**	**O**	**M**	**F**	**O**	**P-value (for total M vs F)**
**If you were working before COVID-19, has COVID-19 created any inconvenience for you?**	N=606	N=645	N=4	N=230	N=380	N=3	N=261	N=363	N=6	N=184	N=342	N=0	N=332	N=591	N=6	N=1,613	N=2,321	N=19	
Loss of earnings	(N=604)<br>508 (83)	(N=640)<br>502 (86)	(N=4)<br>2 (50)	(N=210)<br>75 (42)	(N=343)<br>80 (37)	(N=3)<br>0 (0)	(N=245)<br>97 (45)	(N=333)<br>128 (43)	(N=6)<br>1 (17)	(N=177)<br>99 (54)	(N=319)<br>161 (57)		(N=314)<br>82 (29)	(N=548)<br>135 (31)	(N=5)<br>2 (40)	(N=1,550)<br>861 (55)	(N=2,183)<br>1,006 (57)	(N=18)<br>5 (28)	0.531
Loss of job	(N=576)<br>104 (20)	(N=611)<br>129 (25)	(N=4)<br>0 (0)	(N=202)<br>17 (18)	(N=327)<br>27 (15)	(N=3)<br>0 (0)	(N=233)<br>21 (19)	(N=313)<br>30 (11)	(N=5)<br>0 (0)	(N=168)<br>19 (10)	(N=303)<br>40 (17)		(N=301)<br>3 (1)	(N=526)<br>12 (4)	(N=5)<br>0 (0)	(N=1,480)<br>164 (13)	(N=2,080)<br>238 (16)	(N=17)<br>0 (0)	0.157
Reduction of working hours	(N=586)<br>225 (41)	(N=620)<br>265 (43)	(N=4)<br>2 (50)	(N=205)<br>85 (57)	(N=338)<br>141 (46)	(N=3)<br>2 (67)	(N=240)<br>90 (41)	(N=324)<br>107 (37)	(N=6)<br>4 (67)	(N=174)<br>94 (52)	(N=310)<br>139 (43)		(N=315)<br>128 (44)	(N=541)<br>188 (39)	(N=6)<br>3 (50)	(N=1,520)<br>622 (45)	(N=2,133)<br>840 (42)	(N=19)<br>11 (58)	0.179
Closure of workplace	(N=581)<br>194 (35)	(N=622)<br>231 (37)	(N=4)<br>0 (0)	(N=208)<br>109 (48)	(N=351)<br>178 (60)	(N=3)<br>2 (67)	(N=251)<br>124 (50)	(N=334)<br>169 (51)	(N=6)<br>3 (50)	(N=172)<br>65 (38)	(N=312)<br>102 (41)		(N=302)<br>19 (7)	(N=526)<br>43 (9)	(N=5)<br>1 (20)	(N=1,514)<br>511 (35)	(N=2,145)<br>723 (37)	(N=18)<br>6 (33)	0.365
Did you continue to work during COVID-19?	(N=606)<br>508 (84)	(N=645)<br>507 (75)	(N=4)<br>4 (100)	(N=230)<br>198 (67)	(N=380)<br>332 (73)	(N=3)<br>2 (67)	(N=261)<br>198 (72)	(N=363)<br>258 (67)	(N=6)<br>4 (67)	(N=184)<br>144 (74)	(N=342)<br>244 (60)		(N=332)<br>295 (85)	(N=591)<br>469 (74)	(N=6)<br>4 (67)	(N=1,613)<br>1,343 (78)	(N=2,321)<br>1,810 (71)	(N=19)<br>14 (74)	0.010
**What are/were your concerns if advised no physical contact/not allowed to go out/allowed to go out only for essential needs?**	N=704	N=766	N=6	N=298	N=525	N=4	N=261	N=363	N=6	N=222	N=490	N=0	N=366	N=662	N=6	N=2,016	N=3,015	N=27	
Financial	(N=700)<br>592 (85)	(N=760)<br>619 (86)	(N=6)<br>4 (67)	(N=279)<br>155 (62)	(N=492)<br>261 (59)	(N=4)<br>3 (75)	(N=411)<br>113 (34)	(N=529)<br>154 (31)	(N=10)<br>4 (40)	(N=214)<br>113 (44)	(N=464)<br>202 (38)		(N=361)<br>110 (27)	(N=648)<br>188 (29)	(N=6)<br>4 (67)	(N=1,965)<br>1,083 (54)	(N=2,893)<br>1,424 (53)	(N=26)<br>15 (58)	0.806
Professional/ career progression	(N=675)<br>278 (41)	(N=733)<br>326 (42)	(N=6)<br>3 (50)	(N=270)<br>137 (53)	(N=485)<br>279 (51)	(N=4)<br>2 (50)	(N=409)<br>84 (26)	(N=523)<br>108 (22)	(N=10)<br>6 (60)	(N=211)<br>92 (26)	(N=459)<br>132 (18)		(N=354)<br>77 (14)	(N=641)<br>141 (19)	(N=6)<br>1 (17)	(N=1,919)<br>668 (32)	(N=2,841)<br>986 (31)	(N=26)<br>12 (46)	0.597

Suppl. Table 4 Breakdown of economic impacts of COVID-19 and concerns by country and education level

P/S = primary or lower/secondary education; T = tertiary education. Values in cells are n (weighted %) of respondents who replied ‘yes’.

Variable and categories	Thailand		Malaysia		UK		Italy		Slovenia		Total		
**Education level**	**P/S**	**T**	**P/S**	**T**	**P/S**	**T**	**P/S**	**T**	**P/S**	**T**	**P/S**	**T**	**P-value (for total)**
**If you were working before COVID-19, has COVID-19 created any inconvenience for you?**	N=785	N=470	N=53	N=560	N=122	N=508	N=136	N=390	N=160	N=769	N=1,256	N=2,697	
Loss of earnings	(N=780)<br>725 (90)	(N=468)<br>287 (62)	(N=50)<br>21 (42)	(N=506)<br>134 (28)	(N=116)<br>55 (58)	(N=468)<br>171 (34)	(N=126)<br>75 (58)	(N=370)<br>185 (52)	(N=150)<br>56 (36)	(N=717)<br>163 (24)	(N=1,222)<br>932 (65)	(N=2,529)<br>940 (38)	<0.001
Loss of job	(N=744)<br>164 (24)	(N=447)<br>69 (16)	(N=50)<br>9 (19)	(N=482)<br>35 (7)	(N=108)<br>12 (13)	(N=443)<br>39 (9)	(N=123)<br>18 (14)	(N=348)<br>41 (12)	(N=140)<br>7 (4)	(N=692)<br>8 (1)	(N=1,165)<br>210 (17)	(N=2,412)<br>192 (8)	<0.001
Reduction of working hours	(N=762)<br>332 (43)	(N=448)<br>160 (37)	(N=48)<br>25 (55)	(N=498)<br>203 (40)	(N=110)<br>42 (49)	(N=460)<br>159 (32)	(N=125)<br>63 (47)	(N=359)<br>170 (49)	(N=144)<br>72 (46)	(N=718)<br>247 (35)	(N=1,189)<br>534 (47)	(N=2,483)<br>939 (37)	<0.001
Closure of workplace	(N=753)<br>262 (36)	(N=454)<br>163 (37)	(N=48)<br>28 (55)	(N=514)<br>261 (49)	(N=116)<br>51 (48)	(N=475)<br>245 (52)	(N=130)<br>59 (44)	(N=354)<br>108 (31)	(N=137)<br>14 (8)	(N=696)<br>49 (7)	(N=1,184)<br>414 (37)	(N=2,493)<br>826 (34)	0.180
Did you continue to work during COVID-19?	(N=785)<br>613 (78)	(N=470)<br>406 (86)	(N=53)<br>34 (65)	(N=560)<br>498 (90)	(N=122)<br>73 (59)	(N=508)<br>387 (77)	(N=136)<br>75 (59)	(N=390)<br>313 (79)	(N=160)<br>115 (74)	(N=769)<br>653 (85)	(N=1,256)<br>910 (71)	(N=2,697)<br>2,257 (83)	<0.001
**What are/were your concerns if advised no physical contact/not allowed to go out/allowed to go out only for essential needs?**	N=909	N=567	N=82	N=745	N=247	N=762	N=217	N=495	N=202	N=832	N=1,657	N=3,401	
Financial	(N=904)<br>828 (89)	(N=562)<br>387 (68)	(N=75)<br>46 (62)	(N=700)<br>373 (55)	(N=232)<br>64 (34)	(N=718)<br>207 (31)	(N=205)<br>96 (39)	(N=473)<br>219 (46)	(N=193)<br>71 (29)	(N=822)<br>231 (27)	(N=1,609)<br>1,105 (59)	(N=3,275)<br>1,417 (41)	<0.001
Professional/ career progression	(N=865)<br>326 (39)	(N=549)<br>281 (54)	(N=72)<br>36 (50)	(N=687)<br>382 (59)	(N=228)<br>21 (16)	(N=714)<br>177 (31)	(N=198)<br>42 (15)	(N=472)<br>182 (37)	(N=192)<br>37 (13)	(N=809)<br>182 (22)	(N=1,555)<br>462 (30)	(N=3,231)<br>1,204 (36)	0.004

For peer review only - http://bmjopen.bmj.com/site/about/guidelines.xhtml

Suppl. Table 5 Breakdown of economic impacts of COVID-19 and concerns by country and age group

Values in cells are n (weighted %) of respondents who replied 'yes'.

Variable and categories	Thailand			Malaysia			UK			Italy			Slovenia			Total			
**Age group**	**18-34**	**35-64**	**65+**	**18-34**	**35-64**	**65+**	**18-34**	**35-64**	**65+**	**18-34**	**35-64**	**65+**	**18-34**	**35-64**	**65+**	**18-34**	**35-64**	**65+**	**P-value (for total)**
**If you were working before COVID-19, has COVID-19 created any inconvenience for you?**	N=155	N=1,027	N=73	N=219	N=378	N=16	N=104	N=466	N=60	N=190	N=324	N=12	N=259	N=646	N=24	N=927	N=2,841	N=185	
Loss of earnings	(N=154) 103 (78)	(N=1,021) 851 (89)	(N=73) 58 (80)	(N=207) 48 (43)	(N=334) 98 (34)	(N=15) 9 (57)	(N=100) 32 (49)	(N=427) 168 (41)	(N=57) 26 (46)	(N=185) 97 (51)	(N=299) 155 (54)	(N=12) 8 (87)	(N=253) 67 (31)	(N=595) 144 (29)	(N=19) 8 (39)	(N=899) 347 (53)	(N=2,676) 1,416 (56)	(N=176) 109 (67)	0.102
Loss of job	(N=148) 36 (28)	(N=972) 183 (20)	(N=71) 14 (22)	(N=204) 22 (26)	(N=314) 20 (10)	(N=14) 2 (13)	(N=98) 10 (13)	(N=401) 35 (9)	(N=52) 6 (8)	(N=181) 22 (12)	(N=282) 35 (12)	(N=8) 2 (42)	(N=248) 6 (3)	(N=567) 9 (3)	(N=17) 0 (0)	(N=879) 96 (18)	(N=2,536) 282 (12)	(N=162) 24 (17)	0.054
Reduction of working hours	(N=147) 73 (53)	(N=991) 401 (42)	(N=72) 18 (23)	(N=206) 85 (57)	(N=325) 136 (49)	(N=15) 7 (50)	(N=100) 31 (43)	(N=416) 145 (36)	(N=54) 25 (45)	(N=182) 87 (50)	(N=292) 143 (50)	(N=10) 3 (16)	(N=249) 99 (47)	(N=593) 212 (39)	(N=20) 8 (38)	(N=884) 375 (50)	(N=2,617) 1,037 (43)	(N=171) 61 (31)	0.005
Closure of workplace	(N=151) 66 (46)	(N=984) 340 (35)	(N=72) 19 (24)	(N=207) 93 (55)	(N=340) 184 (48)	(N=15) 12 (83)	(N=100) 57 (56)	(N=434) 215 (49)	(N=57) 24 (44)	(N=185) 76 (49)	(N=289) 85 (32)	(N=10) 6 (86)	(N=246) 27 (14)	(N=570) 35 (6)	(N=17) 1 (3)	(N=889) 319 (44)	(N=2,617) 859 (32)	(N=171) 62 (35)	0.003
Did you continue to work during COVID-19?	(N=155) 120 (77)	(N=1,027) 838 (80)	(N=73) 61 (81)	(N=219) 195 (57)	(N=378) 330 (82)	(N=16) 7 (43)	(N=104) 79 (69)	(N=466) 346 (72)	(N=60) 35 (56)	(N=190) 134 (69)	(N=324) 250 (70)	(N=12) 4 (13)	(N=259) 209 (77)	(N=646) 540 (81)	(N=24) 19 (72)	(N=927) 737 (71)	(N=2,841) 2,304 (78)	(N=185) 126 (68)	0.025
**What are/were your concerns if advised no physical contact/not allowed to go out/allowed to go out only for essential needs?**	N=223	N=1,152	N=101	N=350	N=442	N=35	N=140	N=616	N=253	N=272	N=383	N=57	N=308	N=676	N=50	N=1,293	N=3,269	N=496	
Financial	(N=220) 161 (83)	(N=1,145) 985 (89)	(N=101) 69 (78)	(N=338) 198 (60)	(N=408) 211 (64)	(N=29) 10 (42)	(N=134) 59 (48)	(N=581) 195 (35)	(N=235) 17 (6)	(N=270) 138 (50)	(N=356) 168 (48)	(N=52) 9 (20)	(N=305) 92 (31)	(N=664) 205 (36)	(N=46) 5 (4)	(N=1,267) 648 (59)	(N=3,154) 1,764 (58)	(N=463) 110 (30)	<0.001
Professional/ career progression	(N=215) 126 (52)	(N=1,106) 452 (39)	(N=93) 29 (31)	(N=336) 238 (65)	(N=395) 173 (43)	(N=28) 7 (26)	(N=134) 76 (52)	(N=572) 118 (17)	(N=236) 4 (2)	(N=269) 122 (43)	(N=350) 99 (23)	(N=51) 3 (1)	(N=303) 108 (34)	(N=654) 109 (15)	(N=44) 2 (1)	(N=1,257) 670 (51)	(N=3,077) 951 (28)	(N=452) 45 (11)	<0.001

For peer review only

Suppl. Table 6 Breakdown of economic impacts of COVID-19 and concerns by country and <u>household size</u>

Values in cells are n (weighted %) of respondents who replied 'yes'.

**Variable and categories**	**Thailand**		**Malaysia**		**UK**		**Italy**		**Slovenia**		**Total**		
**Household size (number of persons in the household)**	**1-5**	**≥6**	**1-5**	**≥6**	**1-5**	**≥6**	**1-5**	**≥6**	**1-5**	**≥6**	**1-5**	**≥6**	**P-value (for total)**
**If you were working before COVID-19, has COVID-19 created any inconvenience for you?**	N=1,079	N=176	N=483	N=130	N=618	N=12	N=518	N=8	N=858	N=71	N=3,556	N=397	
Loss of earnings	(N=1,073)<br>864 (85)	(N=175)<br>148 (85)	(N=441)<br>120 (35)	(N=115)<br>35 (53)	(N=573)<br>221 (43)	(N=11)<br>5 (66)	(N=489)<br>256 (55)	(N=7)<br>4 (66)	(N=800)<br>201 (29)	(N=67)<br>18 (39)	(N=3,376)<br>1,662 (54)	(N=375)<br>210 (67)	0.013
Loss of job	(N=1,026)<br>190 (21)	(N=165)<br>43 (29)	(N=423)<br>29 (13)	(N=109)<br>15 (25)	(N=540)<br>51 (11)	(N=11)<br>0 (0)	(N=465)<br>59 (13)	(N=6)<br>0 (0)	(N=768)<br>14 (2)	(N=64)<br>1 (5)	(N=3,222)<br>343 (13)	(N=355)<br>59 (23)	0.009
Reduction of working hours	(N=1,043)<br>423 (42)	(N=167)<br>69 (59)	(N=434)<br>181 (44)	(N=112)<br>47 (72)	(N=558)<br>195 (38)	(N=12)<br>6 (57)	(N=477)<br>231 (52)	(N=7)<br>2 (50)	(N=792)<br>285 (39)	(N=70)<br>34 (61)	(N=3,304)<br>1,315 (42)	(N=368)<br>158 (55)	0.009
Closure of workplace	(N=1,039)<br>364 (36)	(N=168)<br>61 (34)	(N=443)<br>223 (47)	(N=119)<br>66 (72)	(N=579)<br>292 (51)	(N=12)<br>4 (25)	(N=476)<br>162 (39)	(N=8)<br>5 (72)	(N=768)<br>58 (8)	(N=65)<br>5 (7)	(N=3,305)<br>1,099 (35)	(N=372)<br>141 (42)	0.155
Did you continue to work during COVID-19?	(N=1,079)<br>884 (80)	(N=176)<br>135 (78)	(N=483)<br>424 (73)	(N=130)<br>108 (63)	(N=618)<br>450 (70)	(N=12)<br>10 (83)	(N=518)<br>384 (67)	(N=8)<br>4 (56)	(N=858)<br>712 (80)	(N=71)<br>56 (74)	(N=3,556)<br>2,854 (75)	(N=397)<br>313 (72)	0.564
**What are/were your concerns if advised no physical contact/not allowed to go out/allowed to go out only for essential needs?**	N=1,273	N=203	N=646	N=181	N=994	N=15	N=696	N=16	N=953	N=81	N=4,562	N=496	
Financial	(N=1,264)<br>1,050 (87)	(N=202)<br>165 (80)	(N=602)<br>317 (60)	(N=173)<br>102 (63)	(N=935)<br>266 (33)	(N=15)<br>5 (24)	(N=662)<br>306 (41)	(N=16)<br>9 (49)	(N=935)<br>282 (27)	(N=80)<br>20 (37)	(N=4,398)<br>2,221 (52)	(N=486)<br>301 (66)	0.003
Professional/ career progression	(N=1,220)<br>503 (40)	(N=194)<br>104 (49)	(N=593)<br>317 (51)	(N=166)<br>101 (56)	(N=928)<br>196 (24)	(N=14)<br>2 (9)	(N=654)<br>218 (22)	(N=16)<br>6 (28)	(N=920)<br>202 (16)	(N=81)<br>17 (21)	(N=4,315)<br>1,436 (30)	(N=471)<br>230 (46)	<0.001

For peer review only

Suppl. Table 7 Breakdown of economic impacts of COVID-19 and concerns by country and whether or not living with children under 18

Y = living with children under 18; N = not living with children under 18. Values in cells are n (weighted %) of respondents who replied ‘yes’.

**Variable and categories**	**Thailand**		**Malaysia**		**UK**		**Italy**		**Slovenia**		**Total**		
**Living with children under 18**	**Y**	**N**	**Y**	**N**	**Y**	**N**	**Y**	**N**	**Y**	**N**	**Y**	**N**	**P-value (for total)**
**If you were working before COVID-19, has COVID-19 created any inconvenience for you?**	N=546	N=709	N=276	N=337	N=158	N=472	N=112	N=414	N=462	N=467	N=1,554	N=2,399	
Loss of earnings	(N=545)<br>483 (91)	(N=703)<br>529 (79)	(N=239)<br>66 (44)	(N=317)<br>89 (37)	(N=144)<br>52 (46)	(N=440)<br>174 (43)	(N=98)<br>58 (61)	(N=398)<br>202 (54)	(N=428)<br>100 (30)	(N=439)<br>119 (31)	(N=1,454)<br>759 (62)	(N=2,297)<br>1,113 (53)	0.005
Loss of job	(N=525)<br>121 (27)	(N=666)<br>112 (19)	(N=227)<br>20 (26)	(N=305)<br>24 (10)	(N=139)<br>10 (13)	(N=412)<br>41 (9)	(N=92)<br>12 (9)	(N=379)<br>47 (14)	(N=409)<br>6 (3)	(N=423)<br>9 (3)	(N=1,392)<br>169 (18)	(N=2,185)<br>233 (12)	0.008
Reduction of working hours	(N=531)<br>240 (47)	(N=679)<br>252 (38)	(N=230)<br>102 (55)	(N=316)<br>126 (50)	(N=145)<br>48 (38)	(N=425)<br>153 (39)	(N=99)<br>48 (52)	(N=385)<br>185 (49)	(N=427)<br>165 (45)	(N=435)<br>154 (38)	(N=1,432)<br>603 (47)	(N=2,240)<br>870 (41)	0.047
Closure of workplace	(N=528)<br>216 (43)	(N=679)<br>209 (30)	(N=247)<br>141 (66)	(N=315)<br>148 (44)	(N=151)<br>73 (46)	(N=440)<br>223 (52)	(N=96)<br>39 (44)	(N=388)<br>128 (38)	(N=413)<br>27 (7)	(N=420)<br>36 (9)	(N=1,435)<br>496 (38)	(N=2,242)<br>744 (35)	0.268
Did you continue to work during COVID-19?	(N=546)<br>412 (74)	(N=709)<br>607 (84)	(N=276)<br>242 (65)	(N=337)<br>290 (74)	(N=158)<br>124 (71)	(N=472)<br>336 (69)	(N=112)<br>85 (73)	(N=414)<br>303 (65)	(N=462)<br>386 (81)	(N=467)<br>382 (78)	(N=1,554)<br>1,249 (74)	(N=2,399)<br>1,918 (75)	0.655
**What are/were your concerns if advised no physical contact/not allowed to go out/allowed to go out only for essential needs?**	N=664	N=812	N=346	N=481	N=186	N=823	N=144	N=568	N=497	N=537	N=1,837	N=3,221	
Financial	(N=660)<br>594 (92)	(N=806)<br>621 (80)	(N=323)<br>194 (59)	(N=452)<br>225 (62)	(N=174)<br>59 (35)	(N=776)<br>212 (32)	(N=135)<br>76 (61)	(N=543)<br>239 (37)	(N=486)<br>139 (33)	(N=529)<br>163 (24)	(N=1,778)<br>1,062 (64)	(N=3,106)<br>1,460 (47)	<0.001
Professional/ career progression	(N=637)<br>230 (37)	(N=777)<br>377 (45)	(N=315)<br>182 (53)	(N=444)<br>236 (51)	(N=171)<br>58 (35)	(N=771)<br>140 (21)	(N=134)<br>46 (35)	(N=536)<br>178 (19)	(N=483)<br>98 (19)	(N=518)<br>121 (15)	(N=1,740)<br>614 (35)	(N=3,046)<br>1,052 (30)	0.033

For peer review only

Suppl. Table 8 Breakdown of economic impacts of COVID-19 and concerns by country and type of income

FBP = fixed salary, benefits/pension; CF = contract and freelance; O = other/no income. Values in cells are n (weighted %) of respondents who replied 'yes'.

**Variable and categories**	**Thailand**			**Malaysia**			**UK**			**Italy**			**Slovenia**			**Total**			
**Type of income**	**FBP**	**CF**	**O**	**FBP**	**CF**	**O**	**FBP**	**CF**	**O**	**FBP**	**CF**	**O**	**FBP**	**CF**	**O**	**FBP**	**CF**	**O**	**P-value (for total)**
**If you were working before COVID-19, has COVID-19 created any inconvenience for you?**	N=495	N=738	N=22	N=475	N=125	N=13	N=397	N=210	N=23	N=278	N=228	N=20	N=788	N=101	N=40	N=2,433	N=1,402	N=118	
Loss of earnings	(N=493) 320 (74)	(N=733) 674 (91)	(N=22) 18 (89)	(N=428) 69 (26)	(N=117) 79 (65)	(N=11) 7 (92)	(N=361) 91 (28)	(N=200) 125 (67)	(N=23) 10 (50)	(N=253) 87 (39)	(N=224) 157 (75)	(N=19) 16 (95)	(N=731) 128 (21)	(N=96) 70 (77)	(N=40) 21 (53)	(N=2,266) 695 (38)	(N=1,370) 1,105 (81)	(N=115) 72 (69)	<0.001
Loss of job	(N=478) 78 (21)	(N=692) 148 (23)	(N=21) 7 (47)	(N=420) 18 (8)	(N=101) 24 (31)	(N=11) 2 (78)	(N=350) 20 (6)	(N=179) 30 (17)	(N=22) 1 (6)	(N=247) 6 (3)	(N=206) 45 (27)	(N=18) 8 (36)	(N=709) 6 (2)	(N=83) 5 (6)	(N=40) 4 (10)	(N=2,204) 128 (8)	(N=1,261) 252 (22)	(N=112) 22 (27)	<0.001
Reduction of working hours	(N=479) 226 (52)	(N=710) 259 (36)	(N=21) 7 (45)	(N=429) 163 (51)	(N=106) 60 (56)	(N=11) 5 (12)	(N=358) 89 (24)	(N=189) 102 (60)	(N=23) 10 (48)	(N=256) 111 (45)	(N=210) 113 (56)	(N=18) 9 (26)	(N=735) 227 (33)	(N=89) 67 (81)	(N=38) 25 (70)	(N=2,257) 816 (41)	(N=1,304) 601 (47)	(N=111) 56 (49)	0.042
Closure of workplace	(N=480) 195 (44)	(N=706) 224 (30)	(N=21) 6 (43)	(N=438) 214 (52)	(N=113) 67 (54)	(N=11) 8 (89)	(N=376) 188 (47)	(N=192) 98 (56)	(N=23) 10 (51)	(N=252) 63 (27)	(N=213) 94 (54)	(N=19) 10 (68)	(N=710) 33 (5)	(N=85) 20 (20)	(N=38) 10 (23)	(N=2,256) 693 (33)	(N=1,309) 503 (40)	(N=112) 44 (46)	0.015
Did you continue to work during COVID-19?	(N=495) 418 (83)	(N=738) 584 (77)	(N=22) 17 (78)	(N=475) 437 (83)	(N=125) 86 (42)	(N=13) 9 (25)	(N=397) 319 (79)	(N=210) 126 (57)	(N=23) 15 (62)	(N=278) 234 (81)	(N=228) 146 (51)	(N=20) 8 (15)	(N=788) 682 (84)	(N=101) 63 (57)	(N=40) 23 (59)	(N=2,433) 2,090 (82)	(N=1,402) 1,005 (65)	(N=118) 72 (53)	<0.001
**What are/were your concerns if advised no physical contact/not allowed to go out/allowed to go out only for essential needs?**	N=546	N=849	N=81	N=524	N=158	N=145	N=705	N=227	N=77	N=347	N=244	N=121	N=847	N=103	N=84	N=2,969	N=1,581	N=508	
Financial	(N=543) 402 (81)	(N=843) 753 (89)	(N=80) 60 (76)	(N=488) 231 (58)	(N=149) 110 (83)	(N=138) 78 (39)	(N=658) 131 (22)	(N=219) 116 (56)	(N=73) 24 (34)	(N=324) 102 (30)	(N=238) 165 (66)	(N=116) 48 (43)	(N=830) 190 (23)	(N=102) 74 (61)	(N=83) 38 (40)	(N=2,843) 1,056 (40)	(N=1,551) 1,218 (79)	(N=490) 248 (46)	<0.001
Professional/ career progression	(N=530) 221 (43)	(N=804) 348 (41)	(N=80) 38 (37)	(N=481) 247 (41)	(N=142) 81 (71)	(N=136) 90 (56)	(N=657) 104 (17)	(N=212) 66 (36)	(N=73) 28 (40)	(N=319) 71 (15)	(N=235) 112 (38)	(N=116) 41 (22)	(N=821) 156 (14)	(N=97) 35 (23)	(N=83) 28 (33)	(N=2,808) 799 (24)	(N=1,490) 642 (43)	(N=488) 225 (40)	<0.001

Suppl. Table 9 Breakdown of concerns if advised/not allowed physical contact by country

Values in cells are n (weighted %) of respondents who replied 'yes'.

Variable and categories	Thailand	Malaysia	UK	Italy	Slovenia	Total	P-value (for total)
**What are/were your concerns if advised no physical contact/not allowed to go out/allowed to go out only for essential needs?**	N=1,476	N=827	N=1,009	N=712	N=1,034	N=5,058	
Caring responsibilities (e.g. childcare, caring for elderly parents, not having access to care)	(N=1,454) 890 (62)	(N=772) 456 (57)	(N=946) 325 (31)	(N=681) 312 (46)	(N=1,006) 423 (35)	(N=4,859) 2,406 (47)	<0.001
Physical health (e.g. not being able to attend doctor appointments, medication supply for illnesses, lack of exercise)	(N=1,457) 910 (61)	(N=782) 501 (66)	(N=961) 587 (61)	(N=687) 393 (63)	(N=1,007) 437 (45)	(N=4,894) 2,828 (59)	<0.001
Recreational (e.g. not being able to access recreational facilities like cinemas or restaurants, cancelled sports or cultural events)	(N=1,425) 580 (38)	(N=763) 407 (49)	(N=963) 571 (58)	(N=683) 352 (47)	(N=1,011) 636 (65)	(N=4,845) 2,546 (51)	<0.001
Sports (e.g. participating in competitive or professional sports activities)	(N=1,400) 546 (38)	(N=755) 302 (39)	(N=943) 214 (22)	(N=675) 174 (24)	(N=997) 331 (36)	(N=4,770) 1,567 (32)	<0.001
Mental health and wellbeing (e.g. boredom, loneliness, anxiety, depression)	(N=1,427) 798 (55)	(N=769) 476 (61)	(N=970) 699 (75)	(N=691) 448 (60)	(N=1,008) 436 (43)	(N=4,865) 2,857 (58)	<0.001
Living arrangements (e.g. not enough living space, passing on illness to family members, domestic abuse)	(N=1,419) 646 (45)	(N=753) 289 (46)	(N=943) 215 (24)	(N=674) 114 (16)	(N=999) 177 (15)	(N=4,788) 1,441 (31)	<0.001
Infrastructure (e.g. access to transport, network services, internet access)	(N=1,409) 651 (46)	(N=750) 308 (45)	(N=935) 212 (24)	(N=672) 163 (28)	(N=996) 195 (19)	(N=4,762) 1,529 (33)	<0.001
Social (e.g. not being able to see friends or attend social or family events)	(N=1,440) 768 (52)	(N=773) 474 (56)	(N=974) 768 (79)	(N=686) 525 (70)	(N=1,015) 725 (69)	(N=4,888) 3,260 (64)	<0.001
Religious and spiritual (e.g. not being able to go to church, mosque, temple etc.)	(N=1,433) 591 (42)	(N=769) 393 (58)	(N=942) 162 (17)	(N=670) 95 (18)	(N=998) 201 (19)	(N=4,812) 1,442 (31)	<0.001

For peer review only - http://bmjopen.bmj.com/site/about/guidelines.xhtml

Suppl. Table 10 Breakdown of concerns if advised/not allowed physical contact by country and gender

M = male; F = female; O = other/prefer not to say. Values in cells are n (weighted %) of respondents who replied 'yes'.

Variable and categories	Thailand			Malaysia			UK			Italy			Slovenia			Total			
**Gender**	**M**	**F**	**O**	**M**	**F**	**O**	**M**	**F**	**O**	**M**	**F**	**O**	**M**	**F**	**O**	**M**	**F**	**O**	**P-value (for total M vs F)**
**What are/were your concerns if advised no physical contact/not allowed to go out/allowed to go out only for essential needs?**	N=704	N=766	N=6	N=298	N=525	N=4	N=426	N=572	N=11	N=222	N=490	N=0	N=366	N=662	N=6	N=2,016	N=3,015	N=27	
Caring responsibilities	(N=697) 430 (61)	(N=751) 456 (62)	(N=6) 4 (67)	(N=282) 170 (53)	(N=486) 284 (62)	(N=4) 2 (50)	(N=407) 124 (27)	(N=529) 197 (35)	(N=10) 4 (40)	(N=213) 82 (36)	(N=468) 230 (56)		(N=356) 124 (25)	(N=644) 297 (44)	(N=6) 2 (33)	(N=1,955) 930 (42)	(N=2,878) 1,464 (52)	(N=26) 12 (46)	<0.001
Physical health	(N=698) 443 (60)	(N=753) 463 (61)	(N=6) 4 (67)	(N=282) 184 (59)	(N=496) 314 (74)	(N=4) 3 (75)	(N=414) 255 (62)	(N=537) 323 (61)	(N=10) 9 (90)	(N=213) 106 (56)	(N=474) 287 (70)		(N=356) 148 (44)	(N=645) 287 (46)	(N=6) 2 (33)	(N=1,963) 1,136 (56)	(N=2,905) 1,674 (61)	(N=26) 18 (69)	0.058
Recreational	(N=681) 267 (39)	(N=738) 310 (38)	(N=6) 3 (50)	(N=275) 160 (54)	(N=484) 246 (44)	(N=4) 1 (25)	(N=411) 253 (61)	(N=542) 309 (56)	(N=10) 9 (90)	(N=215) 126 (54)	(N=468) 226 (41)		(N=359) 239 (71)	(N=646) 395 (59)	(N=6) 2 (33)	(N=1,941) 1,045 (54)	(N=2,878) 1,486 (47)	(N=26) 15 (58)	0.007
Sports	(N=670) 276 (40)	(N=724) 268 (35)	(N=6) 2 (33)	(N=275) 131 (47)	(N=476) 170 (29)	(N=4) 1 (25)	(N=410) 104 (23)	(N=524) 105 (21)	(N=9) 5 (56)	(N=212) 76 (32)	(N=463) 98 (17)		(N=353) 150 (44)	(N=638) 179 (28)	(N=6) 2 (33)	(N=1,920) 737 (38)	(N=2,825) 820 (27)	(N=25) 10 (40)	<0.001
Mental health and wellbeing	(N=684) 377 (55)	(N=737) 418 (55)	(N=6) 3 (50)	(N=279) 167 (62)	(N=486) 307 (61)	(N=4) 2 (50)	(N=414) 287 (73)	(N=545) 402 (77)	(N=11) 10 (91)	(N=216) 122 (56)	(N=475) 326 (63)		(N=357) 128 (40)	(N=645) 305 (46)	(N=6) 3 (50)	(N=1,950) 1,081 (57)	(N=2,888) 1,758 (60)	(N=27) 18 (67)	0.326
Living arrangements	(N=679) 323 (46)	(N=734) 320 (44)	(N=6) 3 (50)	(N=275) 106 (48)	(N=474) 182 (42)	(N=4) 1 (25)	(N=409) 79 (21)	(N=525) 131 (27)	(N=9) 5 (56)	(N=211) 40 (19)	(N=463) 74 (14)		(N=354) 53 (12)	(N=639) 121 (18)	(N=6) 3 (50)	(N=1,928) 601 (31)	(N=2,835) 828 (31)	(N=25) 12 (48)	0.948
Infrastructure	(N=672) 316 (46)	(N=731) 332 (47)	(N=6) 3 (50)	(N=276) 129 (42)	(N=470) 177 (48)	(N=4) 2 (50)	(N=407) 102 (27)	(N=520) 106 (21)	(N=8) 4 (50)	(N=209) 51 (29)	(N=463) 112 (27)		(N=353) 60 (14)	(N=637) 133 (24)	(N=6) 2 (33)	(N=1,917) 658 (32)	(N=2,821) 860 (34)	(N=24) 11 (46)	0.536
Social	(N=689) 369 (53)	(N=745) 395 (51)	(N=6) 4 (67)	(N=280) 179 (62)	(N=489) 294 (48)	(N=4) 1 (25)	(N=412) 321 (79)	(N=551) 438 (79)	(N=11) 9 (82)	(N=215) 163 (66)	(N=471) 362 (74)		(N=360) 245 (70)	(N=649) 475 (69)	(N=6) 5 (83)	(N=1,956) 1,277 (65)	(N=2,905) 1,964 (63)	(N=27) 19 (70)	0.503
Religious and spiritual	(N=689) 290 (41)	(N=738) 298 (44)	(N=6) 3 (50)	(N=279) 140 (55)	(N=486) 251 (61)	(N=4) 2 (50)	(N=408) 73 (19)	(N=524) 86 (14)	(N=10) 3 (30)	(N=208) 33 (21)	(N=462) 62 (15)		(N=355) 77 (24)	(N=637) 124 (14)	(N=6) 0 (0)	(N=1,939) 613 (33)	(N=2,847) 821 (30)	(N=26) 8 (31)	0.367

For peer review only - http://bmjopen.bmj.com/site/about/guidelines.xhtml

Suppl. Table 11 Breakdown of concerns if advised/not allowed physical contact by country and age group

Values in cells are n (weighted %) of respondents who replied 'yes'.

Variable and categories	Thailand			Malaysia			UK			Italy			Slovenia			Total			
**Age group**	**18-34**	**35-64**	**65+**	**18-34**	**35-64**	**65+**	**18-34**	**35-64**	**65+**	**18-34**	**35-64**	**65+**	**18-34**	**35-64**	**65+**	**18-34**	**35-64**	**65+**	**P-value (for total)**
**What are/were your concerns if advised no physical contact/not allowed to go out/allowed to go out only for essential needs?**	N=223	N=1,152	N=101	N=350	N=442	N=35	N=140	N=616	N=253	N=272	N=383	N=57	N=308	N=676	N=50	N=1,293	N=3,269	N=496	
Caring responsibilities	(N=217) 137 (71)	(N=1,138) 717 (64)	(N=99) 36 (37)	(N=333) 189 (56)	(N=407) 249 (57)	(N=32) 18 (66)	(N=131) 27 (20)	(N=581) 242 (41)	(N=234) 56 (23)	(N=270) 83 (30)	(N=361) 208 (55)	(N=50) 21 (43)	(N=304) 98 (30)	(N=656) 317 (44)	(N=46) 8 (16)	(N=1,255) 534 (46)	(N=3,143) 1,733 (53)	(N=461) 139 (32)	<0.001
Physical health	(N=218) 150 (63)	(N=1,139) 712 (63)	(N=100) 48 (47)	(N=336) 205 (60)	(N=413) 269 (65)	(N=33) 27 (98)	(N=134) 76 (61)	(N=586) 354 (60)	(N=241) 157 (64)	(N=270) 137 (45)	(N=365) 217 (57)	(N=52) 39 (90)	(N=305) 131 (40)	(N=655) 284 (42)	(N=47) 22 (59)	(N=1,263) 699 (56)	(N=3,158) 1,836 (57)	(N=473) 293 (66)	0.044
Recreational	(N=212) 121 (47)	(N=1,118) 425 (35)	(N=95) 34 (34)	(N=331) 183 (55)	(N=403) 209 (44)	(N=29) 15 (40)	(N=136) 96 (66)	(N=589) 339 (57)	(N=238) 136 (53)	(N=270) 169 (66)	(N=362) 166 (44)	(N=51) 17 (38)	(N=302) 213 (71)	(N=663) 395 (60)	(N=46) 28 (70)	(N=1,251) 782 (59)	(N=3,135) 1,534 (47)	(N=459) 230 (48)	0.003
Sports	(N=212) 99 (47)	(N=1,096) 428 (38)	(N=92) 19 (18)	(N=329) 140 (47)	(N=397) 154 (31)	(N=29) 8 (29)	(N=133) 40 (28)	(N=575) 133 (22)	(N=235) 41 (14)	(N=269) 93 (40)	(N=356) 74 (19)	(N=50) 7 (20)	(N=301) 114 (41)	(N=653) 206 (36)	(N=43) 11 (31)	(N=1,244) 486 (42)	(N=3,077) 995 (31)	(N=449) 86 (21)	<0.001
Mental health and wellbeing	(N=212) 146 (63)	(N=1,118) 613 (55)	(N=97) 39 (42)	(N=335) 230 (69)	(N=402) 227 (52)	(N=32) 19 (69)	(N=136) 118 (86)	(N=591) 439 (74)	(N=243) 142 (62)	(N=270) 191 (65)	(N=366) 227 (59)	(N=55) 30 (57)	(N=304) 169 (52)	(N=657) 253 (40)	(N=47) 14 (40)	(N=1,257) 854 (67)	(N=3,134) 1,759 (56)	(N=474) 244 (51)	<0.001
Living arrangements	(N=213) 105 (50)	(N=1,111) 518 (48)	(N=95) 23 (26)	(N=330) 142 (47)	(N=394) 137 (45)	(N=29) 10 (40)	(N=134) 47 (35)	(N=576) 144 (24)	(N=233) 24 (10)	(N=270) 60 (21)	(N=353) 52 (16)	(N=51) 2 (14)	(N=304) 76 (22)	(N=651) 100 (17)	(N=44) 1 (1)	(N=1,251) 430 (38)	(N=3,085) 951 (32)	(N=452) 60 (15)	<0.001
Infrastructure	(N=214) 117 (54)	(N=1,101) 502 (46)	(N=94) 32 (34)	(N=331) 149 (42)	(N=390) 152 (46)	(N=29) 7 (47)	(N=134) 37 (31)	(N=569) 133 (23)	(N=232) 42 (16)	(N=269) 59 (22)	(N=353) 91 (28)	(N=50) 13 (35)	(N=302) 63 (18)	(N=649) 121 (19)	(N=45) 11 (19)	(N=1,250) 425 (37)	(N=3,062) 999 (33)	(N=450) 105 (28)	0.112
Social	(N=216) 147 (59)	(N=1,126) 573 (50)	(N=98) 48 (46)	(N=334) 212 (55)	(N=408) 240 (55)	(N=31) 22 (60)	(N=136) 115 (83)	(N=592) 459 (77)	(N=246) 194 (79)	(N=268) 220 (84)	(N=366) 266 (69)	(N=52) 39 (63)	(N=304) 239 (79)	(N=662) 453 (65)	(N=49) 33 (69)	(N=1,258) 933 (69)	(N=3,154) 1,991 (62)	(N=476) 336 (64)	0.156
Religious and spiritual	(N=213) 86 (45)	(N=1,120) 468 (43)	(N=100) 37 (37)	(N=334) 180 (65)	(N=406) 198 (51)	(N=29) 15 (61)	(N=133) 14 (15)	(N=574) 111 (19)	(N=235) 37 (13)	(N=268) 27 (12)	(N=352) 64 (17)	(N=50) 4 (25)	(N=304) 51 (15)	(N=650) 142 (19)	(N=44) 8 (24)	(N=1,252) 358 (35)	(N=3,102) 983 (31)	(N=458) 101 (28)	0.198

Suppl. Table 12 Breakdown of concerns if advised/not allowed physical contact by country and <u>education level</u>

P/S = primary or lower/secondary education; T = tertiary education. Values in cells are n (weighted %) of respondents who replied 'yes'.

**Variable and categories**	**Thailand**		**Malaysia**		**UK**		**Italy**		**Slovenia**		**Total**		
**Education level**	**P/S**	**T**	**P/S**	**T**	**P/S**	**T**	**P/S**	**T**	**P/S**	**T**	**P/S**	**T**	**P-value (for total)**
**What are/were your concerns if advised no physical contact/not allowed to go out/allowed to go out only for essential needs?**	N=909	N=567	N=82	N=745	N=247	N=762	N=217	N=495	N=202	N=832	N=1,657	N=3,401	
Caring responsibilities	(N=894) 571 (63)	(N=560) 319 (57)	(N=74) 42 (57)	(N=698) 414 (60)	(N=231) 78 (30)	(N=715) 247 (32)	(N=204) 98 (47)	(N=477) 214 (45)	(N=190) 67 (31)	(N=816) 356 (40)	(N=1,593) 856 (49)	(N=3,266) 1,550 (43)	0.002
Physical health	(N=894) 565 (60)	(N=563) 345 (63)	(N=75) 53 (66)	(N=707) 448 (63)	(N=238) 146 (63)	(N=723) 441 (59)	(N=208) 123 (66)	(N=479) 270 (56)	(N=191) 78 (47)	(N=816) 359 (43)	(N=1,606) 965 (60)	(N=3,288) 1,863 (56)	0.045
Recreational	(N=870) 281 (34)	(N=555) 299 (57)	(N=72) 33 (47)	(N=691) 374 (55)	(N=236) 120 (52)	(N=727) 451 (64)	(N=204) 95 (45)	(N=479) 257 (52)	(N=192) 123 (66)	(N=819) 513 (62)	(N=1,574) 652 (46)	(N=3,271) 1,894 (60)	<0.001
Sports	(N=855) 317 (36)	(N=545) 229 (43)	(N=71) 25 (38)	(N=684) 277 (43)	(N=230) 34 (17)	(N=713) 180 (26)	(N=203) 44 (23)	(N=472) 130 (27)	(N=190) 75 (39)	(N=807) 256 (32)	(N=1,549) 495 (32)	(N=3,221) 1,072 (32)	0.953
Mental health and wellbeing	(N=877) 486 (54)	(N=550) 312 (59)	(N=74) 46 (61)	(N=695) 430 (62)	(N=238) 174 (76)	(N=732) 525 (74)	(N=209) 137 (58)	(N=482) 311 (63)	(N=190) 90 (45)	(N=818) 346 (40)	(N=1,588) 933 (58)	(N=3,277) 1,924 (60)	0.256
Living arrangements	(N=866) 422 (46)	(N=553) 224 (42)	(N=71) 32 (47)	(N=682) 257 (39)	(N=232) 46 (23)	(N=711) 169 (25)	(N=204) 37 (17)	(N=470) 77 (15)	(N=189) 36 (14)	(N=810) 141 (16)	(N=1,562) 573 (33)	(N=3,226) 868 (26)	<0.001
Infrastructure	(N=858) 396 (46)	(N=551) 255 (48)	(N=70) 32 (45)	(N=680) 276 (44)	(N=229) 44 (23)	(N=706) 168 (24)	(N=203) 55 (30)	(N=469) 108 (23)	(N=189) 35 (18)	(N=807) 160 (21)	(N=1,549) 562 (35)	(N=3,213) 967 (29)	0.004
Social	(N=887) 440 (49)	(N=553) 328 (62)	(N=72) 38 (54)	(N=701) 436 (63)	(N=242) 183 (77)	(N=732) 585 (80)	(N=207) 157 (67)	(N=479) 368 (77)	(N=194) 137 (69)	(N=821) 588 (70)	(N=1,602) 955 (60)	(N=3,286) 2,305 (73)	<0.001
Religious and spiritual	(N=882) 391 (44)	(N=551) 200 (36)	(N=71) 42 (60)	(N=698) 351 (51)	(N=232) 36 (17)	(N=710) 126 (17)	(N=202) 36 (20)	(N=468) 59 (13)	(N=190) 28 (18)	(N=808) 173 (21)	(N=1,577) 533 (35)	(N=3,235) 909 (24)	<0.001

Suppl. Table 13 Breakdown of concerns if advised/not allowed physical contact by country and <u>household size</u>

Values in cells are n (weighted %) of respondents who replied ‘yes’.

**Variable and categories**	**Thailand**		**Malaysia**		**UK**		**Italy**		**Slovenia**		**Total**		
**Household size (number of persons in household)**	**1-5**	**>=6**	**1-5**	**>=6**	**1-5**	**>=6**	**1-5**	**>=6**	**1-5**	**>=6**	**1-5**	**>=6**	**P-value (for total)**
**What are/were your concerns if advised no physical contact/not allowed to go out/allowed to go out only for essential needs?**	N=1,273	N=203	N=646	N=181	N=994	N=15	N=696	N=16	N=953	N=81	N=4,562	N=496	
Caring responsibilities	(N=1,251) 766 (62)	(N=203) 124 (59)	(N=603) 347 (61)	(N=169) 109 (46)	(N=931) 312 (30)	(N=15) 13 (80)	(N=665) 305 (46)	(N=16) 7 (43)	(N=925) 388 (34)	(N=81) 35 (42)	(N=4,375) 2,118 (47)	(N=484) 288 (53)	0.213
Physical health	(N=1,256) 792 (62)	(N=201) 118 (54)	(N=609) 390 (71)	(N=173) 111 (49)	(N=947) 579 (61)	(N=14) 8 (65)	(N=671) 383 (63)	(N=16) 10 (69)	(N=926) 408 (46)	(N=81) 29 (42)	(N=4,409) 2,552 (60)	(N=485) 276 (51)	0.060
Recreational	(N=1,229) 493 (38)	(N=196) 87 (39)	(N=596) 321 (49)	(N=167) 86 (49)	(N=949) 565 (58)	(N=14) 6 (53)	(N=667) 344 (47)	(N=16) 8 (42)	(N=930) 594 (65)	(N=81) 42 (55)	(N=4,371) 2,317 (51)	(N=474) 229 (46)	0.226
Sports	(N=1,207) 479 (39)	(N=193) 67 (31)	(N=587) 238 (36)	(N=168) 64 (46)	(N=929) 211 (22)	(N=14) 3 (10)	(N=659) 168 (24)	(N=16) 6 (34)	(N=917) 305 (36)	(N=80) 26 (34)	(N=4,299) 1,401 (32)	(N=471) 166 (36)	0.383
Mental health and wellbeing	(N=1,236) 697 (57)	(N=191) 101 (46)	(N=600) 369 (62)	(N=169) 107 (61)	(N=956) 690 (75)	(N=14) 9 (71)	(N=675) 436 (59)	(N=16) 12 (80)	(N=927) 409 (44)	(N=81) 27 (36)	(N=4,394) 2,601 (59)	(N=471) 256 (51)	0.096
Living arrangements	(N=1,224) 574 (48)	(N=195) 72 (34)	(N=585) 219 (44)	(N=168) 70 (50)	(N=928) 206 (23)	(N=15) 9 (60)	(N=658) 112 (16)	(N=16) 2 (20)	(N=918) 163 (15)	(N=81) 14 (17)	(N=4,313) 1,274 (30)	(N=475) 167 (38)	0.072
Infrastructure	(N=1,218) 564 (47)	(N=191) 87 (42)	(N=582) 233 (43)	(N=168) 75 (48)	(N=921) 209 (24)	(N=14) 3 (38)	(N=656) 160 (28)	(N=16) 3 (26)	(N=915) 184 (19)	(N=81) 11 (15)	(N=4,292) 1,350 (32)	(N=470) 179 (40)	0.113
Social	(N=1,243) 667 (52)	(N=197) 101 (49)	(N=602) 369 (51)	(N=171) 105 (68)	(N=959) 757 (79)	(N=15) 11 (76)	(N=670) 511 (70)	(N=16) 14 (78)	(N=934) 667 (70)	(N=81) 58 (68)	(N=4,408) 2,971 (65)	(N=480) 289 (60)	0.270
Religious and spiritual	(N=1,236) 511 (43)	(N=197) 80 (40)	(N=599) 296 (58)	(N=170) 97 (57)	(N=928) 159 (17)	(N=14) 3 (11)	(N=655) 92 (18)	(N=15) 3 (36)	(N=917) 169 (18)	(N=81) 32 (26)	(N=4,335) 1,227 (30)	(N=477) 215 (43)	0.005

Suppl. Table 14 Breakdown of concerns if advised/not allowed physical contact by country and whether or not living with children under 18

Values in cells are n (weighted %) of respondents who replied 'yes'.

**Variable and categories**	**Thailand**		**Malaysia**		**UK**		**Italy**		**Slovenia**		**Total**		
**Living with children under 18**	**Y**	**N**	**Y**	**N**	**Y**	**N**	**Y**	**N**	**Y**	**N**	**Y**	**N**	**P-value (for total)**
**What are/were your concerns if advised no physical contact/not allowed to go out/allowed to go out only for essential needs?**	N=664	N=812	N=346	N=481	N=186	N=823	N=144	N=568	N=497	N=537	N=1,837	N=3,221	
Caring responsibilities	(N=657)<br>487 (73)	(N=797)<br>403 (51)	(N=318)<br>217 (65)	(N=454)<br>239 (52)	(N=177)<br>109 (49)	(N=769)<br>216 (27)	(N=138)<br>88 (63)	(N=543)<br>224 (43)	(N=484)<br>278 (53)	(N=522)<br>145 (22)	(N=1,774)<br>1,179 (64)	(N=3,085)<br>1,227 (38)	<0.001
Physical health	(N=659)<br>458 (67)	(N=798)<br>452 (55)	(N=321)<br>199 (60)	(N=461)<br>302 (70)	(N=179)<br>103 (61)	(N=782)<br>484 (61)	(N=138)<br>77 (56)	(N=549)<br>316 (64)	(N=484)<br>217 (44)	(N=523)<br>220 (46)	(N=1,781)<br>1,054 (59)	(N=3,113)<br>1,774 (59)	0.984
Recreational	(N=644)<br>220 (36)	(N=781)<br>360 (41)	(N=316)<br>169 (48)	(N=447)<br>238 (49)	(N=179)<br>102 (55)	(N=784)<br>469 (59)	(N=139)<br>66 (40)	(N=544)<br>286 (49)	(N=486)<br>284 (60)	(N=525)<br>352 (68)	(N=1,764)<br>841 (46)	(N=3,081)<br>1,705 (53)	0.013
Sports	(N=633)<br>267 (41)	(N=767)<br>279 (35)	(N=318)<br>137 (45)	(N=437)<br>165 (34)	(N=173)<br>52 (24)	(N=770)<br>162 (21)	(N=135)<br>38 (29)	(N=540)<br>136 (23)	(N=478)<br>175 (41)	(N=519)<br>156 (33)	(N=1,737)<br>669 (39)	(N=3,033)<br>898 (29)	<0.001
Mental health and wellbeing	(N=641)<br>415 (63)	(N=786)<br>383 (48)	(N=318)<br>190 (56)	(N=451)<br>286 (65)	(N=180)<br>139 (80)	(N=790)<br>560 (74)	(N=139)<br>91 (60)	(N=552)<br>357 (60)	(N=481)<br>197 (44)	(N=527)<br>239 (43)	(N=1,759)<br>1,032 (59)	(N=3,106)<br>1,825 (58)	0.841
Living arrangements	(N=641)<br>366 (54)	(N=778)<br>280 (37)	(N=311)<br>118 (55)	(N=442)<br>171 (39)	(N=174)<br>56 (36)	(N=769)<br>159 (21)	(N=134)<br>24 (19)	(N=540)<br>90 (16)	(N=479)<br>93 (21)	(N=520)<br>84 (11)	(N=1,739)<br>657 (42)	(N=3,049)<br>784 (24)	<0.001
Infrastructure	(N=632)<br>322 (50)	(N=777)<br>329 (43)	(N=310)<br>131 (48)	(N=440)<br>177 (42)	(N=172)<br>37 (29)	(N=763)<br>175 (23)	(N=135)<br>30 (18)	(N=537)<br>133 (30)	(N=477)<br>81 (17)	(N=519)<br>114 (20)	(N=1,726)<br>601 (37)	(N=3,036)<br>928 (31)	0.018
Social	(N=651)<br>347 (52)	(N=789)<br>421 (52)	(N=322)<br>194 (53)	(N=451)<br>280 (57)	(N=179)<br>141 (82)	(N=795)<br>627 (78)	(N=140)<br>109 (77)	(N=546)<br>416 (69)	(N=488)<br>341 (69)	(N=527)<br>384 (70)	(N=1,780)<br>1,132 (61)	(N=3,108)<br>2,128 (66)	0.098
Religious and spiritual	(N=641)<br>307 (49)	(N=792)<br>284 (36)	(N=319)<br>174 (58)	(N=450)<br>219 (58)	(N=171)<br>30 (19)	(N=771)<br>132 (16)	(N=133)<br>23 (20)	(N=537)<br>72 (18)	(N=479)<br>118 (20)	(N=519)<br>83 (18)	(N=1,743)<br>652 (39)	(N=3,069)<br>790 (28)	<0.001

Suppl. Table 15 Breakdown of concerns if advised/not allowed physical contact by country and income type

FBP = fixed salary, benefits/pension; CF = contract and freelance; O = other/no income. Values in cells are n (weighted %) of respondents who replied 'yes'.

**Variable and categories**	**Thailand**			**Malaysia**			**UK**			**Italy**			**Slovenia**			**Total**			
**Type of income**	**FBP**	**CF**	**O**	**FBP**	**CF**	**O**	**FBP**	**CF**	**O**	**FBP**	**CF**	**O**	**FBP**	**CF**	**O**	**FBP**	**CF**	**O**	**P-value (for total)**
**What are/were your concerns if advised no physical contact/not allowed to go out/allowed to go out only for essential needs?**	N=546	N=849	N=81	N=524	N=158	N=145	N=705	N=227	N=77	N=347	N=244	N=121	N=847	N=103	N=84	N=2,969	N=1,581	N=508	
Caring responsibilities	(N=540) 372 (72)	(N=836) 481 (57)	(N=78) 37 (39)	(N=490) 307 (58)	(N=145) 78 (64)	(N=137) 71 (47)	(N=661) 223 (32)	(N=213) 83 (32)	(N=72) 19 (26)	(N=328) 167 (49)	(N=236) 101 (41)	(N=117) 44 (44)	(N=826) 362 (36)	(N=97) 42 (31)	(N=83) 19 (23)	(N=2,845) 1,431 (47)	(N=1,527) 785 (51)	(N=487) 190 (38)	0.028
Physical health	(N=543) 381 (70)	(N=835) 482 (56)	(N=79) 47 (49)	(N=497) 324 (63)	(N=146) 89 (71)	(N=139) 88 (66)	(N=672) 415 (62)	(N=216) 124 (60)	(N=73) 48 (63)	(N=333) 204 (68)	(N=236) 122 (51)	(N=118) 67 (59)	(N=826) 345 (44)	(N=98) 56 (58)	(N=83) 36 (42)	(N=2,871) 1,669 (59)	(N=1,531) 873 (58)	(N=492) 286 (57)	0.826
Recreational	(N=535) 243 (43)	(N=812) 296 (35)	(N=78) 41 (42)	(N=483) 253 (46)	(N=143) 78 (48)	(N=137) 76 (56)	(N=671) 386 (54)	(N=218) 134 (65)	(N=74) 51 (71)	(N=331) 153 (46)	(N=236) 136 (50)	(N=116) 63 (47)	(N=828) 511 (62)	(N=101) 63 (75)	(N=82) 62 (75)	(N=2,848) 1,546 (52)	(N=1,510) 707 (46)	(N=487) 293 (58)	0.024
Sports	(N=531) 264 (53)	(N=791) 249 (29)	(N=78) 33 (32)	(N=474) 190 (35)	(N=145) 63 (47)	(N=136) 49 (39)	(N=660) 133 (18)	(N=213) 57 (28)	(N=70) 24 (30)	(N=325) 72 (22)	(N=234) 70 (26)	(N=116) 32 (28)	(N=818) 265 (34)	(N=96) 34 (46)	(N=83) 32 (45)	(N=2,808) 924 (32)	(N=1,479) 473 (32)	(N=483) 170 (36)	0.582
Mental health and wellbeing	(N=533) 339 (65)	(N=816) 410 (50)	(N=78) 49 (50)	(N=485) 297 (61)	(N=146) 86 (58)	(N=138) 93 (66)	(N=676) 485 (75)	(N=221) 157 (74)	(N=73) 57 (80)	(N=335) 213 (60)	(N=238) 147 (55)	(N=118) 88 (68)	(N=826) 346 (43)	(N=99) 42 (38)	(N=83) 48 (53)	(N=2,855) 1,680 (59)	(N=1,520) 842 (55)	(N=490) 335 (63)	0.125
Living arrangements	(N=533) 268 (51)	(N=808) 352 (43)	(N=78) 26 (27)	(N=474) 181 (48)	(N=142) 54 (55)	(N=137) 54 (27)	(N=655) 128 (19)	(N=216) 65 (34)	(N=72) 22 (30)	(N=325) 57 (17)	(N=233) 38 (16)	(N=116) 19 (14)	(N=821) 138 (14)	(N=95) 15 (13)	(N=83) 24 (29)	(N=2,808) 772 (27)	(N=1,494) 524 (38)	(N=486) 145 (26)	<0.001
Infrastructure	(N=530) 279 (56)	(N=800) 335 (42)	(N=79) 37 (35)	(N=473) 179 (46)	(N=141) 55 (39)	(N=136) 74 (48)	(N=654) 134 (21)	(N=210) 56 (30)	(N=71) 22 (29)	(N=325) 74 (30)	(N=230) 56 (23)	(N=117) 33 (26)	(N=819) 157 (19)	(N=94) 15 (13)	(N=83) 23 (25)	(N=2,801) 823 (32)	(N=1,475) 517 (36)	(N=486) 189 (35)	0.370
Social	(N=537) 322 (58)	(N=824) 398 (48)	(N=79) 48 (51)	(N=491) 303 (55)	(N=146) 81 (59)	(N=136) 90 (52)	(N=681) 531 (78)	(N=219) 177 (79)	(N=74) 60 (81)	(N=335) 256 (72)	(N=233) 173 (63)	(N=118) 96 (78)	(N=834) 589 (68)	(N=98) 66 (67)	(N=83) 70 (86)	(N=2,878) 2,001 (67)	(N=1,520) 895 (58)	(N=490) 364 (67)	0.004
Religious and spiritual	(N=532) 235 (49)	(N=823) 326 (39)	(N=78) 30 (35)	(N=486) 254 (57)	(N=145) 68 (57)	(N=138) 71 (62)	(N=659) 121 (17)	(N=210) 31 (16)	(N=73) 10 (12)	(N=322) 43 (20)	(N=231) 36 (14)	(N=117) 16 (17)	(N=821) 168 (18)	(N=94) 22 (31)	(N=83) 11 (14)	(N=2,820) 821 (29)	(N=1,503) 483 (34)	(N=489) 138 (33)	0.195

Suppl. Table 16 Breakdown of maximum number of days that people thought they could cope by country

Values in cells are n (weighted %) of respondents who replied 'yes'.

Variable and categories	Thailand	Malaysia	UK	Italy	Slovenia	Total	P-value
**What is the maximum number of days you think you could cope without meeting family or friends not living in your household in person?**	N=1,476	N=827	N=1,009	N=712	N=1,034	N=5,058	<0.001
1 to 14 days	957 (66)	201 (31)	192 (21)	127 (23)	261 (34)	1,738 (39)	
>14 to 28 days	223 (13)	110 (16)	98 (11)	95 (14)	169 (16)	695 (14)	
29 days+	296 (21)	516 (52)	719 (68)	490 (63)	604 (50)	2,625 (47)	
**What is the maximum number of days you think you could cope with not going out in public, assuming that you have sufficient supplies of food, medicines and other essential items?**	N=1,476	N=827	N=1,009	N=712	N=1,034	N=5,058	<0.001
1 to 14 days	805 (54)	270 (41)	393 (40)	304 (45)	601 (61)	2,373 (49)	
>14 to 28 days	249 (17)	114 (16)	124 (14)	161 (21)	151 (13)	799 (16)	
29 days+	422 (29)	443 (43)	492 (46)	247 (34)	282 (26)	1,886 (35)	
**What is the maximum number of days you think you could cope with going out only for essential needs/work?**	N=1,476	N=827	N=1,009	N=712	N=1,034	N=5,058	<0.001
1 to 14 days	808 (56)	268 (40)	272 (29)	205 (33)	310 (37)	1,863 (41)	
>14 to 28 days	258 (17)	98 (14)	100 (10)	110 (17)	182 (18)	748 (15)	
29 days+	410 (26)	461 (46)	637 (60)	397 (51)	542 (45)	2,447 (44)	

For peer review only

Suppl. Table 17 Breakdown of maximum number of days that people thought they could cope by country and <u>gender</u>

M = male; F = female; O = other/prefer not to say. Values in cells are n (weighted %) of respondents who replied 'yes'.

**Variable and categories**	**Thailand**			**Malaysia**			**UK**			**Italy**			**Slovenia**			**Total**			
**Gender**	**M**	**F**	**O**	**M**	**F**	**O**	**M**	**F**	**O**	**M**	**F**	**O**	**M**	**F**	**O**	**M**	**F**	**O**	**P-value (for total M vs F)**
**What is the maximum number of days you think you could cope without meeting family or friends not living in your household in person?**	N=704	N=766	N=6	N=298	N=525	N=4	N=426	N=572	N=11	N=222	N=490	N=0	N=366	N=662	N=6	N=2,016	N=3,015	N=27	0.381
1 to 14 days	479 (66)	476 (66)	2 (33)	68 (29)	132 (34)	1 (25)	87 (23)	102 (19)	3 (27)	46 (28)	81 (18)		113 (38)	147 (31)	1 (17)	793 (40)	938 (37)	7 (26)	
>14 to 28 days	99 (12)	123 (15)	1 (17)	40 (14)	69 (18)	1 (25)	43 (13)	54 (9)	1 (9)	28 (11)	67 (17)		49 (14)	120 (18)	0 (0)	259 (13)	433 (15)	3 (11)	
29 days+	126 (23)	167 (19)	3 (50)	190 (57)	324 (48)	2 (50)	296 (64)	416 (72)	7 (64)	148 (61)	342 (65)		204 (48)	395 (51)	5 (83)	964 (47)	1,644 (47)	17 (63)	
**What is the maximum number of days you think you could cope with not going out in public, assuming that you have sufficient supplies of food, medicines and other essential items?**	N=704	N=766	N=6	N=298	N=525	N=4	N=426	N=572	N=11	N=222	N=490		N=366	N=662	N=6	N=2,016	N=3,015	N=27	0.890
1 to 14 days	398 (53)	405 (55)	2 (33)	96 (41)	173 (40)	1 (25)	170 (42)	219 (38)	4 (36)	100 (48)	204 (42)		217 (57)	382 (65)	2 (33)	981 (49)	1,383 (50)	9 (33)	
>14 to 28 days	116 (18)	132 (16)	1 (17)	47 (18)	66 (14)	1 (25)	53 (14)	71 (13)	0 (0)	46 (18)	115 (24)		40 (14)	111 (12)	0 (0)	302 (16)	495 (16)	2 (7)	
29 days+	190 (30)	229 (29)	3 (50)	155 (41)	286 (46)	2 (50)	203 (43)	282 (49)	7 (64)	76 (34)	171 (34)		109 (29)	169 (23)	4 (67)	733 (35)	1,137 (35)	16 (59)	
**What is the maximum number of days you think you could cope with going out only for essential needs/work?**	N=704	N=766	N=6	N=298	N=525	N=4	N=426	N=572	N=11	N=222	N=490		N=366	N=662	N=6	N=2,016	N=3,015	N=27	0.680
1 to 14 days	418 (57)	388 (55)	2 (33)	94 (41)	173 (38)	1 (25)	127 (32)	141 (27)	4 (36)	72 (35)	133 (31)		125 (35)	183 (40)	2 (33)	836 (42)	1,018 (40)	9 (33)	
>14 to 28 days	114 (17)	142 (17)	2 (33)	35 (11)	62 (17)	1 (25)	40 (10)	60 (10)	0 (0)	31 (17)	79 (17)		73 (23)	109 (13)	0 (0)	293 (16)	452 (15)	3 (11)	
29 days+	172 (25)	236 (27)	2 (33)	169 (47)	290 (45)	2 (50)	259 (58)	371 (62)	7 (64)	119 (49)	278 (52)		168 (43)	370 (47)	4 (67)	887 (42)	1,545 (45)	15 (56)	

For peer review only - http://bmjopen.bmj.com/site/about/guidelines.xhtml

Suppl. Table 18 Breakdown of maximum number of days that people thought they could cope by country and age group

Values in cells are n (weighted %) of respondents who replied ‘yes’.

Variable and categories	Thailand			Malaysia			UK			Italy			Slovenia			Total			
**Age group**	**18-34**	**35-64**	**65+**	**18-34**	**35-64**	**65+**	**18-34**	**35-64**	**65+**	**18-34**	**35-64**	**65+**	**18-34**	**35-64**	**65+**	**18-34**	**35-64**	**65+**	**P-value (for total)**
**What is the maximum number of days you think you could cope without meeting family or friends not living in your household in person?**	N=223	N=1,152	N=101	N=350	N=442	N=35	N=140	N=616	N=253	N=272	N=383	N=57	N=308	N=676	N=50	N=1,293	N=3,269	N=496	0.409
1 to 14 days	115 (57)	774 (70)	68 (67)	96 (32)	96 (25)	9 (55)	22 (22)	112 (18)	58 (24)	37 (19)	81 (26)	9 (19)	78 (29)	167 (31)	16 (49)	348 (36)	1,230 (39)	160 (42)	
>14 to 28 days	29 (10)	179 (15)	15 (15)	51 (19)	53 (13)	6 (22)	16 (13)	55 (10)	27 (12)	42 (20)	42 (11)	11 (17)	49 (17)	112 (15)	8 (18)	187 (10)	441 (13)	67 (16)	
29 days+	79 (33)	199 (15)	18 (18)	203 (49)	293 (62)	20 (23)	102 (65)	449 (72)	168 (64)	193 (62)	260 (63)	37 (64)	181 (54)	397 (54)	26 (34)	758 (50)	1,598 (48)	269 (42)	
**What is the maximum number of days you think you could cope with not going out in public, assuming that you have sufficient supplies of food, medicines and other essential items?**	N=223	N=1,152	N=101	N=350	N=442	N=35	N=140	N=616	N=253	N=272	N=383	N=57	N=308	N=676	N=50	N=1,293	N=3,269	N=496	0.335
1 to 14 days	113 (48)	643 (58)	49 (50)	116 (42)	141 (36)	13 (56)	62 (42)	222 (37)	109 (47)	111 (45)	170 (44)	23 (47)	192 (61)	382 (59)	27 (67)	594 (47)	1,558 (49)	221 (53)	
>14 to 28 days	33 (17)	192 (16)	24 (20)	43 (13)	65 (17)	6 (28)	19 (17)	85 (14)	20 (9)	65 (19)	82 (19)	14 (27)	36 (11)	107 (14)	8 (15)	196 (15)	531 (16)	72 (18)	
29 days+	77 (35)	317 (26)	28 (30)	191 (45)	236 (47)	16 (16)	59 (40)	309 (50)	124 (45)	96 (36)	131 (37)	20 (26)	80 (28)	187 (28)	15 (19)	503 (37)	1,180 (36)	203 (29)	
**What is the maximum number of days you think you could cope with going out only for essential needs/work?**	N=223	N=1,152	N=101	N=350	N=442	N=35	N=140	N=616	N=253	N=272	N=383	N=57	N=308	N=676	N=50	N=1,293	N=3,269	N=496	0.255
1 to 14 days	107 (52)	648 (59)	53 (56)	91 (32)	163 (43)	14 (62)	33 (28)	161 (27)	78 (36)	62 (27)	126 (36)	17 (32)	98 (34)	189 (33)	23 (51)	391 (37)	1,287 (42)	185 (46)	
>14 to 28 days	43 (18)	195 (17)	20 (17)	40 (13)	54 (14)	4 (15)	17 (12)	58 (10)	25 (8)	48 (20)	52 (14)	10 (20)	53 (17)	121 (17)	8 (19)	201 (16)	480 (15)	67 (16)	
29 days+	73 (30)	309 (24)	28 (27)	219 (55)	225 (43)	17 (22)	90 (60)	397 (63)	150 (56)	162 (53)	205 (51)	30 (48)	157 (49)	366 (50)	19 (29)	701 (48)	1,502 (43)	244 (38)	

For peer review only

Suppl. Table 19 Breakdown of maximum number of days that people thought they could cope by country and household size

Values in cells are n (weighted %) of respondents who replied ‘yes’.

**Variable and categories**	**Thailand**		**Malaysia**		**UK**		**Italy**		**Slovenia**		**Total**		
**Household size (number of persons in household)**	**1-5**	**≥6**	**1-5**	**≥6**	**1-5**	**≥6**	**1-5**	**≥6**	**1-5**	**≥6**	**1-5**	**≥6**	**P-value (for total)**
**What is the maximum number of days you think you could cope without meeting family or friends not living in your household in person?**	N=1,273	N=203	N=646	N=181	N=994	N=15	N=696	N=16	N=953	N=81	N=4,562	N=496	0.499
1 to 14 days	835 (68)	122 (56)	152 (30)	49 (36)	191 (21)	1 (4)	122 (23)	5 (44)	247 (35)	14 (24)	1,547 (38)	191 (43)	
>14 to 28 days	189 (13)	34 (15)	85 (16)	25 (17)	98 (11)	0 (0)	94 (15)	1 (3)	156 (16)	13 (15)	622 (14)	73 (15)	
29 days+	249 (19)	47 (29)	409 (54)	107 (47)	705 (68)	14 (96)	480 (63)	10 (53)	550 (49)	54 (61)	2,393 (48)	232 (42)	
**What is the maximum number of days you think you could cope with not going out in public, assuming that you have sufficient supplies of food, medicines and other essential items?**	N=1,273	N=203	N=646	N=181	N=994	N=15	N=696	N=16	N=953	N=81	N=4,562	N=496	0.298
1 to 14 days	712 (56)	93 (43)	209 (34)	61 (59)	389 (40)	4 (40)	296 (45)	8 (58)	558 (62)	43 (55)	2,164 (49)	209 (51)	
>14 to 28 days	211 (16)	38 (23)	86 (15)	28 (19)	121 (13)	3 (23)	159 (21)	2 (8)	139 (13)	12 (12)	716 (16)	83 (20)	
29 days+	350 (28)	72 (34)	351 (50)	92 (22)	484 (46)	8 (37)	241 (34)	6 (34)	256 (25)	26 (32)	1,682 (35)	204 (30)	
**What is the maximum number of days you think you could cope with going out only for essential needs/work?**	N=1,273	N=203	N=646	N=181	N=994	N=15	N=696	N=16	N=953	N=81	N=4,562	N=496	0.134
1 to 14 days	703 (57)	105 (55)	215 (37)	53 (51)	269 (29)	3 (37)	202 (33)	3 (29)	292 (38)	18 (24)	1,681 (40)	182 (49)	
>14 to 28 days	222 (18)	36 (16)	80 (15)	18 (9)	100 (11)	0 (0)	106 (17)	4 (20)	170 (18)	12 (11)	678 (16)	70 (12)	
29 days+	348 (26)	62 (29)	351 (48)	110 (40)	625 (60)	12 (63)	388 (51)	9 (51)	491 (44)	51 (64)	2,203 (44)	244 (39)	

For peer review only

Suppl. Table 20 Breakdown of maximum number of days that people thought they could cope by country and whether or not living with children under 18

Y = living with children under 18; N = not living with children under 18. Values in cells are n (weighted %) of respondents who replied 'yes'.

Variable and categories	Thailand		Malaysia		UK		Italy		Slovenia		Total		
**Living with children under 18**	**Y**	**N**	**Y**	**N**	**Y**	**N**	**Y**	**N**	**Y**	**N**	**Y**	**N**	**P-value (for total)**
**What is the maximum number of days you think you could cope without meeting family or friends not living in your household in person?**	N=664	N=812	N=346	N=481	N=186	N=823	N=144	N=568	N=497	N=537	N=1,837	N=3,221	<0.001
1 to 14 days	490 (72)	467 (60)	97 (40)	104 (25)	24 (14)	168 (22)	24 (18)	103 (24)	115 (30)	146 (38)	750 (46)	988 (35)	
>14 to 28 days	80 (10)	143 (17)	37 (12)	73 (19)	18 (12)	80 (11)	13 (9)	82 (16)	79 (14)	90 (18)	227 (12)	468 (16)	
29 days+	94 (18)	202 (23)	212 (47)	304 (56)	144 (74)	575 (67)	107 (73)	383 (61)	303 (57)	301 (45)	860 (42)	1,765 (50)	
**What is the maximum number of days you think you could cope with not going out in public, assuming that you have sufficient supplies of food, medicines and other essential items?**	N=664	N=812	N=346	N=481	N=186	N=823	N=144	N=568	N=497	N=537	N=1,837	N=3,221	<0.001
1 to 14 days	412 (59)	393 (49)	120 (57)	150 (29)	60 (36)	333 (41)	62 (44)	242 (45)	290 (62)	311 (60)	944 (56)	1,429 (46)	
>14 to 28 days	100 (16)	149 (18)	45 (11)	69 (20)	34 (19)	90 (12)	33 (26)	128 (20)	73 (13)	78 (14)	285 (15)	514 (17)	
29 days+	152 (25)	270 (33)	181 (33)	262 (51)	92 (46)	400 (46)	49 (31)	198 (34)	134 (25)	148 (26)	608 (29)	1,278 (38)	
**What is the maximum number of days you think you could cope with going out only for essential needs/work?**	N=664	N=812	N=346	N=481	N=186	N=823	N=144	N=568	N=497	N=537	N=1,837	N=3,221	0.004
1 to 14 days	407 (63)	401 (51)	117 (47)	151 (35)	33 (21)	239 (31)	42 (35)	163 (32)	139 (35)	171 (39)	738 (47)	1,125 (38)	
>14 to 28 days	112 (16)	146 (18)	37 (8)	61 (18)	17 (8)	83 (11)	20 (11)	90 (18)	90 (16)	92 (18)	276 (14)	472 (16)	
29 days+	145 (21)	265 (31)	192 (45)	269 (47)	136 (71)	501 (58)	82 (53)	315 (50)	268 (49)	274 (42)	823 (40)	1,624 (46)	

For peer review only

Suppl. Table 21 Breakdown of maximum number of days that people thought they could cope by country and <u>education level</u>

P/S = primary or lower/secondary education; T = tertiary education. Values in cells are n (weighted %) of respondents who replied ‘yes’.

**Variable and categories**	**Thailand**		**Malaysia**		**UK**		**Italy**		**Slovenia**		**Total**		
**Education level**	**P/S**	**T**	**P/S**	**T**	**P/S**	**T**	**P/S**	**T**	**P/S**	**T**	**P/S**	**T**	**P-value (for total)**
**What is the maximum number of days you think you could cope without meeting family or friends not living in your household in person?**	N=909	N=567	N=82	N=745	N=247	N=762	N=217	N=495	N=202	N=832	N=1,657	N=3,401	<0.001
1 to 14 days	659 (69)	298 (51)	27 (33)	174 (23)	55 (24)	137 (18)	53 (26)	74 (16)	69 (41)	192 (24)	863 (45)	875 (25)	
>14 to 28 days	122 (12)	101 (17)	15 (17)	95 (13)	30 (13)	68 (9)	31 (15)	64 (13)	33 (16)	136 (16)	231 (15)	464 (13)	
29 days+	128 (18)	168 (32)	40 (50)	476 (64)	162 (63)	557 (73)	133 (59)	357 (72)	100 (43)	504 (60)	563 (41)	2,062 (62)	
**What is the maximum number of days you think you could cope with not going out in public, assuming that you have sufficient supplies of food, medicines and other essential items?**	N=909	N=567	N=82	N=745	N=247	N=762	N=217	N=495	N=202	N=832	N=1,657	N=3,401	0.004
1 to 14 days	541 (56)	264 (47)	34 (43)	236 (32)	101 (41)	292 (40)	95 (46)	209 (43)	119 (63)	482 (58)	890 (51)	1,483 (45)	
>14 to 28 days	144 (17)	105 (18)	15 (17)	99 (13)	31 (15)	93 (13)	41 (20)	120 (24)	23 (12)	128 (15)	254 (16)	545 (16)	
29 days+	224 (28)	198 (35)	33 (40)	410 (55)	115 (44)	377 (48)	81 (34)	166 (33)	60 (25)	222 (27)	513 (33)	1,373 (39)	
**What is the maximum number of days you think you could cope with going out only for essential needs/work?**	N=909	N=567	N=82	N=745	N=247	N=762	N=217	N=495	N=202	N=832	N=1,657	N=3,401	<0.001
1 to 14 days	564 (59)	244 (43)	35 (43)	233 (29)	87 (35)	185 (24)	70 (35)	135 (29)	75 (42)	235 (31)	831 (46)	1,032 (30)	
>14 to 28 days	156 (17)	102 (19)	12 (14)	86 (11)	26 (10)	74 (10)	39 (18)	71 (14)	33 (17)	149 (18)	266 (16)	482 (14)	
29 days+	189 (24)	221 (38)	35 (43)	426 (59)	134 (54)	503 (66)	108 (48)	289 (57)	94 (41)	448 (51)	560 (38)	1,887 (56)	

For peer review only - http://bmjopen.bmj.com/site/about/guidelines.xhtml

Suppl. Table 22 Breakdown of maximum number of days that people thought they could cope by country and type of income

FBP = fixed salary, benefits/pension; CF = contract and freelance; O = other. Values in cells are n (weighted %) of respondents who replied 'yes'.

Variable and categories	Thailand			Malaysia			UK			Italy			Slovenia			Total			
Type of income	FBP	CF	O	FBP	CF	O	FBP	CF	O	FBP	CF	O	FBP	CF	O	FBP	CF	O	P-value (for total)
**What is the maximum number of days you think you could cope without meeting family or friends not living in your household in person?**	N=546	N=849	N=81	N=524	N=158	N=145	N=705	N=227	N=77	N=347	N=244	N=121	N=847	N=103	N=84	N=2,969	N=1,581	N=508	<0.001
1 to 14 days	344 (64)	577 (69)	36 (43)	135 (23)	35 (37)	31 (48)	134 (22)	36 (17)	22 (24)	58 (22)	47 (27)	22 (18)	208 (34)	35 (44)	18 (26)	879 (33)	730 (50)	129 (34)	
>14 to 28 days	74 (11)	134 (14)	15 (17)	57 (15)	24 (16)	29 (19)	69 (11)	25 (14)	4 (7)	46 (15)	30 (12)	19 (15)	141 (17)	19 (16)	9 (9)	387 (14)	232 (14)	76 (14)	
29 days+	128 (25)	138 (16)	30 (41)	332 (62)	99 (47)	85 (33)	502 (68)	166 (69)	51 (69)	243 (63)	167 (60)	80 (66)	498 (49)	49 (40)	57 (65)	1,703 (53)	619 (35)	303 (51)	
**What is the maximum number of days you think you could cope with not going out in public, assuming that you have sufficient supplies of food, medicines and other essential items?**	N=546	N=849	N=81	N=524	N=158	N=145	N=705	N=227	N=77	N=347	N=244	N=121	N=847	N=103	N=84	N=2,969	N=1,581	N=508	0.471
1 to 14 days	313 (55)	461 (55)	31 (39)	183 (38)	46 (39)	41 (49)	273 (40)	87 (41)	33 (42)	147 (45)	108 (47)	49 (40)	485 (560	66 (75)	50 (59)	1,401 (49)	768 (51)	204 (46)	
>14 to 28 days	85 (16)	148 (17)	16 (20)	70 (18)	22 (17)	22 (10)	90 (13)	28 (17)	6 (9)	84 (24)	55 (17)	22 (14)	129 (14)	12 (7)	10 (14)	458 (16)	265 (16)	76 (13)	
29 days+	148 (29)	240 (28)	34 (40)	271 (44)	90 (44)	82 (41)	342 (47)	112 (43)	38 (49)	116 (30)	81 (36)	50 (46)	233 (27)	25 (18)	24 (27)	1,110 (35)	548 (33)	228 (41)	
**What is the maximum number of days you think you could cope with going out only for essential needs/work?**	N=546	N=849	N=81	N=524	N=158	N=145	N=705	N=227	N=77	N=347	N=244	N=121	N=847	N=103	N=84	N=2,969	N=1,581	N=508	<0.001
1 to 14 days	297 (59)	478 (56)	33 (43)	181 (38)	56 (53)	31 (29)	186 (29)	64 (31)	22 (22)	99 (33)	78 (34)	28 (27)	250 (38)	41 (45)	19 (27)	1,013 (39)	717 (49)	133 (30)	
>14 to 28 days	81 (16)	159 (18)	18 (23)	54 (14)	23 (4)	21 (25)	68 (10)	20 (10)	12 (16)	55 (18)	30 (12)	25 (19)	150 (17)	17 (21)	15 (17)	408 (15)	249 (14)	91 (21)	
29 days+	168 (25)	212 (26)	30 (34)	289 (48)	79 (43)	93 (46)	451 (61)	143 (58)	43 (62)	193 (49)	136 (53)	68 (54)	447 (45)	45 (34)	50 (57)	1,548 (46)	615 (37)	284 (50)	

For peer review only - http://bmjopen.bmj.com/site/about/guidelines.xhtml

Suppl. Table 23 Breakdown of behavioural changes and acceptance of government public health measures by country

Values in cells are n (weighted %) of respondents who replied 'yes'.

Variable and categories	Thailand	Malaysia	UK	Italy	Slovenia	Total	P-value
	N=1,476	N=827	N=1,009	N=712	N=1,034	N=5,058	
**Did you change your social behaviour before the implementation of government restrictions?**	1,374 (93)	538 (64)	712 (68)	356 (47)	584 (47)	3,564 (67)	<0.001
**If you answered 'yes' to the previous question: how did you change your social behaviour?**							
No physical contact with anyone	(N=1,374) 1,302 (94)	(N=506) 362 (82)	(N=657) 325 (51)	(N=342) 243 (74)	(N=576) 516 (93)	(N=3,455) 2,748 (82)	<0.001
No physical contact only with elderly and those with serious underlying medical conditions	(N=1,374) 1,200 (88)	(N=494) 292 (63)	(N=644) 393 (60)	(N=332) 272 (79)	(N=566) 516 (91)	(N=3,410) 2,673 (79)	<0.001
Going out only for essential needs	(N=1,374) 1,291 (94)	(N=525) 489 (95)	(N=681) 571 (83)	(N=346) 263 (82)	(N=562) 381 (71)	(N=3,488) 2,995 (87)	<0.001
Moving home to stay with parents/relatives	(N=1,374) 677 (54)	(N=489) 99 (26)	(N=627) 30 (8)	(N=326) 27 (6)	(N=552) 33 (5)	(N=3,368) 866 (30)	<0.001
Use of personal protection equipment (e.g. masks and gloves)	(N=1,374) 1,334 (96)	(N=527) 488 (95)	(N=651) 225 (33)	(N=339) 165 (55)	(N=564) 366 (67)	(N=3,455) 2,578 (76)	<0.001
Use of sanitizer products and alcohol	(N=1,374) 1,321 (95)	(N=529) 504 (96)	(N=685) 559 (83)	(N=350) 307 (91)	(N=569) 521 (94)	(N=3,507) 3,212 (92)	<0.001
**"I would comply with government enforced quarantine/ isolation/social distancing."**	N=1,476	N=827	N=1,009	N=712	N=1,034	N=5,058	<0.001
Agree	1,344 (92)	708 (86)	822 (80)	606 (78)	871 (75)	4,351 (83)	
Neither agree nor disagree	92 (5)	18 (0)	48 (4)	36 (7)	68 (14)	262 (6)	
Disagree	40 (3)	101 (14)	139 (15)	70 (15)	95 (11)	445 (10)	
**"I would enter voluntary quarantine/isolation/social distancing for social/self-responsibility."**	N=1,476	N=827	N=1,009	N=712	N=1,034	N=5,058	<0.001
Agree	1,354 (92)	674 (81)	815 (78)	566 (76)	838 (76)	4,247 (82)	
Neither agree nor disagree	100 (7)	48 (4)	50 (5)	59 (10)	91 (13)	348 (8)	
Disagree	22 (1)	105 (15)	144 (17)	87 (14)	105 (11)	463 (10)	
**How much do you agree with quarantine/isolation/social distancing? "It is a necessary strategy to help control COVID-19."**	N=1,476	N=827	N=1,009	N=712	N=1,034	N=5,058	<0.001
Agree	1,383 (94)	739 (88)	853 (83)	608 (80)	846 (74)	4,429 (85)	
Neither agree nor disagree	65 (4)	12 (0)	27 (3)	28 (5)	76 (11)	208 (5)	
Disagree	28 (2)	76 (12)	129 (14)	76 (15)	112 (15)	421 (10)	

Suppl. Table 24 Breakdown of behavioural changes and acceptance of government public health measures by country and gender

M = male; F = female; O = other/prefer not to say. Values in cells are n (weighted %) of respondents who replied 'yes'.

**Variable and categories**	**Thailand**			**Malaysia**			**UK**			**Italy**			**Slovenia**			**Total**			
**Gender**	**M**	**F**	**O**	**M**	**F**	**O**	**M**	**F**	**O**	**M**	**F**	**O**	**M**	**F**	**O**	**M**	**F**	**O**	**P-value (for total M vs F)**
	N=704	N=766	N=6	N=298	N=525	N=4	N=426	N=572	N=11	N=222	N=490	N=0	N=366	N=662	N=6	N=2,016	N=3,015	N=27	
**Did you change your social behaviour before the implementation of government restrictions?**	660 (94)	709 (92)	5 (83)	184 (60)	351 (68)	3 (75)	288 (64)	415 (71)	9 (82)	99 (43)	257 (52)		179 (42)	402 (51)	3 (50)	1,410 (65)	2,134 (70)	20 (74)	0.039
**If you answered 'yes' to the previous question: how did you change your social behaviour?**																			
No physical contact with anyone	(N=660) 626 (93)	(N=709) 671 (95)	(N=5) 5 (100)	(N=173) 122 (75)	(N=330) 237 (87)	(N=3) 3 (100)	(N=271) 141 (51)	(N=379) 181 (50)	(N=7) 3 (43)	(N=94) 63 (68)	(N=248) 180 (78)		(N=175) 162 (94)	(N=398) 351 (892	(N=3) 3 (100)	(N=1,373) 1,114 (80)	(N=2,064) 1,620 (83)	(N=18) 14 (78)	0.227
No physical contact only with elderly and those with serious underlying medical conditions	(N=660) 584 (88)	(N=709) 611 (89)	(N=5) 5 (100)	(N=170) 104 (59)	(N=321) 186 (67)	(N=3) 2 (67)	(N=268) 148 (58)	(N=370) 243 (62)	(N=6) 2 (33)	(N=90) 75 (75)	(N=242) 197 (81)		(N=171) 152 (88)	(N=392) 361 (94)	(N=3) 3 (100)	(N=1,359) 1,063 (77)	(N=2,034) 1,598 (81)	(N=17) 12 (71)	0.124
Going out only for essential needs	(N=660) 612 (93)	(N=709) 674 (94)	(N=5) 5 (100)	(N=177) 164 (91)	(N=345) 322 (99)	(N=3) 3 (100)	(N=277) 234 (84)	(N=396) 330 (82)	(N=8) 7 (88)	(N=95) 71 (84)	(N=251) 192 (81)		(N=172) 113 (65)	(N=387) 265 (76)	(N=3) 3 (100)	(N=1,381) 1,194 (87)	(N=2,088) 1,783 (88)	(N=19) 18 (95)	0.327
Moving home to stay with parents/relatives	(N=660) 359 (59)	(N=709) 316 (49)	(N=5) 2 (40)	(N=167) 39 (27)	(N=319) 59 (24)	(N=3) 1 (33)	(N=267) 8 (3)	(N=354) 22 (11)	(N=6) 0 (0)	(N=91) 7 (3)	(N=235) 20 (9)		(N=167) 11 (3)	(N=382) 21 (6)	(N=3) 1 (33)	(N=1,352) 424 (32)	(N=1,999) 438 (28)	(N=17) 4 (24)	0.207
Use of personal protection equipment (e.g. masks and gloves)	(N=660) 639 (97)	(N=709) 690 (95)	(N=5) 5 (100)	(N=178) 160 (96)	(N=346) 325 (95)	(N=3) 3 (100)	(N=272) 101 (33)	(N=371) 121 (33)	(N=8) 3 (38)	(N=93) 38 (59)	(N=246) 127 (52)		(N=173) 122 (73)	(N=388) 241 (63)	(N=3) 3 (100)	(N=1,376) 1,060 (78)	(N=2,060) 1,504 (74)	(N=19) 14 (74)	0.079
Use of sanitizer products and alcohol	(N=660) 628 (95)	(N=709) 688 (95)	(N=5) 5 (100)	(N=178) 167 (96)	(N=348) 334 (96)	(N=3) 3 (100)	(N=278) 223 (80)	(N=398) 329 (85)	(N=9) 7 (78)	(N=96) 80 (92)	(N=254) 227 (91)		(N=173) 164 (94)	(N=393) 354 (94)	(N=3) 3 (100)	(N=1,385) 1,262 (92)	(N=2,102) 1,932 (93)	(N=20) 18 (90)	0.474
**"I would comply with government enforced quarantine/ isolation/social distancing."**	N=704	N=766	N=6	N=298	N=525	N=4	N=426	N=572	N=11	N=222	N=490		N=366	N=662	N=6	N=2,016	N=3,015	N=27	0.631
Agree	636 (92)	705 (93)	3 (50)	262 (93)	442 (78)	4 (100)	334 (76)	480 (85)	8 (73)	176 (69)	430 (86)		295 (75)	571 (75)	5 (83)	1,703 (82)	2,628 (84)	20 (74)	
Neither agree nor disagree	49 (6)	40 (4)	3 (50)	9 (1)	9 (0)	0 (0)	26 (6)	19 (3)	3 (27)	14 (10)	22 (5)		24 (10)	44 (17)	0 (0)	122 (6)	134 (6)	6 (22)	
Disagree	19 (2)	21 (3)	0 (0)	27 (7)	74 (22)	0 (0)	66 (18)	73 (12)	0 (0)	32 (21)	38 (9)		47 (15)	47 (8)	1 (17)	191 (11)	253 (10)	1 (4)	

For peer review only - http://bmjopen.bmj.com/site/about/guidelines.xhtml

**"I would enter voluntary quarantine/isolation/social distancing for social/self-responsibility."**	N=704	N=766	N=6	N=298	N=525	N=4	N=426	N=572	N=11	N=222	N=490		N=366	N=662	N=6	N=2,016	N=3,015	N=27	0.761
Agree	644 (91)	707 (92)	3 (50)	258 (93)	412 (68)	4 (100)	340 (78)	465 (78)	10 (91)	163 (67)	403 (85)		285 (76)	548 (77)	5 (83)	1,690 (83)	2,535 (81)	22 (81)	
Neither agree nor disagree	50 (8)	47 (7)	3 (50)	14 (1)	34 (8)	0 (0)	22 (5)	27 (5)	1 (9)	21 (14)	38 (6)		36 (9)	55 (15)	0 (0)	143 (7)	201 (8)	4 (15)	
Disagree	10 (1)	12 (1)	0 (0)	26 (6)	79 (25)	0 (0)	64 (17)	80 (16)	0 (0)	38 (19)	49 (9)		45 (15)	59 (8)	1 (17)	183 (10)	279 (10)	1 (4)	
**How much do you agree with quarantine/isolation/social distancing? "It is a necessary strategy to help control COVID-19."**	N=704	N=766	N=6	N=298	N=525	N=4	N=426	N=572	N=11	N=222	N=490		N=366	N=662	N=6	N=2,016	N=3,015	N=27	0.191
Agree	653 (93)	725 (95)	5 (83)	272 (93)	463 (83)	4 (100)	342 (77)	502 (88)	9 (82)	169 (68)	439 (91)		285 (75)	557 (74)	4 (67)	1,721 (83)	2,686 (87)	22 (81)	
Neither agree nor disagree	38 (5)	26 (3)	1 (17)	6 (0)	6 (0)	0 (0)	16 (4)	11 (3)	0 (0)	15 (9)	13 (2)		28 (7)	47 (15)	1 (17)	103 (5)	103 (5)	2 (7)	
Disagree	13 (1)	15 (2)	0 (0)	20 (6)	56 (17)	0 (0)	68 (19)	59 (10)	2 (18)	38 (23)	38 (8)		53 (18)	58 (12)	1 (17)	192 (12)	226 (9)	3 (11)	

Suppl. Table 25 Breakdown of behavioural changes and acceptance of government public health measures by country and <u>education level</u>

P/S = primary or lower/secondary education; T = tertiary education. Values in cells are n (weighted %) of respondents who replied ‘yes’.

**Variable and Categories**	**Thailand**		**Malaysia**		**UK**		**Italy**		**Slovenia**		**Total**		
**Education level**	**P/S**	**T**	**P/S**	**T**	**P/S**	**T**	**P/S**	**T**	**P/S**	**T**	**P/S**	**T**	**P-value (for total)**
	N=909	N=567	N=82	N=745	N=247	N=762	N=217	N=495	N=202	N=832	N=1,657	N=3,401	
**Did you change your social behaviour before the implementation of government restrictions?**	849 (93)	525 (92)	52 (64)	486 (65)	147 (60)	565 (74)	99 (46)	257 (52)	99 (41)	485 (56)	1,246 (67)	2,318 (69)	0.369
**If you answered 'yes' to the previous question: how did you change your social behaviour?**													
No physical contact with anyone	(N=849) 816 (95)	(N=525) 486 (91)	(N=47) 41 (85)	(N=459) 321 (70)	(N=138) 80 (59)	(N=519) 245 (45)	(N=90) 67 (76)	(N=252) 176 (71)	(N=97) 92 (96)	(N=479) 424 (90)	(N=1,221) 1,096 (87)	(N=2,234) 1,652 (70)	<0.001
No physical contact only with elderly and those with serious underlying medical conditions	(N=849) 771 (90)	(N=525) 429 (81)	(N=43) 29 (64)	(N=451) 263 (59)	(N=131) 76 (58)	(N=513) 317 (61)	(N=87) 73 (77)	(N=245) 199 (82)	(N=91) 83 (93)	(N=475) 433 (90)	(N=1,201) 1,032 (81)	(N=2,209) 1,641 (74)	0.003
Going out only for essential needs	(N=849) 798 (94)	(N=525) 493 (92)	(N=49) 47 (96)	(N=476) 442 (93)	(N=143) 122 (84)	(N=538) 449 (82)	(N=93) 69 (84)	(N=253) 194 (79)	(N=93) 66 (75)	(N=469) 315 (67)	(N=1,227) 1,102 (90)	(N=2,261) 1,893 (82)	<0.001
Moving home to stay with parents/relatives	(N=849) 515 (58)	(N=525) 162 (32)	(N=42) 11 (26)	(N=447) 88 (23)	(N=131) 5 (8)	(N=496) 25 (8)	(N=84) 10 (6)	(N=242) 17 (6)	(N=91) 4 (3)	(N=461) 29 (6)	(N=1,197) 545 (37)	(N=2,171) 321 (15)	<0.001
Use of personal protection equipment (e.g. masks and gloves)	(N=849) 819 (96)	(N=525) 515 (98)	(N=49) 47 (96)	(N=478) 441 (91)	(N=136) 55 (35)	(N=515) 170 (32)	(N=89) 49 (59)	(N=250) 116 (47)	(N=94) 57 (67)	(N=470) 309 (68)	(N=1,217) 1,027 (82)	(N=2,238) 1,551 (62)	<0.001
Use of sanitizer products and alcohol	(N=849) 813 (95)	(N=525) 508 (97)	(N=48) 46 (96)	(N=481) 458 (95)	(N=142) 120 (83)	(N=543) 439 (81)	(N=94) 84 (94)	(N=256) 223 (87)	(N=96) 92 (96)	(N=473) 429 (92)	(N=1,229) 1,155 (94)	(N=2,278) 2,057 (89)	<0.001
**“I would comply with government enforced quarantine/ isolation/social distancing.”**	N=909	N=567	N=82	N=745	N=247	N=762	N=217	N=495	N=202	N=832	N=1,657	N=3,401	0.315
Agree	843 (93)	501 (87)	70 (85)	638 (87)	190 (77)	632 (83)	178 (75)	428 (84)	148 (68)	723 (87)	1,429 (82)	2,922 (85)	
Neither agree nor disagree	43 (4)	49 (10)	0 (0)	18 (3)	14 (5)	34 (4)	9 (7)	27 (7)	22 (19)	46 (6)	88 (7)	174 (6)	
Disagree	23 (3)	17 (3)	12 (15)	89 (11)	43 (18)	96 (13)	30 (17)	40 (9)	32 (14)	63 (7)	140 (11)	305 (9)	
**“I would enter voluntary quarantine/isolation/social distancing for social/self-responsibility.”**	N=909	N=567	N=82	N=745	N=247	N=762	N=217	N=495	N=202	N=832	N=1,657	N=3,401	0.370
Agree	842 (92)	512 (89)	65 (80)	609 (83)	180 (73)	635 (83)	165 (75)	401 (80)	151 (72)	687 (82)	1,403 (81)	2,844 (84)	
Neither agree nor disagree	55 (7)	45 (10)	3 (4)	45 (6)	17 (6)	33 (4)	24 (11)	35 (7)	24 (15)	67 (9)	123 (8)	225 (7)	
Disagree	12 (1)	10 (2)	14 (16)	91 (11)	50 (21)	94 (13)	28 (14)	59 (13)	27 (13)	78 (9)	131 (11)	332 (10)	
**How much do you agree with quarantine/isolation/social distancing? “It is a necessary strategy to help control COVID-19.”**	N=909	N=567	N=82	N=745	N=247	N=762	N=217	N=495	N=202	N=832	N=1,657	N=3,401	0.304
Agree	858 (95)	525 (91)	72 (88)	667 (90)	201 (80)	652 (85)	179 (78)	429 (84)	145 (768	701 (85)	1,455 (84)	2,974 (87)	
Neither agree nor disagree	34 (4)	31 (7)	0 (0)	12 (2)	8 (4)	19 (3)	6 (5)	22 (5)	23 (14)	53 (6)	71 (5)	137 (5)	
Disagree	17 (2)	11 (2)	10 (12)	66 (8)	38 (17)	91 (12)	32 (17)	44 (10)	34 (19)	78 (9)	131 (11)	290 (9)	

For peer review only - http://bmjopen.bmj.com/site/about/guidelines.xhtml

Suppl. Table 26 Breakdown of behavioural changes and acceptance of government public health measures by age group

Values in cells are n (weighted %) of respondents who replied 'yes'.

Variable and categories	Thailand			Malaysia			UK			Italy			Slovenia			Total			
**Age group**	**18-34**	**35-64**	**65+**	**18-34**	**35-64**	**65+**	**18-34**	**35-64**	**65+**	**18-34**	**35-64**	**65+**	**18-34**	**35-64**	**65+**	**18-34**	**35-64**	**65+**	**P-value (for total)**
	N=223	N=1,152	N=101	N=350	N=442	N=35	N=140	N=616	N=253	N=272	N=383	N=57	N=308	N=676	N=50	N=1,293	N=3,269	N=496	
**Did you change your social behaviour before the implementation of government restrictions?**	202 (92)	1,079 (94)	93 (93)	233 (63)	287 (71)	18 (37)	104 (71)	448 (69)	160 (61)	124 (44)	202 (44)	30 (57)	178 (54)	386 (53)	20 (25)	841 (70)	2,402 (70)	321 (57)	0.004
**If you answered 'yes' to the previous question: how did you change your social behaviour?**																			
No physical contact with anyone	(N=202) 180 (91)	(N=1,079) 1,037 (96)	(N=93) 85 (90)	(N=225) 156 (84)	(N=265) 193 (80)	(N=16) 13 (81)	(N=99) 35 (43)	(N=412) 200 (51)	(N=146) 90 (61)	(N=120) 79 (72)	(N=196) 143 (74)	(N=26) 21 (75)	(N=176) 151 (87)	(N=380) 345 (94)	(N=20) 20 (100)	(N=822) 601 (78)	(N=2,332) 1,918 (84)	(N=301) 229 (82)	0.204
No physical contact only with elderly and those with serious underlying medical conditions	(N=202) 168 (88)	(N=1,079) 956 (90)	(N=93) 76 (83)	(N=218) 127 (65)	(N=261) 158 (61)	(N=15) 7 (73)	(N=98) 60 (60)	(N=416) 271 (65)	(N=130) 62 (46)	(N=120) 100 (89)	(N=187) 150 (80)	(N=25) 22 (69)	(N=174) 163 (90)	(N=374) 340 (92)	(N=18) 13 (87)	(N=812) 618 (78)	(N=2,317) 1,875 (81)	(N=281) 180 (73)	0.152
Going out only for essential needs	(N=202) 186 (94)	(N=1,079) 1,022 (95)	(N=93) 83 (89)	(N=230) 212 (98)	(N=278) 262 (94)	(N=17) 15 (82)	(N=102) 79 (76)	(N=427) 362 (86)	(N=152) 130 (86)	(N=121) 79 (68)	(N=198) 159 (79)	(N=27) 25 (99)	(N=174) 102 (55)	(N=370) 266 (75)	(N=18) 13 (87)	(N=829) 658 (85)	(N=2,352) 2,071 (88)	(N=307) 266 (89)	0.153
Moving home to stay with parents/relatives	(N=202) 88 (59)	(N=1,079) 556 (56)	(N=93) 33 (34)	(N=219) 65 (38)	(N=256) 32 (16)	(N=14) 2 (22)	(N=98) 21 (21)	(N=398) 8 (2)	(N=131) 1 (2)	(N=120) 16 (11)	(N=184) 11 (7)	(N=22) 0 (0)	(N=172) 16 (8)	(N=363) 17 (4)	(N=17) 0 (0)	(N=811) 206 (37)	(N=2,280) 624 (29)	(N=277) 36 (17)	<0.001
Use of personal protection equipment (e.g. masks and gloves)	(N=202) 198 (98)	(N=1,079) 1,050 (97)	(N=93) 86 (90)	(N=230) 212 (93)	(N=279) 262 (99)	(N=18) 14 (80)	(N=100) 23 (20)	(N=417) 157 (40)	(N=134) 45 (35)	(N=121) 48 (39)	(N=191) 100 (54)	(N=27) 17 (69)	(N=174) 88 (52)	(N=371) 260 (68)	(N=19) 18 (97)	(N=827) 569 (72)	(N=2,337) 1,829 (79)	(N=291) 180 (74)	0.067
Use of sanitizer products and alcohol	(N=202) 197 (96)	(N=1,079) 1,037 (96)	(N=93) 87 (91)	(N=230) 218 (94)	(N=281) 271 (99)	(N=18) 15 (81)	(N=102) 88 (84)	(N=436) 352 (82)	(N=147) 119 (84)	(N=122) 103 (84)	(N=199) 177 (90)	(N=29) 27 (99)	(N=174) 157 (92)	(N=377) 346 (94)	(N=18) 18 (100)	(N=830) 763 (92)	(N=2,372) 2,183 (93)	(N=305) 266 (91)	0.613
**"I would comply with government enforced quarantine/ isolation/social distancing."**	N=223	N=1,152	N=101	N=350	N=442	N=35	N=140	N=616	N=253	N=272	N=383	N=57	N=308	N=676	N=50	N=1,293	N=3,269	N=496	0.003
Agree	189 (90)	1,058 (92)	97 (96)	307 (82)	371 (88)	30 (91)	120 (85)	493 (78)	209 (80)	247 (88)	311 (77)	48 (72)	272 (85)	559 (75)	40 (65)	1,135 (86)	2,792 (83)	424 (80)	
Neither agree nor disagree	28 (8)	63 (5)	1 (1)	7 (1)	11 (1)	0 (0)	3 (1)	33 (6)	12 (5)	7 (2)	24 (5)	5 (14)	16 (7)	44 (8)	8 (34)	61 (4)	175 (5)	26 (13)	
Disagree	6 (2)	31 (3)	3 (3)	36 (18)	60 (11)	5 (9)	17 (14)	90 (17)	32 (14)	18 (10)	48 (17)	4 (14)	20 (8)	73 (17)	2 (1)	97 (10)	302 (12)	46 (8)	

**"I would enter voluntary quarantine/isolation/social distancing for social/self-responsibility."**	N=223	N=1,152	N=101	N=350	N=442	N=35	N=140	N=616	N=253	N=272	N=383	N=57	N=308	N=676	N=50	N=1,293	N=3,269	N=496	0.327
Agree	188 (86)	1,068 (93)	98 (96)	294 (79)	353 (86)	27 (68)	114 (79)	497 (78)	204 (78)	211 (70)	306 (75)	49 (84)	247 (80)	550 (75)	41 (74)	1,054 (80)	2,774 (83)	419 (82)	
Neither agree nor disagree	33 (13)	64 (5)	3 (4)	23 (7)	23 (1)	2 (9)	6 (4)	30 (5)	14 (7)	28 (15)	28 (8)	3 (10)	28 (9)	57 (11)	6 (20)	118 (9)	202 (6)	28 (10)	
Disagree	2 (1)	20 (2)	0 (0)	33 (15)	66 (13)	6 (24)	20 (17)	89 (17)	35 (15)	33 (16)	49 (17)	5 (6)	33 (11)	69 (13)	3 (7)	121 (11)	293 (11)	49 (8)	
**How much do you agree with quarantine/isolation/social distancing? "It is a necessary strategy to help control COVID-19."**	N=223	N=1,152	N=101	N=350	N=442	N=35	N=140	N=616	N=253	N=272	N=383	N=57	N=308	N=676	N=50	N=1,293	N=3,269	N=496	0.271
Agree	203 (93)	1,083 (94)	97 (96)	313 (85)	393 (89)	33 (100)	120 (83)	521 (83)	212 (82)	243 (86)	315 (78)	50 (79)	254 (79)	549 (76)	43 (67)	1,133 (86)	2,861 (85)	435 (82)	
Neither agree nor disagree	18 (7)	45 (4)	2 (2)	5 (0)	6 (0)	1 (0)	3 (3)	16 (3)	8 (4)	10 (4)	14 (3)	4 (11)	28 (12)	45 (7)	3 (18)	64 (5)	126 (4)	18 (8)	
Disagree	2 (0)	24 (2)	2 (2)	32 (15)	43 (11)	1 (0)	17 (14)	79 (15)	33 (14)	19 (10)	54 (19)	3 (10)	26 (10)	82 (17)	4 (15)	96 (9)	282 (11)	43 (10)	

For peer review only

Suppl. Table 27 Breakdown of behavioural changes and acceptance of government public health measures by self-reported level of understanding of COVID-19

H = high/very high/expert level; S = some; N = a little/none at all. Values in cells are n (weighted %) of respondents who replied 'yes'.

**Variable and categories**	**Thailand**			**Malaysia**			**UK**			**Italy**			**Slovenia**			**Total**			
**Self-reported level of understanding of COVID-19**	**H**	**S**	**N**	**H**	**S**	**N**	**H**	**S**	**N**	**H**	**S**	**N**	**H**	**S**	**N**	**H**	**S**	**N**	**P-value (for total)**
	N=965	N=459	N=52	N=435	N=359	N=33	N=647	N=336	N=26	N=368	N=324	N=20	N=713	N=279	N=42	N=3,128	N=1,757	N=173	
**Did you change your social behaviour before the implementation of government restrictions?**	898 (94)	430 (92)	46 (91)	285 (64)	232 (66)	21 (58)	468 (69)	232 (66)	12 (68)	200 (52)	146 (43)	10 (60)	429 (52)	137 (37)	18 (46)	2,280 (70)	1,177 (64)	107 (65)	0.091
**If you answered 'yes' to the previous question: how did you change your social behaviour?**																			
No physical contact with anyone	(N=898) 849 (94)	(N=430) 411 (95)	(N=46) 42 (9187)	(N=272) 204 (90)	(N=214) 143 (73)	(N=20) 15 (69)	(N=428) 221 (53)	(N=217) 99 (47)	(N=12) 5 (52)	(N=194) 137 (78)	(N=138) 99 (67)	(N=10) 7 (88)	(N=423) 380 (95)	(N=135) 119 (87)	(N=18) 17 (96)	(N=2,215) 1,791 (85)	(N=1,134) 871 (77)	(N=106) 86 (78)	0.033
No physical contact only with elderly and those with serious underlying medical conditions	(N=898) 765 (87)	(N=430) 394 (92)	(N=46) 41 (87)	(N=266) 162 (63)	(N=209) 119 (60)	(N=19) 11 (74)	(N=417) 261 (61)	(N=215) 128 (59)	(N=12) 4 (49)	(N=192) 163 (85)	(N=130) 101 (67)	(N=10) 8 (94)	(N=418) 379 (91)	(N=131) 122 (92)	(N=17) 15 (95)	(N=2,191) 1,730 (80)	(N=1,115) 864 (77)	(N=104) 79 (79)	0.744
Going out only for essential needs	(N=898) 844 (93)	(N=430) 405 (95)	(N=46) 42 (87)	(N=280) 266 (99)	(N=225) 205 (89)	(N=20) 18 (99)	(N=444) 381 (86)	(N=225) 182 (80)	(N=12) 8 (66)	(N=196) 145 (80)	(N=140) 109 (83)	(N=10) 9 (95)	(N=415) 283 (72)	(N=129) 87 (73)	(N=18) 11 (60)	(N=2,233) 1,919 (88)	(N=1,149) 988 (87)	(N=106) 88 (84)	0.711
Moving home to stay with parents/relatives	(N=898) 345 (45)	(N=430) 298 (67)	(N=46) 34 (73)	(N=261) 45 (24)	(N=209) 48 (25)	(N=19) 6 (40)	(N=404) 17 (5)	(N=212) 12 (10)	(N=11) 1 (24)	(N=189) 17 (6)	(N=127) 9 (7)	(N=10) 1 (10)	(N=405) 19 (3)	(N=129) 14 (9)	(N=18) 0 (0)	(N=2,157) 443 (25)	(N=1,107) 381 (36)	(N=104) 42 (42)	<0.001
Use of personal protection equipment (e.g. masks and gloves)	(N=898) 874 (97)	(N=430) 418 (96)	(N=46) 42 (81)	(N=280) 266 (99)	(N=227) 203 (90)	(N=20) 19 (99)	(N=421) 153 (38)	(N=218) 68 (28)	(N=12) 4 (17)	(N=194) 90 (46)	(N=135) 69 (66)	(N=10) 6 (66)	(N=416) 289 (71)	(N=130) 71 (59)	(N=18) 6 (38)	(N=2,209) 1,672 (78)	(N=1,140) 829 (74)	(N=106) 77 (69)	0.172
Use of sanitizer products and alcohol	(N=898) 863 (96)	(N=430) 416 (95)	(N=46) 42 (81)	(N=281) 270 (99)	(N=228) 215 (91)	(N=20) 19 (100)	(N=447) 374 (85)	(N=226) 179 (85)	(N=12) 6 (30)	(N=198) 170 (90)	(N=142) 129 (93)	(N=10) 8 (94)	(N=418) 385 (95)	(N=133) 125 (95)	(N=18) 11 (70)	(N=2,242) 2,062 (94)	(N=1,159) 1,064 (92)	(N=106) 86 (78)	<0.001

**"I would comply with government enforced quarantine/ isolation/social distancing."**	N=965	N=459	N=52	N=435	N=359	N=33	N=647	N=336	N=26	N=368	N=324	N=20	N=713	N=279	N=42	N=3,128	N=1,757	N=173	0.370
Agree	903 (95)	402 (88)	39 (81)	378 (93)	305 (79)	25 (76)	511 (79)	291 (83)	20 (87)	303 (76)	284 (79)	19 (97)	607 (75)	232 (75)	32 (70)	2,702 (85)	1,514 (82)	135 (80)	
Neither agree nor disagree	39 (3)	44 (9)	9 (10)	5 (0)	9 (1)	4 (1)	29 (3)	18 (6)	1 (2)	17 (4)	18 (11)	1 (3)	45 (16)	19 (10)	4 (7)	135 (6)	108 (7)	19 (4)	
Disagree	23 (2)	13 (3)	4 (9)	52 (7)	45 (20)	4 (23)	107 (18)	27 (12)	5 (11)	48 (21)	22 (10)	0 (0)	61 (9)	28 (15)	6 (24)	291 (10)	135 (11)	19 (16)	
**"I would enter voluntary quarantine/isolation/social distancing for social/self-responsibility."**	N=965	N=459	N=52	N=435	N=359	N=33	N=647	N=336	N=26	N=368	N=324	N=20	N=713	N=279	N=42	N=3,128	N=1,757	N=173	0.091
Agree	909 (95)	401 (85)	44 (90)	357 (86)	294 (76)	23 (75)	516 (78)	284 (80)	15 (60)	293 (78)	258 (74)	15 (91)	587 (78)	219 (74)	32 (69)	2,662 (84)	1,456 (79)	129 (77)	
Neither agree nor disagree	41 (4)	51 (13)	8 (10)	21 (1)	21 (10)	6 (1)	29 (5)	18 (5)	3 (8)	27 (8)	30 (12)	2 (6)	58 (14)	26 (9)	7 (23)	176 (6)	146 (10)	26 (8)	
Disagree	15 (1)	7 (1)	0 (0)	57 (13)	44 (14)	4 (23)	102 (17)	34 (15)	8 (32)	48 (15)	36 (13)	3 (4)	68 (9)	34 (17)	3 (7)	290 (9)	155 (11)	18 (15)	
**How much do you agree with quarantine/isolation/social distancing? "It is a necessary strategy to help control COVID-19."**	N=965	N=459	N=52	N=435	N=359	N=33	N=647	N=336	N=26	N=368	N=324	N=20	N=713	N=279	N=42	N=3,128	N=1,757	N=173	0.688
Agree	920 (96)	418 (91)	45 (90)	392 (91)	319 (85)	28 (86)	540 (82)	293 (83)	20 (85)	304 (77)	285 (82)	19 (82)	589 (73)	226 (78)	31 (72)	2,745 (85)	1,541 (85)	143 (84)	
Neither agree nor disagree	26 (2)	33 (8)	6 (8)	5 (0)	5 (0)	2 (1)	16 (3)	10 (3)	1 (2)	10 (2)	18 (9)	0 (0)	45 (12)	27 (9)	4 (7)	102 (4)	93 (6)	13 (4)	
Disagree	19 (1)	8 (2)	1 (2)	38 (9)	35 (15)	3 (13)	91 (15)	33 (13)	5 (13)	54 (21)	21 (9)	1 (18)	79 (16)	26 (13)	7 (21)	281 (11)	123 (10)	17 (12)	

For peer review only - http://bmjopen.bmj.com/site/about/guidelines.xhtml

Suppl. Table 28 Breakdown of self-reported level of understanding of COVID-19 by country

Values in cells are n (weighted %) of respondents who replied 'yes'.

Variable and categories	Thailand	Malaysia	UK	Italy	Slovenia	Total	P-value
	N=1,476	N=827	N=1,009	N=712	N=1,034	N=5,058	<0.001
**High/very high/expert level understanding**	965 (63)	435 (51)	647 (59)	368 (47)	713 (66)	3,128 (59)	
**Some understanding**	459 (33)	359 (38)	336 (38)	324 (50)	279 (30)	1,757 (36)	
**A little/none at all**	52 (4)	33 (11)	26 (4)	20 (3)	42 (4)	173 (5)	

For peer review only

Suppl. Table 29 Breakdown of self-reported level of understanding of COVID-19 by demographic characteristics

H = high/very high/expert level; S = some; N = a little/none at all. Values in cells are n (weighted %) of respondents who replied ‘yes’.

**Variable and categories**	**Thailand**			**Malaysia**			**UK**			**Italy**			**Slovenia**			**Total**			
**Self-reported understanding of COVID-19**	**H**	**S**	**N**	**H**	**S**	**N**	**H**	**S**	**N**	**H**	**S**	**N**	**H**	**S**	**N**	**H**	**S**	**N**	**P-value (for total)**
**Gender**																			0.058
Male	458 (65)	224 (31)	22 (4)	153 (55)	130 (30)	15 (15)	280 (61)	134 (35)	12 (4)	130 (51)	87 (46)	5 (3)	269 (64)	84 (31)	13 (5)	1,290 (60)	659 (34)	67 (6)	
Female	504 (61)	232 (35)	30 (4)	280 (47)	228 (46)	17 (7)	358 (56)	200 (40)	14 (3)	238 (44)	237 (53)	15 (3)	439 (68)	194 (29)	29 (3)	1,819 (57)	1,091 (39)	105 (4)	
Other/prefer not to say	3 (50)	3 (50)	0 (0)	2 (50)	1 (25)	1 (25)	9 (82)	2 (18)	0 (0)				5 (83)	1 (17)	0 (0)	19 (70)	7 (26)	1 (4)	
**Age group**																			0.033
18-34	143 (62)	69 (34)	11 (4)	170 (48)	167 (48)	13 (9)	74 (44)	58 (48)	8 (8)	119 (39)	143 (57)	10 (5)	186 (59)	106 (35)	16 (6)	692 (52)	543 (41)	58 (6)	
35-64	746 (62)	371 (35)	35 (3)	244 (54)	179 (32)	19 (14)	411 (67)	193 (32)	12 (2)	220 (54)	153 (42)	10 (4)	492 (69)	158 (27)	26 (5)	2,113 (62)	1,054 (33)	102 (5)	
65+	76 (68)	19 (25)	6 (7)	21 (52)	13 (42)	1 (6)	162 (59)	85 (39)	6 (2)	29 (42)	28 (58)	0 (0)	35 (68)	15 (32)	0 (0)	323 (60)	160 (38)	13 (3)	
**Education level**																			<0.001
Primary or lower/secondary	537 (60)	341 (36)	31 (4)	42 (51)	30 (36)	10 (13)	140 (52)	101 (44)	6 (4)	92 (43)	114 (53)	11 (4)	124 (63)	67 (33)	11 (4)	935 (56)	653 (39)	69 (6)	
Tertiary	428 (74)	118 (22)	21 (4)	393 (51)	329 (46)	23 (3)	507 (64)	235 (32)	20 (3)	276 (58)	210 (41)	9 (2)	589 (71)	212 (26)	31 (3)	2,193 (66)	1,104 (31)	104 (3)	
**Healthcare worker status**																			0.001
Healthcare worker	172 (72)	59 (26)	8 (3)	128 (49)	79 (50)	6 (1)	90 (76)	24 (21)	4 (3)	45 (67)	18 (29)	1 (4)	291 (78)	44 (21)	6 (1)	726 (70)	224 (28)	25 (2)	
Non-healthcare worker	793 (61)	400 (33)	44 (4)	307 (52)	280 (35)	27 (13)	557 (57)	312 (39)	22 (4)	323 (46)	306 (50)	19 (3)	422 (63)	235 (32)	36 (5)	2,402 (57)	1,533 (38)	148 (5)	

For peer review only - http://bmjopen.bmj.com/site/about/guidelines.xhtml

Suppl. Table 30 Breakdown of self-reported understanding of public health measures by self-reported level of understanding of COVID-19

(H = high/very high/expert level; S = some; N = a little/none at all). Values in cells are n (weighted %) of respondents who replied ‘yes’.

**Variable and categories**	**Thailand**			**Malaysia**			**UK**			**Italy**			**Slovenia**			**Total**			
**Self-reported level of understanding of COVID-19**	**H**	**S**	**N**	**H**	**S**	**N**	**H**	**S**	**N**	**H**	**S**	**N**	**H**	**S**	**N**	**H**	**S**	**N**	**P-value**
**How would you rate your level of understanding of the current quarantine/isolation/social distancing requirements for COVID-19?**	N=965	N=459	N=52	N=435	N=359	N=33	N=647	N=336	N=26	N=368	N=324	N=20	N=713	N=279	N=42	N=3,128	N=1,757	N=173	<0.001
H	855 (89)	116 (23)	19 (24)	399 (89)	193 (52)	9 (21)	532 (81)	182 (57)	8 (21)	338 (93)	213 (71)	7 (36)	652 (89)	212 (59)	24 (46)	2,776 (88)	916 (50)	67 (27)	
S	102 (10)	323 (71)	11 (12)	31 (7)	157 (39)	15 (52)	98 (15)	129 (35)	11 (46)	22 (5)	106 (28)	10 (38)	50 (10)	55 (32)	12 (44)	303 (10)	770 (43)	59 (39)	
N	8 (1)	20 (6)	22 (64)	5 (4)	9 (9)	9 (27)	17 (4)	25 (8)	7 (33)	8 (2)	5 (1)	3 (26)	11 (1)	12 (9)	6 (10)	49 (2)	71 (6)	47 (34)	

For peer review only

Suppl. Table 31 What were the three most common ways people received communication on COVID-19, and what are the three most preferred ways to receive COVID-19 communications? Breakdown by country

Values in cells are n (weighted %) of respondents who replied 'yes'.

**Variable and categories**	**Thailand**	**Malaysia**	**UK**	**Italy**	**Slovenia**	**Total**	**P-value**
	N=1,476	N=827	N=1,009	N=712	N=1,034	N=5,058	
**How do/did you receive information about COVID-19?**							
Face-to-face (e.g. doctors or health workers)	1,096 (78)	275 (19)	155 (15)	276 (32)	413 (34)	2,215 (40)	<0.001
Traditional media (TV, radio, newspapers)	1,407 (95)	795 (93)	940 (93)	650 (85)	994 (95)	4,786 (93)	0.012
Print materials (leaflets, brochures)	803 (55)	256 (32)	403 (36)	119 (23)	479 (43)	2,060 (40)	<0.001
Online (websites, email)	1,101 (69)	779 (90)	918 (89)	651 (88)	964 (87)	4,413 (83)	<0.001
Social media and messenger apps	1,279 (83)	786 (95)	773 (77)	528 (75)	731 (66)	4,097 (79)	<0.001
Government/institution's web page	1,134 (74)	682 (75)	698 (70)	580 (79)	784 (60)	3,878 (71)	<0.001
WHO web page	367 (20)	550 (56)	380 (36)	334 (39)	397 (30)	2,028 (34)	<0.001
**How would you prefer to receive information about COVID-19?**							
Face-to-face (e.g doctors or health workers)	1,200 (83)	417 (44)	361 (36)	584 (77)	577 (55)	3,139 (61)	<0.001
Traditional media (TV, radio, newspapers)	1,347 (90)	759 (91)	648 (64)	467 (62)	806 (76)	4,027 (78)	<0.001
Print materials	893 (63)	340 (40)	418 (41)	149 (29)	481 (52)	2,281 (48)	<0.001
Online (websites, email)	1,105 (71)	742 (88)	812 (75)	473 (71)	856 (79)	3,988 (76)	<0.001
Social media and messenger apps	1,245 (82)	659 (85)	330 (31)	292 (50)	470 (50)	2,996 (61)	<0.001
Government/institution's web page	1,181 (77)	731 (86)	741 (74)	605 (77)	845 (71)	4,103 (77)	0.009
WHO web page	586 (36)	703 (82)	609 (58)	531 (64)	678 (55)	3,107 (56)	<0.001

For peer review only

Suppl. Table 32 What were the three most common ways people received communications on COVID-19, and what are the three most preferred ways to receive COVID-19 communications? Breakdown by country and gender

M = male; F = female; O = other/prefer not to say. Values in cells are n (weighted %) of respondents who replied 'yes'.

**Variable and categories**	**Thailand**			**Malaysia**			**UK**			**Italy**			**Slovenia**			**Total**			
**Gender**	**M**	**F**	**O**	**M**	**F**	**O**	**M**	**F**	**O**	**M**	**F**	**O**	**M**	**F**	**O**	**M**	**F**	**O**	**P-value (for total M vs F)**
	N=704	N=766	N=6	N=298	N=525	N=4	N=426	N=572	N=11	N=222	N=490	N=0	N=366	N=662	N=6	N=2,016	N=3,015	N=27	
**How do/did you receive information about COVID-19?**																			
Face-to-face	563 (81)	529 (75)	4 (67)	93 (17)	180 (21)	2 (50)	68 (16)	84 (14)	3 (27)	82 (29)	194 (34)		126 (31)	285 (37)	2 (33)	932 (40)	1,272 (41)	11 (41)	0.591
Traditional media (TV, radio, newspapers)	669 (94)	732 (96)	6 (100)	284 (92)	507 (93)	4 (100)	390 (92)	539 (95)	11 (100)	199 (82)	451 (88)		353 (98)	635 (93)	6 (100)	1,895 (92)	2,864 (94)	27 (100)	0.468
Print materials (leaflets, brochures)	398 (54)	402 (56)	3 (50)	94 (37)	162 (26)	0 (0)	171 (37)	227 (36)	5 (45)	31 (27)	88 (20)		168 (44)	307 (41)	4 (67)	862 (42)	1,186 (39)	12 (44)	0.265
Online (websites, email)	509 (69)	586 (69)	6 (100)	281 (92)	495 (89)	3 (75)	379 (87)	528 (91)	11 (100)	201 (85)	450 (90)		336 (84)	622 (90)	6 (100)	1,706 (82)	2,681 (84)	26 (96)	0.332
Social media and messenger apps	595 (84)	678 (82)	6 (100)	281 (96)	502 (94)	3 (75)	312 (74)	450 (79)	11 (100)	154 (70)	374 (80)		256 (66)	470 (67)	5 (83)	1,598 (78)	2,474 (80)	25 (93)	0.589
Government/institution's web page	540 (73)	589 (74)	5 (83)	246 (80)	432 (69)	4 (100)	282 (69)	409 (71)	7 (64)	170 (74)	410 (83)		260 (59)	518 (61)	6 (100)	1,498 (71)	2,358 (71)	22 (81)	0.881
WHO web page	150 (18)	214 (22)	3 (50)	173 (52)	374 (60)	3 (75)	136 (34)	239 (39)	5 (45)	81 (27)	253 (50)		108 (26)	286 (33)	3 (50)	648 (30)	1,366 (38)	14 (52)	0.003
**How would you prefer to receive information about COVID-19?**																			
Face-to-face	594 (85)	603 (82)	3 (50)	146 (39)	270 (50)	1 (25)	163 (36)	195 (37)	3 (27)	171 (75)	413 (79)		182 (53)	389 (57)	6 (100)	1,256 (59)	1,870 (63)	13 (48)	0.209
Traditional media (TV, radio, newspapers)	644 (89)	697 (91)	6 (100)	267 (91)	488 (92)	4 (100)	278 (66)	365 (63)	5 (45)	134 (57)	333 (67)		274 (76)	530 (77)	2 (33)	1,597 (77)	2,413 (79)	17 (63)	0.395
Print materials	446 (65)	442 (61)	5 (83)	115 (39)	223 (41)	2 (50)	177 (41)	237 (41)	4 (36)	46 (33)	103 (25)		165 (53)	314 (51)	2 (33)	949 (49)	1,319 (47)	13 (48)	0.408
Online (websites, email)	516 (70)	583 (71)	6 (100)	269 (92)	469 (83)	4 (100)	334 (71)	470 (78)	8 (73)	151 (72)	322 (70)		290 (74)	561 (84)	5 (83)	1,560 (75)	2,405 (77)	23 (85)	0.403
Social media and messenger apps	589 (84)	650 (80)	6 (100)	239 (85)	416 (87)	4 (100)	134 (29)	195 (34)	1 (9)	88 (52)	204 (48)		161 (43)	307 (57)	2 (33)	1,211 (60)	1,772 (63)	13 (48)	0.364
Government/institution's web page	575 (78)	601 (75)	5 (83)	270 (93)	457 (79)	4 (100)	293 (69)	440 (78)	8 (73)	181 (73)	424 (82)		278 (64)	561 (77)	6 (100)	1,597 (75)	2,483 (78)	23 (85)	0.335
WHO web page	248 (36)	334 (36)	4 (67)	242 (80)	457 (83)	4 (100)	234 (54)	370 (62)	5 (45)	143 (54)	388 (74)		209 (49)	466 (60)	3 (50)	1,076 (52)	2,015 (59)	16 (59)	0.020

Suppl. Table 33 What were the three most common ways people received communications on COVID-19, and what are the three most preferred ways to receive COVID-19 communications? Breakdown by country and age group

Values in cells are n (weighted %) of respondents who replied 'yes'.

Variable and categories	Thailand			Malaysia			UK			Italy			Slovenia			Total			
**Age group**	**18-34**	**35-64**	**65+**	**18-34**	**35-64**	**65+**	**18-34**	**35-64**	**65+**	**18-34**	**35-64**	**65+**	**18-34**	**35-64**	**65+**	**18-34**	**35-64**	**65+**	**P-value (for total)**
	N=223	N=1,152	N=101	N=350	N=442	N=35	N=140	N=616	N=253	N=272	N=383	N=57	N=308	N=676	N=50	N=1,293	N=3,269	N=496	
**How do/did you receive information about COVID-19?**																			
Face-to-face	125 (68)	892 (82)	79 (82)	141 (20)	124 (16)	10 (23)	25 (17)	107 (17)	23 (8)	112 (37)	152 (34)	12 (23)	111 (32)	282 (30)	20 (48)	514 (37)	1,557 (42)	144 (40)	0.424
Traditional media (TV, radio, newspapers)	210 (94)	1,099 (95)	98 (96)	337 (89)	424 (95)	34 (100)	130 (93)	567 (92)	243 (97)	247 (92)	352 (90)	51 (70)	299 (98)	647 (96)	48 (91)	1,223 (93)	3,089 (94)	474 (90)	0.336
Print materials (leaflets, brochures)	107 (54)	652 (59)	44 (44)	104 (31)	146 (35)	6 (20)	34 (22)	258 (40)	111 (43)	34 (12)	71 (19)	14 (41)	140 (45)	319 (46)	20 (31)	419 (37)	1,446 (43)	195 (38)	0.106
Online (websites, email)	199 (84)	853 (71)	49 (35)	328 (86)	418 (94)	33 (91)	129 (89)	575 (92)	214 (82)	242 (90)	358 (89)	51 (82)	289 (93)	632 (91)	43 (74)	1,187 (87)	2,836 (85)	390 (69)	<0.001
Social media and messenger apps	206 (91)	1,008 (86)	65 (55)	329 (93)	424 (98)	33 (91)	104 (76)	485 (78)	184 (74)	214 (79)	274 (73)	40 (77)	243 (80)	462 (70)	26 (42)	1,096 (86)	2,653 (81)	348 (63)	<0.001
Government/institution's web page	166 (73)	902 (78)	66 (61)	298 (71)	360 (81)	24 (61)	108 (77)	459 (74)	131 (53)	219 (73)	318 (81)	43 (78)	226 (68)	528 (71)	30 (29)	1,017 (72)	2,567 (77)	294 (54)	<0.001
WHO web page	100 (31)	256 (19)	11 (6)	260 (62	274 (53)	16 (39)	60 (45)	271 (40)	49 (18)	129 (39)	176 (38)	29 (42)	127 (39)	255 (30)	15 (19)	676 (44)	1,232 (33)	120 (22)	<0.001
**How would you prefer to receive information about COVID-19?**																			
Face-to-face	152 (77)	965 (87)	83 (84)	198 (53)	203 (34)	16 (53)	48 (33)	218 (37)	95 (39)	230 (78)	313 (80)	41 (71)	187 (57)	365 (53)	25 (59)	815 (59)	2,064 (61)	260 (62)	0.785
Traditional media (TV, radio, newspapers)	194 (85)	1,056 (91)	97 (93)	327 (90)	402 (91)	30 (99)	89 (65)	396 (64)	163 (64)	179 (60)	247 (58)	41 (72)	228 (73)	534 (75)	44 (83)	1,017 (78)	2,635 (78)	375 (80)	0.712
Print materials	118 (64)	720 (65)	55 (54)	143 (41)	179 (37)	18 (45)	40 (27)	256 (44)	122 (52)	43 (15)	88 (24)	18 (50)	149 (50)	308 (48)	24 (63)	493 (44)	1,551 (48)	237 (54)	0.073
Online (websites, email)	187 (83)	867 (73)	51 (41)	312 (87)	399 (91)	31 (77)	98 (59)	522 (84)	192 (74)	180 (74)	253 (68)	40 (75)	250 (79)	567 (83)	39 (71)	1,027 (78)	2,608 (79)	353 (66)	<0.001
Social media and messenger apps	196 (91)	986 (85)	63 (55)	285 (88)	349 (86)	25 (75)	34 (21)	219 (37)	77 (31)	105 (38)	156 (48)	31 (65)	134 (48)	317 (51)	19 (49)	754 (64)	2,027 (64)	215 (52)	0.005
Government/institution's web page	177 (79)	936 (80)	68 (60)	323 (93)	381 (81)	27 (82)	108 (71)	468 (77)	165 (71)	235 (83)	325 (82)	45 (65)	252 (75)	557 (76)	36 (56)	1,095 (81)	2,667 (79)	341 (64)	<0.001
WHO web page	145 (55)	415 (31)	26 (20)	320 (92)	357 (72)	26 (77)	98 (65)	387 (60)	124 (46)	226 (79)	266 (64)	39 (53)	231 (73)	427 (59)	20 (26)	1,020 (72)	1,852 (53)	235 (39)	<0.001

For peer review only - http://bmjopen.bmj.com/site/about/guidelines.xhtml

Suppl. Table 34 What were the three most common ways people received communications on COVID-19, and what are the three most preferred ways to receive COVID-19 communications? Breakdown by country and education level

P/S = primary or lower/secondary education; T = tertiary education. Values in cells are n (weighted %) of respondents who replied ‘yes’.

**Variable and categories**	**Thailand**		**Malaysia**		**UK**		**Italy**		**Slovenia**		**Total**		
**Education level**	**P/S**	**T**	**P/S**	**T**	**P/S**	**T**	**P/S**	**T**	**P/S**	**T**	**P/S**	**T**	**P-value (for total)**
	N=909	N=567	N=82	N=745	N=247	N=762	N=217	N=495	N=202	N=832	N=1,657	N=3,401	
**How do/did you receive information about COVID-19?**													
Face-to-face	781 (83)	315 (55)	13 (14)	262 (37)	32 (14)	123 (16)	72 (28)	204 (39)	48 (29)	365 (43)	946 (43)	1,269 (35)	<0.001
Traditional media (TV, radio, newspapers)	865 (95)	542 (95)	76 (92)	719 (97)	234 (95)	706 (92)	192 (82)	458 (93)	196 (95)	798 (96)	1,563 (92)	3,223 (94)	0.155
Print materials (leaflets, brochures)	547 (57)	256 (45)	26 (32)	230 (31)	90 (34)	313 (38)	39 (26)	80 (16)	91 (40)	388 (47)	793 (42)	1,267 (38)	0.062
Online (websites, email)	605 (65)	496 (87)	74 (89)	705 (95)	212 (85)	706 (93)	190 (85)	461 (93)	179 (83)	785 (94)	1,260 (79)	3,153 (92)	<0.001
Social media and messenger apps	757 (81)	522 (91)	78 (95)	708 (94)	196 (79)	577 (75)	173 (78)	355 (70)	150 (65)	581 (68)	1,354 (80)	2,743 (77)	0.146
Government/institution’s web page	689 (73)	445 (78)	59 (73)	623 (85)	171 (70)	527 (71)	166 (77)	414 (81)	123 (49)	661 (78)	1,208 (69)	2,670 (77)	<0.001
WHO web page	139 (15)	228 (42)	44 (53)	506 (67)	68 (30)	312 (42)	84 (35)	250 (49)	59 (24)	338 (39)	394 (29)	1,634 (44)	<0.001
**How would you prefer to receive information about COVID-19?**													
Face-to-face	806 (87)	394 (68)	36 (42)	381 (53)	104 (39)	257 (34)	170 (75)	414 (81)	111 (56)	466 (54)	1,227 (65)	1,912 (53)	<0.001
Traditional media (TV, radio, newspapers)	830 (90)	517 (90)	75 (91)	684 (92)	149 (63)	499 (66)	133 (60)	334 (68)	145 (74)	661 (80)	1,332 (79)	2,695 (76)	0.100
Print materials	608 (66)	285 (49)	35 (40)	305 (40)	126 (47)	292 (37)	48 (32)	101 (21)	105 (57)	376 (45)	922 (52)	1,359 (39)	<0.001
Online (websites, email)	632 (68)	473 (82)	71 (87)	671 (90)	186 (68)	626 (81)	156 (74)	317 (64)	160 (77)	696 (83)	1,205 (74)	2,783 (80)	<0.001
Social media and messenger apps	753 (81)	492 (86)	72 (87)	587 (79)	90 (32)	240 (31)	106 (55)	186 (38)	111 (55)	359 (42)	1,132 (67)	1,864 (49)	<0.001
Government/institution’s web page	711 (75)	470 (83)	69 (86)	662 (90)	194 (75)	547 (72)	173 (74)	432 (86)	138 (63)	707 (84)	1,285 (75)	2,818 (81)	0.001
WHO web page	246 (30)	340 (61)	66 (81)	637 (85)	122 (50)	487 (65)	149 (60)	382 (74)	123 (49)	555 (64)	706 (50)	2,401 (67)	<0.001

Suppl. Table 35 Most prevalent topic areas with unclear or conflicting COVID-19 information, and most prevalent 'fake news', breakdown by country

Values in cells are n (weighted %) of respondents who replied 'yes'.

Variable and categories	Thailand	Malaysia	UK	Italy	Slovenia	Total	P-value
	N=1,476	N=827	N=1,009	N=712	N=1,034	N=5,058	
**Have you seen any unclear or conflicting information about COVID-19 in the last month?**							
Ways to avoid the infection	564 (36)	409 (47)	679 (68)	410 (64)	682 (64)	2,744 (54)	<0.001
Symptoms of COVID-19	568 (36)	353 (42)	590 (62)	328 (44)	494 (44)	2,333 (45)	<0.001
What to do in case of symptoms	506 (34)	295 (37)	438 (43)	293 (45)	435 (42)	1,967 (40)	0.058
Social distancing guidance	490 (33)	292 (42)	568 (56)	314 (42)	559 (51)	2,223 (44)	<0.001
Quarantine/isolation	529 (36)	314 (39)	547 (54)	292 (41)	559 (52)	2,241 (44)	<0.001
Penalties if disobey restrictions	614 (41)	384 (42)	620 (60)	378 (52)	508 (45)	2,504 (47)	<0.001
Risks in case of infection	527 (34)	327 (37)	542 (54)	330 (49)	493 (46)	2,219 (43)	<0.001
Numbers of coronavirus cases/deaths related to COVID-19	563 (37)	284 (47)	741 (72)	457 (66)	463 (46)	2,508 (52)	<0.001
Government support schemes (e.g. financial)	779 (51)	432 (53)	438 (46)	492 (69)	572 (51)	2,713 (53)	<0.001
Testing	531 (34)	376 (39)	734 (72)	520 (72)	534 (49)	2,695 (51)	<0.001
Travel restrictions (e.g. curfew, restricted hours of movement)	520 (33)	407 (43)	641 (62)	382 (55)	533 (45)	2,483 (46)	<0.001
**Have you come across news about the following COVID-19 topics that seemed fake to you?**							
General spread of fear	668 (42)	606 (70)	693 (72)	382 (58)	771 (69)	3,120 (60)	<0.001
Coronavirus as an engineered modified virus	543 (32)	613 (65)	819 (81)	613 (82)	864 (75)	3,452 (63)	<0.001
Minimisation of risks	440 (27)	416 (39)	579 (55)	540 (69)	731 (62)	2,706 (48)	<0.001
Numbers of infected/deceased people	512 (33)	400 (47)	615 (61)	475 (75)	574 (54)	2,576 (51)	<0.001
Unreasonable health recommendations	517 (32)	545 (55)	574 (57)	385 (50)	650 (60)	2,671 (49)	<0.001
Pharmaceutical conspiracy	490 (32)	440 (50)	525 (54)	489 (63)	673 (61)	2,617 (49)	<0.001
Home-made recipes to make sanitizer products	538 (32)	573 (61)	557 (56)	516 (70)	603 (51)	2,787 (51)	<0.001
Alternative drugs/cure	537 (33)	581 (60)	697 (67)	444 (58)	612 (51)	2,871 (51)	<0.001
Fear toward products coming from infected countries	458 (29)	549 (63)	483 (49)	425 (56)	519 (48)	2,434 (46)	<0.001

For peer review only - http://bmjopen.bmj.com/site/about/guidelines.xhtml

Suppl. Table 36 Most prevalent topic areas with unclear or conflicting COVID-19 information, and most prevalent ‘fake news’, breakdown by country and education level

P/S = primary or lower/secondary education; T = tertiary education. Values in cells are n (weighted %) of respondents who replied ‘yes’.

Variable and categories	Thailand		Malaysia		UK		Italy		Slovenia		Total		
**Education level**	**P/S**	**T**	**P/S**	**T**	**P/S**	**T**	**P/S**	**T**	**P/S**	**T**	**P/S**	**T**	**P-value (for total)**
	N=909	N=567	N=82	N=745	N=247	N=762	N=217	N=495	N=202	N=832	N=1,657	N=3,401	
**Have you seen any unclear or conflicting information about COVID-19 in the last month?**													
Ways to avoid the infection	276 (33)	288 (51)	37 (46)	372 (49)	153 (66)	526 (69)	119 (65)	291 (60)	125 (63)	557 (67)	710 (50)	2,034 (62)	<0.001
Symptoms	268 (33)	300 (53)	36 (43)	317 (41)	146 (65)	444 (59)	94 (42)	234 (48)	96 (44)	398 (46)	640 (42)	1,693 (51)	<0.001
What to do in case of symptoms	245 (31)	261 (47)	32 (38)	263 (36)	96 (42)	342 (44)	94 (46)	199 (43)	80 (42)	355 (41)	547 (38)	1,420 (43)	0.026
Social distancing guidance	249 (31)	241 (42)	36 (44)	256 (34)	113 (51)	455 (61)	92 (41)	222 (46)	109 (50)	450 (53)	599 (41)	1,624 (51)	<0.001
Quarantine/isolation	278 (34)	251 (45)	32 (40)	282 (38)	123 (51)	424 (56)	84 (41)	208 (43)	102 (50)	457 (55)	619 (41)	1,622 (50)	<0.001
Penalties if disobey restrictions	315 (38)	299 (52)	34 (40)	350 (48)	143 (56)	477 (62)	103 (50)	275 (56)	101 (44)	407 (47)	696 (44)	1,808 (55)	<0.001
Risks in case of infection	257 (31)	270 (49)	32 (36)	295 (39)	127 (54)	415 (55)	105 (50)	225 (46)	93 (45)	400 (47)	614 (40)	1,605 (49)	<0.001
Numbers of coronavirus cases/deaths related to COVID-19	284 (33)	279 (52)	42 (50)	242 (33)	172 (70)	569 (74)	140 (67)	317 (65)	107 (50)	356 (41)	745 (49)	1,763 (56)	0.001
Government support schemes (e.g. financial)	402 (47)	377 (69)	44 (54)	388 (52)	103 (50)	335 (43)	138 (69)	354 (71)	108 (50)	464 (54)	795 (52)	1,918 (55)	0.257
Testing	258 (31)	273 (49)	31 (38)	345 (45)	161 (68)	573 (75)	145 (70)	375 (76)	95 (48)	439 (51)	690 (46)	2,005 (62)	<0.001
Travel restrictions (e.g. curfew, restricted hours of movement)	248 (30)	272 (49)	36 (42)	371 (49)	142 (59)	499 (65)	112 (55)	270 (55)	96 (41)	437 (51)	634 (42)	1,849 (56)	<0.001
**Have you come across news about the following COVID-19 topics that seemed fake to you?**													
General spread of fear	308 (37)	360 (64)	56 (69)	550 (73)	182 (76)	511 (68)	116 (60)	266 (54)	147 (66)	624 (74)	809 (57)	2,311 (67)	<0.001
Coronavirus as an engineered modified virus	209 (26)	334 (61)	52 (62)	561 (76)	193 (80)	626 (82)	174 (80)	439 (89)	156 (70)	708 (84)	784 (56)	2,668 (79)	<0.001
Minimisation of risks	178 (23)	262 (47)	31 (36)	385 (51)	128 (52)	451 (59)	141 (63)	399 (81)	122 (56)	609 (71)	600 (41)	2,106 (62)	<0.001
Numbers of infected/deceased people	231 (29)	281 (51)	40 (47)	360 (49)	152 (62)	463 (61)	153 (719	322 (67)	118 (55)	456 (54)	694 (49)	1,882 (57)	<0.001
Unreasonable health recommendations	204 (27)	313 (57)	45 (52)	500 (66)	131 (55)	443 (59)	101 (46)	284 (60)	122 (58)	528 (64)	603 (44)	2,068 (61)	<0.001
Pharmaceutical conspiracy	239 (29)	251 (45)	41 (49)	399 (54)	131 (56)	394 (52)	138 (60)	351 (71)	125 (58)	548 (64)	674 (46)	1,943 (57)	<0.001
Home-made recipes to make sanitizer products	230 (27)	308 (55)	51 (59)	522 (69)	158 (62)	399 (51)	149 (68)	367 (75)	104 (46)	499 (59)	692 (47)	2,095 (59)	<0.001
Alternative drugs/cure	240 (28)	297 (53)	48 (57)	533 (71)	168 (65)	529 (69)	125 (55)	319 (66)	105 (44)	507 (61)	686 (46)	2,185 (64)	<0.001
Fear toward products coming from infected countries	197 (25)	261 (46)	52 (62)	497 (67)	127 (52)	356 (46)	126 (55)	299 (59)	100 (46)	419 (51)	602 (44)	1,832 (51)	<0.001

Suppl. Table 37 Most prevalent topic areas with unclear or conflicting COVID-19 information, and most prevalent 'fake news', breakdown by country and self-reported level of understanding of COVID-19

H = high/very high/expert level; S = some; N = a little/none at all. Values in cells are n (weighted %) of respondents who replied 'yes'.

**Variable and categories**	**Thailand**			**Malaysia**			**UK**			**Italy**			**Slovenia**			**Total**			
**Self-reported level of understanding of COVID-19**	**H**	**S**	**N**	**H**	**S**	**N**	**H**	**S**	**N**	**H**	**S**	**N**	**H**	**S**	**N**	**H**	**S**	**N**	**P-value (for total)**
	N=965	N=459	N=52	N=435	N=359	N=33	N=647	N=336	N=26	N=368	N=324	N=20	N=713	N=279	N=42	N=3,128	N=1,757	N=173	
**Have you seen any unclear or conflicting information about COVID-19 in the last month?**																			
Ways to avoid the infection	401 (40)	145 (32)	18 (19)	197 (43)	191 (46)	21 (63)	416 (63)	248 (76)	15 (53)	202 (54)	193 (72)	15 (73)	445 (61)	211 (73)	26 (53)	1,661 (51)	988 (58)	95 (51)	0.094
Symptoms of COVID-19	400 (40)	150 (33)	18 (19)	170 (36)	167 (49)	16 (51)	363 (58)	210 (66)	17 (79)	147 (31)	163 (53)	18 (81)	312 (40)	164 (54)	18 (41)	1,392 (42)	854 (50)	87 (49)	0.026
What to do in case of symptoms	361 (37)	129 (30)	16 (17)	134 (34)	145 (41)	16 (39)	272 (39)	156 (49)	10 (59)	138 (34)	144 (55)	11 (49)	285 (37)	130 (52)	20 (40)	1,190 (37)	704 (44)	73 (37)	0.041
Social distancing guidance	349 (37)	124 (27)	17 (19)	132 (36)	144 (43)	16 (62)	355 (52)	199 (62)	14 (70)	163 (38)	140 (45)	11 (65)	362 (47)	170 (58)	27 (64)	1,361 (42)	777 (46)	85 (54)	0.168
Quarantine/isolation	379 (39)	139 (32)	11 (11)	153 (33)	145 (39)	16 (71)	338 (49)	193 (59)	16 (76)	148 (39)	135 (44)	9 (39)	372 (50)	165 (58)	22 (41)	1,390 (43)	777 (46)	74 (50)	0.397
Penalties if disobey restrictions	477 (49)	126 (28)	11 (11)	186 (35)	180 (46)	18 (56)	381 (54)	225 (68)	14 (66)	187 (47)	180 (56)	11 (69)	324 (44)	162 (48)	22 (53)	1,555 (47)	873 (48)	76 (47)	0.906
Risks in case of infection	381 (38)	132 (29)	14 (15)	152 (29)	158 (43)	17 (50)	337 (50)	191 (62)	14 (46)	158 (43)	156 (53)	16 (73)	312 (46)	159 (45)	22 (45)	1,340 (41)	796 (46)	83 (42)	0.343
Numbers of coronavirus cases/deaths related to COVID-19	416 (42)	134 (29)	13 (15)	129 (41)	137 (50)	18 (68)	463 (66)	261 (81)	17 (77)	233 (67)	214 (66)	10 (57)	284 (43)	156 (53)	23 (57)	1,525 (50)	902 (54)	81 (54)	0.276
Government support schemes (e.g. financial)	583 (60)	178 (38)	18 (20)	208 (46)	203 (61)	21 (62)	269 (40)	158 (53)	11 (56)	248 (67)	227 (71)	17 (78)	372 (48)	176 (59)	24 (48)	1,680 (52)	942 (55)	91 (50)	0.590
Testing	392 (39)	124 (29)	15 (15)	181 (36)	179 (46)	16 (32)	467 (70)	249 (74)	18 (77)	266 (71)	239 (71)	15 (86)	357 (48)	154 (55)	23 (31)	1,663 (50)	945 (53)	87 (39)	0.108
Travel restrictions (e.g. curfew, restricted hours of movement)	391 (39)	118 (25)	11 (11)	209 (37)	178 (46)	20 (62)	398 (60)	228 (71)	15 (52)	192 (50)	176 (58)	14 (78)	341 (43)	167 (50)	25 (41)	1,531 (44)	867 (49)	85 (47)	0.356

For peer review only - http://bmjopen.bmj.com/site/about/guidelines.xhtml

**Have you come across news about the following COVID-19 topics that seemed fake to you?**																			
General spread of fear	488 (47)	158 (36)	22 (23)	320 (65)	266 (80)	20 (56)	449 (70)	228 (73)	16 (81)	208 (57)	163 (59)	11 (61)	518 (71)	222 (65)	31 (66)	1,983 (61)	1,037 (60)	100 (54)	0.594
Coronavirus as an engineered modified virus	390 (37)	134 (26)	19 (19)	327 (71)	266 (62)	20 (46)	532 (83)	268 (79)	19 (70)	320 (87)	277 (80)	16 (60)	598 (80)	231 (65)	35 (75)	2,167 (66)	1,176 (60)	109 (49)	0.007
Minimisation of risks	305 (30)	120 (24)	15 (13)	222 (38)	176 (41)	18 (32)	377 (56)	191 (56)	11 (39)	277 (64)	249 (74)	14 (54)	510 (64)	196 (57)	25 (47)	1,691 (48)	932 (49)	83 (33)	0.063
Numbers of infected/deceased people	345 (34)	148 (33)	19 (18)	206 (49)	174 (48)	20 (39)	392 (58)	207 (66)	16 (75)	252 (76)	214 (75)	9 (63)	377 (51)	172 (62)	25 (61)	1,572 (49)	915 (55)	89 (45)	0.105
Unreasonable health recommendations	387 (36)	113 (26)	17 (17)	286 (54)	237 (53)	22 (63)	375 (55)	186 (58)	13 (71)	211 (57)	163 (44)	11 (54)	440 (59)	186 (65)	24 (48)	1,699 (50)	885 (47)	87 (50)	0.538
Pharmaceutical conspiracy	358 (36)	112 (25)	20 (21)	238 (53)	188 (48)	14 (38)	355 (55)	158 (51)	12 (56)	266 (69)	209 (57)	14 (65)	453 (61)	192 (61)	28 (45)	1,670 (52)	859 (46)	88 (40)	0.059
Home-made recipes to make sanitizer products	400 (38)	122 (24)	16 (15)	309 (62)	241 (62)	23 (57)	366 (56)	179 (55)	12 (68)	274 (78)	227 (62)	15 (71)	411 (52)	170 (51)	22 (45)	1,760 (52)	939 (49)	88 (48)	0.390
Alternative drugs/cure	409 (39)	112 (24)	16 (16)	305 (57)	257 (75)	19 (20)	468 (72)	214 (62)	15 (50)	243 (64)	188 (52)	13 (66)	430 (53)	159 (45)	23 (58)	1,855 (54)	930 (49)	86 (33)	0.004
Fear toward products coming from infected countries	330 (33)	109 (23)	19 (20)	297 (65)	234 (68)	18 (39)	317 (50)	155 (48)	11 (44)	226 (58)	187 (55)	12 (64)	352 (47)	145 (49)	22 (46)	1,522 (47)	830 (46)	82 (39)	0.456

For peer review only - http://bmjopen.bmj.com/site/about/guidelines.xhtml

STROBE 2007 (v4) checklist of items to be included in reports of observational studies in epidemiology*
Checklist for cohort, case-control, and cross-sectional studies (combined)

Section/Topic	Item #	Recommendation	Reported on page #
**Title and abstract**	1	(*a*) Indicate the study's design with a commonly used term in the title or the abstract	2
		(*b*) Provide in the abstract an informative and balanced summary of what was done and what was found	2
**Introduction**			
Background/rationale	2	Explain the scientific background and rationale for the investigation being reported	3
Objectives	3	State specific objectives, including any pre-specified hypotheses	4
**Methods**			
Study design	4	Present key elements of study design early in the paper	4
Setting	5	Describe the setting, locations, and relevant dates, including periods of recruitment, exposure, follow-up, and data collection	4,5
Participants	6	(*a*) *Cohort study*—Give the eligibility criteria, and the sources and methods of selection of participants. Describe methods of follow-up<br>*Case-control study*—Give the eligibility criteria, and the sources and methods of case ascertainment and control selection. Give the rationale for the choice of cases and controls<br>*Cross-sectional study*—Give the eligibility criteria, and the sources and methods of selection of participants	This is a survey<br>5
		(*b*) *Cohort study*—For matched studies, give matching criteria and number of exposed and unexposed<br>*Case-control study*—For matched studies, give matching criteria and the number of controls per case	NA
Variables	7	Clearly define all outcomes, exposures, predictors, potential confounders, and effect modifiers. Give diagnostic criteria, if applicable	4
Data sources/ measurement	8*	For each variable of interest, give sources of data and details of methods of assessment (measurement). Describe comparability of assessment methods if there is more than one group	4
Bias	9	Describe any efforts to address potential sources of bias	6
Study size	10	Explain how the study size was arrived at	6
Quantitative variables	11	Explain how quantitative variables were handled in the analyses. If applicable, describe which groupings were chosen and why	6
Statistical methods	12	(*a*) Describe all statistical methods, including those used to control for confounding	6
		(*b*) Describe any methods used to examine subgroups and interactions	6
		(*c*) Explain how missing data were addressed	No missing data. only completed surveys can be submitted

For peer review only

		(*d*) *Cohort study*—If applicable, explain how loss to follow-up was addressed<br>*Case-control study*—If applicable, explain how matching of cases and controls was addressed<br>*Cross-sectional study*—If applicable, describe analytical methods taking account of sampling strategy	NA
		(*e*) Describe any sensitivity analyses	NA
**Results**			
Participants	13*	(a) Report numbers of individuals at each stage of study—eg numbers potentially eligible, examined for eligibility, confirmed eligible, included in the study, completing follow-up, and analysed	6
		(b) Give reasons for non-participation at each stage	5
		(c) Consider use of a flow diagram	NA
Descriptive data	14*	(a) Give characteristics of study participants (eg demographic, clinical, social) and information on exposures and potential confounders	6
		(b) Indicate number of participants with missing data for each variable of interest	NA
		(c) *Cohort study*—Summarise follow-up time (eg, average and total amount)	NA
Outcome data	15*	*Cohort study*—Report numbers of outcome events or summary measures over time	NA
		Case-control study—Report numbers in each exposure category, or summary measures of exposure	NA
		Cross-sectional study—Report numbers of outcome events or summary measures	NA
Main results	16	(*a*) Give unadjusted estimates and, if applicable, confounder-adjusted estimates and their precision (eg, 95% confidence interval). Make clear which confounders were adjusted for and why they were included	7-12
		(*b*) Report category boundaries when continuous variables were categorized	7-12
		(*c*) If relevant, consider translating estimates of relative risk into absolute risk for a meaningful time period	NA
Other analyses	17	Report other analyses done—eg analyses of subgroups and interactions, and sensitivity analyses	7-12
**Discussion**			
Key results	18	Summarise key results with reference to study objectives	12
Limitations	19	Discuss limitations of the study, taking into account sources of potential bias or imprecision. Discuss both direction and magnitude of any potential bias	14-15
Interpretation	20	Give a cautious overall interpretation of results considering objectives, limitations, multiplicity of analyses, results from similar studies, and other relevant evidence	12-14
Generalisability	21	Discuss the generalisability (external validity) of the study results	14-15
**Other information**			
Funding	22	Give the source of funding and the role of the funders for the present study and, if applicable, for the original study on which the present article is based	16

*Give information separately for cases and controls in case-control studies and, if applicable, for exposed and unexposed groups in cohort and cross-sectional studies.

For peer review only

Note: An Explanation and Elaboration article discusses each checklist item and gives methodological background and published examples of transparent reporting. The STROBE checklist is best used in conjunction with this article (freely available on the Web sites of PLoS Medicine at http://www.plosmedicine.org/, Annals of Internal Medicine at http://www.annals.org/, and Epidemiology at http://www.epidem.com/). Information on the STROBE Initiative is available at www.strobe-statement.org.

For peer review only

BMJ Open

Economic and social impacts of COVID-19 and public health measures: results from an anonymous online survey in Thailand, Malaysia, the United Kingdom, Italy and Slovenia

Journal:	*BMJ Open*
Manuscript ID	bmjopen-2020-046863.R1
Article Type:	Original research
Date Submitted by the Author:	17-Mar-2021

Complete List of Authors:	Osterrieder, Anne; Mahidol University Rajvithi Campus, Mahidol Oxford Tropical Medicine Research Unit, Faculty of Tropical Medicine; University of Oxford, Centre for Tropical Medicine and Global Health, Nuffield Department of Medicine<br>Cuman, Giulia; University Hospital of Padova, Paediatric Ethics Committee; Research Ethics Committee<br>Pan-Ngum, Wirichada; Mahidol University Rajvithi Campus, Department of Tropical Hygiene Faculty of Tropical Medicine; Mahidol University Rajvithi Campus, Mahidol Oxford Tropical Medicine Research Unit, Faculty of Tropical Medicine<br>Cheah, Phaik Kin; Universiti Tunku Abdul Rahman, Faculty of Arts and Social Science<br>Cheah, Phee-Kheng; Ministry of Health, Malaysia, Emergency & Trauma Department, Sabah Women & Children's Hospital<br>Peerawaranun, Pimnara; Mahidol University Rajvithi Campus, Mahidol Oxford Tropical Medicine Research Unit, Faculty of Tropical Medicine<br>Silan, Margherita; University of Padua, Department of Statistical Sciences<br>Orazem, Miha; University of Ljubljana, Faculty of Medicine; Institute of Oncology Ljubljana, Department of Radiation Oncology<br>Perkovic, Ksenija; Science and Research Centre Koper, Institute for Social Studies<br>Groselj, Urh; University of Ljubljana, Faculty of Medicine; University Children's Hospital, University 26 Medical Center – University Children's Hospital Ljubljana,, Department of Endocrinology, Diabetes and Metabolic Diseases<br>Schneiders, Mira Leonie; Mahidol University Rajvithi Campus, Mahidol Oxford Tropical Medicine Research Unit, Faculty of Tropical Medicine; Mahidol University Rajvithi Campus, Mahidol Oxford Tropical Medicine Research Unit, Faculty of Tropical Medicine<br>Poomchaichote, Tassawan; Mahidol University Rajvithi Campus, Mahidol Oxford Tropical Medicine Research Unit, Faculty of Tropical Medicine<br>Waithira, Naomi; Mahidol University Rajvithi Campus, Mahidol Oxford Tropical Medicine Research Unit, Faculty of Tropical Medicine; University of Oxford, Centre for Tropical Medicine and Global Health, Nuffield Department of Medicine<br>Asarath, Supa-at; Mahidol University Rajvithi Campus, Mahidol Oxford Tropical Medicine Research Unit, Faculty of Tropical Medicine<br>Naemiratch, Bhensri; Mahidol University Rajvithi Campus, Mahidol

	Oxford Tropical Medicine Research Unit, Faculty of Tropical Medicine<br>Ruangkajorn, Supanat; Mahidol University Rajvithi Campus, Mahidol Oxford Tropical Medicine Research Unit, Faculty of Tropical Medicine<br>Skof, Lenart; Science and Research Centre Koper, Institute for Philosophical Studies<br>Kulpijit, Natinee; Mahidol University Rajvithi Campus, Mahidol Oxford Tropical Medicine Research Unit, Faculty of Tropical Medicine<br>Mackworth-Young, Constance; London School of Hygiene & Tropical Medicine, Department of Global Health & Development<br>Ongkili, Darlene; Ministry of Health, Malaysia, Emergency & Trauma Department, Queen Elizabeth Hospital<br>Chanviriyavuth, Rita; Mahidol University Rajvithi Campus, Mahidol Oxford Tropical Medicine Research Unit, Faculty of Tropical Medicine<br>Mukaka, Mavuto; Mahidol University Rajvithi Campus, Mahidol Oxford Tropical Medicine Research Unit, Faculty of Tropical Medicine; University of Oxford, Centre for Tropical Medicine and Global Health, Nuffield Department of Medicine<br>Cheah, Phaikyeong; Mahidol University Rajvithi Campus, Mahidol Oxford Tropical Medicine Research Unit, Faculty of Tropical Medicine; University of Oxford, Centre for Tropical Medicine and Global Health, Nuffield Department of Medicine
**Primary Subject Heading**:	Global health
Secondary Subject Heading:	Public health
Keywords:	Public health < INFECTIOUS DISEASES, Epidemiology < TROPICAL MEDICINE, PUBLIC HEALTH

SCHOLARONE™
Manuscripts

I, the Submitting Author has the right to grant and does grant on behalf of all authors of the Work (as defined in the below author licence), an exclusive licence and/or a non-exclusive licence for contributions from authors who are: i) UK Crown employees; ii) where BMJ has agreed a CC-BY licence shall apply, and/or iii) in accordance with the terms applicable for US Federal Government officers or employees acting as part of their official duties; on a worldwide, perpetual, irrevocable, royalty-free basis to BMJ Publishing Group Ltd ("BMJ") its licensees and where the relevant Journal is co-owned by BMJ to the co-owners of the Journal, to publish the Work in this journal and any other BMJ products and to exploit all rights, as set out in our licence.

The Submitting Author accepts and understands that any supply made under these terms is made by BMJ to the Submitting Author unless you are acting as an employee on behalf of your employer or a postgraduate student of an affiliated institution which is paying any applicable article publishing charge ("APC") for Open Access articles. Where the Submitting Author wishes to make the Work available on an Open Access basis (and intends to pay the relevant APC), the terms of reuse of such Open Access shall be governed by a Creative Commons licence – details of these licences and which Creative Commons licence will apply to this Work are set out in our licence referred to above.

Other than as permitted in any relevant BMJ Author's Self Archiving Policies, I confirm this Work has not been accepted for publication elsewhere, is not being considered for publication elsewhere and does not duplicate material already published. I confirm all authors consent to publication of this Work and authorise the granting of this licence.

For peer review only

Economic and social impacts of COVID-19 and public health measures: results from an anonymous online survey in Thailand, Malaysia, the United Kingdom, Italy and Slovenia

Anne Osterrieder[1,2], Giulia Cuman[3], Wirichada Pan-ngum[1,4], Phaik Kin Cheah[5], Phee-Kheng Cheah[6], Pimnara Peerawaranun[1], Margherita Silan[7], Miha Orazem[8,9], Ksenija Perkovic[10], Urh Groselj[8,11], Mira Leonie Schneiders[1,2,12], Tassawan Poomchaichote[1,13], Naomi Waithira[1,2], Supa-at Asarath[1], Bhensri Naemiratch[1], Supanat Ruangkajorn[1], Lenart Skof[14], Natinee Kulpijit[1], Constance R.S. Mackworth-Young[15], Darlene Ongkili[16], Rita Chanviriyavuth[1], Mavuto Mukaka[1,2], Phaik Yeong Cheah[1,2,12,13]

[1]Mahidol Oxford Tropical Medicine Research Unit, Faculty of Tropical Medicine, Mahidol University, Bangkok, 10400, Thailand

[2]Centre for Tropical Medicine & Global Health, Nuffield Department of Medicine, University of Oxford, Oxford, UK

[3]Paediatric Ethics Committee; Research Ethics Committee, University Hospital of Padua, Padua, Italy

[4]Department of Tropical Hygiene, Faculty of Tropical Medicine, Mahidol University, Bangkok, 10400, Thailand

[5]Faculty of Arts and Social Science, Universiti Tunku Abdul Rahman, Kampar, Malaysia

[6]Emergency and Trauma Department, Sabah Women and Children's Hospital, Ministry of Health Malaysia, Kota Kinabalu, Malaysia

[7]Department of Statistical Sciences, University of Padua, Padua, Italy

[8]Faculty of Medicine, University of Ljubljana, Ljubljana, Slovenia

[9]Department of Radiation Oncology, Institute of Oncology Ljubljana, Ljubljana, Slovenia

[10]Institute for Social Studies, Science and Research Centre Koper, Koper, Slovenia

[11]Department of Endocrinology, Diabetes and Metabolic Diseases, University Children's Hospital, University Medical Center – University Children's Hospital Ljubljana, Ljubljana, Slovenia

[12]Ethox Centre, Nuffield Department of Population Health, University of Oxford, Oxford, UK

[13]The SoNAR-Global Network

[14]Institute for Philosophical Studies, Science and Research Centre Koper, Koper, Slovenia

[15]Department of Global Health and Development, London School of Hygiene and Tropical Medicine, London, UK

[16]Emergency and Trauma Department, Queen Elizabeth Hospital, Ministry of Health Malaysia, Kota Kinabalu, Malaysia

Corresponding author: Phaik Yeong Cheah, 420/6 Mahidol-Oxford Tropical Medicine 24 Research Unit, Faculty of Tropical Medicine, Rajvithi Road, Bangkok, Thailand, 10400. Email: phaikyeong@tropmedres.ac

Word count: abstract 300 words, main text 5757 words

For peer review only - http://bmjopen.bmj.com/site/about/guidelines.xhtml

Keywords: COVID-19, SARS-CoV-2, non-pharmaceutical interventions, restrictions, social distancing, quarantine, isolation, compliance, information, fake news, social, behavioural, survey

Abstract

Objectives

To understand the impact of coronavirus disease 2019 (COVID-19) and public health measures on different social groups, we conducted a mixed-methods study in five countries ('SEBCOV - Social, ethical and behavioural aspects of COVID-19'). Here we report the results of the online survey.

Study design and statistical analysis

Overall, 5,058 respondents from Thailand, Malaysia, the United Kingdom, Italy and Slovenia completed the self-administered survey between May and June 2020. Post-stratification weighting was applied, and associations between categorical variables assessed. Frequency counts and percentages were used to summarise categorical data. Associations between categorical variables were assessed using Pearson's Chi-squared test. Data was analysed in Stata 15.0

Results

Among the five countries, Thai respondents reported having been most, and Slovenian respondents least, affected economically. The following factors were associated with greater negative economic impacts: being 18-24 years or 65 years or older; lower education levels; larger households; having children under 18 in the household; and and having flexible/no income. Regarding social impact, respondents expressed most concern about their social life, physical health, mental health and wellbeing.

There were large differences between countries in terms of voluntary behavioural change, and in compliance and agreement with COVID-19 restrictions. Overall, self-reported compliance was higher among respondents who self-reported a high understanding of COVID-19. UK respondents felt able to cope the longest and Thai respondents the shortest with only going out for essential needs or work. Many respondents reported seeing news perceived to be fake, the proportion varying between countries, with education level and self-reported levels of understanding of COVID-19.

Conclusions

Our data showed that COVID-19 and public health measures have uneven economic and social impacts on people from different countries and social groups. Understanding the factors associated with these impacts can help to inform future public health interventions and mitigate their negative consequences.

Registration: TCTR20200401002

For peer review only - http://bmjopen.bmj.com/site/about/guidelines.xhtml

Summary

Strengths

- Our research findings help to address an evidence gap as identified by the global research community in a recent study on COVID-19 research priorities, which identified public health messaging, compliance and trust in public health interventions, and evaluation of these interventions in varied settings as areas of high priority (BMJ Global Health Vol 5, Issue 7 (https://gh.bmj.com/content/5/7/e003306).
- Because we recruited a reasonably large sample size in each country (between 700-1400), we were able to compare population segments (e.g. men versus women, younger versus older people, those with lower versus higher levels of education) in the whole cohort, and between countries.
- Our online survey enabled us to capture people's experiences and concerns in multiple domains, in five countries, all of which had restrictions in place, during the relatively early stage of the COVID-19 pandemic.

Limitations

- We did not aim to obtain nationally representative samples and acknowledge that although we used weighting strategies in our analysis, our results may not be fully representative of the populations in the respective countries.
- Our study captured the views and perceptions of respondents on the socio-economic impact of COVID-19 public health measures, rather than data on standard indicators of economic and social impacts.

For peer review only

Introduction

Coronavirus disease 2019 (COVID-19) is a respiratory disease caused by the novel coronavirus 'severe acute respiratory syndrome coronavirus 2' (SARS-CoV2), which is transmitted through droplets, close contact, and aerosols[1 2]. The SARS-CoV2 outbreak was first reported in December 2019 in Wuhan, China[3], with the World Health Organization (WHO) declaring it Public Health Emergency of International Concern on 30th January 2020 and a global pandemic on 11th March 2020[1].

In the absence of widely available vaccines and pharmaceutical treatments, the impact of COVID-19 is being mitigated using non-pharmaceutical interventions (NPIs)[4 5]. Examples of NPIs include: social distancing (or 'physical distancing') measures, such as isolation of sick individuals, quarantine of exposed individuals, contact tracing, voluntary shielding, travel-related restrictions; and personal protective measures, such as hand hygiene and wearing face masks[4 6 7]. Scientific evidence indicates

For peer review only - http://bmjopen.bmj.com/site/about/guidelines.xhtml

that NPIs are effective measures to contain a pandemic and ease pressures on health care systems[6-12]. However, authorities and policy makers need to consider the societal, economic and ethical impacts of these public health measures, in particular on vulnerable groups[13 14]. Such groups might be disproportionally affected by NPIs and/or might be unable to comply with them[15], e.g. due to loss of income when having to isolate at home, crowded living conditions[14], or not being able to afford masks[16].

As the COVID-19 pandemic continues, evidence is urgently needed to understand how people perceive and experience NPIs, which groups are disproportionally negatively affected by NPIs, and how communication is perceived by various social groups[17]. These data can be used to supplement standard indicators of economic and social impacts to provide a better understanding of the effects of COVID-19 and its related public health measures. This understanding is important so that the policies can be improved to minimize the negative impact of COVID-19 on people's lives, and to improve communications.

Here we report the highlights of an online survey conducted in Southeast Asia (Thailand and Malaysia, both upper middle-income countries), and Europe (the United Kingdom, Italy and Slovenia, all high-income countries) between May 1 to June 30, 2020 as part of the mixed-methods study 'Social, ethical and behavioural aspects of COVID-19' (SEBCOV)[18]. These findings help to address an evidence gap as identified by the global research community in a recent study on COVID-19 research priorities[19], which identified public health messaging, compliance and trust in public health interventions, and evaluation of these interventions in varied settings as areas of high priority[19].

Methods

Study area

The survey was conducted in five countries (population in 2020 indicated in brackets[20]): Thailand (69.8 million) and Malaysia (population = 32.4 million) in Southeast Asia; and United Kingdom (67.9 million), Italy (60.5 million) and Slovenia (2.1 million) in Europe.

Survey development

The survey contained five sections with 36 questions (single-answer multiple choice and five-point Likert scales) on (1) socio-demographic information; (2) income, occupation status and economic impacts of COVID-19 restrictions; (3) sources of, preferences and perceptions regarding COVID-19 related communication, and the occurrence of 'fake news' (untrue information presented as news); and (4) perceived levels of understanding of COVID-19 and NPIs, agreement with NPIs, voluntary behavioural changes, and concerns and coping strategies relating to restrictions[21]. The Malaysia and

For peer review only - http://bmjopen.bmj.com/site/about/guidelines.xhtml

UK surveys were administered in English, with the other surveys translated into the respective country languages. The self-administered online survey was set up using the 'JISC Online surveys' platform[22].

Patient and public involvement

The survey questions were pilot-tested with 25 people from participating countries, and revised accordingly based on feedback. In addition, the Bangkok Health Research Ethics Interest Group, a public involvement group set up by the Mahidol Oxford Tropical Medicine Research Unit (MORU)[23], discussed the study and the survey questions in a dedicated virtual meeting. Selected questions were tested using an adapted cognitive testing technique using the "thinking out loud" approach[24], and the collaborative virtual sticky notes board 'Padlet'[25].

Participant selection and recruitment

Adults of any age residing in Thailand, Italy, Malaysia, United Kingdom (UK) or Slovenia at the time of the study were eligible to take part. Participants needed to be able to use a computer or smart phone to access the survey and provide online consent to participate.

The survey was open from 1st May to 30th June 2020 (1st-30th June for Slovenia due to late start). Participants were recruited using a combination of approaches: snowball sampling through personal and professional networks (via email, social media and messenger apps, mailing lists, and organisations such as the Medical Chamber[26] in Slovenia); a polling company[27] in Thailand; and through promoted posts on Facebook. Facebook allows users to 'boost' posts to selected demographic audiences for a small fee, so that the post appears on their Facebook newsfeed[28]. To achieve more balanced responses in the categories of gender, education level and geographic distribution, promoted Facebook posts were targeted at people with primary or lower/secondary education in UK and Malaysia; potential participants in Wales, Scotland and Northern Ireland in the UK; and at men in the UK and Italy.

Sample size

Each country aimed to recruit a minimum sample of 600 respondents, exceeding the 40-200 respondents recommended for a mixed-methods study[29]. A minimum sample size of 600 respondents is adequate to estimate the prevalence of a response assuming a 50% prevalence rate, with 95% confidence and with a precision of 4%. The 50% prevalence is the standard assumption for precision sample size calculations when the true prevalence is not available, as this gives the highest sample size for a binomial distribution for a desired level of precision. The following sample size formula

For peer review only - http://bmjopen.bmj.com/site/about/guidelines.xhtml

was used $$n = \frac{Z^2_{1-\alpha/2} P(1-P)}{d^2}$$ where P is the anticipated prevalence, d is the margin of error, $Z_{1-\alpha/2}$ is the standard normal value corresponding to the upper tail probability of α/2, α=0.05 (for a 95% confidence interval), n is the sample size.

Statistical analysis

To simplify analysis, answers in the following categories were combined as follows: "slightly agree/highly agree" were combined into one "agree", category, and "slightly/strongly disagree" responses into one "disagree" category. To understand the distribution of the basic demographic variables in the respondent sample, the observed frequencies and sample characteristics are reported using unweighted percentages (Suppl. Table 1). The characteristics for the rest of the variables are presented using the observed survey frequency counts followed by weighted percentages (Suppl. Tables 2-37). Post-stratification weighting was used to align the composition of the respondents' sample with the known distribution of the whole population's characteristics, reducing sampling error. Weights were computed considering three stratifying variables that were available from population census data from each country[30], namely, gender, age and education level. Weights were obtained as the ratio between the proportion of each possible combination of the three variables in the whole country population and the correspondent proportion in the respondent sample.

Survey data was analysed using Stata 15.0 software[31]. Frequency counts and percentages were used to summarise categorical data. Associations between categorical variables were assessed using Pearson's Chi-squared test. P-values have been provided in the tables and considered statistically significant below the two-sided alpha=0.05 level. All p-values presented in the tables are for global tests of significance. Practical significance was taken into account when interpreting differences in the results.

Results

At the time of the inception of this study, governments in Thailand, Malaysia, Italy, the UK and Slovenia had initiated public health measures, using varying degrees of "lockdowns" to curb the pandemic. Figure 1 shows a visualization of the 'Stringency Index' (SI) of the public health responses of the five government over the study period, drawing upon data provided by the Oxford COVID-19 Government Response Tracker (OxCGRT)[32]. The OxCGRT tool tracks government policies and interventions from more than 180 countries on standardized indicators, and aggregates the data into a 'Stringency Index' for each country on a scale from 0-100, with 100 indicating the strictest response[32]. For example, Italy had the strictest public health measures in early May (SI = 93) and then gradually lifted and reintroduced restrictions, whereas restrictions in the UK remained at around the

For peer review only - http://bmjopen.bmj.com/site/about/guidelines.xhtml

same level (SI = 69-76) throughout the study period. Restrictions in Slovenia were substantially eased from June onwards (SI = 33).

Characteristics of survey respondents

A total of 5,058 participants took part in the survey: 1,476 respondents from Thailand (29%), 827 from Malaysia (16%), 1,009 from the UK (20%), 712 from Italy (14%), and 1,034 from Slovenia (20%;Suppl. Table 1, unweighted data). Overall, around 40% identified as male, around 60% as female, and around 1% as 'other/prefer not to say'. Of all respondents, 8% were 18-24 years old, 17% were aged -25-34 years old, 65% were 35-64 years old, and 10% fell into the 65+ age group. Overall, 33% had primary or lower (from here on referred to as 'primary') or secondary education, whereas 67% had tertiary education. Overall, 21% of respondents lived in large households with five or more people. A total of 59% of respondents received a fixed income (salary/benefits/pension), 31% had flexible income (contract and freelance), and 10% received no or 'other income'. Overall, 36% lived with children under 18 years in their household, and 29% reported that they or a household member belonged to a "vulnerable group" (persons aged 70 or older, pregnant women, or people with serious health conditions). Lastly, 19% were healthcare provider/workers. Supplementary Table 1 provides the breakdown by country. All results in the following subsections are presented as weighted percentages.

Views on economic impacts of COVID-19 and public health measures

In order to understand the economic impacts of COVID-19, respondents who had been working before the pandemic (paid or unpaid work) were asked whether COVID-19 had created any work-related inconvenience for them. Overall, 56% of respondents said that they experienced loss of earnings, 44% reduction of working hours, 36% closure of workplace and 14% job loss (Fig. 2, Suppl. Table 2). A total of 75% reported that they continued to work during COVID-19. Of all respondents, 53% expressed financial concerns, and 32% worried about professional/career progression. Our results indicated that the most affected country was Thailand, with 85% of respondents reporting loss of earnings, 23% loss of job, and 86% expressing financial concerns (Suppl. Table 2). In contrast, fewer Slovenian respondents appeared to be affected economically, e.g. 30% reported loss of earnings, 3% reported loss of job, and 27% had financial concerns.

To investigate the impact of public health measures on different social groups, we analyzed responses based on gender, level of education, age group, household size, whether respondents lived with children under 18 years old, and income type.

For peer review only - http://bmjopen.bmj.com/site/about/guidelines.xhtml

Overall, there were no significant differences between male, female and respondents who identified as ‘other/prefer not to say’, and who had been working before COVID-19, in terms of loss of earnings, loss of job, reduction of working hours and closure of workplace (Fig. 2, Suppl. Table 3). Overall, fewer women continued to work during COVID-19 (71% women vs 78% men; p=0.010). The trend was similar at country level, except for Malaysia (73% women versus 67% men; Suppl. Table 3).

Overall, 65% of respondents with primary and secondary education who had been working before COVID-19 reported a loss of earnings, compared to 38% of respondents with tertiary education (p<0.001; Fig. 2, Suppl. Table 4). More respondents with primary/secondary education lost their job (17% versus 8%; p<0.001), and had their working hours reduced (47% versus 37%; p<0.001). Fewer respondents with primary/secondary education continued to work (71%, versus 83%, p<0.001), and 59% reported financial concerns (versus 41%; p<0.001). This trend was mirrored at country level. Respondents with primary/secondary education were most affected in Thailand, where 90% reported loss of earnings, 24% reported loss of job, and 89% reported financial concerns (Suppl. Table 4). Only 65% of respondents with primary/secondary education in Malaysia (versus 90% with tertiary education) and 59% in Italy (versus 79%) continued to work during COVID-19.

In order to assess whether age was a factor associated with economic impact, respondents were divided into four age groups in the analysis: 18-24 year olds, 25-34 year olds, 35-64 year olds, and over 65 year olds (Fig. 2, Suppl. Tables 5a-b). There were significant differences between age groups regarding loss of earnings (p=0.044): 67% of 65+ year olds reported loss of earnings, compared to 59% of 18-24 year olds, 47% of 25-34 year olds and 56% of 35-64 year olds. . There were no significant differences overall regarding loss of job (p=0.053). However, the 18-24 year olds appeared to be most affected through reduction of working hours (p=0.016) and closure of workplace (p=<0.001). Only 54%% of 18-24 year olds and 68% of 65+ year olds continued to work during COVID-19, compared to 78% of 25-34 and 78% of 35-64 year olds (p=0. <0.001). Analysing by country, the 18-24 year olds reported the higher job losses compared to the other groups in Thailand (32%), Malaysia (42%) and the UK (19%). Those over 65 years old were particularly affected in Italy, where 87% of 65+ year olds who had been working before COVID-19 reported loss of earnings, and 42% reported loss of job (N=12). In all countries, fewer 18-24 year olds continued to work during COVID-19, and in all countries except Thailand, fewer 65+ year olds continued to work during COVID-19.

Overall, more respondents living in larger households, and more respondents living with children under 18 in the household reported economic impacts (Fig. 2, Suppl. Tables 6 and 7). Overall, 64% of respondents whose household included 5 people or more reported loss of earnings (compared to 53% of households with 1-4 people; p=0.003), and 20% reported loss of job (compared to 12%; p=0.005; Suppl. Table 6). More respondents with children reported a loss of earnings compared to respondents

For peer review only - http://bmjopen.bmj.com/site/about/guidelines.xhtml

without children (62% versus 53%; p=0.005), and higher job loss (18% versus 12%; p=0.008; Suppl. Table 7). Analysing by country, respondents living with children appeared to be particularly affected in Thailand and Malaysia.

We also analysed responses according to three types of income: fixed income (e.g. fixed salary, benefits or pension), flexible income (e.g. contract, freelance), and other/no income (Fig. 2; Suppl. Table 8). We did not ask for amount of income. Overall, respondents with fixed income were less affected economically than those with flexible or other/no income. Of the latter only 38% reported loss of earnings, compared to 81% of respondents with flexible income and 69% of respondents with other/no income (p<0.001). Only 8% of people with fixed income had lost their job, compared to 22% with flexible income and 27% with other/no income (p<0.001). At country level, the trends were similar (Suppl. Table 8). Fewer people with flexible or other/no income continued to work in Malaysia (42% with flexible/25% with no/other income, compared to 83% with fixed income; p<0.001), UK (57%/62%, compared to 79%; p<0.001), Italy (51%/15%, compared to 81%; p<0.001) and Slovenia (57%/59%, compared to 84%; p<0.001).

Views on social impacts of COVID-19 and public health measures

We asked respondents if they were concerned about the following areas of life if advised no physical contact/not allowed to go out/allowed to go out only for essential needs: caring responsibilities, physical health, recreational pursuits, sports, mental health and wellbeing, living arrangements, infrastructure (e.g. access to transport, internet), social, and religious and spiritual needs/aspects (Suppl. Table 9). Overall, respondents expressed most concern about their social life (64%), their physical health (59%), and their mental health and wellbeing (58%). This trend was largely similar in individual countries, except for Thailand, where caring responsibilities attracted the most concern (62%); Malaysia, where 58% were concerned about religion and spirituality; and Slovenia, where 65% of people worried about recreational aspects. In general, there were no major differences between gender (Suppl. Table 10), age groups (Suppl. Table 11), education level (Suppl. Table 12), household size (Suppl. Table 13), living with children (Suppl. Table 14) or income type (Suppl. Table 15). However, two areas with the most significant differences between demographic groups were caring responsibilities and living arrangements. For example, 52% of women (compared to 42% of men and 46% of 'other/prefer not to say', p<0.001; Suppl. Table 10), and 64% of those living with children under 18 (compared to 38% of those without children, p<0.001; Suppl. Table 14) expressed concerns about caring responsilibities. Concerns about living arrangements were reported by 33% of those with primary/secondary education (compared to 26% with tertiary, p<0.001; Suppl. Table 12), and 41% of those living in househoulds with 5 or more people (compared to 28% in households with 1-4 people, p<0.001; Suppl. Table 13). We asked respondents how many days they could cope with not going out except for essential needs/work, with answer options ranging from one to 59 days or

For peer review only - http://bmjopen.bmj.com/site/about/guidelines.xhtml

more. In total, 44% of respondents said that they could cope for 29 days or longer (Suppl. Table 16). However, coping time varied significantly between countries (p<0.001): in the UK, 60% of people felt they would be able to cope for 29 days or longer, whereas in Thailand, only 26% of respondents said that they could cope this long. Overall, gender and age did not appear to be associated with coping time (Suppl. Tables 17-18). Factors that appeared to be associated with lower coping times were living in households with 5 or more people (p=0.023, Suppl. Table 19), with children under 18 years (p=0.004, Suppl. Table 20), having primary/secondary education (p<0.001, Suppl. Table 21), and receiving flexible income (p<0.001; Suppl. Table 22). Indicators varied at country level.

Compliance and acceptance of public health measures

Next, we explored which factors were associated with compliance and agreement with public health measures. Of all respondents, 67% reported that they had changed their social behaviour *before* government restrictions were implemented (Fig. 3; Suppl. Table 23). There were significant differences at country level (p<0.001): 93% of Thai respondents reported voluntary pre-restriction behaviour change, followed by the UK (68%) and Malaysia (64%). Slovenian (47%) and Italian respondents (47%) reported the lowest levels of voluntary pre-restriction behaviour change. Overall, 92% of respondents had used sanitizer products and alcohol , 82% avoided physical contact with anyone, and 79% avoided physical contact with only vulnerable groups. In Thailand and Malaysia, 96% and 95% of respondents indicated that they had been using personal protective equipment (PPE; e.g. face masks and gloves), compared to only 33% in UK, 55% in Italy, and 67% in Slovenia (p<0.001). We also asked respondents how much they agreed with quarantine/isolation/social distancing measures and the statement that these are a necessary strategy to help control COVID-19 (Suppl. Table 23). There was a significant difference between countries (p<0.001): agreement with public health measures was highest amongst respondents from Thailand (94%) and lowest amongst those from Slovenia (around 75%).

Overall, fewer male than female respondents changed their social behaviour before the government implemented official restrictions (65% and 70%, respectively, p=0.039; Fig. 3, Suppl. Table 24). At country level, fewer men than women reported changing their social behaviour voluntarily, except in Thailand, where reported changes among men and women were similar (94%/92%, p=0.426). Overall, there were no significant differences between men and women when asked about how much they agreed with public health measures and the statement that these are a necessary strategy to help control COVID-19 (p=0.191; Suppl. Table 24).

When it came to education level, there were no significant differences between respondents with primary/secondary and those with tertiary education regarding voluntary behaviour change before government-imposed restrictions (p=0.369), and agreement with public health measures and the

For peer review only - http://bmjopen.bmj.com/site/about/guidelines.xhtml

statement that these are a necessary strategy to help control COVID-19 (p=0.304; Fig. 3, Suppl. Table 25). Indicators varied at country level.

Overall, 70% of 18-34 year olds and 70% of 35-64 year olds indicated that they had voluntarily changed their behaviour before government restrictions, compared to only 57% of 65+ year olds (p=0.004; Fig. 3, Suppl. Table 26). This trend was similar at country level, except in Italy where 57% of 65+ year olds were most likely to change their behaviour, compared with 44% of 18-34 and 44% of 35-64 year olds. Overall, agreement with voluntary restrictions was similar across age groups (p=0.271; Suppl. Table 26), but fewer 65+ year expressed agreement with restrictions that were government-enforced (p=0.003). Respondents over 65 years old in Slovenia reported the lowest agreement with the statement that quarantine/isolation/social distancing are a necessary strategy to help control COVID-19 (67%), compared to 96% in Thailand and 100% in Malaysia.

Lastly, self-reported levels of understanding of COVID-19 did not significantly affect voluntary change of behaviour (p=0.091), or agreement with public health measures (p=0.688; Suppl. Table 27).

Self-perceived level of understanding of COVID-19

We asked respondents to indicate their perceived level of understanding of COVID-19. Overall, 59% of respondents indicated a ‘high/very high’ level of understanding, 36% reported ‘some’ understanding, and only 5% reported ‘very little/none’ (Fig. 4, Suppl. Table 28). There were significant differences at country level (p<0.001): perceived levels of understanding were highest in Slovenia (66% reported ‘high/very high’, and 30% ‘some’ understanding) and Thailand (63% ‘high/very high’ and 33% ‘some’), and lowest in Italy, with 47% reporting ‘high/very high’, and 50% reporting ‘some’ level of understanding.

To probe for factors associated with perceived level of understanding of COVID-19, we broke down responses by gender, age, education and healthcare worker status (Fig. 4, Suppl. Table 29). Overall, there was no significant difference between men, women and people who identified as other or preferred not to say (p=0.058; Fig. 4, Suppl. Table 29). Age appeared to be a factor, as only 52% of 18-34 year old respondents self-reported ‘high/very high’ understanding compared to 62% of 35-64 year olds and 60% of 65+ year olds (p=0.033). Overall, fewer respondents with primary and secondary education self-reported ‘high/very high’ understanding (56% indicated ‘high/very high’ compared to 66% with tertiary education, p<0.001). Lastly, healthcare worker status was associated with perceived higher understanding (p=0.001). This trend was similar at country level, except for Malaysia, where 49% of healthcare workers reported ‘high/very high’ understanding compared to 52% of non-healthcare workers (p=0.805) (Suppl. Table 29).

Overall, higher levels of perceived understanding of COVID-19 were associated with higher levels of perceived understanding of public health measures (p<0.001; Suppl. Table 30). For example, 88% of

For peer review only - http://bmjopen.bmj.com/site/about/guidelines.xhtml

respondents who self-reported 'high/very high' understanding of COVID-19 and 50% who reported 'some' understanding felt that they had a 'high/very high' level of understanding of public health measures. In contrast, only 27% of respondents who reported 'very little/no' understanding of COVID-19 indicated a high understanding of public health measures.

Information about COVID-19, unclear information and fake news

Throughout the study period, all five countries were running coordinated public information campaigns (Suppl. Fig 1[32 33]). When respondents were asked how they receive/received information about COVID-19 (Suppl. Table 31), most reported traditional mass media (TV, radio, newspapers; 93%), followed by online methods (websites, email; 83%) and social media and messenger apps (79%). When asked about their preferences for receiving information, the top three responses were traditional mass media (78%), government or institution's website (77%), and online (76%). There were no significant differences based on gender (Suppl. Table 32). Fewer respondents over 65 years said that they had used online channels or social media and messenger apps, and they expressed significantly lower preference for these channels too. For example, only 66% of over 65 year olds wanted to receive information online, compared to 78%/79% of the other age groups ($p<0.001$), and only 52% of over 65 year olds expressed preference for social media and messenger apps, compared to 64%/64% ($p=0.005$; Suppl. Table 33). Overall, most respondents with primary/secondary education and those with tertiary education had received information through traditional mass media, and social media/messenger apps (Suppl. Table 34). Fewer respondents with primary/secondary education had used online channels in the form of websites and emails (79% versus 92%, $p<0.001$), and more had received face-to-face information compared to those with tertiary education (43% versus 35%, $p<0.001$; Suppl. Table 34). However, both education level groups indicated that their preferred methods of communication were mass media channels, online methods and government/institutions' websites.

We asked respondents if they had seen unclear or conflicting information about COVID-19 in nine categories relating to infection, symptoms and various public health measures. Overall, between 36-54% of respondents indicated that they had seen such information. Ways to avoid the infection (54%), government support schemes (52%) and testing (51%) were identified as the most unclear areas (Suppl. Table 35). Thailand reported the lowest levels of seeing unclear or conflicting information in most categories (around 35-40%), while respondents in the UK reported the highest levels in most categories (around 55-70%). Overall, those with tertiary education reported significantly higher levels of seeing unclear information than those with primary/secondary education in almost all categories ($p<0.001$) except government support schemes (Suppl. Table 36).

For peer review only - http://bmjopen.bmj.com/site/about/guidelines.xhtml

For peer review only

When asked “Have you come across news about the following COVID-19 topics that seemed fake to you?”, overall 63% of respondents had encountered news on “Coronavirus as an engineered modified virus”, 60% reported seeing “general spread of fear”, and 51% had come across seemingly fake news about “numbers of infected/deceased people”, “home-made recipes to make sanitizer products” and “alternative drugs/cure” (Fig. 5, Suppl. Table 35). Thailand reported the lowest percentages in all ‘fake news’ categories, with a range of 27-42% (Suppl. Table 35). Overall, respondents with tertiary education reported significantly higher levels of seeing ‘fake news’ in all categories compared to those with primary/secondary education (p<0.001; Fig. 5, Suppl. Table 36). For example, only 56% of people with primary/secondary education reported coming across fake news about “coronavirus as an engineered modified virus” versus 79% of those with tertiary education (p<0.001). There did not appear to be an association between self-reported levels of understanding of COVID-19 and seeing unclear/conflicting information or ‘fake news’ (Suppl. Table 37).

Discussion

Our results indicate how public health measures that were in place between 1st May and 30th June 2020 affected a cohort of over 5,000 respondents across five countries, and thus contribute new data and insights to these research areas.

Groups most affected by COVID-19 public health measures

The following factors were associated with a negative economic impact:belonging to the age group 18-24 years or 65 and over, having lower education levels, living in larger households with 5 or more people, having children under 18 in the household, , and having flexible/no income. This suggests that COVID-19 public health measures can have greater negative impacts on already disadvantaged groups. Overall, it appeared that the 35-64 year old age group was less affected than other age groups. Possible explanations for this could be the types of sectors that younger and older people work in (e.g low paid or service industries)[34 35], or for older workers, shielding guidance issued by governments, lower levels of digital skills for remote working[36], or discrimination in the form of ageism[34 37]. There were no significant differences between gender groups in our overall analysis. However, other studies have shown that COVID-19 has had a greater impact on women (e.g. women are more likely to have temporary contracts[38 39] and disproportionally carry the burden of unpaid care[40 41]). A more detailed gender analysis to further break down our survey results is currently underway.

Our results showed that among the countries surveyed, respondents from Thailand reported the most adverse impacts. Thailand is a middle-income country with a large informal economy, and relies heavily on the tourism industry (15% GDP)[42]. Thailand also had a high government stringency index during the period of the study (Fig. 1), which included closure of borders, businesses and nighttime

For peer review only - http://bmjopen.bmj.com/site/about/guidelines.xhtml

curfews[43]. This meant that many informal street vendors and those working in the tourism industry (e.g. tour operators) had no income or lost their jobs.

Overall, about two thirds of respondents were most concerned about the effects of public health measures on their social life, their physical health, and their mental health and wellbeing. These findings resonate with other studies showing the substantial negative impact of COVID-19 restrictions on mental health, wellbeing and social life[44-46].

Self-reported compliance and behavioural changes

A number of quantitative online surveys have examined experiences, knowledge, attitude and perceptions towards COVID-19 and public health measures, at country level[38 47-56], and among different social groups[57-60]. Our findings show that self-reported compliance and behavioural change seemed to differ between countries. For example, respondents in Thailand indicated significantly higher levels of compliance, acceptance of public health measures and voluntary behavioural change compared to other countries. Although our survey was unable to implicate causality, it may contribute to better understanding of why Thailand has the lowest number of COVID cases relative to its population among the countries who took part in the survey[61]. Some of our results with regards to gender and age were similar to trends reported in other studies. For example, results from a Hong Kong study showed that female respondents, and those who reported higher levels of understanding of COVID-19, were more likely to adopt social distancing measures[62]. Similarly, a Chinese study found that men and those with a lower COVID-19 knowledge score were less likely to avoid crowded places or wear a mask outside[51]. Using survey data from 27 countries, Daoust[57] observed that compliance was not higher in older people even though they might be expected to comply more due to being a risk group. Similarly, our data showed that overall and in Malaysia, UK and Slovenia, far fewer respondents over 65 years reported changing their behaviour voluntarily before official restrictions came into place. However, overall, over 80% of respondents in all three age groups expressed agreement when asked if they would comply voluntarily or with government-mandated restrictions (Suppl. Table 26).

For peer review only

Improving COVID-19 communication

Our findings indicated that younger age and lower education levels appeared to be associated with lower self-perceived/subjective levels of understanding of COVID-19. Also, higher self-reported levels of understanding of COVID-19 seemed to be associated with higher self-perceived levels of understanding of public health measures. A recent modelling study suggests that self-imposed public health measures combined with fast spreading of disease awareness in the population can help reduce transmission of the virus[11]. Our findings suggest that specific groups of people, such as those with

For peer review only - http://bmjopen.bmj.com/site/about/guidelines.xhtml

primary/secondary education levels and those 18-34 year old, may benefit most from targeted COVID-19 communication initiatives.

In terms of channels of communications, the three most popular channels across countries were traditional mass media, government or institutional websites, and online media. Similar results emerged from a recent survey carried out in the Netherlands, Germany and Italy[54]. However, respondents in Thailand reported that they preferred to receive information face-to-face, especially those with primary/secondary education. This suggests that in order for communication strategies to be effective, they need to be sensitive to population preferences and tailored to local contextual factors (e.g. levels of connectivity, literacy[63]).

Our survey showed that a significant proportion of the population received conflicting information and news that seemed fake to them, in particular about coronavirus being an engineered modified virus. These findings confirm other reports that misinformation and what has been termed the COVID-19 'infodemic' is widespread[58 64 65]. More efforts should be made to curb misinformation and disinformation, taking into account the needs of different groups[46].

Strengths and limitations

Our online survey enabled us to capture people's experiences and concerns in multiple domains, in five countries, all of which had restrictions in place, during the relatively early stage of the COVID-19 pandemic. To our knowledge, the SEBCOV study was one of the largest international mixed-methods studies conducted on the impact of COVID-19. To maximise the number of respondents and the likelihood of getting honest answers, the survey was completely anonymous. Due to the relatively large sample of respondents in each country, we were able to compare population segments (e.g. men versus women or younger versus older people) in our overall cohort and at country level. We did not aim to obtain nationally representative samples and acknowledge that although we used weighting strategies in our analysis, our results may not be fully representative of the populations in the respective countries. Similarly, there might be differences in the frequency of demographic groups (e.g. 18-24 years old who had been working before COVID-19) between the different countries, which might affect the interpretation of our data at country level. Overall, there was a high proportion of respondents who were healthcare workers (19%), and some variation in this proportion between countries. This may have influenced the country level analysis, in particular in the areas of perceived understanding, compliance/agreement and communication preferences.

Because the survey was online, only people who were literate, had internet access, and had access to computers or smartphones could take part. Due to COVID-19 related restrictions, it was not possible to conduct face-to-face data collection to reach groups who were illiterate in the language of the survey, or who did not have access to online technology. This is likely to have biased our data

For peer review only - http://bmjopen.bmj.com/site/about/guidelines.xhtml

towards more educated and economically advantaged populations. Our study was also subject to response bias and other biases arising from self-reporting and recall. Our study was designed to capture the views and perceptions of respondents on how COVID-19 impacted them socially and economically rather than standard social and economic impact indicator, which are captured by other studies. Similarly, our survey captured perceived level of understanding of COVID-19 and public health measures rather than actual level of understanding. We were able to identify communication needs and preferences of our respondents, which can be used as guidance for organisations running public health communication initiatives. As the media landscapes vary among countries, other factors like freedom of press or the proportion of digital media consumption are likely to influence people's responses. Lastly, as a cross-sectional survey, our data only sheds light on the prevalence of certain phenomena and opinions of respondents but does not imply causality.

The results of the survey reported here form part of a mixed-methods study, which also includes an in-depth qualitative study, the findings of which are currently being analysed and will be published separately. Combined, our results may help explain some of the trends reported in this survey, as well as the differences between countries and social groups. We have also conducted a preliminary analysis of unweighted Thai survey responses during May 2020, which includes more detailed breakdowns by regions within Thailand[66].

Conclusion

Our data confirmed that COVID-19 and public health measures have unequal effects on different countries and different social groups within countries. As such, this study helps to expose some of the social and economic inequalities resulting from COVID-19 and public health measures, and contributes to an important body of research showing that NPIs have a greater impact on those who are socio-economically disadvantaged.. Our findings provide an indication of the social groups who may be most in need of support during pandemics, so that existing social inequalities are not perpetuated and worsened. Lastly, our data can help to inform future strategies for effective communication in order to mitigate the impacts of COVID-19.

Ethics approval

Ethics approval was granted by Oxford Tropical Research Ethics Committee (OxTREC, reference no.520-20), covering all countries; the Faculty of Tropical Medicine Ethics Committee, Thailand (FTMEC, ref: MUTM 2020-031-01); the Medical Research and Ethics Committee (MREC), Ministry of Health Malaysia (MOH), Malaysia, ref: NMRR-20-595-54437 (IIR), and the Universiti Tunku Abdul Rahman (Utar) Scientific and Ethical Review Committee (SERC, ref: (U/SERC/63/2020), Malaysia; and the National Medical Ethics Committee of the Republic of Slovenia (0120-

For peer review only

For peer review only - http://bmjopen.bmj.com/site/about/guidelines.xhtml

237/2020/7). Additional ethics committee approval from Italy was not required for the study to be conducted there.

Acknowledgements

We would like to thank all participants who took part in our study. We also thank the people who helped to test and promote the survey, and the members of the Bangkok Health Research and Ethics Group for their input into the study. We would like to thank the SoNAR-Global Network for the social science expertise in the development of this project and the ODK core team for their input in design of the quantitative survey. Lastly, we would like to thank Prof Nicholas PJ Day, Prof Richard Maude and Dr Tamara Giles-Vernick for critical reading of the manuscript.

Data availability statement

Data are available upon reasonable request. All authors recognize the value of sharing individual level data. We aim to ensure that data generated from all our research are collected, curated, managed and shared in a way that maximizes their benefit. Data underlying this publication are available upon request to the Mahidol Oxford Tropical Medicine Research Uni Data Access Committee at https://www.tropmedres.ac/units/moru-bangkok/bioethics-engagement/data-sharing.

Funding

This research was funded by a Wellcome Trust Institutional Translational Partnership Award (iTPA), Thailand [210559], and a Wellcome Trust Strategic Award [096527]. The Mahidol Oxford Tropical Medicine Research Unit is funded by the Wellcome Trust [106698 and 220221]. This study was also supported by the Sonar-Global project which has received funding from the European Union Horizon 2020 Research and Innovation Program [825671]. The research programme in Slovenia is funded by the Slovenian Research Agency (Javna agencija za raziskovalno dejavnost RS) [P6-0279]

Conflicts of Interest

The authors declare no conflict of interest. The funders had no role in the design of the study; in the collection, analyses, or interpretation of data; in the writing of the manuscript, or in the decision to publish the results.

Contributorship statement

AO and PYC oversaw the whole project and wrote the initial draft of the manuscript. AO, GC, WP, PKC, PC, MS, MLS, TP, NW, SA, BN, SR, NK, DO, RC and PYC developed the survey and

For peer review only - http://bmjopen.bmj.com/site/about/guidelines.xhtml

translations. AO, GC, WP, PC, LS led the project in the UK, Italy, Thailand, Malaysia and Slovenia, respectively. MM and PP conducted the statistical analysis, figures and tables, with critical input from MS, AO and PYC. MLS critically reviewed the manuscript, figures and tables. AO, GC, WP, PKC, PC, MLS, MO, KP, UG, MLS, TP, SA, BN, SR, LS, NK, CRSM, DO, RC and PYC implemented the survey in their respective countries. All authors contributed to the draft paper, and approved the final version of the paper. PYC conceived the project and is the guarantor of the paper.

Transparency declaration

The corresponding author (manuscript guarantor) affirms that this manuscript is an honest, accurate, and transparent account of the study being reported; that no important aspects of the study have been omitted; and that any discrepancies from the study as planned (and, if relevant, registered) have been explained.

Figure legends

Figure 1: Government stringency indices in Thailand, Malaysia, UK, Italy and Slovenia between 1st May – 30th June 2020. A higher score indicates a stricter government response, i.e. 100 = strictest[31].

Figure 2: Bar chart showing how respondents from the following demographic groups were affected economically by COVID-19: at country level (TH = Thailand, MY = Malaysia, UK = United Kingdom, IT = Italy, SI = Slovenia), gender (M = male, F = female, O = Other/prefer not to say); education level (P/S = primary or lower/secondary, T = tertiary); age (18-24 years old, 25-34 years old, 35-64 years old, 65+ years old); household size (1-4 people, ≥5 people); living with children under 18 years (Y = yes, N = no); and type of income (FBP = fixed/benefits/pension, CF = contract/freelance, O = other/no income).

Figure 3: Breakdown of responses to the question "Did you change your social behaviour before the implementation of government restrictions?" by country (TH = Thailand, MY = Malaysia, UK = United Kingdom, IT = Italy, SI = Slovenia) and demographic groups: gender (M = male, F = female, O = other/prefer not to say); education level (P/S = primary or lower/secondary, T = tertiary); age (18-34 years old, 35-64 years old, 65+ years old); self-reported/perceived level of understanding of COVID-19 (H = high/very high/expert level, S = some, N = a little/none at all).

For peer review only - http://bmjopen.bmj.com/site/about/guidelines.xhtml

Figure 4: Breakdown of responses to the question “How would you rate your level understanding of the current quarantine/isolation/social distancing requirements for COVID-19?” Self-reported/perceived level of understanding of COVID-19 ((H = high/very high/expert level, S = some, N = a little/none at all) shown by country (TH = Thailand, MY = Malaysia, UK = United Kingdom, IT = Italy, SI = Slovenia) and demographic groups: gender (M = male, F = female, O = other/prefer not to say); age (18-34 years old, 35-64 years old, 65+ years old); education level (P/S = primary/secondary, T = tertiary); healthcare worker status (HCW = healthcare worker, Non-HCW = non-healthcare worker).

Figure 5: Diagram showing how many survey respondents had come across five ‘fake news’ categories, in response to the question “Have you come across news about the following COVID-19 topics that seemed fake to you?”. Breakdown by country (TH = Thailand, MY = Malaysia, UK = United Kingdom, IT = Italy, SI = Slovenia), gender (M = male, F = female, O = other/prefer not to say), age (18-34 years old, 35-64 years old, 65+ years old), education level (P/S = primary or lower/secondary, T = tertiary), and perceived level of understanding of COVID-19 (H = high/very high/expert level, S = some, N = a little/none at all).

References

1. Guo G, Ye L, Pan K, et al. New insights of emerging SARS-CoV-2: epidemiology, etiology, clinical features, clinical treatment, and prevention. *Frontiers in Cell and Developmental Biology* 2020;8(410) doi: https://doi.org/10.3389/fcell.2020.00410

2. Wang L, Wang Y, Ye D, et al. Review of the 2019 novel coronavirus (SARS-CoV-2) based on current evidence. *International Journal of Antimicrobial Agents* 2020;55(6):105948. doi: https://doi.org/10.1016/j.ijantimicag.2020.105948

3. Yan Y, Shin WI, Pang YX, et al. The first 75 days of novel coronavirus (SARS-CoV-2) outbreak: recent advances, prevention, and treatment. *Int J Environ Res Public Health* 2020;17(7) doi: https://doi.org/10.3390/ijerph17072323

4. World Health Organization. Non-pharmaceutical public health measures for mitigating the risk and impact of epidemic and pandemic influenza. 2019 [Available from: https://www.who.int/influenza/publications/public_health_measures/publication/en/ accessed 9 October 2020.

For peer review only - http://bmjopen.bmj.com/site/about/guidelines.xhtml

5. Centers for Disease Control and Prevention. Non-pharmaceutical interventions (NPIs). 2020 [Available from: https://www.cdc.gov/nonpharmaceutical-interventions/index.html accessed 17th July 2020.

6. Aledort JE, Lurie N, Wasserman J, et al. Non-pharmaceutical public health interventions for pandemic influenza: an evaluation of the evidence base. *BMC Public Health* 2007;7(1):208. doi: https://doi.org/10.1186/1471-2458-7-208

7. Martinez DL, Das TK. Design of non-pharmaceutical intervention strategies for pandemic influenza outbreaks. *BMC Public Health* 2014;14(1):1328. doi: https://doi.org/10.1186/1471-2458-14-1328

8. Ferguson N, Laydon D, Nedjati Gilani G, et al. Report 9: Impact of non-pharmaceutical interventions (NPIs) to reduce COVID19 mortality and healthcare demand. 2020. http://hdl.handle.net/10044/1/77482 (accessed 9 October 2020).

9. Koo JR, Cook AR, Park M, et al. Interventions to mitigate early spread of SARS-CoV-2 in Singapore: a modelling study. *The Lancet Infectious Diseases* 2020;20(6):678-88. doi: https://doi.org/10.1016/S1473-3099(20)30162-6

10. Flaxman S, Mishra S, Gandy A, et al. Estimating the effects of non-pharmaceutical interventions on COVID-19 in Europe. *Nature* 2020;584(7820):257-61. doi: https://doi.org/10.1038/s41586-020-2405-7

11. Teslya A, Pham TM, Godijk NG, et al. Impact of self-imposed prevention measures and short-term government-imposed social distancing on mitigating and delaying a COVID-19 epidemic: a modelling study. *PLoS Med* 2020;17(7):e1003166. doi: https://doi.org/10.1371/journal.pmed.1003166

12. Doung-ngern P, Suphanchaimat R, Panjangampatthana A, et al. Case-control study of use of personal protective measures and risk for severe acute respiratory syndrome coronavirus 2 Infection, Thailand. *Emerging Infectious Diseases* 2020;26(11) doi: https://doi.org/10.3201/eid2611.203003

13. Lewnard JA, Lo NC. Scientific and ethical basis for social-distancing interventions against COVID-19. *Lancet Infect Dis* 2020;20(6):631-33. doi: https://doi.org/10.1016/S1473-3099(20)30190-0

14. Xafis V. 'What is inconvenient for you is life-saving for me': How health inequities are playing out during the COVID-19 pandemic. *Asian Bioethics Review* 2020;12(2):223-34. doi: https://doi.org/10.1007/s41649-020-00119-1

15. Bavel JJV, Baicker K, Boggio PS, et al. Using social and behavioural science to support COVID-19 pandemic response. *Nature Human Behaviour* 2020;4(5):460-71. doi: https://doi.org/10.1038/s41562-020-0884-z

16. Seale H, Dyer CEF, Abdi I, et al. Improving the impact of non-pharmaceutical interventions during COVID-19: examining the factors that influence engagement and the impact on individuals. *BMC Infectious Diseases* 2020;20(1):607. doi: https://doi.org/10.1186/s12879-020-05340-9

17. World Health Organisation. A coordinated global research roadmap: 2019 novel coronavirus 2020 [Available from: https://www.who.int/blueprint/priority-diseases/key-action/Coronavirus_Roadmap_V9.pdf accessed 9 October 2020.

For peer review only - http://bmjopen.bmj.com/site/about/guidelines.xhtml

18. Pan-ngum W, Poomchaichote T, Cuman G, et al. Social, ethical and behavioural aspects of COVID-19 [version 2; peer review: 2 approved]. *Wellcome Open Research* 2020;5(90) doi: https://doi.org/10.12688/wellcomeopenres.15813.2

19. Norton A, De La Horra Gozalo A, Feune de Colombi N, et al. The remaining unknowns: a mixed methods study of the current and global health research priorities for COVID-19. *BMJ Global Health* 2020;5(7):e003306. doi: http://dx.doi.org/10.1136/bmjgh-2020-003306

20. Worldometer. Countries in the world by population (2021) 2021 [Available from: https://www.worldometers.info/world-population/population-by-country/ accessed 8 March 2021.

21. Osterrieder A, Poomchaichote T, Cuman G, et al. Online survey questions: Social, ethical and behavioural aspects of COVID-19 (Version Version 2.0 7 July 2020) 2020 [Available from: http://doi.org/10.5281/zenodo.4049821 accessed 25 September 2020.

22. JISC. Online surveys (formerly BOS). 2020 [Available from: https://www.onlinesurveys.ac.uk/ accessed 13 July 2020.

23. Cheah PY. Thailand “Asia and Africa Programme” Stakeholder Engagement Strategy 2020 - 2025 (Version Version 1, 19 Oct 2019). 2019.

24. National Research Council. Cognitive aspects of survey methodology: building a bridge between disciplines. Washington, DC: The National Academies Press 1984.

25. Padlet. 2020 [Available from: http://padlet.com/ accessed 2 October 2020.

26. The Medical Chamber of Slovenia. 2020 [Available from: https://www.zdravniskazbornica.si/en/medical-chamber-of-slovenia accessed 2 October 2020.

27. Super Poll Thailand. Super Poll Thailand. 2020 [Available from: https://www.superpollthailand.net/ accessed 16 September 2020.

28. Facebook. About boosted posts. 2020 [Available from: https://www.facebook.com/business/help/240208966080581?id=352109282177656 accessed 25 September 2020.

29. Castro FG, Kellison JG, Boyd SJ, et al. A Methodology for Conducting Integrative Mixed Methods Research and Data Analyses. *J Mix Methods Res* 2010;4(4):342-60. doi: 10.1177/1558689810382916 [published Online First: 2010/09/20]

30. Lutz W, Goujon A, Kc S, et al. Demographic and human capital scenarios for the 21st century: 2018 assessment for 201 countries.: Publications Office of the European Union; 2018 [Available from: https://ec.europa.eu/jrc/en/publication/demographic-and-human-capital-scenarios-21st-century-2018-assessment-201-countries accessed 9 October 2020.

31. StataCorp. Stata Statistical Software: Release 15. College Station, TX: StataCorp LLC, 2017.

32. Hale T, Webster S, Petherick A, et al. Oxford COVID-19 Government Response Tracker, Blavatnik School of Government.: Blavatnik School of Government; 2020 [Available from: https://covidtracker.bsg.ox.ac.uk/.

33. Our World in Data. Public information campaigns on the COVID-19 pandemic 2021 [Available from: https://ourworldindata.org/grapher/public-campaigns-covid accessed 8 March 2021.

For peer review only

For peer review only - http://bmjopen.bmj.com/site/about/guidelines.xhtml

34. Alwin RL, Schramm J. Coronavirus' devastating economic impact on workers age 50-plus 2020 [Available from: https://www.aarp.org/politics-society/advocacy/info-2020/coronavirus-economic-impact-older-workers.html accessed 16 September 2020.

35. Business in the Community. COVID-19: economic impact on age in the workplace. 2020 [Available from: https://www.bitc.org.uk/fact-sheet/covid-19-economic-impact-on-age-in-the-workplace/ accessed 16 September 2020.

36. McIvor C. The risk older workers face in the wake of COVID-19. Nesta Blogs 2020 [Available from: https://www.nesta.org.uk/blog/risk-older-workers-face-wake-covid-19/ accessed 13 October 2020.

37. Officer A, Schneiders ML, Wu D, et al. Valuing older people: time for a global campaign to combat ageism. *Bull World Health Organ* 2016;94(10):710-10a. doi: https://doi.org/10.2471/blt.16.184960 [published Online First: 2016/11/16]

38. Eurofound. Living, working and COVID-19: First findings – April 2020 2020 [Available from: https://www.eurofound.europa.eu/publications/report/2020/living-working-and-covid-19-first-findings-april-2020 accessed 13 October 2020.

39. Burki T. The indirect impact of COVID-19 on women. *The Lancet Infectious Diseases* 2020;20(8):904-05. doi: https://doi.org/10.1016/S1473-3099(20)30568-5

40. Anu M, Olivia W, Mekala K, et al. COVID-19 and gender equality: Countering the regressive effects: McKinsey Global Institute; 2020 [Available from: https://www.mckinsey.com/featured-insights/future-of-work/covid-19-and-gender-equality-countering-the-regressive-effects accessed 16 October 2020.

41. Power K. The COVID-19 pandemic has increased the care burden of women and families. *Sustainability: Science, Practice and Policy* 2020;16(1):67-73. doi: https://doi.org/10.1080/15487733.2020.1776561

42. World Bank Group. Thailand Economic Monitor: Thailand in the Time of COVID-19 (English). Washington, D.C. 2020 [Available from: http://documents.worldbank.org/curated/en/456171593190431246/Thailand-Economic-Monitor-Thailand-in-the-Time-of-COVID-19 accessed 13 October 2020.

43. Ministry of Public Health. Thailand's experience in the COVID-19 response 2020 [Available from: https://ddc.moph.go.th/viralpneumonia/eng/file/pub_doc/LDoc9.pdf accessed 13 October 2020.

44. Brooks SK, Webster RK, Smith LE, et al. The psychological impact of quarantine and how to reduce it: rapid review of the evidence. *The Lancet* 2020;395(10227):912-20. doi: https://doi.org/10.1016/S0140-6736(20)30460-8

45. Pierce M, Hope H, Ford T, et al. Mental health before and during the COVID-19 pandemic: a longitudinal probability sample survey of the UK population. *The Lancet Psychiatry* 2020;7(10):883-92. doi: https://doi.org/10.1016/S2215-0366(20)30308-4

46. Social Science in Humanitarian Action Platform. Quarantine in the context of COVID-19. [Available from: https://www.socialscienceinaction.org/resources/february-2020-social-science-humanitarian-action-platform/ accessed 16 September 2020.

For peer review only - http://bmjopen.bmj.com/site/about/guidelines.xhtml

47. Azlan AA, Hamzah MR, Sern TJ, et al. Public knowledge, attitudes and practices towards COVID-19: A cross-sectional study in Malaysia. *PLoS One* 2020;15(5):e0233668. doi: https://doi.org/10.1371/journal.pone.0233668

48. Lin Y, Hu Z, Alias H, et al. Knowledge, attitudes, impact, and anxiety regarding COVID-19 infection among the public in China. *Front Public Health* 2020;8:236. doi: https://doi.org/10.3389/fpubh.2020.00236

49. Roy D, Tripathy S, Kar SK, et al. Study of knowledge, attitude, anxiety & perceived mental healthcare need in Indian population during COVID-19 pandemic. *Asian J Psychiatr* 2020;51:102083. doi: https://doi.org/10.1016/j.ajp.2020.102083 [published Online First: 2020/04/14]

50. Geldsetzer P. Use of rapid online surveys to assess people's perceptions during infectious disease outbreaks: A cross-sectional survey on COVID-19. *J Med Internet Res* 2020;22(4):e18790. doi: https://doi.org/10.2196/18790 [published Online First: 2020/04/03]

51. Zhong BL, Luo W, Li HM, et al. Knowledge, attitudes, and practices towards COVID-19 among Chinese residents during the rapid rise period of the COVID-19 outbreak: a quick online cross-sectional survey. *Int J Biol Sci* 2020;16(10):1745-52. doi: 10.7150/ijbs.45221 [published Online First: 2020/04/01]

52. Bonaccorsi G, Pierri F, Cinelli M, et al. Economic and social consequences of human mobility restrictions under COVID-19. *Proceedings of the National Academy of Sciences of the United States of America* 2020;117(27):15530-35. doi: https://doi.org/10.1073/pnas.2007658117

53. Murphy K, Williamson H, Sargeant E, et al. Why people comply with COVID-19 social distancing restrictions: Self-interest or duty? *Australian and New Zealand Journal of Criminology* 2020;0(0):0004865820954484. doi: https://doi.org/10.1177/0004865820954484

54. Meier K, Glatz T, Guijt MC, et al. Public perspectives on protective measures during the COVID-19 pandemic in the Netherlands, Germany and Italy: A survey study. *PLOS ONE* 2020;15(8):e0236917. doi: https://doi.org/10.1371/journal.pone.0236917

55. Bezerra ACV, Silva C, Soares FRG, et al. Factors associated with people's behavior in social isolation during the COVID-19 pandemic. *Ciencia e Saude Coletiva* 2020;25(suppl 1):2411-21. doi: https://doi.org/10.1590/1413-81232020256.1.10792020 [published Online First: 2020/06/11]

56. Daly M, Ebbinghaus B, Lehner L, et al. Oxford Supertracker: The Global Directory for COVID Policy Trackers and Surveys, Department of Social Policy and Intervention 2020 [Available from: https://supertracker.spi.ox.ac.uk/ accessed 9 October 2020.

57. Daoust JF. Elderly people and responses to COVID-19 in 27 Countries. *PLoS One* 2020;15(7):e0235590. doi: https://doi.org/10.1371/journal.pone.0235590

58. Cuan-Baltazar JY, Muñoz-Perez MJ, Robledo-Vega C, et al. Misinformation of COVID-19 on the internet: infodemiology study. *JMIR Public Health Surveill* 2020;6(2):e18444-e44. doi: https://doi.org/10.2196/18444

59. Biroli P, Bosworth SJ, Della Giusta M, et al. Family Life in Lockdown. Bonn: Institute of Labor Economics (IZA); 2020 [Available from: https://www.iza.org/publications/dp/13398/family-life-in-lockdown accessed 13 October 2020.

For peer review only

For peer review only - http://bmjopen.bmj.com/site/about/guidelines.xhtml

60. Hamadani JD, Hasan MI, Baldi AJ, et al. Immediate impact of stay-at-home orders to control COVID-19 transmission on socioeconomic conditions, food insecurity, mental health, and intimate partner violence in Bangladeshi women and their families: an interrupted time series. *The Lancet Global Health* 2020 doi: https://doi.org/10.1016/S2214-109X(20)30366-1

61. World Health Organisation. WHO Coronavirus Disease (COVID-19) Dashboard 2020 [Available from: https://covid19.who.int/table accessed 16 September 2020.

62. Kwok KO, Li KK, Chan HHH, et al. Community responses during early phase of COVID-19 epidemic, Hong Kong. *Emerg Infect Dis* 2020;26(7):1575-79. doi: https://dx.doi.org/10.3201/eid2607.200500 [published Online First: 2020/04/17]

63. Vaughan E, Tinker T. Effective health risk communication about pandemic influenza for vulnerable populations. *Am J Public Health* 2009;99 Suppl 2(Suppl 2):S324-S32. doi: https://dx.doi.org/10.2105%2FAJPH.2009.162537

64. The Lancet Infectious Diseases. The COVID-19 infodemic. *The Lancet Infectious Diseases* 2020;20(8):875. doi: 10.1016/S1473-3099(20)30565-X

65. Yusof ANM, Muuti MZ, Ariffin LA, et al. Sharing Information on COVID-19: the ethical challenges in the Malaysian setting. *Asian Bioethics Review* 2020;12(3):349-61. doi: https://doi.org/10.1007/s41649-020-00132-4

66. Pan-ngum W, Poomchaichote T, Peerawaranun P, et al. Perspectives on public health interventions in the management of the COVID-19 pandemic in Thailand [version 1; peer review: 1 approved with reservations]. *Wellcome Open Research* 2020;5(245) doi: https://doi.org/10.12688/wellcomeopenres.16293.1

For peer review only

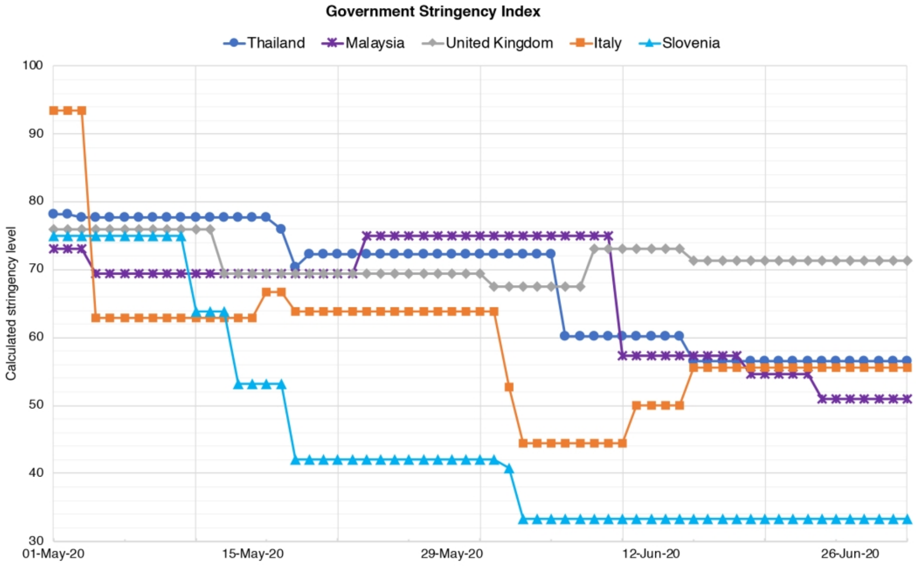

Figure 1: Government stringency indices in Thailand, Malaysia, UK, Italy and Slovenia between 1st May – 30th June 2020. A higher score indicates a stricter government response, i.e. 100 = strictest

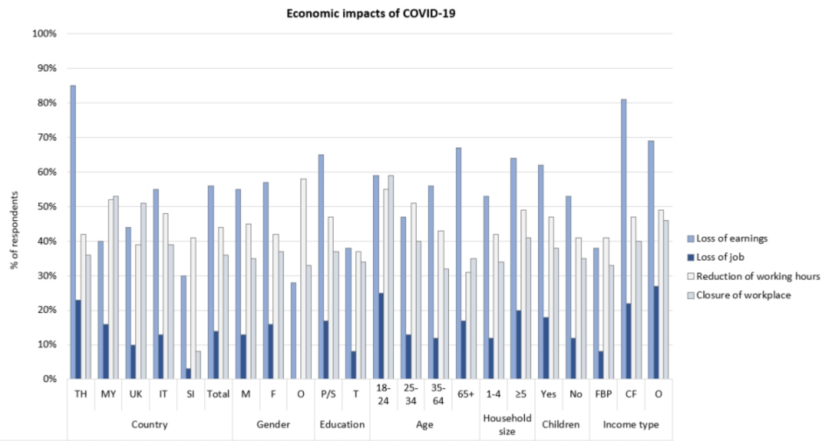

	TH	MY	UK	IT	SI	Total	M	F	O	P/S	T	18-24	25-34	35-64	65+	1-4	≥5	Yes	No	FBP	CF	O
	Country						Gender			Education		Age				Household size		Children		Income type		
Loss of earnings	85%	40%	44%	55%	30%	56%	55%	57%	28%	65%	38%	59%	47%	56%	67%	53%	64%	62%	53%	38%	81%	69%
Loss of job	23%	16%	10%	13%	3%	14%	13%	16%	0%	17%	8%	25%	13%	12%	17%	12%	20%	18%	12%	8%	22%	27%
Reduction of working hours	42%	52%	39%	48%	41%	44%	45%	42%	58%	47%	37%	55%	51%	43%	31%	42%	49%	47%	41%	41%	47%	49%
Closure of workplace	36%	53%	51%	39%	8%	36%	35%	37%	33%	37%	34%	59%	40%	32%	35%	34%	41%	38%	35%	33%	40%	46%

Figure 2. Bar chart showing how respondents from the following demographic groups were affected economically by COVID-19: at country level (TH = Thailand, MY = Malaysia, UK = United Kingdom, IT = Italy, SI = Slovenia), gender (M = male, F = female, O = Other/prefer not to say); education level (P/S = primary or lower/secondary, T = tertiary); age (18-24 years old, 25-34 years old, 35-64 years old, 65+ years old); household size (1-4 people, ≥5 people); living with children under 18 years (Y = yes, N = no); and type of income (FBP = fixed/benefits/pension, CF = contract/freelance, O = other/no income).

122x72mm (300 x 300 DPI)

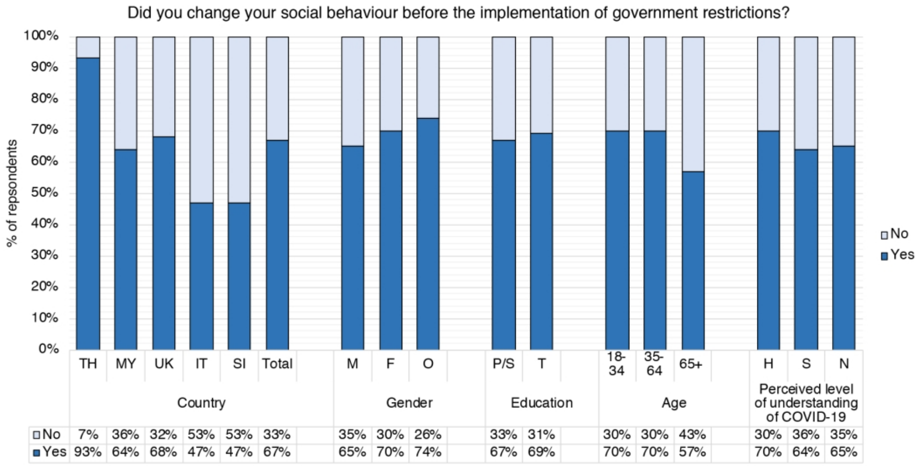

Figure 3: Breakdown of responses to the question “Did you change your social behaviour before the implementation of government restrictions?” by country (TH = Thailand, MY = Malaysia, UK = United Kingdom, IT = Italy, SI = Slovenia) and demographic groups: gender (M = male, F = female, O = other/prefer not to say); education level (P/S = primary or lower/secondary, T = tertiary); age (18-34 years old, 35-64 years old, 65+ years old); self-reported/perceived level of understanding of COVID-19 (H = high/very high/expert level, S = some, N = a little/none at all).

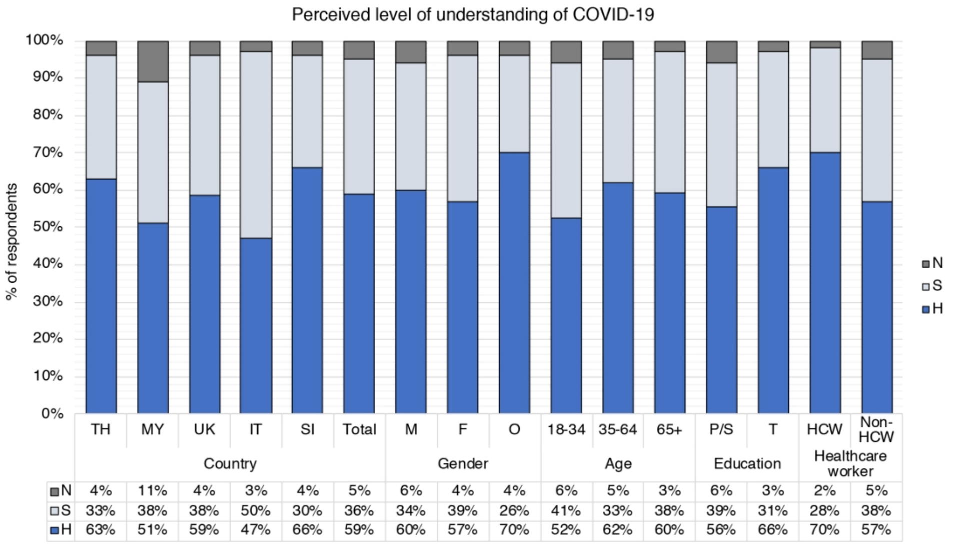

	TH	MY	UK	IT	SI	Total	M	F	O	18-34	35-64	65+	P/S	T	HCW	Non-HCW
	Country						Gender			Age			Education		Healthcare worker	
N	4%	11%	4%	3%	4%	5%	6%	4%	4%	6%	5%	3%	6%	3%	2%	5%
S	33%	38%	38%	50%	30%	36%	34%	39%	26%	41%	33%	38%	39%	31%	28%	38%
H	63%	51%	59%	47%	66%	59%	60%	57%	70%	52%	62%	60%	56%	66%	70%	57%

Figure 4: Breakdown of responses to the question “How would you rate your level understanding of the current quarantine/isolation/social distancing requirements for COVID-19?” Self-reported/perceived level of understanding of COVID-19 ((H = high/very high/expert level, S = some, N = a little/none at all) shown by country (TH = Thailand, MY = Malaysia, UK = United Kingdom, IT = Italy, SI = Slovenia) and demographic groups: gender (M = male, F = female, O = other/prefer not to say); age (18-34 years old, 35-64 years old, 65+ years old); education level (P/S = primary/secondary, T = tertiary); healthcare worker status (HCW = healthcare worker, Non-HCW = non-healthcare worker).

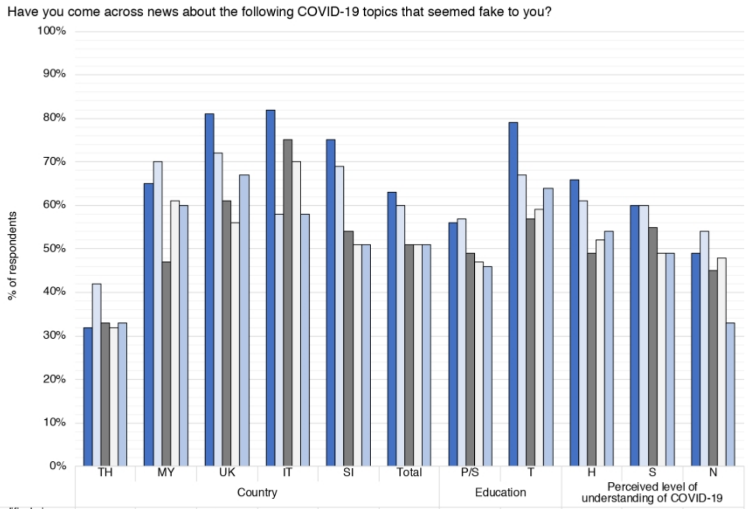

	TH	MY	UK	IT	SI	Total	P/S	T	H	S	N
	Country						Education		Perceived level of understanding of COVID-19		
Coronavirus as an engineered modified virus	32%	65%	81%	82%	75%	63%	56%	79%	66%	60%	49%
General spread of fear	42%	70%	72%	58%	69%	60%	57%	67%	61%	60%	54%
Numbers of infected/deceased people	33%	47%	61%	75%	54%	51%	49%	57%	49%	55%	45%
Home-made recipes to make sanitizer products	32%	61%	56%	70%	51%	51%	47%	59%	52%	49%	48%
Alternative drugs/cure	33%	60%	67%	58%	51%	51%	46%	64%	54%	49%	33%

Figure 5: Diagram showing how many survey respondents had come across five 'fake news' categories, in response to the question "Have you come across news about the following COVID-19 topics that seemed fake to you?". Breakdown by country (TH = Thailand, MY = Malaysia, UK = United Kingdom, IT = Italy, SI = Slovenia), gender (M = male, F = female, O = other/prefer not to say), age (18-34 years old, 35-64 years old, 65+ years old), education level (P/S = primary or lower/secondary, T = tertiary), and perceived level of understanding of COVID-19 (H = high/very high/expert level, S = some, N = a little/none at all).

Supplementary tables for "Economic and social impacts of COVID-19 and public health measures: results from an anonymous online survey in Thailand, Malaysia, the United Kingdom, Italy and Slovenia"

Anne Osterrieder[1,2], Giulia Cuman[3], Wirichada Pan-ngum[1,4], Phaik Kin Cheah[5], Phee-Kheng Cheah[6], Pimnara Peerawaranun[1], Margherita Silan[7], Miha Orazem[8,9], Ksenija Perkovic[10], Urh Groselj[8,11], Mira Leonie Schneiders[1,2,12], Tassawan Poomchaichote[1,13], Naomi Waithira[1,2], Supa-at Asarath[1], Bhensri Naemiratch[1], Supanat Ruangkajorn[1], Lenart Skof[14], Natinee Kulpijit[1], Constance R.S. Mackworth-Young[15], Darlene Ongkili[16], Rita Chanviriyavuth[1], Mavuto Mukaka[1,2], Phaik Yeong Cheah[1,2,12,,13]

[1]Mahidol Oxford Tropical Medicine Research Unit, Faculty of Tropical Medicine, Mahidol University, Bangkok, 10400, Thailand
[2]Centre for Tropical Medicine & Global Health, Nuffield Department of Medicine, University of Oxford, Oxford, UK
[3]Paediatric Ethics Committee; Research Ethics Committee, University Hospital of Padua, Padua, Italy
[4]Department of Tropical Hygiene, Faculty of Tropical Medicine, Mahidol University, Bangkok, 10400, Thailand
[5]Faculty of Arts and Social Science, Universiti Tunku Abdul Rahman, Kampar, Malaysia
[6]Emergency and Trauma Department, Sabah Women and Children's Hospital, Ministry of Health Malaysia, Kota Kinabalu, Malaysia
[7]Department of Statistical Sciences, University of Padua, Padua, Italy
[8]Faculty of Medicine, University of Ljubljana, Ljubljana, Slovenia
[9]Department of Radiation Oncology, Institute of Oncology Ljubljana, Ljubljana, Slovenia
[10]Institute for Social Studies, Science and Research Centre Koper, Koper, Slovenia
[11]Department of Endocrinology, Diabetes and Metabolic Diseases, University Children's Hospital, University Medical Center, University Children's Hospital Ljubljana, Ljubljana, Slovenia
[12]Ethox Centre, Nuffield Department of Population Health, University of Oxford, Oxford, UK
[13]The SoNAR-Global Network
[14]Institute for Philosophical Studies, Science and Research Centre Koper, Koper, Slovenia
[15]Department of Global Health and Development, London School of Hygiene and Tropical Medicine, London, UK
[16]Emergency and Trauma Department, Queen Elizabeth Hospital, Ministry of Health Malaysia, Kota Kinabalu, Malaysia

Corresponding author: Phaik Yeong Cheah, 420/6 Mahidol-Oxford Tropical Medicine 24 Research Unit, Faculty of Tropical Medicine, Rajvithi Road, Bangkok, Thailand, 10400. Email: phaikyeong@tropmedres.ac

Notes

- There are a total of 37 tables in this document. Suppl. Table 1 reports the distribution of the basic demographic variables in the respondent sample (N= number of respondents), followed by unweighted percentages (unweighted %) in brackets. The values displayed in the cells in Suppl. Tables 2-37 show the number of respondents (N) who replied 'yes' to the respective survey categories, followed by weighted percentages (weighted %) in brackets.
- Because of rounding to the nearest integer, percentages do not always add up to 100% exactly.
- For gender, due to small number in the "other/prefer not to say" category, p-values are presented for comparison between the male and female groups only.

Contents

Supplementary tables for "Economic and social impacts of COVID-19 and public health measures: results from an anonymous online survey in Thailand, Malaysia, the United Kingdom, Italy and Slovenia" ... 1

Suppl. Table 1 Distribution of respondents by demographic characteristics and country (unweighted data) ... 4

Suppl. Table 2 Breakdown of economic impacts of COVID-19 and concerns by country ... 5

Suppl. Table 3 Breakdown of economic impacts of COVID-19 and concerns by country and gender ... 6

Suppl. Table 4 Breakdown of economic impacts of COVID-19 and concerns by country and education level ... 7

Suppl. Table 5 Breakdown of economic impacts of COVID-19 and concerns by country and age group ... 8

Suppl. Table 6 Breakdown of economic impacts of COVID-19 and concerns by country and household size ... 10

Suppl. Table 7 Breakdown of economic impacts of COVID-19 and concerns by country and whether or not living with children under 18 ... 11

Suppl. Table 8 Breakdown of economic impacts of COVID-19 and concerns by country and type of income ... 12

Suppl. Table 9 Breakdown of concerns if advised/not allowed physical contact by country ... 13

Suppl. Table 10 Breakdown of concerns if advised/not allowed physical contact by country and gender ... 14

Suppl. Table 11 Breakdown of concerns if advised/not allowed physical contact by country and age group ... 15

Suppl. Table 12 Breakdown of concerns if advised/not allowed physical contact by country and education level ... 16

Suppl. Table 13 Breakdown of concerns if advised/not allowed physical contact by country and household size ... 17

Suppl. Table 14 Breakdown of concerns if advised/not allowed physical contact by country and whether or not living with children under 18 ... 17

Suppl. Table 15 Breakdown of concerns if advised/not allowed physical contact by country and income type ... 19

Suppl. Table 16 Breakdown of maximum number of days that people thought they could cope by country ... 20

Suppl. Table 17 Breakdown of maximum number of days that people thought they could cope by country and gender ... 21

Suppl. Table 18 Breakdown of maximum number of days that people thought they could cope by country and age group ... 22

Suppl. Table 19 Breakdown of maximum number of days that people thought they could cope by country and household size ... 23

Suppl. Table 20 Breakdown of maximum number of days that people thought they could cope by country and whether or not living with children under 18 ... 24

Suppl. Table 21 Breakdown of maximum number of days that people thought they could cope by country and education level ... 25

Suppl. Table 22 Breakdown of maximum number of days that people thought they could cope by country and type of income ... 26

Suppl. Table 23 Breakdown of behavioural changes and acceptance of government public health measures by country ... 27

Suppl. Table 24 Breakdown of behavioural changes and acceptance of government public health measures by country and gender ... 28

Suppl. Table 25 Breakdown of behavioural changes and acceptance of government public health measures by country and education level ... 30

Suppl. Table 26 Breakdown of behavioural changes and acceptance of government public health measures by age group ... 31

For peer review only - http://bmjopen.bmj.com/site/about/guidelines.xhtml

Suppl. Table 27 Breakdown of behavioural changes and acceptance of government public health measures by self-reported level of understanding of COVID-19 33
Suppl. Table 28 Breakdown of self-reported level of understanding of COVID-19 by country 35
Suppl. Table 29 Breakdown of self-reported level of understanding of COVID-19 by demographic characteristics 36
Suppl. Table 30 Breakdown of self-reported understanding of public health measures by self-reported level of understanding of COVID-19 37
Suppl. Table 31 What were the three most common ways people received communication on COVID-19, and what are the three most preferred ways to receive COVID-19 communications? Breakdown by country 38
Suppl. Table 32 What were the three most common ways people received communications on COVID-19, and what are the three most preferred ways to receive COVID-19 communications? Breakdown by country and gender 39
Suppl. Table 33 What were the three most common ways people received communications on COVID-19, and what are the three most preferred ways to receive COVID-19 communications? Breakdown by country and age group 40
Suppl. Table 34 What were the three most common ways people received communications on COVID-19, and what are the three most preferred ways to receive COVID-19 communications? Breakdown by country and education level 41
Suppl. Table 35 Most prevalent topic areas with unclear or conflicting COVID-19 information, and most prevalent 'fake news', breakdown by country 42
Suppl. Table 36 Most prevalent topic areas with unclear or conflicting COVID-19 information, and most prevalent 'fake news', breakdown by country and education level 43
Suppl. Table 37 Most prevalent topic areas with unclear or conflicting COVID-19 information, and most prevalent 'fake news', breakdown by country and self-reported level of understanding of COVID-19 44

For peer review only

Suppl. Table 1 Distribution of respondents by demographic characteristics and country (unweighted data)

Variable and categories	Thailand (N=1,476, 29%)	Malaysia (N=827, 16%)	UK (N=1,009, 20%)	Italy (N=712, 14%)	Slovenia (N=1,034, 20%)	Total (N=5,058)
**Gender**						
Male	704 (48)	298 (36)	426 (42)	222 (31)	366 (35)	2,016 (40)
Female	766 (52)	525 (63)	572 (57)	490 (69)	662 (64)	3,015 (60)
Other/prefer not to say	6 (0)	4 (0)	11 (1)	0 (0)	6 (1)	27 (1)
**Age (years)**						
18-24	83 (6)	139 (17)	54 (5)	75 (11)	62 (6)	413 (8)
25-34	140 (9)	211 (26)	86 (9)	197 (28)	246 (24)	880 (17)
35-64	1,152 (78)	442 (53)	616 (61)	383 (54)	676 (65)	3,269 (65)
65+	101 (7)	35 (4)	253 (25)	57 (8)	50 (5)	496 (10)
**Education level**						
Primary or lower/ secondary	909 (62)	82 (10)	247 (24)	217 (30)	202 (20)	1,657 (33)
Tertiary	567 (38)	745 (90)	762 (76)	495 (70)	832 (80)	3,401 (67)
**Household structure**						
Living alone	134 (9)	74 (9)	206 (20)	106 (15)	97 (9)	617 (12)
Living only with partner/spouse	173 (12)	95 (11)	391 (39)	192 (27)	210 (20)	1,061 (21)
Living with partner/spouse and children; living as single parent with children	847 (57)	312 (38)	260 (26)	188 (26)	518 (50)	2,125 (42)
Living with other relatives/non-relatives/other	322 (22)	346 (42)	152 (15)	226 (32)	209 (20)	1,255 (25)
**Household size**						
1	107 (7)	68 (8)	222 (22)	106 (15)	128 (12)	631 (12)
2	171 (12)	121 (15)	439 (44)	230 (32)	220 (21)	1,181 (23)
3-4	760 (51)	305 (37)	300 (30)	323 (45)	479 (46)	2,167 (43)
≥5	438 (30)	333 (40)	48 (5)	53 (7)	207 (20)	1,079 (21)
**Type of income**						
Fixed salary/benefits/pension	546 (37)	524 (63)	705 (70)	347 (49)	847 (82)	2,969 (59)
Contract and freelance	849 (58)	158 (19)	227 (22)	244 (34)	103 (10)	1,581 (31)
Other/no income	81 (5)	145 (18)	77 (8)	121 (17)	84 (8)	508 (10)
**Living with children under 18**	664 (45)	346 (42)	186 (18)	144 (20)	497 (48)	1,837 (36)
**Living with vulnerable group***	457 (31)	230 (28)	367 (36)	151 (21)	280 (27)	1,485 (29)
**Healthcare provider/worker****	239 (16)	213 (26)	118 (12)	64 (9)	341 (33)	975 (19)

Values in cells are n (%)
* Persons aged 70 or older; pregnant woman; people with serious health conditions
** Included respondents who were not working before COVID-19

Suppl. Table 2 Breakdown of economic impacts of COVID-19 and concerns by country

Values in cells are n (weighted %) of respondents who replied 'yes'.

Variable and categories	Thailand	Malaysia	UK	Italy	Slovenia	Total	P-value
**If you were working before COVID-19, has COVID-19 created any inconvenience for you?**	N=1,255	N=613	N=630	N=526	N=929	N=3,953	
Loss of earnings	(N=1,248) 1,012 (85)	(N=556) 155 (40)	(N=584) 226 (44)	(N=496) 260 (55)	(N=867) 219 (30)	(N=3,751) 1,872 (56)	<0.001
Loss of job	(N=1,191) 233 (23)	(N=532) 44 (16)	(N=551) 51 (10)	(N=471) 59 (13)	(N=832) 15 (3)	(N=3,577) 402 (14)	<0.001
Reduction of working hours	(N=1,210) 492 (42)	(N=546) 228 (52)	(N=570) 201 (39)	(N=484) 233 (48)	(N=862) 319 (41)	(N=3,672) 1,473 (44)	0.107
Closure of workplace	(N=1,207) 425 (36)	(N=562) 289 (53)	(N=591) 296 (51)	(N=484) 167 (39)	(N=833) 63 (8)	(N=3,677) 1,240 (36)	<0.001
**Did you continue to work during COVID-19?**	(N=1,255) 1,019 (79)	(N=613) 532 (70)	(N=630) 460 (70)	(N=526) 388 (67)	(N=929) 768 (79)	(N=3,953) 3,167 (75)	0.011
**What are/were your concerns if advised no physical contact/not allowed to go out/allowed to go out only for essential needs?**	N=1,476	N=827	N=1,009	N=712	N=1,034	N=5,058	
Financial (e.g. loss of income, loss of job)	(N=1,466) 1,215 (86)	(N=775) 419 (60)	(N=950) 271 (32)	(N=678) 315 (41)	(N=1,015) 302 (28)	(N=4,884) 2,522 (53)	<0.001
Professional/ career progression	(N=1,414) 607 (42)	(N=759) 418 (52)	(N=942) 198 (24)	(N=670) 224 (22)	(N=1,001) 219 (17)	(N=4,786) 1,666 (32)	<0.001

For peer review only

Suppl. Table 3 Breakdown of economic impacts of COVID-19 and concerns by country and gender

M = male; F = female; O = other/prefer not to say. Values in cells are n (weighted %) of respondents who replied 'yes'.

Variable and categories	Thailand			Malaysia			UK			Italy			Slovenia			Total			
**Gender**	**M**	**F**	**O**	**M**	**F**	**O**	**M**	**F**	**O**	**M**	**F**	**O**	**M**	**F**	**O**	**M**	**F**	**O**	**P-value (for total M vs F)**
**If you were working before COVID-19, has COVID-19 created any inconvenience for you?**	N=606	N=645	N=4	N=230	N=380	N=3	N=261	N=363	N=6	N=184	N=342	N=0	N=332	N=591	N=6	N=1,613	N=2,321	N=19	
Loss of earnings	(N=604) 508 (83)	(N=640) 502 (86)	(N=4) 2 (50)	(N=210) 75 (42)	(N=343) 80 (37)	(N=3) 0 (0)	(N=245) 97 (45)	(N=333) 128 (43)	(N=6) 1 (17)	(N=177) 99 (54)	(N=319) 161 (57)		(N=314) 82 (29)	(N=548) 135 (31)	(N=5) 2 (40)	(N=1,550) 861 (55)	(N=2,183) 1,006 (57)	(N=18) 5 (28)	0.531
Loss of job	(N=576) 104 (20)	(N=611) 129 (25)	(N=4) 0 (0)	(N=202) 17 (18)	(N=327) 27 (15)	(N=3) 0 (0)	(N=233) 21 (19)	(N=313) 30 (11)	(N=5) 0 (0)	(N=168) 19 (10)	(N=303) 40 (17)		(N=301) 3 (1)	(N=526) 12 (4)	(N=5) 0 (0)	(N=1,480) 164 (13)	(N=2,080) 238 (16)	(N=17) 0 (0)	0.157
Reduction of working hours	(N=586) 225 (41)	(N=620) 265 (43)	(N=4) 2 (50)	(N=205) 85 (57)	(N=338) 141 (46)	(N=3) 2 (67)	(N=240) 90 (41)	(N=324) 107 (37)	(N=6) 4 (67)	(N=174) 94 (52)	(N=310) 139 (43)		(N=315) 128 (44)	(N=541) 188 (39)	(N=6) 3 (50)	(N=1,520) 622 (45)	(N=2,133) 840 (42)	(N=19) 11 (58)	0.179
Closure of workplace	(N=581) 194 (35)	(N=622) 231 (37)	(N=4) 0 (0)	(N=208) 109 (48)	(N=351) 178 (60)	(N=3) 2 (67)	(N=251) 124 (50)	(N=334) 169 (51)	(N=6) 3 (50)	(N=172) 65 (38)	(N=312) 102 (41)		(N=302) 19 (7)	(N=526) 43 (9)	(N=5) 1 (20)	(N=1,514) 511 (35)	(N=2,145) 723 (37)	(N=18) 6 (33)	0.365
Did you continue to work during COVID-19?	(N=606) 508 (84)	(N=645) 507 (75)	(N=4) 4 (100)	(N=230) 198 (67)	(N=380) 332 (73)	(N=3) 2 (67)	(N=261) 198 (72)	(N=363) 258 (67)	(N=6) 4 (67)	(N=184) 144 (74)	(N=342) 244 (60)		(N=332) 295 (85)	(N=591) 469 (74)	(N=6) 4 (67)	(N=1,613) 1,343 (78)	(N=2,321) 1,810 (71)	(N=19) 14 (74)	0.010
**What are/were your concerns if advised no physical contact/not allowed to go out/allowed to go out only for essential needs?**	N=704	N=766	N=6	N=298	N=525	N=4	N=261	N=363	N=6	N=222	N=490	N=0	N=366	N=662	N=6	N=2,016	N=3,015	N=27	
Financial	(N=700) 592 (85)	(N=760) 619 (86)	(N=6) 4 (67)	(N=279) 155 (62)	(N=492) 261 (59)	(N=4) 3 (75)	(N=411) 113 (34)	(N=529) 154 (31)	(N=10) 4 (40)	(N=214) 113 (44)	(N=464) 202 (38)		(N=361) 110 (27)	(N=648) 188 (29)	(N=6) 4 (67)	(N=1,965) 1,083 (54)	(N=2,893) 1,424 (53)	(N=26) 15 (58)	0.806
Professional/ career progression	(N=675) 278 (41)	(N=733) 326 (42)	(N=6) 3 (50)	(N=270) 137 (53)	(N=485) 279 (51)	(N=4) 2 (50)	(N=409) 84 (26)	(N=523) 108 (22)	(N=10) 6 (60)	(N=211) 92 (26)	(N=459) 132 (18)		(N=354) 77 (14)	(N=641) 141 (19)	(N=6) 1 (17)	(N=1,919) 668 (32)	(N=2,841) 986 (31)	(N=26) 12 (46)	0.597

Suppl. Table 4 Breakdown of economic impacts of COVID-19 and concerns by country and education level

P/S = primary or lower/secondary education; T = tertiary education. Values in cells are n (weighted %) of respondents who replied ‘yes’.

**Variable and categories**	**Thailand**		**Malaysia**		**UK**		**Italy**		**Slovenia**		**Total**		
**Education level**	**P/S**	**T**	**P/S**	**T**	**P/S**	**T**	**P/S**	**T**	**P/S**	**T**	**P/S**	**T**	**P-value (for total)**
**If you were working before COVID-19, has COVID-19 created any inconvenience for you?**	N=785	N=470	N=53	N=560	N=122	N=508	N=136	N=390	N=160	N=769	N=1,256	N=2,697	
Loss of earnings	(N=780)<br>725 (90)	(N=468)<br>287 (62)	(N=50)<br>21 (42)	(N=506)<br>134 (28)	(N=116)<br>55 (58)	(N=468)<br>171 (34)	(N=126)<br>75 (58)	(N=370)<br>185 (52)	(N=150)<br>56 (36)	(N=717)<br>163 (24)	(N=1,222)<br>932 (65)	(N=2,529)<br>940 (38)	<0.001
Loss of job	(N=744)<br>164 (24)	(N=447)<br>69 (16)	(N=50)<br>9 (19)	(N=482)<br>35 (7)	(N=108)<br>12 (13)	(N=443)<br>39 (9)	(N=123)<br>18 (14)	(N=348)<br>41 (12)	(N=140)<br>7 (4)	(N=692)<br>8 (1)	(N=1,165)<br>210 (17)	(N=2,412)<br>192 (8)	<0.001
Reduction of working hours	(N=762)<br>332 (43)	(N=448)<br>160 (37)	(N=48)<br>25 (55)	(N=498)<br>203 (40)	(N=110)<br>42 (49)	(N=460)<br>159 (32)	(N=125)<br>63 (47)	(N=359)<br>170 (49)	(N=144)<br>72 (46)	(N=718)<br>247 (35)	(N=1,189)<br>534 (47)	(N=2,483)<br>939 (37)	<0.001
Closure of workplace	(N=753)<br>262 (36)	(N=454)<br>163 (37)	(N=48)<br>28 (55)	(N=514)<br>261 (49)	(N=116)<br>51 (48)	(N=475)<br>245 (52)	(N=130)<br>59 (44)	(N=354)<br>108 (31)	(N=137)<br>14 (8)	(N=696)<br>49 (7)	(N=1,184)<br>414 (37)	(N=2,493)<br>826 (34)	0.180
Did you continue to work during COVID-19?	(N=785)<br>613 (78)	(N=470)<br>406 (86)	(N=53)<br>34 (65)	(N=560)<br>498 (90)	(N=122)<br>73 (59)	(N=508)<br>387 (77)	(N=136)<br>75 (59)	(N=390)<br>313 (79)	(N=160)<br>115 (74)	(N=769)<br>653 (85)	(N=1,256)<br>910 (71)	(N=2,697)<br>2,257 (83)	<0.001
**What are/were your concerns if advised no physical contact/not allowed to go out/allowed to go out only for essential needs?**	N=909	N=567	N=82	N=745	N=247	N=762	N=217	N=495	N=202	N=832	N=1,657	N=3,401	
Financial	(N=904)<br>828 (89)	(N=562)<br>387 (68)	(N=75)<br>46 (62)	(N=700)<br>373 (55)	(N=232)<br>64 (34)	(N=718)<br>207 (31)	(N=205)<br>96 (39)	(N=473)<br>219 (46)	(N=193)<br>71 (29)	(N=822)<br>231 (27)	(N=1,609)<br>1,105 (59)	(N=3,275)<br>1,417 (41)	<0.001
Professional/ career progression	(N=865)<br>326 (39)	(N=549)<br>281 (54)	(N=72)<br>36 (50)	(N=687)<br>382 (59)	(N=228)<br>21 (16)	(N=714)<br>177 (31)	(N=198)<br>42 (15)	(N=472)<br>182 (37)	(N=192)<br>37 (13)	(N=809)<br>182 (22)	(N=1,555)<br>462 (30)	(N=3,231)<br>1,204 (36)	0.004

For peer review only - http://bmjopen.bmj.com/site/about/guidelines.xhtml

Suppl. Table 5 Breakdown of economic impacts of COVID-19 and concerns by country and age group

Values in cells are n (weighted %) of respondents who replied ‘yes’.

Suppl. Table 5a Breakdown of economic impacts of COVID-19 and concerns by country and age group

Values in cells are n (weighted %) of respondents who replied ‘yes’.

**Variable and categories**	**Thailand**				**Malaysia**				**UK**			
**Age group**	**18-24**	**25-34**	**35-64**	**65+**	**18-24**	**25-34**	**35-64**	**65+**	**18-24**	**25-34**	**35-64**	**65+**
**If you were working before COVID-19, has COVID-19 created any inconvenience for you?**	N=35	N=120	N=1,027	N=73	N=43	N=176	N=378	N=16	N=34	N=70	N=466	N=60
Loss of earnings	(N=34)<br>28 (61)	(N=120)<br>75 (76)	(N=1,021)<br>851 (89)	(N=73)<br>58 (80)	(N=41)<br>15 (54)	(N=166)<br>33 (38)	(N=334)<br>98 (34)	(N=15)<br>9 (57)	(N=31)<br>16 (71)	(N=69)<br>16 (38)	(N=427)<br>168 (41)	(N=57)<br>26 (46)
Loss of job	(N=34)<br>15 (32)	(N=114)<br>21 (25)	(N=972)<br>183 (20)	(N=71)<br>14 (22)	(N=40)<br>10 (42)	(N=164)<br>12 (14)	(N=314)<br>20 (10)	(N=14)<br>2 (13)	(N=30)<br>5 (19)	(N=68)<br>5 (8)	(N=401)<br>35 (9)	(N=52)<br>6 (8)
Reduction of working hours	(N=34)<br>18 (42)	(N=113)<br>55 (54)	(N=991)<br>401 (42)	(N=72)<br>18 (23)	(N=38)<br>18 (44)	(N=168)<br>67 (75)	(N=325)<br>136 (49)	(N=15)<br>7 (50)	(N=32)<br>17 (74)	(N=68)<br>14 (27)	(N=416)<br>145 (36)	(N=54)<br>25 (45)
Closure of workplace	(N=34)<br>21 (60)	(N=117)<br>45 (42)	(N=984)<br>340 (35)	(N=72)<br>19 (24)	(N=40)<br>29 (65)	(N=167)<br>64 (51)	(N=340)<br>184 (48)	(N=15)<br>12 (83)	(N=32)<br>19 (75)	(N=68)<br>38 (52)	(N=434)<br>215 (49)	(N=57)<br>24 (44)
Did you continue to work during COVID-19?	(N=35)<br>19 (70)	(N=120)<br>101 (82)	(N=1,027)<br>838 (80)	(N=73)<br>61 (81)	(N=43)<br>32 (40)	(N=176)<br>163 (67)	(N=378)<br>330 (82)	(N=16)<br>7 (43)	(N=34)<br>19 (33)	(N=70)<br>60 (85)	(N=466)<br>346 (72)	(N=60)<br>35 (56)
**What are/were your concerns if advised no physical contact/not allowed to go out/allowed to go out only for essential needs?**	N=83	N=140	N=1,152	N=101	N=139	N=211	N=442	N=35	N=54	N=86	N=616	N=253
Financial	(N=81)<br>59 (69)	(N=139)<br>102 (84)	(N=1,145)<br>985 (89)	(N=101)<br>69 (78)	(N=134)<br>83 (51)	(N=204)<br>115 (82)	(N=408)<br>211 (64)	(N=29)<br>10 (42)	(N=52)<br>30 (62)	(N=82)<br>29 (37)	(N=581)<br>195 (35)	(N=235)<br>17 (6)
Professional/ career progression	(N=82)<br>58 (61)	(N=133)<br>68 (48)	(N=1,106)<br>452 (39)	(N=93)<br>29 (31)	(N=130)<br>96 (64)	(N=206)<br>142 (68)	(N=395)<br>173 (43)	(N=28)<br>7 (26)	(N=51)<br>40 (64)	(N=83)<br>36 (40)	(N=572)<br>118 (17)	(N=236)<br>4 (2)

For peer review only

Suppl. Table 5b Breakdown of economic impacts of COVID-19 and concerns by country and age group

Values in cells are n (weighted %) of respondents who replied ‘yes’.

**Variable and categories**	**Italy**				**Slovenia**				**Total**				
**Age group**	**18-24**	**25-34**	**35-64**	**65+**	**18-24**	**25-34**	**35-64**	**65+**	**18-24**	**25-34**	**35-64**	**65+**	**P-value (for total)**
**If you were working before COVID-19, has COVID-19 created any inconvenience for you?**	N=31	N=159	N=324	N=12	N=37	N=222	N=646	N=24	N=180	N=747	N=2,841	N=185	
Loss of earnings	(N=31)<br>24 (67)	(N=154)<br>73 (47)	(N=299)<br>155 (54)	(N=12)<br>8 (87)	(N=37)<br>15 (45)	(N=216)<br>52 (25)	(N=595)<br>144 (29)	(N=19)<br>8 (39)	(N=174)<br>98 (59)	(N=725)<br>249 (47)	(N=2,676)<br>1,416 (56)	(N=176)<br>109 (67)	0.044
Loss of job	(N=30)<br>4 (10)	(N=151)<br>18 (12)	(N=282)<br>35 (12)	(N=8)<br>2 (42)	(N=37)<br>2 (5)	(N=211)<br>4 (2)	(N=567)<br>9 (3)	(N=17)<br>0 (0)	(N=171)<br>36 (25)	(N=708)<br>60 (13)	(N=2,536)<br>282 (12)	(N=162)<br>24 (17)	0.053
Reduction of working hours	(N=30)<br>18 (58)	(N=152)<br>69 (47)	(N=292)<br>143 (50)	(N=10)<br>3 (16)	(N=36)<br>22 (67)	(N=213)<br>77 (40)	(N=593)<br>212 (39)	(N=20)<br>8 (38)	(N=170)<br>93 (55)	(N=714)<br>282 (51)	(N=2,617)<br>1,037 (43)	(N=171)<br>61 (31)	0.016
Closure of workplace	(N=31)<br>22 (66)	(N=154)<br>54 (43)	(N=289)<br>85 (32)	(N=10)<br>6 (86)	(N=36)<br>8 (25)	(N=210)<br>19 (12)	(N=570)<br>35 (6)	(N=17)<br>1 (3)	(N=173)<br>99 (59)	(N=716)<br>220 (40)	(N=2,617)<br>859 (32)	(N=171)<br>62 (35)	<0.001
Did you continue to work during COVID-19?	(N=31)<br>16 (66)	(N=159)<br>118 (71)	(N=324)<br>250 (70)	(N=12)<br>4 (13)	(N=37)<br>22 (56)	(N=222)<br>187 (83)	(N=646)<br>540 (81)	(N=24)<br>19 (72)	(N=180)<br>108 (54)	(N=747)<br>629 (78)	(N=2,841)<br>2,304 (78)	(N=185)<br>126 (68)	<0.001
**What are/were your concerns if advised no physical contact/not allowed to go out/allowed to go out only for essential needs?**	N=75	N=197	N=383	N=57	N=62	N=246	N=676	N=50	N=413	N=880	N=3,269	N=496	
Financial	(N=75)<br>36 (46)	(N=195)<br>102 (52)	(N=356)<br>168 (48)	(N=52)<br>9 (20)	(N=62)<br>26 (45)	(N=243)<br>66 (24)	(N=664)<br>205 (36)	(N=46)<br>5 (4)	(N=404)<br>234 (57)	(N=863)<br>414 (60)	(N=3,154)<br>1,764 (58)	(N=463)<br>110 (30)	<0.001
Professional/ career progression	(N=75)<br>25 (30)	(N=194)<br>97 (48)	(N=350)<br>99 (23)	(N=51)<br>3 (1)	(N=61)<br>28 (44)	(N=242)<br>80 (29)	(N=654)<br>109 (15)	(N=44)<br>2 (1)	(N=399)<br>247 (57)	(N=858)<br>423 (48)	(N=3,077)<br>951 (28)	(N=452)<br>45 (11)	<0.001

Suppl. Table 6 Breakdown of economic impacts of COVID-19 and concerns by country and household size

Values in cells are n (weighted %) of respondents who replied 'yes'.

Variable and categories	Thailand		Malaysia		UK		Italy		Slovenia		Total		
**Household size (number of persons in the household)**	**1-4**	**≥5**	**1-4**	**≥5**	**1-4**	**≥5**	**1-4**	**≥5**	**1-4**	**≥5**	**1-4**	**≥5**	**P-value (for total)**
**If you were working before COVID-19, has COVID-19 created any inconvenience for you?**	N=862	N=393	N=376	N=237	N=592	N=38	N=491	N=35	N=743	N=186	N=3,064	N=889	
Loss of earnings	(N=857) 685 (84)	(N=391) 327 (85)	(N=348) 97 (36)	(N=208) 58 (45)	(N=547) 213 (43)	(N=37) 13 (51)	(N=464) 243 (55)	(N=32) 17 (68)	(N=693) 181 (30)	(N=174) 38 (32)	(N=2,909) 1,419 (53)	(N=842) 453 (64)	0.003
Loss of job	(N=821) 150 (21)	(N=370) 83 (26)	(N=335) 22 (13)	(N=197) 22 (22)	(N=515) 49 (9)	(N=36) 2 (21)	(N=442) 59 (14)	(N=29) 0 (0)	(N=666) 13 (3)	(N=166) 2 (3)	(N=2,779) 293 (12)	(N=798) 109 (20)	0.005
Reduction of working hours	(N=835) 335 (41)	(N=375) 157 (44)	(N=346) 143 (50)	(N=200) 85 (56)	(N=532) 185 (37)	(N=38) 16 (59)	(N=454) 216 (47)	(N=30) 17 (68)	(N=686) 247 (39)	(N=176) 72 (50)	(N=2,853) 1,126 (42)	(N=819) 347 (49)	0.037
Closure of workplace	(N=832) 278 (34)	(N=375) 147 (40)	(N=349) 168 (45)	(N=213) 121 (67)	(N=553) 280 (51)	(N=38) 16 (42)	(N=451) 153 (38)	(N=33) 14 (55)	(N=666) 51 (8)	(N=167) 12 (8)	(N=2,851) 930 (34)	(N=826) 310 (41)	0.057
Did you continue to work during COVID-19?	(N=862) 703 (78)	(N=393) 316 (81)	(N=376) 330 (72)	(N=237) 202 (67)	(N=592) 430 (71)	(N=38) 30 (58)	(N=491) 363 (67)	(N=35) 25 (63)	(N=743) 612 (79)	(N=186) 156 (79)	(N=3,064) 2,438 (75)	(N=889) 729 (75)	0.873
**What are/were your concerns if advised no physical contact/not allowed to go out/allowed to go out only for essential needs?**	N=1,038	N=438	N=494	N=333	N=961	N=48	N=659	N=53	N=827	N=207	N=3,979	N=1,079	
Financial	(N=1,031) 860 (87)	(N=435) 355 (82)	(N=461) 234 (59)	(N=314) 185 (62)	(N=906) 258 (32)	(N=44) 13 (34)	(N=627) 285 (40)	(N=51) 30 (66)	(N=813) 249 (26)	(N=202) 53 (37)	(N=3,838) 1,886 (50)	(N=1,046) 636 (66)	<0.001
Professional/ career progression	(N=996) 411 (38)	(N=418) 196 (49)	(N=454) 228 (47)	(N=305) 190 (59)	(N=899) 187 (23)	(N=43) 11 (32)	(N=620) 200 (21)	(N=50) 24 (46)	(N=799) 180 (16)	(N=202) 39 (20)	(N=3,768) 1,206 (28)	(N=1,018) 460 (46)	<0.001

For peer review only - http://bmjopen.bmj.com/site/about/guidelines.xhtml

Suppl. Table 7 Breakdown of economic impacts of COVID-19 and concerns by country and whether or not living with children under 18

Y = living with children under 18; N = not living with children under 18. Values in cells are n (weighted %) of respondents who replied 'yes'.

Variable and categories	Thailand		Malaysia		UK		Italy		Slovenia		Total		
**Living with children under 18**	**Y**	**N**	**Y**	**N**	**Y**	**N**	**Y**	**N**	**Y**	**N**	**Y**	**N**	**P-value (for total)**
**If you were working before COVID-19, has COVID-19 created any inconvenience for you?**	N=546	N=709	N=276	N=337	N=158	N=472	N=112	N=414	N=462	N=467	N=1,554	N=2,399	
Loss of earnings	(N=545) 483 (91)	(N=703) 529 (79)	(N=239) 66 (44)	(N=317) 89 (37)	(N=144) 52 (46)	(N=440) 174 (43)	(N=98) 58 (61)	(N=398) 202 (54)	(N=428) 100 (30)	(N=439) 119 (31)	(N=1,454) 759 (62)	(N=2,297) 1,113 (53)	0.005
Loss of job	(N=525) 121 (27)	(N=666) 112 (19)	(N=227) 20 (26)	(N=305) 24 (10)	(N=139) 10 (13)	(N=412) 41 (9)	(N=92) 12 (9)	(N=379) 47 (14)	(N=409) 6 (3)	(N=423) 9 (3)	(N=1,392) 169 (18)	(N=2,185) 233 (12)	0.008
Reduction of working hours	(N=531) 240 (47)	(N=679) 252 (38)	(N=230) 102 (55)	(N=316) 126 (50)	(N=145) 48 (38)	(N=425) 153 (39)	(N=99) 48 (52)	(N=385) 185 (49)	(N=427) 165 (45)	(N=435) 154 (38)	(N=1,432) 603 (47)	(N=2,240) 870 (41)	0.047
Closure of workplace	(N=528) 216 (43)	(N=679) 209 (30)	(N=247) 141 (66)	(N=315) 148 (44)	(N=151) 73 (46)	(N=440) 223 (52)	(N=96) 39 (44)	(N=388) 128 (38)	(N=413) 27 (7)	(N=420) 36 (9)	(N=1,435) 496 (38)	(N=2,242) 744 (35)	0.268
Did you continue to work during COVID-19?	(N=546) 412 (74)	(N=709) 607 (84)	(N=276) 242 (65)	(N=337) 290 (74)	(N=158) 124 (71)	(N=472) 336 (69)	(N=112) 85 (73)	(N=414) 303 (65)	(N=462) 386 (81)	(N=467) 382 (78)	(N=1,554) 1,249 (74)	(N=2,399) 1,918 (75)	0.655
**What are/were your concerns if advised no physical contact/not allowed to go out/allowed to go out only for essential needs?**	N=664	N=812	N=346	N=481	N=186	N=823	N=144	N=568	N=497	N=537	N=1,837	N=3,221	
Financial	(N=660) 594 (92)	(N=806) 621 (80)	(N=323) 194 (59)	(N=452) 225 (62)	(N=174) 59 (35)	(N=776) 212 (32)	(N=135) 76 (61)	(N=543) 239 (37)	(N=486) 139 (33)	(N=529) 163 (24)	(N=1,778) 1,062 (64)	(N=3,106) 1,460 (47)	<0.001
Professional/ career progression	(N=637) 230 (37)	(N=777) 377 (45)	(N=315) 182 (53)	(N=444) 236 (51)	(N=171) 58 (35)	(N=771) 140 (21)	(N=134) 46 (35)	(N=536) 178 (19)	(N=483) 98 (19)	(N=518) 121 (15)	(N=1,740) 614 (35)	(N=3,046) 1,052 (30)	0.033

For peer review only

Suppl. Table 8 Breakdown of economic impacts of COVID-19 and concerns by country and type of income

FBP = fixed salary, benefits/pension; CF = contract and freelance; O = other/no income. Values in cells are n (weighted %) of respondents who replied ‘yes’.

Variable and categories	Thailand			Malaysia			UK			Italy			Slovenia			Total			
**Type of income**	**FBP**	**CF**	**O**	**FBP**	**CF**	**O**	**FBP**	**CF**	**O**	**FBP**	**CF**	**O**	**FBP**	**CF**	**O**	**FBP**	**CF**	**O**	**P-value (for total)**
**If you were working before COVID-19, has COVID-19 created any inconvenience for you?**	N=495	N=738	N=22	N=475	N=125	N=13	N=397	N=210	N=23	N=278	N=228	N=20	N=788	N=101	N=40	N=2,433	N=1,402	N=118	
Loss of earnings	(N=493) 320 (74)	(N=733) 674 (91)	(N=22) 18 (89)	(N=428) 69 (26)	(N=117) 79 (65)	(N=11) 7 (92)	(N=361) 91 (28)	(N=200) 125 (67)	(N=23) 10 (50)	(N=253) 87 (39)	(N=224) 157 (75)	(N=19) 16 (95)	(N=731) 128 (21)	(N=96) 70 (77)	(N=40) 21 (53)	(N=2,266) 695 (38)	(N=1,370) 1,105 (81)	(N=115) 72 (69)	<0.001
Loss of job	(N=478) 78 (21)	(N=692) 148 (23)	(N=21) 7 (47)	(N=420) 18 (8)	(N=101) 24 (31)	(N=11) 2 (78)	(N=350) 20 (6)	(N=179) 30 (17)	(N=22) 1 (6)	(N=247) 6 (3)	(N=206) 45 (27)	(N=18) 8 (36)	(N=709) 6 (2)	(N=83) 5 (6)	(N=40) 4 (10)	(N=2,204) 128 (8)	(N=1,261) 252 (22)	(N=112) 22 (27)	<0.001
Reduction of working hours	(N=479) 226 (52)	(N=710) 259 (36)	(N=21) 7 (45)	(N=429) 163 (51)	(N=106) 60 (56)	(N=11) 5 (12)	(N=358) 89 (24)	(N=189) 102 (60)	(N=23) 10 (48)	(N=256) 111 (45)	(N=210) 113 (56)	(N=18) 9 (26)	(N=735) 227 (33)	(N=89) 67 (81)	(N=38) 25 (70)	(N=2,257) 816 (41)	(N=1,304) 601 (47)	(N=111) 56 (49)	0.042
Closure of workplace	(N=480) 195 (44)	(N=706) 224 (30)	(N=21) 6 (43)	(N=438) 214 (52)	(N=113) 67 (54)	(N=11) 8 (89)	(N=376) 188 (47)	(N=192) 98 (56)	(N=23) 10 (51)	(N=252) 63 (27)	(N=213) 94 (54)	(N=19) 10 (68)	(N=710) 33 (5)	(N=85) 20 (20)	(N=38) 10 (23)	(N=2,256) 693 (33)	(N=1,309) 503 (40)	(N=112) 44 (46)	0.015
Did you continue to work during COVID-19?	(N=495) 418 (83)	(N=738) 584 (77)	(N=22) 17 (78)	(N=475) 437 (83)	(N=125) 86 (42)	(N=13) 9 (25)	(N=397) 319 (79)	(N=210) 126 (57)	(N=23) 15 (62)	(N=278) 234 (81)	(N=228) 146 (51)	(N=20) 8 (15)	(N=788) 682 (84)	(N=101) 63 (57)	(N=40) 23 (59)	(N=2,433) 2,090 (82)	(N=1,402) 1,005 (65)	(N=118) 72 (53)	<0.001
**What are/were your concerns if advised no physical contact/not allowed to go out/allowed to go out only for essential needs?**	N=546	N=849	N=81	N=524	N=158	N=145	N=705	N=227	N=77	N=347	N=244	N=121	N=847	N=103	N=84	N=2,969	N=1,581	N=508	
Financial	(N=543) 402 (81)	(N=843) 753 (89)	(N=80) 60 (76)	(N=488) 231 (58)	(N=149) 110 (83)	(N=138) 78 (39)	(N=658) 131 (22)	(N=219) 116 (56)	(N=73) 24 (34)	(N=324) 102 (30)	(N=238) 165 (66)	(N=116) 48 (43)	(N=830) 190 (23)	(N=102) 74 (61)	(N=83) 38 (40)	(N=2,843) 1,056 (40)	(N=1,551) 1,218 (79)	(N=490) 248 (46)	<0.001
Professional/ career progression	(N=530) 221 (43)	(N=804) 348 (41)	(N=80) 38 (37)	(N=481) 247 (41)	(N=142) 81 (71)	(N=136) 90 (56)	(N=657) 104 (17)	(N=212) 66 (36)	(N=73) 28 (40)	(N=319) 71 (15)	(N=235) 112 (38)	(N=116) 41 (22)	(N=821) 156 (14)	(N=97) 35 (23)	(N=83) 28 (33)	(N=2,808) 799 (24)	(N=1,490) 642 (43)	(N=488) 225 (40)	<0.001

Suppl. Table 9 Breakdown of concerns if advised/not allowed physical contact by country

Values in cells are n (weighted %) of respondents who replied 'yes'.

Variable and categories	Thailand	Malaysia	UK	Italy	Slovenia	Total	P-value (for total)
**What are/were your concerns if advised no physical contact/not allowed to go out/allowed to go out only for essential needs?**	N=1,476	N=827	N=1,009	N=712	N=1,034	N=5,058	
Caring responsibilities (e.g. childcare, caring for elderly parents, not having access to care)	(N=1,454) 890 (62)	(N=772) 456 (57)	(N=946) 325 (31)	(N=681) 312 (46)	(N=1,006) 423 (35)	(N=4,859) 2,406 (47)	<0.001
Physical health (e.g. not being able to attend doctor appointments, medication supply for illnesses, lack of exercise)	(N=1,457) 910 (61)	(N=782) 501 (66)	(N=961) 587 (61)	(N=687) 393 (63)	(N=1,007) 437 (45)	(N=4,894) 2,828 (59)	<0.001
Recreational (e.g. not being able to access recreational facilities like cinemas or restaurants, cancelled sports or cultural events)	(N=1,425) 580 (38)	(N=763) 407 (49)	(N=963) 571 (58)	(N=683) 352 (47)	(N=1,011) 636 (65)	(N=4,845) 2,546 (51)	<0.001
Sports (e.g. participating in competitive or professional sports activities)	(N=1,400) 546 (38)	(N=755) 302 (39)	(N=943) 214 (22)	(N=675) 174 (24)	(N=997) 331 (36)	(N=4,770) 1,567 (32)	<0.001
Mental health and wellbeing (e.g. boredom, loneliness, anxiety, depression)	(N=1,427) 798 (55)	(N=769) 476 (61)	(N=970) 699 (75)	(N=691) 448 (60)	(N=1,008) 436 (43)	(N=4,865) 2,857 (58)	<0.001
Living arrangements (e.g. not enough living space, passing on illness to family members, domestic abuse)	(N=1,419) 646 (45)	(N=753) 289 (46)	(N=943) 215 (24)	(N=674) 114 (16)	(N=999) 177 (15)	(N=4,788) 1,441 (31)	<0.001
Infrastructure (e.g. access to transport, network services, internet access)	(N=1,409) 651 (46)	(N=750) 308 (45)	(N=935) 212 (24)	(N=672) 163 (28)	(N=996) 195 (19)	(N=4,762) 1,529 (33)	<0.001
Social (e.g. not being able to see friends or attend social or family events)	(N=1,440) 768 (52)	(N=773) 474 (56)	(N=974) 768 (79)	(N=686) 525 (70)	(N=1,015) 725 (69)	(N=4,888) 3,260 (64)	<0.001
Religious and spiritual (e.g. not being able to go to church, mosque, temple etc.)	(N=1,433) 591 (42)	(N=769) 393 (58)	(N=942) 162 (17)	(N=670) 95 (18)	(N=998) 201 (19)	(N=4,812) 1,442 (31)	<0.001

Suppl. Table 10 Breakdown of concerns if advised/not allowed physical contact by country and gender

M = male; F = female; O = other/prefer not to say. Values in cells are n (weighted %) of respondents who replied 'yes'.

**Variable and categories**	**Thailand**			**Malaysia**			**UK**			**Italy**			**Slovenia**			**Total**			
**Gender**	**M**	**F**	**O**	**M**	**F**	**O**	**M**	**F**	**O**	**M**	**F**	**O**	**M**	**F**	**O**	**M**	**F**	**O**	**P-value (for total M vs F)**
**What are/were your concerns if advised no physical contact/not allowed to go out/allowed to go out only for essential needs?**	N=704	N=766	N=6	N=298	N=525	N=4	N=426	N=572	N=11	N=222	N=490	N=0	N=366	N=662	N=6	N=2,016	N=3,015	N=27	
Caring responsibilities	(N=697) 430 (61)	(N=751) 456 (62)	(N=6) 4 (67)	(N=282) 170 (53)	(N=486) 284 (62)	(N=4) 2 (50)	(N=407) 124 (27)	(N=529) 197 (35)	(N=10) 4 (40)	(N=213) 82 (36)	(N=468) 230 (56)		(N=356) 124 (25)	(N=644) 297 (44)	(N=6) 2 (33)	(N=1,955) 930 (42)	(N=2,878) 1,464 (52)	(N=26) 12 (46)	<0.001
Physical health	(N=698) 443 (60)	(N=753) 463 (61)	(N=6) 4 (67)	(N=282) 184 (59)	(N=496) 314 (74)	(N=4) 3 (75)	(N=414) 255 (62)	(N=537) 323 (61)	(N=10) 9 (90)	(N=213) 106 (56)	(N=474) 287 (70)		(N=356) 148 (44)	(N=645) 287 (46)	(N=6) 2 (33)	(N=1,963) 1,136 (56)	(N=2,905) 1,674 (61)	(N=26) 18 (69)	0.058
Recreational	(N=681) 267 (39)	(N=738) 310 (38)	(N=6) 3 (50)	(N=275) 160 (54)	(N=484) 246 (44)	(N=4) 1 (25)	(N=411) 253 (61)	(N=542) 309 (56)	(N=10) 9 (90)	(N=215) 126 (54)	(N=468) 226 (41)		(N=359) 239 (71)	(N=646) 395 (59)	(N=6) 2 (33)	(N=1,941) 1,045 (54)	(N=2,878) 1,486 (47)	(N=26) 15 (58)	0.007
Sports	(N=670) 276 (40)	(N=724) 268 (35)	(N=6) 2 (33)	(N=275) 131 (47)	(N=476) 170 (29)	(N=4) 1 (25)	(N=410) 104 (23)	(N=524) 105 (21)	(N=9) 5 (56)	(N=212) 76 (32)	(N=463) 98 (17)		(N=353) 150 (44)	(N=638) 179 (28)	(N=6) 2 (33)	(N=1,920) 737 (38)	(N=2,825) 820 (27)	(N=25) 10 (40)	<0.001
Mental health and wellbeing	(N=684) 377 (55)	(N=737) 418 (55)	(N=6) 3 (50)	(N=279) 167 (62)	(N=486) 307 (61)	(N=4) 2 (50)	(N=414) 287 (73)	(N=545) 402 (77)	(N=11) 10 (91)	(N=216) 122 (56)	(N=475) 326 (63)		(N=357) 128 (40)	(N=645) 305 (46)	(N=6) 3 (50)	(N=1,950) 1,081 (57)	(N=2,888) 1,758 (60)	(N=27) 18 (67)	0.326
Living arrangements	(N=679) 323 (46)	(N=734) 320 (44)	(N=6) 3 (50)	(N=275) 106 (48)	(N=474) 182 (42)	(N=4) 1 (25)	(N=409) 79 (21)	(N=525) 131 (27)	(N=9) 5 (56)	(N=211) 40 (19)	(N=463) 74 (14)		(N=354) 53 (12)	(N=639) 121 (18)	(N=6) 3 (50)	(N=1,928) 601 (31)	(N=2,835) 828 (31)	(N=25) 12 (48)	0.948
Infrastructure	(N=672) 316 (46)	(N=731) 332 (47)	(N=6) 3 (50)	(N=276) 129 (42)	(N=470) 177 (48)	(N=4) 2 (50)	(N=407) 102 (27)	(N=520) 106 (21)	(N=8) 4 (50)	(N=209) 51 (29)	(N=463) 112 (27)		(N=353) 60 (14)	(N=637) 133 (24)	(N=6) 2 (33)	(N=1,917) 658 (32)	(N=2,821) 860 (34)	(N=24) 11 (46)	0.536
Social	(N=689) 369 (53)	(N=745) 395 (51)	(N=6) 4 (67)	(N=280) 179 (62)	(N=489) 294 (48)	(N=4) 1 (25)	(N=412) 321 (79)	(N=551) 438 (79)	(N=11) 9 (82)	(N=215) 163 (66)	(N=471) 362 (74)		(N=360) 245 (70)	(N=649) 475 (69)	(N=6) 5 (83)	(N=1,956) 1,277 (65)	(N=2,905) 1,964 (63)	(N=27) 19 (70)	0.503
Religious and spiritual	(N=689) 290 (41)	(N=738) 298 (44)	(N=6) 3 (50)	(N=279) 140 (55)	(N=486) 251 (61)	(N=4) 2 (50)	(N=408) 73 (19)	(N=524) 86 (14)	(N=10) 3 (30)	(N=208) 33 (21)	(N=462) 62 (15)		(N=355) 77 (24)	(N=637) 124 (14)	(N=6) 0 (0)	(N=1,939) 613 (33)	(N=2,847) 821 (30)	(N=26) 8 (31)	0.367

Suppl. Table 11 Breakdown of concerns if advised/not allowed physical contact by country and age group

Values in cells are n (weighted %) of respondents who replied 'yes'.

**Variable and categories**	**Thailand**			**Malaysia**			**UK**			**Italy**			**Slovenia**			**Total**			
**Age group**	**18-34**	**35-64**	**65+**	**18-34**	**35-64**	**65+**	**18-34**	**35-64**	**65+**	**18-34**	**35-64**	**65+**	**18-34**	**35-64**	**65+**	**18-34**	**35-64**	**65+**	**P-value (for total)**
**What are/were your concerns if advised no physical contact/not allowed to go out/allowed to go out only for essential needs?**	N=223	N=1,152	N=101	N=350	N=442	N=35	N=140	N=616	N=253	N=272	N=383	N=57	N=308	N=676	N=50	N=1,293	N=3,269	N=496	
Caring responsibilities	(N=217) 137 (71)	(N=1,138) 717 (64)	(N=99) 36 (37)	(N=333) 189 (56)	(N=407) 249 (57)	(N=32) 18 (66)	(N=131) 27 (20)	(N=581) 242 (41)	(N=234) 56 (23)	(N=270) 83 (30)	(N=361) 208 (55)	(N=50) 21 (43)	(N=304) 98 (30)	(N=656) 317 (44)	(N=46) 8 (16)	(N=1,255) 534 (46)	(N=3,143) 1,733 (53)	(N=461) 139 (32)	<0.001
Physical health	(N=218) 150 (63)	(N=1,139) 712 (63)	(N=100) 48 (47)	(N=336) 205 (60)	(N=413) 269 (65)	(N=33) 27 (98)	(N=134) 76 (61)	(N=586) 354 (60)	(N=241) 157 (64)	(N=270) 137 (45)	(N=365) 217 (57)	(N=52) 39 (90)	(N=305) 131 (40)	(N=655) 284 (42)	(N=47) 22 (59)	(N=1,263) 699 (56)	(N=3,158) 1,836 (57)	(N=473) 293 (66)	0.044
Recreational	(N=212) 121 (47)	(N=1,118) 425 (35)	(N=95) 34 (34)	(N=331) 183 (55)	(N=403) 209 (44)	(N=29) 15 (40)	(N=136) 96 (66)	(N=589) 339 (57)	(N=238) 136 (53)	(N=270) 169 (66)	(N=362) 166 (44)	(N=51) 17 (38)	(N=302) 213 (71)	(N=663) 395 (60)	(N=46) 28 (70)	(N=1,251) 782 (59)	(N=3,135) 1,534 (47)	(N=459) 230 (48)	0.003
Sports	(N=212) 99 (47)	(N=1,096) 428 (38)	(N=92) 19 (18)	(N=329) 140 (47)	(N=397) 154 (31)	(N=29) 8 (29)	(N=133) 40 (28)	(N=575) 133 (22)	(N=235) 41 (14)	(N=269) 93 (40)	(N=356) 74 (19)	(N=50) 7 (20)	(N=301) 114 (41)	(N=653) 206 (36)	(N=43) 11 (31)	(N=1,244) 486 (42)	(N=3,077) 995 (31)	(N=449) 86 (21)	<0.001
Mental health and wellbeing	(N=212) 146 (63)	(N=1,118) 613 (55)	(N=97) 39 (42)	(N=335) 230 (69)	(N=402) 227 (52)	(N=32) 19 (69)	(N=136) 118 (86)	(N=591) 439 (74)	(N=243) 142 (62)	(N=270) 191 (65)	(N=366) 227 (59)	(N=55) 30 (57)	(N=304) 169 (52)	(N=657) 253 (40)	(N=47) 14 (40)	(N=1,257) 854 (67)	(N=3,134) 1,759 (56)	(N=474) 244 (51)	<0.001
Living arrangements	(N=213) 105 (50)	(N=1,111) 518 (48)	(N=95) 23 (26)	(N=330) 142 (47)	(N=394) 137 (45)	(N=29) 10 (40)	(N=134) 47 (35)	(N=576) 144 (24)	(N=233) 24 (10)	(N=270) 60 (21)	(N=353) 52 (16)	(N=51) 2 (14)	(N=304) 76 (22)	(N=651) 100 (17)	(N=44) 1 (1)	(N=1,251) 430 (38)	(N=3,085) 951 (32)	(N=452) 60 (15)	<0.001
Infrastructure	(N=214) 117 (54)	(N=1,101) 502 (46)	(N=94) 32 (34)	(N=331) 149 (42)	(N=390) 152 (46)	(N=29) 7 (47)	(N=134) 37 (31)	(N=569) 133 (23)	(N=232) 42 (16)	(N=269) 59 (22)	(N=353) 91 (28)	(N=50) 13 (35)	(N=302) 63 (18)	(N=649) 121 (19)	(N=45) 11 (19)	(N=1,250) 425 (37)	(N=3,062) 999 (33)	(N=450) 105 (28)	0.112
Social	(N=216) 147 (59)	(N=1,126) 573 (50)	(N=98) 48 (46)	(N=334) 212 (55)	(N=408) 240 (55)	(N=31) 22 (60)	(N=136) 115 (83)	(N=592) 459 (77)	(N=246) 194 (79)	(N=268) 220 (84)	(N=366) 266 (69)	(N=52) 39 (63)	(N=304) 239 (79)	(N=662) 453 (65)	(N=49) 33 (69)	(N=1,258) 933 (69)	(N=3,154) 1,991 (62)	(N=476) 336 (64)	0.156
Religious and spiritual	(N=213) 86 (45)	(N=1,120) 468 (43)	(N=100) 37 (37)	(N=334) 180 (65)	(N=406) 198 (51)	(N=29) 15 (61)	(N=133) 14 (15)	(N=574) 111 (19)	(N=235) 37 (13)	(N=268) 27 (12)	(N=352) 64 (17)	(N=50) 4 (25)	(N=304) 51 (15)	(N=650) 142 (19)	(N=44) 8 (24)	(N=1,252) 358 (35)	(N=3,102) 983 (31)	(N=458) 101 (28)	0.198

Suppl. Table 12 Breakdown of concerns if advised/not allowed physical contact by country and <u>education level</u>

P/S = primary or lower/secondary education; T = tertiary education. Values in cells are n (weighted %) of respondents who replied ‘yes’.

**Variable and categories**	**Thailand**		**Malaysia**		**UK**		**Italy**		**Slovenia**		**Total**		
**Education level**	**P/S**	**T**	**P/S**	**T**	**P/S**	**T**	**P/S**	**T**	**P/S**	**T**	**P/S**	**T**	**P-value (for total)**
**What are/were your concerns if advised no physical contact/not allowed to go out/allowed to go out only for essential needs?**	N=909	N=567	N=82	N=745	N=247	N=762	N=217	N=495	N=202	N=832	N=1,657	N=3,401	
Caring responsibilities	(N=894)<br>571 (63)	(N=560)<br>319 (57)	(N=74)<br>42 (57)	(N=698)<br>414 (60)	(N=231)<br>78 (30)	(N=715)<br>247 (32)	(N=204)<br>98 (47)	(N=477)<br>214 (45)	(N=190)<br>67 (31)	(N=816)<br>356 (40)	(N=1,593)<br>856 (49)	(N=3,266)<br>1,550 (43)	0.002
Physical health	(N=894)<br>565 (60)	(N=563)<br>345 (63)	(N=75)<br>53 (66)	(N=707)<br>448 (63)	(N=238)<br>146 (63)	(N=723)<br>441 (59)	(N=208)<br>123 (66)	(N=479)<br>270 (56)	(N=191)<br>78 (47)	(N=816)<br>359 (43)	(N=1,606)<br>965 (60)	(N=3,288)<br>1,863 (56)	0.045
Recreational	(N=870)<br>281 (34)	(N=555)<br>299 (57)	(N=72)<br>33 (47)	(N=691)<br>374 (55)	(N=236)<br>120 (52)	(N=727)<br>451 (64)	(N=204)<br>95 (45)	(N=479)<br>257 (52)	(N=192)<br>123 (66)	(N=819)<br>513 (62)	(N=1,574)<br>652 (46)	(N=3,271)<br>1,894 (60)	<0.001
Sports	(N=855)<br>317 (36)	(N=545)<br>229 (43)	(N=71)<br>25 (38)	(N=684)<br>277 (43)	(N=230)<br>34 (17)	(N=713)<br>180 (26)	(N=203)<br>44 (23)	(N=472)<br>130 (27)	(N=190)<br>75 (39)	(N=807)<br>256 (32)	(N=1,549)<br>495 (32)	(N=3,221)<br>1,072 (32)	0.953
Mental health and wellbeing	(N=877)<br>486 (54)	(N=550)<br>312 (59)	(N=74)<br>46 (61)	(N=695)<br>430 (62)	(N=238)<br>174 (76)	(N=732)<br>525 (74)	(N=209)<br>137 (58)	(N=482)<br>311 (63)	(N=190)<br>90 (45)	(N=818)<br>346 (40)	(N=1,588)<br>933 (58)	(N=3,277)<br>1,924 (60)	0.256
Living arrangements	(N=866)<br>422 (46)	(N=553)<br>224 (42)	(N=71)<br>32 (47)	(N=682)<br>257 (39)	(N=232)<br>46 (23)	(N=711)<br>169 (25)	(N=204)<br>37 (17)	(N=470)<br>77 (15)	(N=189)<br>36 (14)	(N=810)<br>141 (16)	(N=1,562)<br>573 (33)	(N=3,226)<br>868 (26)	<0.001
Infrastructure	(N=858)<br>396 (46)	(N=551)<br>255 (48)	(N=70)<br>32 (45)	(N=680)<br>276 (44)	(N=229)<br>44 (23)	(N=706)<br>168 (24)	(N=203)<br>55 (30)	(N=469)<br>108 (23)	(N=189)<br>35 (18)	(N=807)<br>160 (21)	(N=1,549)<br>562 (35)	(N=3,213)<br>967 (29)	0.004
Social	(N=887)<br>440 (49)	(N=553)<br>328 (62)	(N=72)<br>38 (54)	(N=701)<br>436 (63)	(N=242)<br>183 (77)	(N=732)<br>585 (80)	(N=207)<br>157 (67)	(N=479)<br>368 (77)	(N=194)<br>137 (69)	(N=821)<br>588 (70)	(N=1,602)<br>955 (60)	(N=3,286)<br>2,305 (73)	<0.001
Religious and spiritual	(N=882)<br>391 (44)	(N=551)<br>200 (36)	(N=71)<br>42 (60)	(N=698)<br>351 (51)	(N=232)<br>36 (17)	(N=710)<br>126 (17)	(N=202)<br>36 (20)	(N=468)<br>59 (13)	(N=190)<br>28 (18)	(N=808)<br>173 (21)	(N=1,577)<br>533 (35)	(N=3,235)<br>909 (24)	<0.001

Suppl. Table 13 Breakdown of concerns if advised/not allowed physical contact by country and household size

Values in cells are n (weighted %) of respondents who replied ‘yes’.

**Variable and categories**	**Thailand**		**Malaysia**		**UK**		**Italy**		**Slovenia**		**Total**		
**Household size (number of persons in household)**	**1-4**	**>=5**	**1-4**	**>=5**	**1-4**	**>=5**	**1-4**	**>=5**	**1-4**	**>=5**	**1-4**	**>=5**	**P-value (for total)**
**What are/were your concerns if advised no physical contact/not allowed to go out/allowed to go out only for essential needs?**	N=1,038	N=438	N=494	N=333	N=961	N=48	N=659	N=53	N=827	N=207	N=3,979	N=1,079	
Caring responsibilities	(N=1,019)<br>618 (62)	(N=435)<br>272 (61)	(N=461)<br>246 (59)	(N=311)<br>210 (56)	(N=900)<br>299 (30)	(N=46)<br>26 (44)	(N=630)<br>286 (46)	(N=51)<br>26 (52)	(N=806)<br>329 (33)	(N=200)<br>94 (44)	(N=3,816)<br>1,778 (45)	(N=1,043)<br>628 (56)	0.002
Physical health	(N=1,022)<br>639 (61)	(N=435)<br>271 (59)	(N=467)<br>293 (73)	(N=315)<br>208 (56)	(N=916)<br>557 (61)	(N=45)<br>30 (65)	(N=637)<br>363 (63)	(N=50)<br>30 (56)	(N=805)<br>360 (47)	(N=202)<br>77 (38)	(N=3,847)<br>2,212 (60)	(N=1,047)<br>616 (55)	0.153
Recreational	(N=1,002)<br>385 (35)	(N=423)<br>195 (46)	(N=456)<br>241 (47)	(N=307)<br>166 (51)	(N=918)<br>549 (59)	(N=45)<br>22 (53)	(N=633)<br>327 (47)	(N=50)<br>25 (50)	(N=809)<br>518 (65)	(N=202)<br>118 (61)	(N=3,818)<br>2,020 (51)	(N=1,027)<br>526 (50)	0.896
Sports	(N=984)<br>379 (38)	(N=416)<br>167 (38)	(N=447)<br>169 (33)	(N=308)<br>133 (45)	(N=900)<br>207 (22)	(N=43)<br>7 (8)	(N=625)<br>155 (23)	(N=50)<br>19 (42)	(N=798)<br>262 (35)	(N=199)<br>69 (41)	(N=3,754)<br>1,172 (30)	(N=1,016)<br>395 (39)	0.008
Mental health and wellbeing	(N=1,007)<br>567 (57)	(N=420)<br>231 (51)	(N=458)<br>282 (64)	(N=311)<br>194 (58)	(N=925)<br>672 (76)	(N=45)<br>27 (63)	(N=641)<br>414 (59)	(N=50)<br>34 (62)	(N=807)<br>363 (44)	(N=201)<br>73 (41)	(N=3,838)<br>2,298 (60)	(N=1,027)<br>559 (53)	0.031
Living arrangements	(N=1,000)<br>465 (47)	(N=419)<br>181 (42)	(N=448)<br>164 (40)	(N=305)<br>125 (53)	(N=899)<br>199 (23)	(N=44)<br>16 (45)	(N=624)<br>107 (16)	(N=50)<br>7 (16)	(N=798)<br>143 (14)	(N=201)<br>34 (18)	(N=3,769)<br>1,078 (28)	(N=1,019)<br>363 (41)	<0.001
Infrastructure	(N=995)<br>455 (46)	(N=414)<br>196 (47)	(N=445)<br>170 (42)	(N=305)<br>138 (48)	(N=892)<br>204 (23)	(N=43)<br>8 (35)	(N=622)<br>154 (28)	(N=50)<br>9 (19)	(N=796)<br>165 (20)	(N=200)<br>30 (15)	(N=3,750)<br>1,148 (31)	(N=1,012)<br>381 (40)	0.007
Social	(N=1,012)<br>534 (51)	(N=428)<br>234 (53)	(N=461)<br>277 (50)	(N=312)<br>197 (62)	(N=928)<br>736 (80)	(N=46)<br>32 (66)	(N=636)<br>491 (70)	(N=50)<br>34 (70)	(N=811)<br>584 (69)	(N=204)<br>141 (72)	(N=3,848)<br>2,622 (65)	(N=1,040)<br>638 (60)	0.120
Religious and spiritual	(N=1,008)<br>405 (42)	(N=425)<br>186 (44)	(N=457)<br>211 (58)	(N=312)<br>182 (58)	(N=898)<br>151 (16)	(N=44)<br>11 (30)	(N=621)<br>86 (18)	(N=49)<br>9 (24)	(N=797)<br>134 (17)	(N=201)<br>67 (27)	(N=3,781)<br>987 (28)	(N=1,031)<br>455 (44)	<0.001

Suppl. Table 14 Breakdown of concerns if advised/not allowed physical contact by country and whether or not living with children under 18

Values in cells are n (weighted %) of respondents who replied ‘yes’.

**Variable and categories**	**Thailand**		**Malaysia**		**UK**		**Italy**		**Slovenia**		**Total**		
**Living with children under 18**	**Y**	**N**	**Y**	**N**	**Y**	**N**	**Y**	**N**	**Y**	**N**	**Y**	**N**	**P-value (for total)**

**What are/were your concerns if advised no physical contact/not allowed to go out/allowed to go out only for essential needs?**	N=664	N=812	N=346	N=481	N=186	N=823	N=144	N=568	N=497	N=537	N=1,837	N=3,221	
Caring responsibilities	(N=657)<br>487 (73)	(N=797)<br>403 (51)	(N=318)<br>217 (65)	(N=454)<br>239 (52)	(N=177)<br>109 (49)	(N=769)<br>216 (27)	(N=138)<br>88 (63)	(N=543)<br>224 (43)	(N=484)<br>278 (53)	(N=522)<br>145 (22)	(N=1,774)<br>1,179 (64)	(N=3,085)<br>1,227 (38)	<0.001
Physical health	(N=659)<br>458 (67)	(N=798)<br>452 (55)	(N=321)<br>199 (60)	(N=461)<br>302 (70)	(N=179)<br>103 (61)	(N=782)<br>484 (61)	(N=138)<br>77 (56)	(N=549)<br>316 (64)	(N=484)<br>217 (44)	(N=523)<br>220 (46)	(N=1,781)<br>1,054 (59)	(N=3,113)<br>1,774 (59)	0.984
Recreational	(N=644)<br>220 (36)	(N=781)<br>360 (41)	(N=316)<br>169 (48)	(N=447)<br>238 (49)	(N=179)<br>102 (55)	(N=784)<br>469 (59)	(N=139)<br>66 (40)	(N=544)<br>286 (49)	(N=486)<br>284 (60)	(N=525)<br>352 (68)	(N=1,764)<br>841 (46)	(N=3,081)<br>1,705 (53)	0.013
Sports	(N=633)<br>267 (41)	(N=767)<br>279 (35)	(N=318)<br>137 (45)	(N=437)<br>165 (34)	(N=173)<br>52 (24)	(N=770)<br>162 (21)	(N=135)<br>38 (29)	(N=540)<br>136 (23)	(N=478)<br>175 (41)	(N=519)<br>156 (33)	(N=1,737)<br>669 (39)	(N=3,033)<br>898 (29)	<0.001
Mental health and wellbeing	(N=641)<br>415 (63)	(N=786)<br>383 (48)	(N=318)<br>190 (56)	(N=451)<br>286 (65)	(N=180)<br>139 (80)	(N=790)<br>560 (74)	(N=139)<br>91 (60)	(N=552)<br>357 (60)	(N=481)<br>197 (44)	(N=527)<br>239 (43)	(N=1,759)<br>1,032 (59)	(N=3,106)<br>1,825 (58)	0.841
Living arrangements	(N=641)<br>366 (54)	(N=778)<br>280 (37)	(N=311)<br>118 (55)	(N=442)<br>171 (39)	(N=174)<br>56 (36)	(N=769)<br>159 (21)	(N=134)<br>24 (19)	(N=540)<br>90 (16)	(N=479)<br>93 (21)	(N=520)<br>84 (11)	(N=1,739)<br>657 (42)	(N=3,049)<br>784 (24)	<0.001
Infrastructure	(N=632)<br>322 (50)	(N=777)<br>329 (43)	(N=310)<br>131 (48)	(N=440)<br>177 (42)	(N=172)<br>37 (29)	(N=763)<br>175 (23)	(N=135)<br>30 (18)	(N=537)<br>133 (30)	(N=477)<br>81 (17)	(N=519)<br>114 (20)	(N=1,726)<br>601 (37)	(N=3,036)<br>928 (31)	0.018
Social	(N=651)<br>347 (52)	(N=789)<br>421 (52)	(N=322)<br>194 (53)	(N=451)<br>280 (57)	(N=179)<br>141 (82)	(N=795)<br>627 (78)	(N=140)<br>109 (77)	(N=546)<br>416 (69)	(N=488)<br>341 (69)	(N=527)<br>384 (70)	(N=1,780)<br>1,132 (61)	(N=3,108)<br>2,128 (66)	0.098
Religious and spiritual	(N=641)<br>307 (49)	(N=792)<br>284 (36)	(N=319)<br>174 (58)	(N=450)<br>219 (58)	(N=171)<br>30 (19)	(N=771)<br>132 (16)	(N=133)<br>23 (20)	(N=537)<br>72 (18)	(N=479)<br>118 (20)	(N=519)<br>83 (18)	(N=1,743)<br>652 (39)	(N=3,069)<br>790 (28)	<0.001

For peer review only - http://bmjopen.bmj.com/site/about/guidelines.xhtml

Suppl. Table 15 Breakdown of concerns if advised/not allowed physical contact by country and income type

FBP = fixed salary, benefits/pension; CF = contract and freelance; O = other/no income. Values in cells are n (weighted %) of respondents who replied ‘yes’.

Variable and categories	Thailand			Malaysia			UK			Italy			Slovenia			Total			
**Type of income**	**FBP**	**CF**	**O**	**FBP**	**CF**	**O**	**FBP**	**CF**	**O**	**FBP**	**CF**	**O**	**FBP**	**CF**	**O**	**FBP**	**CF**	**O**	**P-value (for total)**
**What are/were your concerns if advised no physical contact/not allowed to go out/allowed to go out only for essential needs?**	N=546	N=849	N=81	N=524	N=158	N=145	N=705	N=227	N=77	N=347	N=244	N=121	N=847	N=103	N=84	N=2,969	N=1,581	N=508	
Caring responsibilities	(N=540) 372 (72)	(N=836) 481 (57)	(N=78) 37 (39)	(N=490) 307 (58)	(N=145) 78 (64)	(N=137) 71 (47)	(N=661) 223 (32)	(N=213) 83 (32)	(N=72) 19 (26)	(N=328) 167 (49)	(N=236) 101 (41)	(N=117) 44 (44)	(N=826) 362 (36)	(N=97) 42 (31)	(N=83) 19 (23)	(N=2,845) 1,431 (47)	(N=1,527) 785 (51)	(N=487) 190 (38)	0.028
Physical health	(N=543) 381 (70)	(N=835) 482 (56)	(N=79) 47 (49)	(N=497) 324 (63)	(N=146) 89 (71)	(N=139) 88 (66)	(N=672) 415 (62)	(N=216) 124 (60)	(N=73) 48 (63)	(N=333) 204 (68)	(N=236) 122 (51)	(N=118) 67 (59)	(N=826) 345 (44)	(N=98) 56 (58)	(N=83) 36 (42)	(N=2,871) 1,669 (59)	(N=1,531) 873 (58)	(N=492) 286 (57)	0.826
Recreational	(N=535) 243 (43)	(N=812) 296 (35)	(N=78) 41 (42)	(N=483) 253 (46)	(N=143) 78 (48)	(N=137) 76 (56)	(N=671) 386 (54)	(N=218) 134 (65)	(N=74) 51 (71)	(N=331) 153 (46)	(N=236) 136 (50)	(N=116) 63 (47)	(N=828) 511 (62)	(N=101) 63 (75)	(N=82) 62 (75)	(N=2,848) 1,546 (52)	(N=1,510) 707 (46)	(N=487) 293 (58)	0.024
Sports	(N=531) 264 (53)	(N=791) 249 (29)	(N=78) 33 (32)	(N=474) 190 (35)	(N=145) 63 (47)	(N=136) 49 (39)	(N=660) 133 (18)	(N=213) 57 (28)	(N=70) 24 (30)	(N=325) 72 (22)	(N=234) 70 (26)	(N=116) 32 (28)	(N=818) 265 (34)	(N=96) 34 (46)	(N=83) 32 (45)	(N=2,808) 924 (32)	(N=1,479) 473 (32)	(N=483) 170 (36)	0.582
Mental health and wellbeing	(N=533) 339 (65)	(N=816) 410 (50)	(N=78) 49 (50)	(N=485) 297 (61)	(N=146) 86 (58)	(N=138) 93 (66)	(N=676) 485 (75)	(N=221) 157 (74)	(N=73) 57 (80)	(N=335) 213 (60)	(N=238) 147 (55)	(N=118) 88 (68)	(N=826) 346 (43)	(N=99) 42 (38)	(N=83) 48 (53)	(N=2,855) 1,680 (59)	(N=1,520) 842 (55)	(N=490) 335 (63)	0.125
Living arrangements	(N=533) 268 (51)	(N=808) 352 (43)	(N=78) 26 (27)	(N=474) 181 (48)	(N=142) 54 (55)	(N=137) 54 (27)	(N=655) 128 (19)	(N=216) 65 (34)	(N=72) 22 (30)	(N=325) 57 (17)	(N=233) 38 (16)	(N=116) 19 (14)	(N=821) 138 (14)	(N=95) 15 (13)	(N=83) 24 (29)	(N=2,808) 772 (27)	(N=1,494) 524 (38)	(N=486) 145 (26)	<0.001
Infrastructure	(N=530) 279 (56)	(N=800) 335 (42)	(N=79) 37 (35)	(N=473) 179 (46)	(N=141) 55 (39)	(N=136) 74 (48)	(N=654) 134 (21)	(N=210) 56 (30)	(N=71) 22 (29)	(N=325) 74 (30)	(N=230) 56 (23)	(N=117) 33 (26)	(N=819) 157 (19)	(N=94) 15 (13)	(N=83) 23 (25)	(N=2,801) 823 (32)	(N=1,475) 517 (36)	(N=486) 189 (35)	0.370
Social	(N=537) 322 (58)	(N=824) 398 (48)	(N=79) 48 (51)	(N=491) 303 (55)	(N=146) 81 (59)	(N=136) 90 (52)	(N=681) 531 (78)	(N=219) 177 (79)	(N=74) 60 (81)	(N=335) 256 (72)	(N=233) 173 (63)	(N=118) 96 (78)	(N=834) 589 (68)	(N=98) 66 (67)	(N=83) 70 (86)	(N=2,878) 2,001 (67)	(N=1,520) 895 (58)	(N=490) 364 (67)	0.004
Religious and spiritual	(N=532) 235 (49)	(N=823) 326 (39)	(N=78) 30 (35)	(N=486) 254 (57)	(N=145) 68 (57)	(N=138) 71 (62)	(N=659) 121 (17)	(N=210) 31 (16)	(N=73) 10 (12)	(N=322) 43 (20)	(N=231) 36 (14)	(N=117) 16 (17)	(N=821) 168 (18)	(N=94) 22 (31)	(N=83) 11 (14)	(N=2,820) 821 (29)	(N=1,503) 483 (34)	(N=489) 138 (33)	0.195

Suppl. Table 16 Breakdown of maximum number of days that people thought they could cope by country

Values in cells are n (weighted %) of respondents who replied 'yes'.

Variable and categories	Thailand	Malaysia	UK	Italy	Slovenia	Total	P-value
**What is the maximum number of days you think you could cope without meeting family or friends not living in your household in person?**	N=1,476	N=827	N=1,009	N=712	N=1,034	N=5,058	<0.001
1 to 14 days	957 (66)	201 (31)	192 (21)	127 (23)	261 (34)	1,738 (39)	
>14 to 28 days	223 (13)	110 (16)	98 (11)	95 (14)	169 (16)	695 (14)	
29 days+	296 (21)	516 (52)	719 (68)	490 (63)	604 (50)	2,625 (47)	
**What is the maximum number of days you think you could cope with not going out in public, assuming that you have sufficient supplies of food, medicines and other essential items?**	N=1,476	N=827	N=1,009	N=712	N=1,034	N=5,058	<0.001
1 to 14 days	805 (54)	270 (41)	393 (40)	304 (45)	601 (61)	2,373 (49)	
>14 to 28 days	249 (17)	114 (16)	124 (14)	161 (21)	151 (13)	799 (16)	
29 days+	422 (29)	443 (43)	492 (46)	247 (34)	282 (26)	1,886 (35)	
**What is the maximum number of days you think you could cope with going out only for essential needs/work?**	N=1,476	N=827	N=1,009	N=712	N=1,034	N=5,058	<0.001
1 to 14 days	808 (56)	268 (40)	272 (29)	205 (33)	310 (37)	1,863 (41)	
>14 to 28 days	258 (17)	98 (14)	100 (10)	110 (17)	182 (18)	748 (15)	
29 days+	410 (26)	461 (46)	637 (60)	397 (51)	542 (45)	2,447 (44)	

For peer review only - http://bmjopen.bmj.com/site/about/guidelines.xhtml

Suppl. Table 17 Breakdown of maximum number of days that people thought they could cope by country and gender

M = male; F = female; O = other/prefer not to say. Values in cells are n (weighted %) of respondents who replied ‘yes’.

**Variable and categories**	**Thailand**			**Malaysia**			**UK**			**Italy**			**Slovenia**			**Total**			
**Gender**	**M**	**F**	**O**	**M**	**F**	**O**	**M**	**F**	**O**	**M**	**F**	**O**	**M**	**F**	**O**	**M**	**F**	**O**	**P-value (for total M vs F)**
**What is the maximum number of days you think you could cope without meeting family or friends not living in your household in person?**	N=704	N=766	N=6	N=298	N=525	N=4	N=426	N=572	N=11	N=222	N=490	N=0	N=366	N=662	N=6	N=2,016	N=3,015	N=27	0.381
1 to 14 days	479 (66)	476 (66)	2 (33)	68 (29)	132 (34)	1 (25)	87 (23)	102 (19)	3 (27)	46 (28)	81 (18)		113 (38)	147 (31)	1 (17)	793 (40)	938 (37)	7 (26)	
>14 to 28 days	99 (12)	123 (15)	1 (17)	40 (14)	69 (18)	1 (25)	43 (13)	54 (9)	1 (9)	28 (11)	67 (17)		49 (14)	120 (18)	0 (0)	259 (13)	433 (15)	3 (11)	
29 days+	126 (23)	167 (19)	3 (50)	190 (57)	324 (48)	2 (50)	296 (64)	416 (72)	7 (64)	148 (61)	342 (65)		204 (48)	395 (51)	5 (83)	964 (47)	1,644 (47)	17 (63)	
**What is the maximum number of days you think you could cope with not going out in public, assuming that you have sufficient supplies of food, medicines and other essential items?**	N=704	N=766	N=6	N=298	N=525	N=4	N=426	N=572	N=11	N=222	N=490		N=366	N=662	N=6	N=2,016	N=3,015	N=27	0.890
1 to 14 days	398 (53)	405 (55)	2 (33)	96 (41)	173 (40)	1 (25)	170 (42)	219 (38)	4 (36)	100 (48)	204 (42)		217 (57)	382 (65)	2 (33)	981 (49)	1,383 (50)	9 (33)	
>14 to 28 days	116 (18)	132 (16)	1 (17)	47 (18)	66 (14)	1 (25)	53 (14)	71 (13)	0 (0)	46 (18)	115 (24)		40 (14)	111 (12)	0 (0)	302 (16)	495 (16)	2 (7)	
29 days+	190 (30)	229 (29)	3 (50)	155 (41)	286 (46)	2 (50)	203 (43)	282 (49)	7 (64)	76 (34)	171 (34)		109 (29)	169 (23)	4 (67)	733 (35)	1,137 (35)	16 (59)	
**What is the maximum number of days you think you could cope with going out only for essential needs/work?**	N=704	N=766	N=6	N=298	N=525	N=4	N=426	N=572	N=11	N=222	N=490		N=366	N=662	N=6	N=2,016	N=3,015	N=27	0.680
1 to 14 days	418 (57)	388 (55)	2 (33)	94 (41)	173 (38)	1 (25)	127 (32)	141 (27)	4 (36)	72 (35)	133 (31)		125 (35)	183 (40)	2 (33)	836 (42)	1,018 (40)	9 (33)	
>14 to 28 days	114 (17)	142 (17)	2 (33)	35 (11)	62 (17)	1 (25)	40 (10)	60 (10)	0 (0)	31 (17)	79 (17)		73 (23)	109 (13)	0 (0)	293 (16)	452 (15)	3 (11)	
29 days+	172 (25)	236 (27)	2 (33)	169 (47)	290 (45)	2 (50)	259 (58)	371 (62)	7 (64)	119 (49)	278 (52)		168 (43)	370 (47)	4 (67)	887 (42)	1,545 (45)	15 (56)	

Suppl. Table 18 Breakdown of maximum number of days that people thought they could cope by country and age group

Values in cells are n (weighted %) of respondents who replied 'yes'.

Variable and categories	Thailand			Malaysia			UK			Italy			Slovenia			Total			
**Age group**	**18-34**	**35-64**	**65+**	**18-34**	**35-64**	**65+**	**18-34**	**35-64**	**65+**	**18-34**	**35-64**	**65+**	**18-34**	**35-64**	**65+**	**18-34**	**35-64**	**65+**	**P-value (for total)**
**What is the maximum number of days you think you could cope without meeting family or friends not living in your household in person?**	N=223	N=1,152	N=101	N=350	N=442	N=35	N=140	N=616	N=253	N=272	N=383	N=57	N=308	N=676	N=50	N=1,293	N=3,269	N=496	0.409
1 to 14 days	115 (57)	774 (70)	68 (67)	96 (32)	96 (25)	9 (55)	22 (22)	112 (18)	58 (24)	37 (19)	81 (26)	9 (19)	78 (29)	167 (31)	16 (49)	348 (36)	1,230 (39)	160 (42)	
>14 to 28 days	29 (10)	179 (15)	15 (15)	51 (19)	53 (13)	6 (22)	16 (13)	55 (10)	27 (12)	42 (20)	42 (11)	11 (17)	49 (17)	112 (15)	8 (18)	187 (10)	441 (13)	67 (16)	
29 days+	79 (33)	199 (15)	18 (18)	203 (49)	293 (62)	20 (23)	102 (65)	449 (72)	168 (64)	193 (62)	260 (63)	37 (64)	181 (54)	397 (54)	26 (34)	758 (50)	1,598 (48)	269 (42)	
**What is the maximum number of days you think you could cope with not going out in public, assuming that you have sufficient supplies of food, medicines and other essential items?**	N=223	N=1,152	N=101	N=350	N=442	N=35	N=140	N=616	N=253	N=272	N=383	N=57	N=308	N=676	N=50	N=1,293	N=3,269	N=496	0.335
1 to 14 days	113 (48)	643 (58)	49 (50)	116 (42)	141 (36)	13 (56)	62 (42)	222 (37)	109 (47)	111 (45)	170 (44)	23 (47)	192 (61)	382 (59)	27 (67)	594 (47)	1,558 (49)	221 (53)	
>14 to 28 days	33 (17)	192 (16)	24 (20)	43 (13)	65 (17)	6 (28)	19 (17)	85 (14)	20 (9)	65 (19)	82 (19)	14 (27)	36 (11)	107 (14)	8 (15)	196 (15)	531 (16)	72 (18)	
29 days+	77 (35)	317 (26)	28 (30)	191 (45)	236 (47)	16 (16)	59 (40)	309 (50)	124 (45)	96 (36)	131 (37)	20 (26)	80 (28)	187 (28)	15 (19)	503 (37)	1,180 (36)	203 (29)	
**What is the maximum number of days you think you could cope with going out only for essential needs/work?**	N=223	N=1,152	N=101	N=350	N=442	N=35	N=140	N=616	N=253	N=272	N=383	N=57	N=308	N=676	N=50	N=1,293	N=3,269	N=496	0.255
1 to 14 days	107 (52)	648 (59)	53 (56)	91 (32)	163 (43)	14 (62)	33 (28)	161 (27)	78 (36)	62 (27)	126 (36)	17 (32)	98 (34)	189 (33)	23 (51)	391 (37)	1,287 (42)	185 (46)	
>14 to 28 days	43 (18)	195 (17)	20 (17)	40 (13)	54 (14)	4 (15)	17 (12)	58 (10)	25 (8)	48 (20)	52 (14)	10 (20)	53 (17)	121 (17)	8 (19)	201 (16)	480 (15)	67 (16)	
29 days+	73 (30)	309 (24)	28 (27)	219 (55)	225 (43)	17 (22)	90 (60)	397 (63)	150 (56)	162 (53)	205 (51)	30 (48)	157 (49)	366 (50)	19 (29)	701 (48)	1,502 (43)	244 (38)	

Suppl. Table 19 Breakdown of maximum number of days that people thought they could cope by country and household size

Values in cells are n (weighted %) of respondents who replied 'yes'.

**Variable and categories**	**Thailand**		**Malaysia**		**UK**		**Italy**		**Slovenia**		**Total**		
**Household size (number of persons in household)**	**1-4**	**≥5**	**1-4**	**≥5**	**1-4**	**≥5**	**1-4**	**≥5**	**1-4**	**≥5**	**1-4**	**≥5**	**P-value (for total)**
**What is the maximum number of days you think you could cope without meeting family or friends not living in your household in person?**	N=1,038	N=438	N=494	N=333	N=961	N=48	N=659	N=53	N=827	N=207	N=3,979	N=1,079	0.023
1 to 14 days	674 (68)	283 (61)	105 (26)	96 (38)	185 (21)	7 (12)	118 (23)	9 (24)	216 (36)	45 (28)	1,298 (37)	440 (44)	
>14 to 28 days	150 (13)	73 (15)	67 (12)	43 (22)	95 (12)	3 (3)	93 (15)	2 (2)	139 (17)	30 (12)	544 (14)	151 (16)	
29 days+	214 (19)	82 (24)	322 (62)	194 (40)	681 (67)	38 (85)	448 (62)	42 (75)	472 (47)	132 (59)	2,137 (49)	488 (40)	
**What is the maximum number of days you think you could cope with not going out in public, assuming that you have sufficient supplies of food, medicines and other essential items?**	N=1,038	N=438	N=494	N=333	N=961	N=48	N=659	N=53	N=827	N=207	N=3,979	N=1,079	0.492
1 to 14 days	594 (59)	211 (44)	160 (29)	110 (56)	375 (40)	18 (49)	285 (45)	19 (46)	487 (61)	114 (63)	1,901 (49)	472 (51)	
>14 to 28 days	158 (14)	91 (22)	68 (19)	46 (12)	114 (14)	10 (14)	146 (21)	15 (24)	123 (14)	28 (11)	609 (16)	190 (17)	
29 days+	286 (27)	136 (34)	266 (52)	177 (32)	472 (47)	20 (37)	228 (34)	19 (30)	217 (26)	65 (26)	1,469 (36)	417 (32)	
**What is the maximum number of days you think you could cope with going out only for essential needs/work?**	N=1,038	N=438	N=494	N=333	N=961	N=48	N=659	N=53	N=827	N=207	N=3,979	N=1,079	0.079
1 to 14 days	579 (58)	229 (54)	165 (35)	103 (47)	262 (30)	10 (24)	197 (33)	8 (21)	255 (37)	55 (40)	1,458 (39)	405 (47)	
>14 to 28 days	172 (15)	86 (21)	63 (20)	35 (6)	96 (11)	4 (5)	104 (17)	6 (8)	146 (18)	36 (14)	581 (16)	167 (14)	
29 days+	287 (27)	123 (25)	266 (46)	195 (46)	603 (60)	34 (72)	358 (50)	39 (72)	426 (45)	116 (46)	1,940 (45)	507 (39)	

For peer review only - http://bmjopen.bmj.com/site/about/guidelines.xhtml

Suppl. Table 20 Breakdown of maximum number of days that people thought they could cope by country and whether or not living with children under 18

Y = living with children under 18; N = not living with children under 18. Values in cells are n (weighted %) of respondents who replied 'yes'.

**Variable and categories**	**Thailand**		**Malaysia**		**UK**		**Italy**		**Slovenia**		**Total**		
**Living with children under 18**	**Y**	**N**	**Y**	**N**	**Y**	**N**	**Y**	**N**	**Y**	**N**	**Y**	**N**	**P-value (for total)**
**What is the maximum number of days you think you could cope without meeting family or friends not living in your household in person?**	N=664	N=812	N=346	N=481	N=186	N=823	N=144	N=568	N=497	N=537	N=1,837	N=3,221	<0.001
1 to 14 days	490 (72)	467 (60)	97 (40)	104 (25)	24 (14)	168 (22)	24 (18)	103 (24)	115 (30)	146 (38)	750 (46)	988 (35)	
>14 to 28 days	80 (10)	143 (17)	37 (12)	73 (19)	18 (12)	80 (11)	13 (9)	82 (16)	79 (14)	90 (18)	227 (12)	468 (16)	
29 days+	94 (18)	202 (23)	212 (47)	304 (56)	144 (74)	575 (67)	107 (73)	383 (61)	303 (57)	301 (45)	860 (42)	1,765 (50)	
**What is the maximum number of days you think you could cope with not going out in public, assuming that you have sufficient supplies of food, medicines and other essential items?**	N=664	N=812	N=346	N=481	N=186	N=823	N=144	N=568	N=497	N=537	N=1,837	N=3,221	<0.001
1 to 14 days	412 (59)	393 (49)	120 (57)	150 (29)	60 (36)	333 (41)	62 (44)	242 (45)	290 (62)	311 (60)	944 (56)	1,429 (46)	
>14 to 28 days	100 (16)	149 (18)	45 (11)	69 (20)	34 (19)	90 (12)	33 (26)	128 (20)	73 (13)	78 (14)	285 (15)	514 (17)	
29 days+	152 (25)	270 (33)	181 (33)	262 (51)	92 (46)	400 (46)	49 (31)	198 (34)	134 (25)	148 (26)	608 (29)	1,278 (38)	
**What is the maximum number of days you think you could cope with going out only for essential needs/work?**	N=664	N=812	N=346	N=481	N=186	N=823	N=144	N=568	N=497	N=537	N=1,837	N=3,221	0.004
1 to 14 days	407 (63)	401 (51)	117 (47)	151 (35)	33 (21)	239 (31)	42 (35)	163 (32)	139 (35)	171 (39)	738 (47)	1,125 (38)	
>14 to 28 days	112 (16)	146 (18)	37 (8)	61 (18)	17 (8)	83 (11)	20 (11)	90 (18)	90 (16)	92 (18)	276 (14)	472 (16)	
29 days+	145 (21)	265 (31)	192 (45)	269 (47)	136 (71)	501 (58)	82 (53)	315 (50)	268 (49)	274 (42)	823 (40)	1,624 (46)	

For peer review only

Suppl. Table 21 Breakdown of maximum number of days that people thought they could cope by country and education level

P/S = primary or lower/secondary education; T = tertiary education. Values in cells are n (weighted %) of respondents who replied ‘yes’.

**Variable and categories**	**Thailand**		**Malaysia**		**UK**		**Italy**		**Slovenia**		**Total**		
**Education level**	**P/S**	**T**	**P/S**	**T**	**P/S**	**T**	**P/S**	**T**	**P/S**	**T**	**P/S**	**T**	**P-value (for total)**
**What is the maximum number of days you think you could cope without meeting family or friends not living in your household in person?**	N=909	N=567	N=82	N=745	N=247	N=762	N=217	N=495	N=202	N=832	N=1,657	N=3,401	<0.001
1 to 14 days	659 (69)	298 (51)	27 (33)	174 (23)	55 (24)	137 (18)	53 (26)	74 (16)	69 (41)	192 (24)	863 (45)	875 (25)	
>14 to 28 days	122 (12)	101 (17)	15 (17)	95 (13)	30 (13)	68 (9)	31 (15)	64 (13)	33 (16)	136 (16)	231 (15)	464 (13)	
29 days+	128 (18)	168 (32)	40 (50)	476 (64)	162 (63)	557 (73)	133 (59)	357 (72)	100 (43)	504 (60)	563 (41)	2,062 (62)	
**What is the maximum number of days you think you could cope with not going out in public, assuming that you have sufficient supplies of food, medicines and other essential items?**	N=909	N=567	N=82	N=745	N=247	N=762	N=217	N=495	N=202	N=832	N=1,657	N=3,401	0.004
1 to 14 days	541 (56)	264 (47)	34 (43)	236 (32)	101 (41)	292 (40)	95 (46)	209 (43)	119 (63)	482 (58)	890 (51)	1,483 (45)	
>14 to 28 days	144 (17)	105 (18)	15 (17)	99 (13)	31 (15)	93 (13)	41 (20)	120 (24)	23 (12)	128 (15)	254 (16)	545 (16)	
29 days+	224 (28)	198 (35)	33 (40)	410 (55)	115 (44)	377 (48)	81 (34)	166 (33)	60 (25)	222 (27)	513 (33)	1,373 (39)	
**What is the maximum number of days you think you could cope with going out only for essential needs/work?**	N=909	N=567	N=82	N=745	N=247	N=762	N=217	N=495	N=202	N=832	N=1,657	N=3,401	<0.001
1 to 14 days	564 (59)	244 (43)	35 (43)	233 (29)	87 (35)	185 (24)	70 (35)	135 (29)	75 (42)	235 (31)	831 (46)	1,032 (30)	
>14 to 28 days	156 (17)	102 (19)	12 (14)	86 (11)	26 (10)	74 (10)	39 (18)	71 (14)	33 (17)	149 (18)	266 (16)	482 (14)	
29 days+	189 (24)	221 (38)	35 (43)	426 (59)	134 (54)	503 (66)	108 (48)	289 (57)	94 (41)	448 (51)	560 (38)	1,887 (56)	

For peer review only - http://bmjopen.bmj.com/site/about/guidelines.xhtml

Suppl. Table 22 Breakdown of maximum number of days that people thought they could cope by country and type of income

FBP = fixed salary, benefits/pension; CF = contract and freelance; O = other. Values in cells are n (weighted %) of respondents who replied ‘yes’.

Variable and categories	Thailand			Malaysia			UK			Italy			Slovenia			Total			
Type of income	FBP	CF	O	FBP	CF	O	FBP	CF	O	FBP	CF	O	FBP	CF	O	FBP	CF	O	P-value (for total)
**What is the maximum number of days you think you could cope without meeting family or friends not living in your household in person?**	N=546	N=849	N=81	N=524	N=158	N=145	N=705	N=227	N=77	N=347	N=244	N=121	N=847	N=103	N=84	N=2,969	N=1,581	N=508	<0.001
1 to 14 days	344 (64)	577 (69)	36 (43)	135 (23)	35 (37)	31 (48)	134 (22)	36 (17)	22 (24)	58 (22)	47 (27)	22 (18)	208 (34)	35 (44)	18 (26)	879 (33)	730 (50)	129 (34)	
>14 to 28 days	74 (11)	134 (14)	15 (17)	57 (15)	24 (16)	29 (19)	69 (11)	25 (14)	4 (7)	46 (15)	30 (12)	19 (15)	141 (17)	19 (16)	9 (9)	387 (14)	232 (14)	76 (14)	
29 days+	128 (25)	138 (16)	30 (41)	332 (62)	99 (47)	85 (33)	502 (68)	166 (69)	51 (69)	243 (63)	167 (60)	80 (66)	498 (49)	49 (40)	57 (65)	1,703 (53)	619 (35)	303 (51)	
**What is the maximum number of days you think you could cope with not going out in public, assuming that you have sufficient supplies of food, medicines and other essential items?**	N=546	N=849	N=81	N=524	N=158	N=145	N=705	N=227	N=77	N=347	N=244	N=121	N=847	N=103	N=84	N=2,969	N=1,581	N=508	0.471
1 to 14 days	313 (55)	461 (55)	31 (39)	183 (38)	46 (39)	41 (49)	273 (40)	87 (41)	33 (42)	147 (45)	108 (47)	49 (40)	485 (560	66 (75)	50 (59)	1,401 (49)	768 (51)	204 (46)	
>14 to 28 days	85 (16)	148 (17)	16 (20)	70 (18)	22 (17)	22 (10)	90 (13)	28 (17)	6 (9)	84 (24)	55 (17)	22 (14)	129 (14)	12 (7)	10 (14)	458 (16)	265 (16)	76 (13)	
29 days+	148 (29)	240 (28)	34 (40)	271 (44)	90 (44)	82 (41)	342 (47)	112 (43)	38 (49)	116 (30)	81 (36)	50 (46)	233 (27)	25 (18)	24 (27)	1,110 (35)	548 (33)	228 (41)	
**What is the maximum number of days you think you could cope with going out only for essential needs/work?**	N=546	N=849	N=81	N=524	N=158	N=145	N=705	N=227	N=77	N=347	N=244	N=121	N=847	N=103	N=84	N=2,969	N=1,581	N=508	<0.001
1 to 14 days	297 (59)	478 (56)	33 (43)	181 (38)	56 (53)	31 (29)	186 (29)	64 (31)	22 (22)	99 (33)	78 (34)	28 (27)	250 (38)	41 (45)	19 (27)	1,013 (39)	717 (49)	133 (30)	
>14 to 28 days	81 (16)	159 (18)	18 (23)	54 (14)	23 (4)	21 (25)	68 (10)	20 (10)	12 (16)	55 (18)	30 (12)	25 (19)	150 (17)	17 (21)	15 (17)	408 (15)	249 (14)	91 (21)	
29 days+	168 (25)	212 (26)	30 (34)	289 (48)	79 (43)	93 (46)	451 (61)	143 (58)	43 (62)	193 (49)	136 (53)	68 (54)	447 (45)	45 (34)	50 (57)	1,548 (46)	615 (37)	284 (50)	

For peer review only - http://bmjopen.bmj.com/site/about/guidelines.xhtml

Suppl. Table 23 Breakdown of behavioural changes and acceptance of government public health measures by country

Values in cells are n (weighted %) of respondents who replied 'yes'.

Variable and categories	Thailand	Malaysia	UK	Italy	Slovenia	Total	P-value
	N=1,476	N=827	N=1,009	N=712	N=1,034	N=5,058	
**Did you change your social behaviour before the implementation of government restrictions?**	1,374 (93)	538 (64)	712 (68)	356 (47)	584 (47)	3,564 (67)	<0.001
**If you answered 'yes' to the previous question: how did you change your social behaviour?**							
No physical contact with anyone	(N=1,374) 1,302 (94)	(N=506) 362 (82)	(N=657) 325 (51)	(N=342) 243 (74)	(N=576) 516 (93)	(N=3,455) 2,748 (82)	<0.001
No physical contact only with elderly and those with serious underlying medical conditions	(N=1,374) 1,200 (88)	(N=494) 292 (63)	(N=644) 393 (60)	(N=332) 272 (79)	(N=566) 516 (91)	(N=3,410) 2,673 (79)	<0.001
Going out only for essential needs	(N=1,374) 1,291 (94)	(N=525) 489 (95)	(N=681) 571 (83)	(N=346) 263 (82)	(N=562) 381 (71)	(N=3,488) 2,995 (87)	<0.001
Moving home to stay with parents/relatives	(N=1,374) 677 (54)	(N=489) 99 (26)	(N=627) 30 (8)	(N=326) 27 (6)	(N=552) 33 (5)	(N=3,368) 866 (30)	<0.001
Use of personal protection equipment (e.g. masks and gloves)	(N=1,374) 1,334 (96)	(N=527) 488 (95)	(N=651) 225 (33)	(N=339) 165 (55)	(N=564) 366 (67)	(N=3,455) 2,578 (76)	<0.001
Use of sanitizer products and alcohol	(N=1,374) 1,321 (95)	(N=529) 504 (96)	(N=685) 559 (83)	(N=350) 307 (91)	(N=569) 521 (94)	(N=3,507) 3,212 (92)	<0.001
**"I would comply with government enforced quarantine/ isolation/social distancing."**	N=1,476	N=827	N=1,009	N=712	N=1,034	N=5,058	<0.001
Agree	1,344 (92)	708 (86)	822 (80)	606 (78)	871 (75)	4,351 (83)	
Neither agree nor disagree	92 (5)	18 (0)	48 (4)	36 (7)	68 (14)	262 (6)	
Disagree	40 (3)	101 (14)	139 (15)	70 (15)	95 (11)	445 (10)	
**"I would enter voluntary quarantine/isolation/social distancing for social/self-responsibility."**	N=1,476	N=827	N=1,009	N=712	N=1,034	N=5,058	<0.001
Agree	1,354 (92)	674 (81)	815 (78)	566 (76)	838 (76)	4,247 (82)	
Neither agree nor disagree	100 (7)	48 (4)	50 (5)	59 (10)	91 (13)	348 (8)	
Disagree	22 (1)	105 (15)	144 (17)	87 (14)	105 (11)	463 (10)	
**How much do you agree with quarantine/isolation/social distancing? "It is a necessary strategy to help control COVID-19."**	N=1,476	N=827	N=1,009	N=712	N=1,034	N=5,058	<0.001
Agree	1,383 (94)	739 (88)	853 (83)	608 (80)	846 (74)	4,429 (85)	
Neither agree nor disagree	65 (4)	12 (0)	27 (3)	28 (5)	76 (11)	208 (5)	
Disagree	28 (2)	76 (12)	129 (14)	76 (15)	112 (15)	421 (10)	

For peer review only - http://bmjopen.bmj.com/site/about/guidelines.xhtml

Suppl. Table 24 Breakdown of behavioural changes and acceptance of government public health measures by country and gender

M = male; F = female; O = other/prefer not to say. Values in cells are n (weighted %) of respondents who replied ‘yes’.

**Variable and categories**	**Thailand**			**Malaysia**			**UK**			**Italy**			**Slovenia**			**Total**			
**Gender**	**M**	**F**	**O**	**M**	**F**	**O**	**M**	**F**	**O**	**M**	**F**	**O**	**M**	**F**	**O**	**M**	**F**	**O**	**P-value (for total M vs F)**
	N=704	N=766	N=6	N=298	N=525	N=4	N=426	N=572	N=11	N=222	N=490	N=0	N=366	N=662	N=6	N=2,016	N=3,015	N=27	
**Did you change your social behaviour before the implementation of government restrictions?**	660 (94)	709 (92)	5 (83)	184 (60)	351 (68)	3 (75)	288 (64)	415 (71)	9 (82)	99 (43)	257 (52)		179 (42)	402 (51)	3 (50)	1,410 (65)	2,134 (70)	20 (74)	0.039
**If you answered 'yes' to the previous question: how did you change your social behaviour?**																			
No physical contact with anyone	(N=660) 626 (93)	(N=709) 671 (95)	(N=5) 5 (100)	(N=173) 122 (75)	(N=330) 237 (87)	(N=3) 3 (100)	(N=271) 141 (51)	(N=379) 181 (50)	(N=7) 3 (43)	(N=94) 63 (68)	(N=248) 180 (78)		(N=175) 162 (94)	(N=398) 351 (892	(N=3) 3 (100)	(N=1,373) 1,114 (80)	(N=2,064) 1,620 (83)	(N=18) 14 (78)	0.227
No physical contact only with elderly and those with serious underlying medical conditions	(N=660) 584 (88)	(N=709) 611 (89)	(N=5) 5 (100)	(N=170) 104 (59)	(N=321) 186 (67)	(N=3) 2 (67)	(N=268) 148 (58)	(N=370) 243 (62)	(N=6) 2 (33)	(N=90) 75 (75)	(N=242) 197 (81)		(N=171) 152 (88)	(N=392) 361 (94)	(N=3) 3 (100)	(N=1,359) 1,063 (77)	(N=2,034) 1,598 (81)	(N=17) 12 (71)	0.124
Going out only for essential needs	(N=660) 612 (93)	(N=709) 674 (94)	(N=5) 5 (100)	(N=177) 164 (91)	(N=345) 322 (99)	(N=3) 3 (100)	(N=277) 234 (84)	(N=396) 330 (82)	(N=8) 7 (88)	(N=95) 71 (84)	(N=251) 192 (81)		(N=172) 113 (65)	(N=387) 265 (76)	(N=3) 3 (100)	(N=1,381) 1,194 (87)	(N=2,088) 1,783 (88)	(N=19) 18 (95)	0.327
Moving home to stay with parents/relatives	(N=660) 359 (59)	(N=709) 316 (49)	(N=5) 2 (40)	(N=167) 39 (27)	(N=319) 59 (24)	(N=3) 1 (33)	(N=267) 8 (3)	(N=354) 22 (11)	(N=6) 0 (0)	(N=91) 7 (3)	(N=235) 20 (9)		(N=167) 11 (3)	(N=382) 21 (6)	(N=3) 1 (33)	(N=1,352) 424 (32)	(N=1,999) 438 (28)	(N=17) 4 (24)	0.207
Use of personal protection equipment (e.g. masks and gloves)	(N=660) 639 (97)	(N=709) 690 (95)	(N=5) 5 (100)	(N=178) 160 (96)	(N=346) 325 (95)	(N=3) 3 (100)	(N=272) 101 (33)	(N=371) 121 (33)	(N=8) 3 (38)	(N=93) 38 (59)	(N=246) 127 (52)		(N=173) 122 (73)	(N=388) 241 (63)	(N=3) 3 (100)	(N=1,376) 1,060 (78)	(N=2,060) 1,504 (74)	(N=19) 14 (74)	0.079
Use of sanitizer products and alcohol	(N=660) 628 (95)	(N=709) 688 (95)	(N=5) 5 (100)	(N=178) 167 (96)	(N=348) 334 (96)	(N=3) 3 (100)	(N=278) 223 (80)	(N=398) 329 (85)	(N=9) 7 (78)	(N=96) 80 (92)	(N=254) 227 (91)		(N=173) 164 (94)	(N=393) 354 (94)	(N=3) 3 (100)	(N=1,385) 1,262 (92)	(N=2,102) 1,932 (93)	(N=20) 18 (90)	0.474
**“I would comply with government enforced quarantine/ isolation/social distancing.”**	N=704	N=766	N=6	N=298	N=525	N=4	N=426	N=572	N=11	N=222	N=490		N=366	N=662	N=6	N=2,016	N=3,015	N=27	0.631
Agree	636 (92)	705 (93)	3 (50)	262 (93)	442 (78)	4 (100)	334 (76)	480 (85)	8 (73)	176 (69)	430 (86)		295 (75)	571 (75)	5 (83)	1,703 (82)	2,628 (84)	20 (74)	
Neither agree nor disagree	49 (6)	40 (4)	3 (50)	9 (1)	9 (0)	0 (0)	26 (6)	19 (3)	3 (27)	14 (10)	22 (5)		24 (10)	44 (17)	0 (0)	122 (6)	134 (6)	6 (22)	
Disagree	19 (2)	21 (3)	0 (0)	27 (7)	74 (22)	0 (0)	66 (18)	73 (12)	0 (0)	32 (21)	38 (9)		47 (15)	47 (8)	1 (17)	191 (11)	253 (10)	1 (4)	

For peer review only - http://bmjopen.bmj.com/site/about/guidelines.xhtml

**"I would enter voluntary quarantine/isolation/social distancing for social/self-responsibility."**	N=704	N=766	N=6	N=298	N=525	N=4	N=426	N=572	N=11	N=222	N=490		N=366	N=662	N=6	N=2,016	N=3,015	N=27	0.761
Agree	644 (91)	707 (92)	3 (50)	258 (93)	412 (68)	4 (100)	340 (78)	465 (78)	10 (91)	163 (67)	403 (85)		285 (76)	548 (77)	5 (83)	1,690 (83)	2,535 (81)	22 (81)	
Neither agree nor disagree	50 (8)	47 (7)	3 (50)	14 (1)	34 (8)	0 (0)	22 (5)	27 (5)	1 (9)	21 (14)	38 (6)		36 (9)	55 (15)	0 (0)	143 (7)	201 (8)	4 (15)	
Disagree	10 (1)	12 (1)	0 (0)	26 (6)	79 (25)	0 (0)	64 (17)	80 (16)	0 (0)	38 (19)	49 (9)		45 (15)	59 (8)	1 (17)	183 (10)	279 (10)	1 (4)	
**How much do you agree with quarantine/isolation/social distancing? "It is a necessary strategy to help control COVID-19."**	N=704	N=766	N=6	N=298	N=525	N=4	N=426	N=572	N=11	N=222	N=490		N=366	N=662	N=6	N=2,016	N=3,015	N=27	0.191
Agree	653 (93)	725 (95)	5 (83)	272 (93)	463 (83)	4 (100)	342 (77)	502 (88)	9 (82)	169 (68)	439 (91)		285 (75)	557 (74)	4 (67)	1,721 (83)	2,686 (87)	22 (81)	
Neither agree nor disagree	38 (5)	26 (3)	1 (17)	6 (0)	6 (0)	0 (0)	16 (4)	11 (3)	0 (0)	15 (9)	13 (2)		28 (7)	47 (15)	1 (17)	103 (5)	103 (5)	2 (7)	
Disagree	13 (1)	15 (2)	0 (0)	20 (6)	56 (17)	0 (0)	68 (19)	59 (10)	2 (18)	38 (23)	38 (8)		53 (18)	58 (12)	1 (17)	192 (12)	226 (9)	3 (11)	

For peer review only - http://bmjopen.bmj.com/site/about/guidelines.xhtml

Suppl. Table 25 Breakdown of behavioural changes and acceptance of government public health measures by country and <u>education level</u>

P/S = primary or lower/secondary education; T = tertiary education. Values in cells are n (weighted %) of respondents who replied 'yes'.

Variable and Categories	Thailand		Malaysia		UK		Italy		Slovenia		Total		
**Education level**	**P/S**	**T**	**P/S**	**T**	**P/S**	**T**	**P/S**	**T**	**P/S**	**T**	**P/S**	**T**	**P-value (for total)**
	N=909	N=567	N=82	N=745	N=247	N=762	N=217	N=495	N=202	N=832	N=1,657	N=3,401	
**Did you change your social behaviour before the implementation of government restrictions?**	849 (93)	525 (92)	52 (64)	486 (65)	147 (60)	565 (74)	99 (46)	257 (52)	99 (41)	485 (56)	1,246 (67)	2,318 (69)	0.369
**If you answered 'yes' to the previous question: how did you change your social behaviour?**													
No physical contact with anyone	(N=849) 816 (95)	(N=525) 486 (91)	(N=47) 41 (85)	(N=459) 321 (70)	(N=138) 80 (59)	(N=519) 245 (45)	(N=90) 67 (76)	(N=252) 176 (71)	(N=97) 92 (96)	(N=479) 424 (90)	(N=1,221) 1,096 (87)	(N=2,234) 1,652 (70)	<0.001
No physical contact only with elderly and those with serious underlying medical conditions	(N=849) 771 (90)	(N=525) 429 (81)	(N=43) 29 (64)	(N=451) 263 (59)	(N=131) 76 (58)	(N=513) 317 (61)	(N=87) 73 (77)	(N=245) 199 (82)	(N=91) 83 (93)	(N=475) 433 (90)	(N=1,201) 1,032 (81)	(N=2,209) 1,641 (74)	0.003
Going out only for essential needs	(N=849) 798 (94)	(N=525) 493 (92)	(N=49) 47 (96)	(N=476) 442 (93)	(N=143) 122 (84)	(N=538) 449 (82)	(N=93) 69 (84)	(N=253) 194 (79)	(N=93) 66 (75)	(N=469) 315 (67)	(N=1,227) 1,102 (90)	(N=2,261) 1,893 (82)	<0.001
Moving home to stay with parents/relatives	(N=849) 515 (58)	(N=525) 162 (32)	(N=42) 11 (26)	(N=447) 88 (23)	(N=131) 5 (8)	(N=496) 25 (8)	(N=84) 10 (6)	(N=242) 17 (6)	(N=91) 4 (3)	(N=461) 29 (6)	(N=1,197) 545 (37)	(N=2,171) 321 (15)	<0.001
Use of personal protection equipment (e.g. masks and gloves)	(N=849) 819 (96)	(N=525) 515 (98)	(N=49) 47 (96)	(N=478) 441 (91)	(N=136) 55 (35)	(N=515) 170 (32)	(N=89) 49 (59)	(N=250) 116 (47)	(N=94) 57 (67)	(N=470) 309 (68)	(N=1,217) 1,027 (82)	(N=2,238) 1,551 (62)	<0.001
Use of sanitizer products and alcohol	(N=849) 813 (95)	(N=525) 508 (97)	(N=48) 46 (96)	(N=481) 458 (95)	(N=142) 120 (83)	(N=543) 439 (81)	(N=94) 84 (94)	(N=256) 223 (87)	(N=96) 92 (96)	(N=473) 429 (92)	(N=1,229) 1,155 (94)	(N=2,278) 2,057 (89)	<0.001
**"I would comply with government enforced quarantine/ isolation/social distancing."**	N=909	N=567	N=82	N=745	N=247	N=762	N=217	N=495	N=202	N=832	N=1,657	N=3,401	0.315
Agree	843 (93)	501 (87)	70 (85)	638 (87)	190 (77)	632 (83)	178 (75)	428 (84)	148 (68)	723 (87)	1,429 (82)	2,922 (85)	
Neither agree nor disagree	43 (4)	49 (10)	0 (0)	18 (3)	14 (5)	34 (4)	9 (7)	27 (7)	22 (19)	46 (6)	88 (7)	174 (6)	
Disagree	23 (3)	17 (3)	12 (15)	89 (11)	43 (18)	96 (13)	30 (17)	40 (9)	32 (14)	63 (7)	140 (11)	305 (9)	
**"I would enter voluntary quarantine/isolation/social distancing for social/self-responsibility."**	N=909	N=567	N=82	N=745	N=247	N=762	N=217	N=495	N=202	N=832	N=1,657	N=3,401	0.370
Agree	842 (92)	512 (89)	65 (80)	609 (83)	180 (73)	635 (83)	165 (75)	401 (80)	151 (72)	687 (82)	1,403 (81)	2,844 (84)	
Neither agree nor disagree	55 (7)	45 (10)	3 (4)	45 (6)	17 (6)	33 (4)	24 (11)	35 (7)	24 (15)	67 (9)	123 (8)	225 (7)	
Disagree	12 (1)	10 (2)	14 (16)	91 (11)	50 (21)	94 (13)	28 (14)	59 (13)	27 (13)	78 (9)	131 (11)	332 (10)	
**How much do you agree with quarantine/isolation/social distancing? "It is a necessary strategy to help control COVID-19."**	N=909	N=567	N=82	N=745	N=247	N=762	N=217	N=495	N=202	N=832	N=1,657	N=3,401	0.304
Agree	858 (95)	525 (91)	72 (88)	667 (90)	201 (80)	652 (85)	179 (78)	429 (84)	145 (768	701 (85)	1,455 (84)	2,974 (87)	
Neither agree nor disagree	34 (4)	31 (7)	0 (0)	12 (2)	8 (4)	19 (3)	6 (5)	22 (5)	23 (14)	53 (6)	71 (5)	137 (5)	
Disagree	17 (2)	11 (2)	10 (12)	66 (8)	38 (17)	91 (12)	32 (17)	44 (10)	34 (19)	78 (9)	131 (11)	290 (9)	

Suppl. Table 26 Breakdown of behavioural changes and acceptance of government public health measures by age group

Values in cells are n (weighted %) of respondents who replied 'yes'.

Variable and categories	Thailand			Malaysia			UK			Italy			Slovenia			Total			
**Age group**	**18-34**	**35-64**	**65+**	**18-34**	**35-64**	**65+**	**18-34**	**35-64**	**65+**	**18-34**	**35-64**	**65+**	**18-34**	**35-64**	**65+**	**18-34**	**35-64**	**65+**	**P-value (for total)**
	N=223	N=1,152	N=101	N=350	N=442	N=35	N=140	N=616	N=253	N=272	N=383	N=57	N=308	N=676	N=50	N=1,293	N=3,269	N=496	
**Did you change your social behaviour before the implementation of government restrictions?**	202 (92)	1,079 (94)	93 (93)	233 (63)	287 (71)	18 (37)	104 (71)	448 (69)	160 (61)	124 (44)	202 (44)	30 (57)	178 (54)	386 (53)	20 (25)	841 (70)	2,402 (70)	321 (57)	0.004
**If you answered 'yes' to the previous question: how did you change your social behaviour?**																			
No physical contact with anyone	(N=202) 180 (91)	(N=1,079) 1,037 (96)	(N=93) 85 (90)	(N=225) 156 (84)	(N=265) 193 (80)	(N=16) 13 (81)	(N=99) 35 (43)	(N=412) 200 (51)	(N=146) 90 (61)	(N=120) 79 (72)	(N=196) 143 (74)	(N=26) 21 (75)	(N=176) 151 (87)	(N=380) 345 (94)	(N=20) 20 (100)	(N=822) 601 (78)	(N=2,332) 1,918 (84)	(N=301) 229 (82)	0.204
No physical contact only with elderly and those with serious underlying medical conditions	(N=202) 168 (88)	(N=1,079) 956 (90)	(N=93) 76 (83)	(N=218) 127 (65)	(N=261) 158 (61)	(N=15) 7 (73)	(N=98) 60 (60)	(N=416) 271 (65)	(N=130) 62 (46)	(N=120) 100 (89)	(N=187) 150 (80)	(N=25) 22 (69)	(N=174) 163 (90)	(N=374) 340 (92)	(N=18) 13 (87)	(N=812) 618 (78)	(N=2,317) 1,875 (81)	(N=281) 180 (73)	0.152
Going out only for essential needs	(N=202) 186 (94)	(N=1,079) 1,022 (95)	(N=93) 83 (89)	(N=230) 212 (98)	(N=278) 262 (94)	(N=17) 15 (82)	(N=102) 79 (76)	(N=427) 362 (86)	(N=152) 130 (86)	(N=121) 79 (68)	(N=198) 159 (79)	(N=27) 25 (99)	(N=174) 102 (55)	(N=370) 266 (75)	(N=18) 13 (87)	(N=829) 658 (85)	(N=2,352) 2,071 (88)	(N=307) 266 (89)	0.153
Moving home to stay with parents/relatives	(N=202) 88 (59)	(N=1,079) 556 (56)	(N=93) 33 (34)	(N=219) 65 (38)	(N=256) 32 (16)	(N=14) 2 (22)	(N=98) 21 (21)	(N=398) 8 (2)	(N=131) 1 (2)	(N=120) 16 (11)	(N=184) 11 (7)	(N=22) 0 (0)	(N=172) 16 (8)	(N=363) 17 (4)	(N=17) 0 (0)	(N=811) 206 (37)	(N=2,280) 624 (29)	(N=277) 36 (17)	<0.001
Use of personal protection equipment (e.g. masks and gloves)	(N=202) 198 (98)	(N=1,079) 1,050 (97)	(N=93) 86 (90)	(N=230) 212 (93)	(N=279) 262 (99)	(N=18) 14 (80)	(N=100) 23 (20)	(N=417) 157 (40)	(N=134) 45 (35)	(N=121) 48 (39)	(N=191) 100 (54)	(N=27) 17 (69)	(N=174) 88 (52)	(N=371) 260 (68)	(N=19) 18 (97)	(N=827) 569 (72)	(N=2,337) 1,829 (79)	(N=291) 180 (74)	0.067
Use of sanitizer products and alcohol	(N=202) 197 (96)	(N=1,079) 1,037 (96)	(N=93) 87 (91)	(N=230) 218 (94)	(N=281) 271 (99)	(N=18) 15 (81)	(N=102) 88 (84)	(N=436) 352 (82)	(N=147) 119 (84)	(N=122) 103 (84)	(N=199) 177 (90)	(N=29) 27 (99)	(N=174) 157 (92)	(N=377) 346 (94)	(N=18) 18 (100)	(N=830) 763 (92)	(N=2,372) 2,183 (93)	(N=305) 266 (91)	0.613
**"I would comply with government enforced quarantine/ isolation/social distancing."**	N=223	N=1,152	N=101	N=350	N=442	N=35	N=140	N=616	N=253	N=272	N=383	N=57	N=308	N=676	N=50	N=1,293	N=3,269	N=496	0.003
Agree	189 (90)	1,058 (92)	97 (96)	307 (82)	371 (88)	30 (91)	120 (85)	493 (78)	209 (80)	247 (88)	311 (77)	48 (72)	272 (85)	559 (75)	40 (65)	1,135 (86)	2,792 (83)	424 (80)	
Neither agree nor disagree	28 (8)	63 (5)	1 (1)	7 (1)	11 (1)	0 (0)	3 (1)	33 (6)	12 (5)	7 (2)	24 (5)	5 (14)	16 (7)	44 (8)	8 (34)	61 (4)	175 (5)	26 (13)	
Disagree	6 (2)	31 (3)	3 (3)	36 (18)	60 (11)	5 (9)	17 (14)	90 (17)	32 (14)	18 (10)	48 (17)	4 (14)	20 (8)	73 (17)	2 (1)	97 (10)	302 (12)	46 (8)	

**“I would enter voluntary quarantine/isolation/social distancing for social/self-responsibility.”**	N=223	N=1,152	N=101	N=350	N=442	N=35	N=140	N=616	N=253	N=272	N=383	N=57	N=308	N=676	N=50	N=1,293	N=3,269	N=496	0.327
Agree	188 (86)	1,068 (93)	98 (96)	294 (79)	353 (86)	27 (68)	114 (79)	497 (78)	204 (78)	211 (70)	306 (75)	49 (84)	247 (80)	550 (75)	41 (74)	1,054 (80)	2,774 (83)	419 (82)	
Neither agree nor disagree	33 (13)	64 (5)	3 (4)	23 (7)	23 (1)	2 (9)	6 (4)	30 (5)	14 (7)	28 (15)	28 (8)	3 (10)	28 (9)	57 (11)	6 (20)	118 (9)	202 (6)	28 (10)	
Disagree	2 (1)	20 (2)	0 (0)	33 (15)	66 (13)	6 (24)	20 (17)	89 (17)	35 (15)	33 (16)	49 (17)	5 (6)	33 (11)	69 (13)	3 (7)	121 (11)	293 (11)	49 (8)	
**How much do you agree with quarantine/isolation/social distancing? “It is a necessary strategy to help control COVID-19.”**	N=223	N=1,152	N=101	N=350	N=442	N=35	N=140	N=616	N=253	N=272	N=383	N=57	N=308	N=676	N=50	N=1,293	N=3,269	N=496	0.271
Agree	203 (93)	1,083 (94)	97 (96)	313 (85)	393 (89)	33 (100)	120 (83)	521 (83)	212 (82)	243 (86)	315 (78)	50 (79)	254 (79)	549 (76)	43 (67)	1,133 (86)	2,861 (85)	435 (82)	
Neither agree nor disagree	18 (7)	45 (4)	2 (2)	5 (0)	6 (0)	1 (0)	3 (3)	16 (3)	8 (4)	10 (4)	14 (3)	4 (11)	28 (12)	45 (7)	3 (18)	64 (5)	126 (4)	18 (8)	
Disagree	2 (0)	24 (2)	2 (2)	32 (15)	43 (11)	1 (0)	17 (14)	79 (15)	33 (14)	19 (10)	54 (19)	3 (10)	26 (10)	82 (17)	4 (15)	96 (9)	282 (11)	43 (10)	

For peer review only

For peer review only - http://bmjopen.bmj.com/site/about/guidelines.xhtml

Suppl. Table 27 Breakdown of behavioural changes and acceptance of government public health measures by self-reported level of understanding of COVID-19

H = high/very high/expert level; S = some; N = a little/none at all. Values in cells are n (weighted %) of respondents who replied 'yes'.

**Variable and categories**	**Thailand**			**Malaysia**			**UK**			**Italy**			**Slovenia**			**Total**			
**Self-reported level of understanding of COVID-19**	**H**	**S**	**N**	**H**	**S**	**N**	**H**	**S**	**N**	**H**	**S**	**N**	**H**	**S**	**N**	**H**	**S**	**N**	**P-value (for total)**
	N=965	N=459	N=52	N=435	N=359	N=33	N=647	N=336	N=26	N=368	N=324	N=20	N=713	N=279	N=42	N=3,128	N=1,757	N=173	
**Did you change your social behaviour before the implementation of government restrictions?**	898 (94)	430 (92)	46 (91)	285 (64)	232 (66)	21 (58)	468 (69)	232 (66)	12 (68)	200 (52)	146 (43)	10 (60)	429 (52)	137 (37)	18 (46)	2,280 (70)	1,177 (64)	107 (65)	0.091
**If you answered 'yes' to the previous question: how did you change your social behaviour?**																			
No physical contact with anyone	(N=898) 849 (94)	(N=430) 411 (95)	(N=46) 42 (9187)	(N=272) 204 (90)	(N=214) 143 (73)	(N=20) 15 (69)	(N=428) 221 (53)	(N=217) 99 (47)	(N=12) 5 (52)	(N=194) 137 (78)	(N=138) 99 (67)	(N=10) 7 (88)	(N=423) 380 (95)	(N=135) 119 (87)	(N=18) 17 (96)	(N=2,215) 1,791 (85)	(N=1,134) 871 (77)	(N=106) 86 (78)	0.033
No physical contact only with elderly and those with serious underlying medical conditions	(N=898) 765 (87)	(N=430) 394 (92)	(N=46) 41 (87)	(N=266) 162 (63)	(N=209) 119 (60)	(N=19) 11 (74)	(N=417) 261 (61)	(N=215) 128 (59)	(N=12) 4 (49)	(N=192) 163 (85)	(N=130) 101 (67)	(N=10) 8 (94)	(N=418) 379 (91)	(N=131) 122 (92)	(N=17) 15 (95)	(N=2,191) 1,730 (80)	(N=1,115) 864 (77)	(N=104) 79 (79)	0.744
Going out only for essential needs	(N=898) 844 (93)	(N=430) 405 (95)	(N=46) 42 (87)	(N=280) 266 (99)	(N=225) 205 (89)	(N=20) 18 (99)	(N=444) 381 (86)	(N=225) 182 (80)	(N=12) 8 (66)	(N=196) 145 (80)	(N=140) 109 (83)	(N=10) 9 (95)	(N=415) 283 (72)	(N=129) 87 (73)	(N=18) 11 (60)	(N=2,233) 1,919 (88)	(N=1,149) 988 (87)	(N=106) 88 (84)	0.711
Moving home to stay with parents/relatives	(N=898) 345 (45)	(N=430) 298 (67)	(N=46) 34 (73)	(N=261) 45 (24)	(N=209) 48 (25)	(N=19) 6 (40)	(N=404) 17 (5)	(N=212) 12 (10)	(N=11) 1 (24)	(N=189) 17 (6)	(N=127) 9 (7)	(N=10) 1 (10)	(N=405) 19 (3)	(N=129) 14 (9)	(N=18) 0 (0)	(N=2,157) 443 (25)	(N=1,107) 381 (36)	(N=104) 42 (42)	<0.001
Use of personal protection equipment (e.g. masks and gloves)	(N=898) 874 (97)	(N=430) 418 (96)	(N=46) 42 (81)	(N=280) 266 (99)	(N=227) 203 (90)	(N=20) 19 (99)	(N=421) 153 (38)	(N=218) 68 (28)	(N=12) 4 (17)	(N=194) 90 (46)	(N=135) 69 (66)	(N=10) 6 (66)	(N=416) 289 (71)	(N=130) 71 (59)	(N=18) 6 (38)	(N=2,209) 1,672 (78)	(N=1,140) 829 (74)	(N=106) 77 (69)	0.172
Use of sanitizer products and alcohol	(N=898) 863 (96)	(N=430) 416 (95)	(N=46) 42 (81)	(N=281) 270 (99)	(N=228) 215 (91)	(N=20) 19 (100)	(N=447) 374 (85)	(N=226) 179 (85)	(N=12) 6 (30)	(N=198) 170 (90)	(N=142) 129 (93)	(N=10) 8 (94)	(N=418) 385 (95)	(N=133) 125 (95)	(N=18) 11 (70)	(N=2,242) 2,062 (94)	(N=1,159) 1,064 (92)	(N=106) 86 (78)	<0.001

For peer review only - http://bmjopen.bmj.com/site/about/guidelines.xhtml

**"I would comply with government enforced quarantine/ isolation/social distancing."**	N=965	N=459	N=52	N=435	N=359	N=33	N=647	N=336	N=26	N=368	N=324	N=20	N=713	N=279	N=42	N=3,128	N=1,757	N=173	0.370
Agree	903 (95)	402 (88)	39 (81)	378 (93)	305 (79)	25 (76)	511 (79)	291 (83)	20 (87)	303 (76)	284 (79)	19 (97)	607 (75)	232 (75)	32 (70)	2,702 (85)	1,514 (82)	135 (80)	
Neither agree nor disagree	39 (3)	44 (9)	9 (10)	5 (0)	9 (1)	4 (1)	29 (3)	18 (6)	1 (2)	17 (4)	18 (11)	1 (3)	45 (16)	19 (10)	4 (7)	135 (6)	108 (7)	19 (4)	
Disagree	23 (2)	13 (3)	4 (9)	52 (7)	45 (20)	4 (23)	107 (18)	27 (12)	5 (11)	48 (21)	22 (10)	0 (0)	61 (9)	28 (15)	6 (24)	291 (10)	135 (11)	19 (16)	
**"I would enter voluntary quarantine/isolation/social distancing for social/self-responsibility."**	N=965	N=459	N=52	N=435	N=359	N=33	N=647	N=336	N=26	N=368	N=324	N=20	N=713	N=279	N=42	N=3,128	N=1,757	N=173	0.091
Agree	909 (95)	401 (85)	44 (90)	357 (86)	294 (76)	23 (75)	516 (78)	284 (80)	15 (60)	293 (78)	258 (74)	15 (91)	587 (78)	219 (74)	32 (69)	2,662 (84)	1,456 (79)	129 (77)	
Neither agree nor disagree	41 (4)	51 (13)	8 (10)	21 (1)	21 (10)	6 (1)	29 (5)	18 (5)	3 (8)	27 (8)	30 (12)	2 (6)	58 (14)	26 (9)	7 (23)	176 (6)	146 (10)	26 (8)	
Disagree	15 (1)	7 (1)	0 (0)	57 (13)	44 (14)	4 (23)	102 (17)	34 (15)	8 (32)	48 (15)	36 (13)	3 (4)	68 (9)	34 (17)	3 (7)	290 (9)	155 (11)	18 (15)	
**How much do you agree with quarantine/isolation/social distancing? "It is a necessary strategy to help control COVID-19."**	N=965	N=459	N=52	N=435	N=359	N=33	N=647	N=336	N=26	N=368	N=324	N=20	N=713	N=279	N=42	N=3,128	N=1,757	N=173	0.688
Agree	920 (96)	418 (91)	45 (90)	392 (91)	319 (85)	28 (86)	540 (82)	293 (83)	20 (85)	304 (77)	285 (82)	19 (82)	589 (73)	226 (78)	31 (72)	2,745 (85)	1,541 (85)	143 (84)	
Neither agree nor disagree	26 (2)	33 (8)	6 (8)	5 (0)	5 (0)	2 (1)	16 (3)	10 (3)	1 (2)	10 (2)	18 (9)	0 (0)	45 (12)	27 (9)	4 (7)	102 (4)	93 (6)	13 (4)	
Disagree	19 (1)	8 (2)	1 (2)	38 (9)	35 (15)	3 (13)	91 (15)	33 (13)	5 (13)	54 (21)	21 (9)	1 (18)	79 (16)	26 (13)	7 (21)	281 (11)	123 (10)	17 (12)	

For peer review only - http://bmjopen.bmj.com/site/about/guidelines.xhtml

Suppl. Table 28 Breakdown of self-reported level of understanding of COVID-19 by country

Values in cells are n (weighted %) of respondents who replied 'yes'.

**Variable and categories**	**Thailand**	**Malaysia**	**UK**	**Italy**	**Slovenia**	**Total**	**P-value**
	N=1,476	N=827	N=1,009	N=712	N=1,034	N=5,058	<0.001
**High/very high/expert level understanding**	965 (63)	435 (51)	647 (59)	368 (47)	713 (66)	3,128 (59)	
**Some understanding**	459 (33)	359 (38)	336 (38)	324 (50)	279 (30)	1,757 (36)	
**A little/none at all**	52 (4)	33 (11)	26 (4)	20 (3)	42 (4)	173 (5)	

For peer review only

Suppl. Table 29 Breakdown of self-reported level of understanding of COVID-19 by demographic characteristics

H = high/very high/expert level; S = some; N = a little/none at all. Values in cells are n (weighted %) of respondents who replied 'yes'.

Variable and categories	Thailand			Malaysia			UK			Italy			Slovenia			Total			
**Self-reported understanding of COVID-19**	**H**	**S**	**N**	**H**	**S**	**N**	**H**	**S**	**N**	**H**	**S**	**N**	**H**	**S**	**N**	**H**	**S**	**N**	**P-value (for total)**
**Gender**																			0.058
Male	458 (65)	224 (31)	22 (4)	153 (55)	130 (30)	15 (15)	280 (61)	134 (35)	12 (4)	130 (51)	87 (46)	5 (3)	269 (64)	84 (31)	13 (5)	1,290 (60)	659 (34)	67 (6)	
Female	504 (61)	232 (35)	30 (4)	280 (47)	228 (46)	17 (7)	358 (56)	200 (40)	14 (3)	238 (44)	237 (53)	15 (3)	439 (68)	194 (29)	29 (3)	1,819 (57)	1,091 (39)	105 (4)	
Other/prefer not to say	3 (50)	3 (50)	0 (0)	2 (50)	1 (25)	1 (25)	9 (82)	2 (18)	0 (0)				5 (83)	1 (17)	0 (0)	19 (70)	7 (26)	1 (4)	
**Age group**																			0.033
18-34	143 (62)	69 (34)	11 (4)	170 (48)	167 (48)	13 (9)	74 (44)	58 (48)	8 (8)	119 (39)	143 (57)	10 (5)	186 (59)	106 (35)	16 (6)	692 (52)	543 (41)	58 (6)	
35-64	746 (62)	371 (35)	35 (3)	244 (54)	179 (32)	19 (14)	411 (67)	193 (32)	12 (2)	220 (54)	153 (42)	10 (4)	492 (69)	158 (27)	26 (5)	2,113 (62)	1,054 (33)	102 (5)	
65+	76 (68)	19 (25)	6 (7)	21 (52)	13 (42)	1 (6)	162 (59)	85 (39)	6 (2)	29 (42)	28 (58)	0 (0)	35 (68)	15 (32)	0 (0)	323 (60)	160 (38)	13 (3)	
**Education level**																			<0.001
Primary or lower/secondary	537 (60)	341 (36)	31 (4)	42 (51)	30 (36)	10 (13)	140 (52)	101 (44)	6 (4)	92 (43)	114 (53)	11 (4)	124 (63)	67 (33)	11 (4)	935 (56)	653 (39)	69 (6)	
Tertiary	428 (74)	118 (22)	21 (4)	393 (51)	329 (46)	23 (3)	507 (64)	235 (32)	20 (3)	276 (58)	210 (41)	9 (2)	589 (71)	212 (26)	31 (3)	2,193 (66)	1,104 (31)	104 (3)	
**Healthcare worker status**																			0.001
Healthcare worker	172 (72)	59 (26)	8 (3)	128 (49)	79 (50)	6 (1)	90 (76)	24 (21)	4 (3)	45 (67)	18 (29)	1 (4)	291 (78)	44 (21)	6 (1)	726 (70)	224 (28)	25 (2)	
Non-healthcare worker	793 (61)	400 (33)	44 (4)	307 (52)	280 (35)	27 (13)	557 (57)	312 (39)	22 (4)	323 (46)	306 (50)	19 (3)	422 (63)	235 (32)	36 (5)	2,402 (57)	1,533 (38)	148 (5)	

Suppl. Table 30 Breakdown of self-reported understanding of public health measures by self-reported level of understanding of COVID-19

(H = high/very high/expert level; S = some; N = a little/none at all). Values in cells are n (weighted %) of respondents who replied ‘yes’.

Variable and categories	Thailand			Malaysia			UK			Italy			Slovenia			Total			
**Self-reported level of understanding of COVID-19**	**H**	**S**	**N**	**H**	**S**	**N**	**H**	**S**	**N**	**H**	**S**	**N**	**H**	**S**	**N**	**H**	**S**	**N**	**P-value**
**How would you rate your level of understanding of the current quarantine/isolation/social distancing requirements for COVID-19?**	N=965	N=459	N=52	N=435	N=359	N=33	N=647	N=336	N=26	N=368	N=324	N=20	N=713	N=279	N=42	N=3,128	N=1,757	N=173	<0.001
H	855 (89)	116 (23)	19 (24)	399 (89)	193 (52)	9 (21)	532 (81)	182 (57)	8 (21)	338 (93)	213 (71)	7 (36)	652 (89)	212 (59)	24 (46)	2,776 (88)	916 (50)	67 (27)	
S	102 (10)	323 (71)	11 (12)	31 (7)	157 (39)	15 (52)	98 (15)	129 (35)	11 (46)	22 (5)	106 (28)	10 (38)	50 (10)	55 (32)	12 (44)	303 (10)	770 (43)	59 (39)	
N	8 (1)	20 (6)	22 (64)	5 (4)	9 (9)	9 (27)	17 (4)	25 (8)	7 (33)	8 (2)	5 (1)	3 (26)	11 (1)	12 (9)	6 (10)	49 (2)	71 (6)	47 (34)	

For peer review only

Suppl. Table 31 What were the three most common ways people received communication on COVID-19, and what are the three most preferred ways to receive COVID-19 communications? Breakdown by country

Values in cells are n (weighted %) of respondents who replied 'yes'.

**Variable and categories**	**Thailand**	**Malaysia**	**UK**	**Italy**	**Slovenia**	**Total**	**P-value**
	N=1,476	N=827	N=1,009	N=712	N=1,034	N=5,058	
**How do/did you receive information about COVID-19?**							
Face-to-face (e.g. doctors or health workers)	1,096 (78)	275 (19)	155 (15)	276 (32)	413 (34)	2,215 (40)	<0.001
Traditional media (TV, radio, newspapers)	1,407 (95)	795 (93)	940 (93)	650 (85)	994 (95)	4,786 (93)	0.012
Print materials (leaflets, brochures)	803 (55)	256 (32)	403 (36)	119 (23)	479 (43)	2,060 (40)	<0.001
Online (websites, email)	1,101 (69)	779 (90)	918 (89)	651 (88)	964 (87)	4,413 (83)	<0.001
Social media and messenger apps	1,279 (83)	786 (95)	773 (77)	528 (75)	731 (66)	4,097 (79)	<0.001
Government/institution's web page	1,134 (74)	682 (75)	698 (70)	580 (79)	784 (60)	3,878 (71)	<0.001
WHO web page	367 (20)	550 (56)	380 (36)	334 (39)	397 (30)	2,028 (34)	<0.001
**How would you prefer to receive information about COVID-19?**							
Face-to-face (e.g doctors or health workers)	1,200 (83)	417 (44)	361 (36)	584 (77)	577 (55)	3,139 (61)	<0.001
Traditional media (TV, radio, newspapers)	1,347 (90)	759 (91)	648 (64)	467 (62)	806 (76)	4,027 (78)	<0.001
Print materials	893 (63)	340 (40)	418 (41)	149 (29)	481 (52)	2,281 (48)	<0.001
Online (websites, email)	1,105 (71)	742 (88)	812 (75)	473 (71)	856 (79)	3,988 (76)	<0.001
Social media and messenger apps	1,245 (82)	659 (85)	330 (31)	292 (50)	470 (50)	2,996 (61)	<0.001
Government/institution's web page	1,181 (77)	731 (86)	741 (74)	605 (77)	845 (71)	4,103 (77)	0.009
WHO web page	586 (36)	703 (82)	609 (58)	531 (64)	678 (55)	3,107 (56)	<0.001

For peer review only

Suppl. Table 32 What were the three most common ways people received communications on COVID-19, and what are the three most preferred ways to receive COVID-19 communications? Breakdown by country and gender

M = male; F = female; O = other/prefer not to say. Values in cells are n (weighted %) of respondents who replied 'yes'.

**Variable and categories**	**Thailand**			**Malaysia**			**UK**			**Italy**			**Slovenia**			**Total**			
**Gender**	**M**	**F**	**O**	**M**	**F**	**O**	**M**	**F**	**O**	**M**	**F**	**O**	**M**	**F**	**O**	**M**	**F**	**O**	**P-value (for total M vs F)**
	N=704	N=766	N=6	N=298	N=525	N=4	N=426	N=572	N=11	N=222	N=490	N=0	N=366	N=662	N=6	N=2,016	N=3,015	N=27	
**How do/did you receive information about COVID-19?**																			
Face-to-face	563 (81)	529 (75)	4 (67)	93 (17)	180 (21)	2 (50)	68 (16)	84 (14)	3 (27)	82 (29)	194 (34)		126 (31)	285 (37)	2 (33)	932 (40)	1,272 (41)	11 (41)	0.591
Traditional media (TV, radio, newspapers)	669 (94)	732 (96)	6 (100)	284 (92)	507 (93)	4 (100)	390 (92)	539 (95)	11 (100)	199 (82)	451 (88)		353 (98)	635 (93)	6 (100)	1,895 (92)	2,864 (94)	27 (100)	0.468
Print materials (leaflets, brochures)	398 (54)	402 (56)	3 (50)	94 (37)	162 (26)	0 (0)	171 (37)	227 (36)	5 (45)	31 (27)	88 (20)		168 (44)	307 (41)	4 (67)	862 (42)	1,186 (39)	12 (44)	0.265
Online (websites, email)	509 (69)	586 (69)	6 (100)	281 (92)	495 (89)	3 (75)	379 (87)	528 (91)	11 (100)	201 (85)	450 (90)		336 (84)	622 (90)	6 (100)	1,706 (82)	2,681 (84)	26 (96)	0.332
Social media and messenger apps	595 (84)	678 (82)	6 (100)	281 (96)	502 (94)	3 (75)	312 (74)	450 (79)	11 (100)	154 (70)	374 (80)		256 (66)	470 (67)	5 (83)	1,598 (78)	2,474 (80)	25 (93)	0.589
Government/institution's web page	540 (73)	589 (74)	5 (83)	246 (80)	432 (69)	4 (100)	282 (69)	409 (71)	7 (64)	170 (74)	410 (83)		260 (59)	518 (61)	6 (100)	1,498 (71)	2,358 (71)	22 (81)	0.881
WHO web page	150 (18)	214 (22)	3 (50)	173 (52)	374 (60)	3 (75)	136 (34)	239 (39)	5 (45)	81 (27)	253 (50)		108 (26)	286 (33)	3 (50)	648 (30)	1,366 (38)	14 (52)	0.003
**How would you prefer to receive information about COVID-19?**																			
Face-to-face	594 (85)	603 (82)	3 (50)	146 (39)	270 (50)	1 (25)	163 (36)	195 (37)	3 (27)	171 (75)	413 (79)		182 (53)	389 (57)	6 (100)	1,256 (59)	1,870 (63)	13 (48)	0.209
Traditional media (TV, radio, newspapers)	644 (89)	697 (91)	6 (100)	267 (91)	488 (92)	4 (100)	278 (66)	365 (63)	5 (45)	134 (57)	333 (67)		274 (76)	530 (77)	2 (33)	1,597 (77)	2,413 (79)	17 (63)	0.395
Print materials	446 (65)	442 (61)	5 (83)	115 (39)	223 (41)	2 (50)	177 (41)	237 (41)	4 (36)	46 (33)	103 (25)		165 (53)	314 (51)	2 (33)	949 (49)	1,319 (47)	13 (48)	0.408
Online (websites, email)	516 (70)	583 (71)	6 (100)	269 (92)	469 (83)	4 (100)	334 (71)	470 (78)	8 (73)	151 (72)	322 (70)		290 (74)	561 (84)	5 (83)	1,560 (75)	2,405 (77)	23 (85)	0.403
Social media and messenger apps	589 (84)	650 (80)	6 (100)	239 (85)	416 (87)	4 (100)	134 (29)	195 (34)	1 (9)	88 (52)	204 (48)		161 (43)	307 (57)	2 (33)	1,211 (60)	1,772 (63)	13 (48)	0.364
Government/institution's web page	575 (78)	601 (75)	5 (83)	270 (93)	457 (79)	4 (100)	293 (69)	440 (78)	8 (73)	181 (73)	424 (82)		278 (64)	561 (77)	6 (100)	1,597 (75)	2,483 (78)	23 (85)	0.335
WHO web page	248 (36)	334 (36)	4 (67)	242 (80)	457 (83)	4 (100)	234 (54)	370 (62)	5 (45)	143 (54)	388 (74)		209 (49)	466 (60)	3 (50)	1,076 (52)	2,015 (59)	16 (59)	0.020

Suppl. Table 33 What were the three most common ways people received communications on COVID-19, and what are the three most preferred ways to receive COVID-19 communications? Breakdown by country and age group

Values in cells are n (weighted %) of respondents who replied ‘yes’.

**Variable and categories**	**Thailand**			**Malaysia**			**UK**			**Italy**			**Slovenia**			**Total**			
**Age group**	**18-34**	**35-64**	**65+**	**18-34**	**35-64**	**65+**	**18-34**	**35-64**	**65+**	**18-34**	**35-64**	**65+**	**18-34**	**35-64**	**65+**	**18-34**	**35-64**	**65+**	**P-value (for total)**
	N=223	N=1,152	N=101	N=350	N=442	N=35	N=140	N=616	N=253	N=272	N=383	N=57	N=308	N=676	N=50	N=1,293	N=3,269	N=496	
**How do/did you receive information about COVID-19?**																			
Face-to-face	125 (68)	892 (82)	79 (82)	141 (20)	124 (16)	10 (23)	25 (17)	107 (17)	23 (8)	112 (37)	152 (34)	12 (23)	111 (32)	282 (30)	20 (48)	514 (37)	1,557 (42)	144 (40)	0.424
Traditional media (TV, radio, newspapers)	210 (94)	1,099 (95)	98 (96)	337 (89)	424 (95)	34 (100)	130 (93)	567 (92)	243 (97)	247 (92)	352 (90)	51 (70)	299 (98)	647 (96)	48 (91)	1,223 (93)	3,089 (94)	474 (90)	0.336
Print materials (leaflets, brochures)	107 (54)	652 (59)	44 (44)	104 (31)	146 (35)	6 (20)	34 (22)	258 (40)	111 (43)	34 (12)	71 (19)	14 (41)	140 (45)	319 (46)	20 (31)	419 (37)	1,446 (43)	195 (38)	0.106
Online (websites, email)	199 (84)	853 (71)	49 (35)	328 (86)	418 (94)	33 (91)	129 (89)	575 (92)	214 (82)	242 (90)	358 (89)	51 (82)	289 (93)	632 (91)	43 (74)	1,187 (87)	2,836 (85)	390 (69)	<0.001
Social media and messenger apps	206 (91)	1,008 (86)	65 (55)	329 (93)	424 (98)	33 (91)	104 (76)	485 (78)	184 (74)	214 (79)	274 (73)	40 (77)	243 (80)	462 (70)	26 (42)	1,096 (86)	2,653 (81)	348 (63)	<0.001
Government/institution’s web page	166 (73)	902 (78)	66 (61)	298 (71)	360 (81)	24 (61)	108 (77)	459 (74)	131 (53)	219 (73)	318 (81)	43 (78)	226 (68)	528 (71)	30 (29)	1,017 (72)	2,567 (77)	294 (54)	<0.001
WHO web page	100 (31)	256 (19)	11 (6)	260 (62	274 (53)	16 (39)	60 (45)	271 (40)	49 (18)	129 (39)	176 (38)	29 (42)	127 (39)	255 (30)	15 (19)	676 (44)	1,232 (33)	120 (22)	<0.001
**How would you prefer to receive information about COVID-19?**																			
Face-to-face	152 (77)	965 (87)	83 (84)	198 (53)	203 (34)	16 (53)	48 (33)	218 (37)	95 (39)	230 (78)	313 (80)	41 (71)	187 (57)	365 (53)	25 (59)	815 (59)	2,064 (61)	260 (62)	0.785
Traditional media (TV, radio, newspapers)	194 (85)	1,056 (91)	97 (93)	327 (90)	402 (91)	30 (99)	89 (65)	396 (64)	163 (64)	179 (60)	247 (58)	41 (72)	228 (73)	534 (75)	44 (83)	1,017 (78)	2,635 (78)	375 (80)	0.712
Print materials	118 (64)	720 (65)	55 (54)	143 (41)	179 (37)	18 (45)	40 (27)	256 (44)	122 (52)	43 (15)	88 (24)	18 (50)	149 (50)	308 (48)	24 (63)	493 (44)	1,551 (48)	237 (54)	0.073
Online (websites, email)	187 (83)	867 (73)	51 (41)	312 (87)	399 (91)	31 (77)	98 (59)	522 (84)	192 (74)	180 (74)	253 (68)	40 (75)	250 (79)	567 (83)	39 (71)	1,027 (78)	2,608 (79)	353 (66)	<0.001
Social media and messenger apps	196 (91)	986 (85)	63 (55)	285 (88)	349 (86)	25 (75)	34 (21)	219 (37)	77 (31)	105 (38)	156 (48)	31 (65)	134 (48)	317 (51)	19 (49)	754 (64)	2,027 (64)	215 (52)	0.005
Government/institution’s web page	177 (79)	936 (80)	68 (60)	323 (93)	381 (81)	27 (82)	108 (71)	468 (77)	165 (71)	235 (83)	325 (82)	45 (65)	252 (75)	557 (76)	36 (56)	1,095 (81)	2,667 (79)	341 (64)	<0.001
WHO web page	145 (55)	415 (31)	26 (20)	320 (92)	357 (72)	26 (77)	98 (65)	387 (60)	124 (46)	226 (79)	266 (64)	39 (53)	231 (73)	427 (59)	20 (26)	1,020 (72)	1,852 (53)	235 (39)	<0.001

Suppl. Table 34 What were the three most common ways people received communications on COVID-19, and what are the three most preferred ways to receive COVID-19 communications? Breakdown by country and education level

P/S = primary or lower/secondary education; T = tertiary education. Values in cells are n (weighted %) of respondents who replied 'yes'.

Variable and categories	Thailand		Malaysia		UK		Italy		Slovenia		Total		
**Education level**	**P/S**	**T**	**P/S**	**T**	**P/S**	**T**	**P/S**	**T**	**P/S**	**T**	**P/S**	**T**	**P-value (for total)**
	N=909	N=567	N=82	N=745	N=247	N=762	N=217	N=495	N=202	N=832	N=1,657	N=3,401	
**How do/did you receive information about COVID-19?**													
Face-to-face	781 (83)	315 (55)	13 (14)	262 (37)	32 (14)	123 (16)	72 (28)	204 (39)	48 (29)	365 (43)	946 (43)	1,269 (35)	<0.001
Traditional media (TV, radio, newspapers)	865 (95)	542 (95)	76 (92)	719 (97)	234 (95)	706 (92)	192 (82)	458 (93)	196 (95)	798 (96)	1,563 (92)	3,223 (94)	0.155
Print materials (leaflets, brochures)	547 (57)	256 (45)	26 (32)	230 (31)	90 (34)	313 (38)	39 (26)	80 (16)	91 (40)	388 (47)	793 (42)	1,267 (38)	0.062
Online (websites, email)	605 (65)	496 (87)	74 (89)	705 (95)	212 (85)	706 (93)	190 (85)	461 (93)	179 (83)	785 (94)	1,260 (79)	3,153 (92)	<0.001
Social media and messenger apps	757 (81)	522 (91)	78 (95)	708 (94)	196 (79)	577 (75)	173 (78)	355 (70)	150 (65)	581 (68)	1,354 (80)	2,743 (77)	0.146
Government/institution's web page	689 (73)	445 (78)	59 (73)	623 (85)	171 (70)	527 (71)	166 (77)	414 (81)	123 (49)	661 (78)	1,208 (69)	2,670 (77)	<0.001
WHO web page	139 (15)	228 (42)	44 (53)	506 (67)	68 (30)	312 (42)	84 (35)	250 (49)	59 (24)	338 (39)	394 (29)	1,634 (44)	<0.001
**How would you prefer to receive information about COVID-19?**													
Face-to-face	806 (87)	394 (68)	36 (42)	381 (53)	104 (39)	257 (34)	170 (75)	414 (81)	111 (56)	466 (54)	1,227 (65)	1,912 (53)	<0.001
Traditional media (TV, radio, newspapers)	830 (90)	517 (90)	75 (91)	684 (92)	149 (63)	499 (66)	133 (60)	334 (68)	145 (74)	661 (80)	1,332 (79)	2,695 (76)	0.100
Print materials	608 (66)	285 (49)	35 (40)	305 (40)	126 (47)	292 (37)	48 (32)	101 (21)	105 (57)	376 (45)	922 (52)	1,359 (39)	<0.001
Online (websites, email)	632 (68)	473 (82)	71 (87)	671 (90)	186 (68)	626 (81)	156 (74)	317 (64)	160 (77)	696 (83)	1,205 (74)	2,783 (80)	<0.001
Social media and messenger apps	753 (81)	492 (86)	72 (87)	587 (79)	90 (32)	240 (31)	106 (55)	186 (38)	111 (55)	359 (42)	1,132 (67)	1,864 (49)	<0.001
Government/institution's web page	711 (75)	470 (83)	69 (86)	662 (90)	194 (75)	547 (72)	173 (74)	432 (86)	138 (63)	707 (84)	1,285 (75)	2,818 (81)	0.001
WHO web page	246 (30)	340 (61)	66 (81)	637 (85)	122 (50)	487 (65)	149 (60)	382 (74)	123 (49)	555 (64)	706 (50)	2,401 (67)	<0.001

Suppl. Table 35 Most prevalent topic areas with unclear or conflicting COVID-19 information, and most prevalent 'fake news', breakdown by country

Values in cells are n (weighted %) of respondents who replied 'yes'.

Variable and categories	Thailand	Malaysia	UK	Italy	Slovenia	Total	P-value
	N=1,476	N=827	N=1,009	N=712	N=1,034	N=5,058	
**Have you seen any unclear or conflicting information about COVID-19 in the last month?**							
Ways to avoid the infection	564 (36)	409 (47)	679 (68)	410 (64)	682 (64)	2,744 (54)	<0.001
Symptoms of COVID-19	568 (36)	353 (42)	590 (62)	328 (44)	494 (44)	2,333 (45)	<0.001
What to do in case of symptoms	506 (34)	295 (37)	438 (43)	293 (45)	435 (42)	1,967 (40)	0.058
Social distancing guidance	490 (33)	292 (42)	568 (56)	314 (42)	559 (51)	2,223 (44)	<0.001
Quarantine/isolation	529 (36)	314 (39)	547 (54)	292 (41)	559 (52)	2,241 (44)	<0.001
Penalties if disobey restrictions	614 (41)	384 (42)	620 (60)	378 (52)	508 (45)	2,504 (47)	<0.001
Risks in case of infection	527 (34)	327 (37)	542 (54)	330 (49)	493 (46)	2,219 (43)	<0.001
Numbers of coronavirus cases/deaths related to COVID-19	563 (37)	284 (47)	741 (72)	457 (66)	463 (46)	2,508 (52)	<0.001
Government support schemes (e.g. financial)	779 (51)	432 (53)	438 (46)	492 (69)	572 (51)	2,713 (53)	<0.001
Testing	531 (34)	376 (39)	734 (72)	520 (72)	534 (49)	2,695 (51)	<0.001
Travel restrictions (e.g. curfew, restricted hours of movement)	520 (33)	407 (43)	641 (62)	382 (55)	533 (45)	2,483 (46)	<0.001
**Have you come across news about the following COVID-19 topics that seemed fake to you?**							
General spread of fear	668 (42)	606 (70)	693 (72)	382 (58)	771 (69)	3,120 (60)	<0.001
Coronavirus as an engineered modified virus	543 (32)	613 (65)	819 (81)	613 (82)	864 (75)	3,452 (63)	<0.001
Minimisation of risks	440 (27)	416 (39)	579 (55)	540 (69)	731 (62)	2,706 (48)	<0.001
Numbers of infected/deceased people	512 (33)	400 (47)	615 (61)	475 (75)	574 (54)	2,576 (51)	<0.001
Unreasonable health recommendations	517 (32)	545 (55)	574 (57)	385 (50)	650 (60)	2,671 (49)	<0.001
Pharmaceutical conspiracy	490 (32)	440 (50)	525 (54)	489 (63)	673 (61)	2,617 (49)	<0.001
Home-made recipes to make sanitizer products	538 (32)	573 (61)	557 (56)	516 (70)	603 (51)	2,787 (51)	<0.001
Alternative drugs/cure	537 (33)	581 (60)	697 (67)	444 (58)	612 (51)	2,871 (51)	<0.001
Fear toward products coming from infected countries	458 (29)	549 (63)	483 (49)	425 (56)	519 (48)	2,434 (46)	<0.001

For peer review only - http://bmjopen.bmj.com/site/about/guidelines.xhtml

Suppl. Table 36 Most prevalent topic areas with unclear or conflicting COVID-19 information, and most prevalent 'fake news', breakdown by country and education level

P/S = primary or lower/secondary education; T = tertiary education. Values in cells are n (weighted %) of respondents who replied 'yes'.

**Variable and categories**	**Thailand**		**Malaysia**		**UK**		**Italy**		**Slovenia**		**Total**		
**Education level**	**P/S**	**T**	**P/S**	**T**	**P/S**	**T**	**P/S**	**T**	**P/S**	**T**	**P/S**	**T**	**P-value (for total)**
	N=909	N=567	N=82	N=745	N=247	N=762	N=217	N=495	N=202	N=832	N=1,657	N=3,401	
**Have you seen any unclear or conflicting information about COVID-19 in the last month?**													
Ways to avoid the infection	276 (33)	288 (51)	37 (46)	372 (49)	153 (66)	526 (69)	119 (65)	291 (60)	125 (63)	557 (67)	710 (50)	2,034 (62)	<0.001
Symptoms	268 (33)	300 (53)	36 (43)	317 (41)	146 (65)	444 (59)	94 (42)	234 (48)	96 (44)	398 (46)	640 (42)	1,693 (51)	<0.001
What to do in case of symptoms	245 (31)	261 (47)	32 (38)	263 (36)	96 (42)	342 (44)	94 (46)	199 (43)	80 (42)	355 (41)	547 (38)	1,420 (43)	0.026
Social distancing guidance	249 (31)	241 (42)	36 (44)	256 (34)	113 (51)	455 (61)	92 (41)	222 (46)	109 (50)	450 (53)	599 (41)	1,624 (51)	<0.001
Quarantine/isolation	278 (34)	251 (45)	32 (40)	282 (38)	123 (51)	424 (56)	84 (41)	208 (43)	102 (50)	457 (55)	619 (41)	1,622 (50)	<0.001
Penalties if disobey restrictions	315 (38)	299 (52)	34 (40)	350 (48)	143 (56)	477 (62)	103 (50)	275 (56)	101 (44)	407 (47)	696 (44)	1,808 (55)	<0.001
Risks in case of infection	257 (31)	270 (49)	32 (36)	295 (39)	127 (54)	415 (55)	105 (50)	225 (46)	93 (45)	400 (47)	614 (40)	1,605 (49)	<0.001
Numbers of coronavirus cases/deaths related to COVID-19	284 (33)	279 (52)	42 (50)	242 (33)	172 (70)	569 (74)	140 (67)	317 (65)	107 (50)	356 (41)	745 (49)	1,763 (56)	0.001
Government support schemes (e.g. financial)	402 (47)	377 (69)	44 (54)	388 (52)	103 (50)	335 (43)	138 (69)	354 (71)	108 (50)	464 (54)	795 (52)	1,918 (55)	0.257
Testing	258 (31)	273 (49)	31 (38)	345 (45)	161 (68)	573 (75)	145 (70)	375 (76)	95 (48)	439 (51)	690 (46)	2,005 (62)	<0.001
Travel restrictions (e.g. curfew, restricted hours of movement)	248 (30)	272 (49)	36 (42)	371 (49)	142 (59)	499 (65)	112 (55)	270 (55)	96 (41)	437 (51)	634 (42)	1,849 (56)	<0.001
**Have you come across news about the following COVID-19 topics that seemed fake to you?**													
General spread of fear	308 (37)	360 (64)	56 (69)	550 (73)	182 (76)	511 (68)	116 (60)	266 (54)	147 (66)	624 (74)	809 (57)	2,311 (67)	<0.001
Coronavirus as an engineered modified virus	209 (26)	334 (61)	52 (62)	561 (76)	193 (80)	626 (82)	174 (80)	439 (89)	156 (70)	708 (84)	784 (56)	2,668 (79)	<0.001
Minimisation of risks	178 (23)	262 (47)	31 (36)	385 (51)	128 (52)	451 (59)	141 (63)	399 (81)	122 (56)	609 (71)	600 (41)	2,106 (62)	<0.001
Numbers of infected/deceased people	231 (29)	281 (51)	40 (47)	360 (49)	152 (62)	463 (61)	153 (719	322 (67)	118 (55)	456 (54)	694 (49)	1,882 (57)	<0.001
Unreasonable health recommendations	204 (27)	313 (57)	45 (52)	500 (66)	131 (55)	443 (59)	101 (46)	284 (60)	122 (58)	528 (64)	603 (44)	2,068 (61)	<0.001
Pharmaceutical conspiracy	239 (29)	251 (45)	41 (49)	399 (54)	131 (56)	394 (52)	138 (60)	351 (71)	125 (58)	548 (64)	674 (46)	1,943 (57)	<0.001
Home-made recipes to make sanitizer products	230 (27)	308 (55)	51 (59)	522 (69)	158 (62)	399 (51)	149 (68)	367 (75)	104 (46)	499 (59)	692 (47)	2,095 (59)	<0.001
Alternative drugs/cure	240 (28)	297 (53)	48 (57)	533 (71)	168 (65)	529 (69)	125 (55)	319 (66)	105 (44)	507 (61)	686 (46)	2,185 (64)	<0.001
Fear toward products coming from infected countries	197 (25)	261 (46)	52 (62)	497 (67)	127 (52)	356 (46)	126 (55)	299 (59)	100 (46)	419 (51)	602 (44)	1,832 (51)	<0.001

Suppl. Table 37 Most prevalent topic areas with unclear or conflicting COVID-19 information, and most prevalent 'fake news', breakdown by country and self-reported level of understanding of COVID-19

H = high/very high/expert level; S = some; N = a little/none at all. Values in cells are n (weighted %) of respondents who replied 'yes'.

**Variable and categories**	**Thailand**			**Malaysia**			**UK**			**Italy**			**Slovenia**			**Total**			
**Self-reported level of understanding of COVID-19**	**H**	**S**	**N**	**H**	**S**	**N**	**H**	**S**	**N**	**H**	**S**	**N**	**H**	**S**	**N**	**H**	**S**	**N**	**P-value (for total)**
	N=965	N=459	N=52	N=435	N=359	N=33	N=647	N=336	N=26	N=368	N=324	N=20	N=713	N=279	N=42	N=3,128	N=1,757	N=173	
**Have you seen any unclear or conflicting information about COVID-19 in the last month?**																			
Ways to avoid the infection	401 (40)	145 (32)	18 (19)	197 (43)	191 (46)	21 (63)	416 (63)	248 (76)	15 (53)	202 (54)	193 (72)	15 (73)	445 (61)	211 (73)	26 (53)	1,661 (51)	988 (58)	95 (51)	0.094
Symptoms of COVID-19	400 (40)	150 (33)	18 (19)	170 (36)	167 (49)	16 (51)	363 (58)	210 (66)	17 (79)	147 (31)	163 (53)	18 (81)	312 (40)	164 (54)	18 (41)	1,392 (42)	854 (50)	87 (49)	0.026
What to do in case of symptoms	361 (37)	129 (30)	16 (17)	134 (34)	145 (41)	16 (39)	272 (39)	156 (49)	10 (59)	138 (34)	144 (55)	11 (49)	285 (37)	130 (52)	20 (40)	1,190 (37)	704 (44)	73 (37)	0.041
Social distancing guidance	349 (37)	124 (27)	17 (19)	132 (36)	144 (43)	16 (62)	355 (52)	199 (62)	14 (70)	163 (38)	140 (45)	11 (65)	362 (47)	170 (58)	27 (64)	1,361 (42)	777 (46)	85 (54)	0.168
Quarantine/isolation	379 (39)	139 (32)	11 (11)	153 (33)	145 (39)	16 (71)	338 (49)	193 (59)	16 (76)	148 (39)	135 (44)	9 (39)	372 (50)	165 (58)	22 (41)	1,390 (43)	777 (46)	74 (50)	0.397
Penalties if disobey restrictions	477 (49)	126 (28)	11 (11)	186 (35)	180 (46)	18 (56)	381 (54)	225 (68)	14 (66)	187 (47)	180 (56)	11 (69)	324 (44)	162 (48)	22 (53)	1,555 (47)	873 (48)	76 (47)	0.906
Risks in case of infection	381 (38)	132 (29)	14 (15)	152 (29)	158 (43)	17 (50)	337 (50)	191 (62)	14 (46)	158 (43)	156 (53)	16 (73)	312 (46)	159 (45)	22 (45)	1,340 (41)	796 (46)	83 (42)	0.343
Numbers of coronavirus cases/deaths related to COVID-19	416 (42)	134 (29)	13 (15)	129 (41)	137 (50)	18 (68)	463 (66)	261 (81)	17 (77)	233 (67)	214 (66)	10 (57)	284 (43)	156 (53)	23 (57)	1,525 (50)	902 (54)	81 (54)	0.276
Government support schemes (e.g. financial)	583 (60)	178 (38)	18 (20)	208 (46)	203 (61)	21 (62)	269 (40)	158 (53)	11 (56)	248 (67)	227 (71)	17 (78)	372 (48)	176 (59)	24 (48)	1,680 (52)	942 (55)	91 (50)	0.590
Testing	392 (39)	124 (29)	15 (15)	181 (36)	179 (46)	16 (32)	467 (70)	249 (74)	18 (77)	266 (71)	239 (71)	15 (86)	357 (48)	154 (55)	23 (31)	1,663 (50)	945 (53)	87 (39)	0.108
Travel restrictions (e.g. curfew, restricted hours of movement)	391 (39)	118 (25)	11 (11)	209 (37)	178 (46)	20 (62)	398 (60)	228 (71)	15 (52)	192 (50)	176 (58)	14 (78)	341 (43)	167 (50)	25 (41)	1,531 (44)	867 (49)	85 (47)	0.356

For peer review only - http://bmjopen.bmj.com/site/about/guidelines.xhtml

**Have you come across news about the following COVID-19 topics that seemed fake to you?**																			
General spread of fear	488 (47)	158 (36)	22 (23)	320 (65)	266 (80)	20 (56)	449 (70)	228 (73)	16 (81)	208 (57)	163 (59)	11 (61)	518 (71)	222 (65)	31 (66)	1,983 (61)	1,037 (60)	100 (54)	0.594
Coronavirus as an engineered modified virus	390 (37)	134 (26)	19 (19)	327 (71)	266 (62)	20 (46)	532 (83)	268 (79)	19 (70)	320 (87)	277 (80)	16 (60)	598 (80)	231 (65)	35 (75)	2,167 (66)	1,176 (60)	109 (49)	0.007
Minimisation of risks	305 (30)	120 (24)	15 (13)	222 (38)	176 (41)	18 (32)	377 (56)	191 (56)	11 (39)	277 (64)	249 (74)	14 (54)	510 (64)	196 (57)	25 (47)	1,691 (48)	932 (49)	83 (33)	0.063
Numbers of infected/deceased people	345 (34)	148 (33)	19 (18)	206 (49)	174 (48)	20 (39)	392 (58)	207 (66)	16 (75)	252 (76)	214 (75)	9 (63)	377 (51)	172 (62)	25 (61)	1,572 (49)	915 (55)	89 (45)	0.105
Unreasonable health recommendations	387 (36)	113 (26)	17 (17)	286 (54)	237 (53)	22 (63)	375 (55)	186 (58)	13 (71)	211 (57)	163 (44)	11 (54)	440 (59)	186 (65)	24 (48)	1,699 (50)	885 (47)	87 (50)	0.538
Pharmaceutical conspiracy	358 (36)	112 (25)	20 (21)	238 (53)	188 (48)	14 (38)	355 (55)	158 (51)	12 (56)	266 (69)	209 (57)	14 (65)	453 (61)	192 (61)	28 (45)	1,670 (52)	859 (46)	88 (40)	0.059
Home-made recipes to make sanitizer products	400 (38)	122 (24)	16 (15)	309 (62)	241 (62)	23 (57)	366 (56)	179 (55)	12 (68)	274 (78)	227 (62)	15 (71)	411 (52)	170 (51)	22 (45)	1,760 (52)	939 (49)	88 (48)	0.390
Alternative drugs/cure	409 (39)	112 (24)	16 (16)	305 (57)	257 (75)	19 (20)	468 (72)	214 (62)	15 (50)	243 (64)	188 (52)	13 (66)	430 (53)	159 (45)	23 (58)	1,855 (54)	930 (49)	86 (33)	0.004
Fear toward products coming from infected countries	330 (33)	109 (23)	19 (20)	297 (65)	234 (68)	18 (39)	317 (50)	155 (48)	11 (44)	226 (58)	187 (55)	12 (64)	352 (47)	145 (49)	22 (46)	1,522 (47)	830 (46)	82 (39)	0.456

Supplementary figure for "Economic and social impacts of COVID-19 and public health measures: results from an anonymous online survey in Thailand, Malaysia, the United Kingdom, Italy and Slovenia"

Anne Osterrieder[1,2], Giulia Cuman[3], Wirichada Pan-ngum[1,4], Phaik Kin Cheah[5], Phee-Kheng Cheah[6], Pimnara Peerawaranun[1], Margherita Silan[7], Miha Orazem[8,9], Ksenija Perkovic[10], Urh Groselj[8,11], Mira Leonie Schneiders[1,2,12], Tassawan Poomchaichote[1,13], Naomi Waithira[1,2], Supa-at Asarath[1], Bhensri Naemiratch[1], Supanat Ruangkajorn[1], Lenart Skof[14], Natinee Kulpijit[1], Constance R.S. Mackworth-Young[15], Darlene Ongkili[16], Rita Chanviriyavuth[1], Mavuto Mukaka[1,2], Phaik Yeong Cheah[1,2,12,,13]

[1]Mahidol Oxford Tropical Medicine Research Unit, Faculty of Tropical Medicine, Mahidol University, Bangkok, 10400, Thailand
[2]Centre for Tropical Medicine & Global Health, Nuffield Department of Medicine, University of Oxford, Oxford, UK
[3]Paediatric Ethics Committee; Research Ethics Committee, University Hospital of Padua, Padua, Italy
[4]Department of Tropical Hygiene, Faculty of Tropical Medicine, Mahidol University, Bangkok, 10400, Thailand
[5]Faculty of Arts and Social Science, Universiti Tunku Abdul Rahman, Kampar, Malaysia
[6]Emergency and Trauma Department, Sabah Women and Children's Hospital, Ministry of Health Malaysia, Kota Kinabalu, Malaysia
[7]Department of Statistical Sciences, University of Padua, Padua, Italy
[8]Faculty of Medicine, University of Ljubljana, Ljubljana, Slovenia
[9]Department of Radiation Oncology, Institute of Oncology Ljubljana, Ljubljana, Slovenia
[10]Institute for Social Studies, Science and Research Centre Koper, Koper, Slovenia
[11]Department of Endocrinology, Diabetes and Metabolic Diseases, University Children's Hospital, University Medical Center, University Children's Hospital Ljubljana, Ljubljana, Slovenia
[12]Ethox Centre, Nuffield Department of Population Health, University of Oxford, Oxford, UK
[13]The SoNAR-Global Network
[14]Institute for Philosophical Studies, Science and Research Centre Koper, Koper, Slovenia
[15]Department of Global Health and Development, London School of Hygiene and Tropical Medicine, London, UK
[16]Emergency and Trauma Department, Queen Elizabeth Hospital, Ministry of Health Malaysia, Kota Kinabalu, Malaysia

Corresponding author: Phaik Yeong Cheah, 420/6 Mahidol-Oxford Tropical Medicine 24 Research Unit, Faculty of Tropical Medicine, Rajvithi Road, Bangkok, Thailand, 10400. Email: phaikyeong@tropmedres.ac

Supplementary Figure 1: Diagram showing the level of coordination of public information campaigns on COVID-19 in the five study countries.

Levels of coordination: 0 = no COVID-19 public information campaign; 1 = public officials urging caution about COVID-19; 2 = coordinated public information campaign (e.g. across traditional and social media). All countries ran public information campaigns at level 2 during the study period from 1st May to 30th June 2020. Data was provided by the Oxford COVID-19 Government Response Tracker[32] and downloaded from 'Our World in Data'[33].

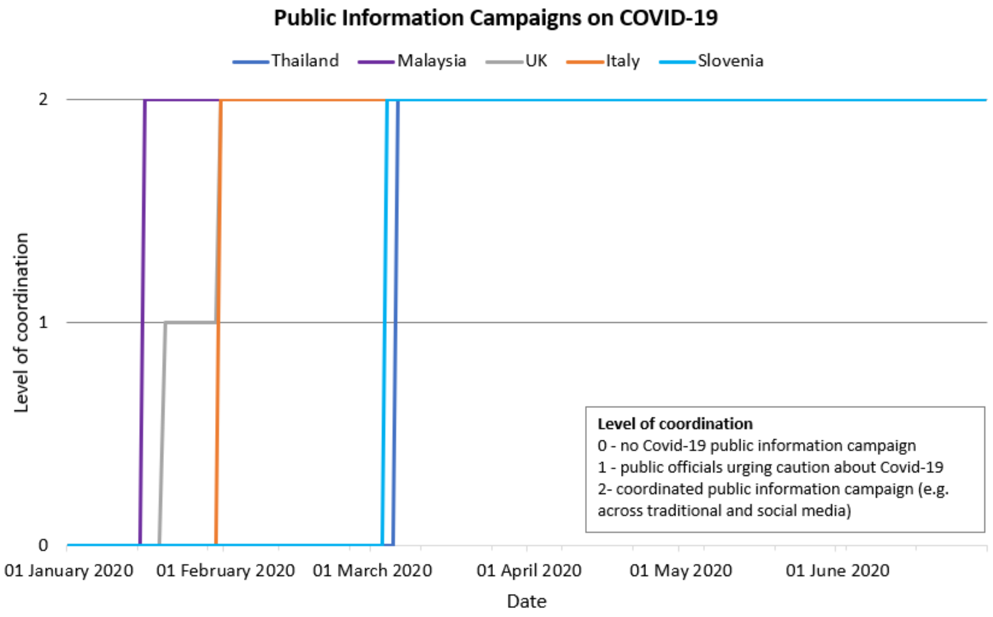

STROBE 2007 (v4) checklist of items to be included in reports of observational studies in epidemiology*

Checklist for cohort, case-control, and cross-sectional studies (combined)

Section/Topic	Item #	Recommendation	Reported on page #
**Title and abstract**	1	(*a*) Indicate the study's design with a commonly used term in the title or the abstract	2
		(*b*) Provide in the abstract an informative and balanced summary of what was done and what was found	2
**Introduction**			
Background/rationale	2	Explain the scientific background and rationale for the investigation being reported	3
Objectives	3	State specific objectives, including any pre-specified hypotheses	4
**Methods**			
Study design	4	Present key elements of study design early in the paper	4
Setting	5	Describe the setting, locations, and relevant dates, including periods of recruitment, exposure, follow-up, and data collection	4,5
Participants	6	(*a*) *Cohort study*—Give the eligibility criteria, and the sources and methods of selection of participants. Describe methods of follow-up<br>*Case-control study*—Give the eligibility criteria, and the sources and methods of case ascertainment and control selection. Give the rationale for the choice of cases and controls<br>*Cross-sectional study*—Give the eligibility criteria, and the sources and methods of selection of participants	This is a survey<br>5
		(*b*) *Cohort study*—For matched studies, give matching criteria and number of exposed and unexposed<br>*Case-control study*—For matched studies, give matching criteria and the number of controls per case	NA
Variables	7	Clearly define all outcomes, exposures, predictors, potential confounders, and effect modifiers. Give diagnostic criteria, if applicable	4
Data sources/ measurement	8*	For each variable of interest, give sources of data and details of methods of assessment (measurement). Describe comparability of assessment methods if there is more than one group	4
Bias	9	Describe any efforts to address potential sources of bias	6
Study size	10	Explain how the study size was arrived at	6
Quantitative variables	11	Explain how quantitative variables were handled in the analyses. If applicable, describe which groupings were chosen and why	6
Statistical methods	12	(*a*) Describe all statistical methods, including those used to control for confounding	6
		(*b*) Describe any methods used to examine subgroups and interactions	6
		(*c*) Explain how missing data were addressed	No missing data. only completed surveys can be submitted

For peer review only

		(*d*) *Cohort study*—If applicable, explain how loss to follow-up was addressed<br>*Case-control study*—If applicable, explain how matching of cases and controls was addressed<br>*Cross-sectional study*—If applicable, describe analytical methods taking account of sampling strategy	NA
		(*e*) Describe any sensitivity analyses	NA
**Results**			
Participants	13*	(a) Report numbers of individuals at each stage of study—eg numbers potentially eligible, examined for eligibility, confirmed eligible, included in the study, completing follow-up, and analysed	6
		(b) Give reasons for non-participation at each stage	5
		(c) Consider use of a flow diagram	NA
Descriptive data	14*	(a) Give characteristics of study participants (eg demographic, clinical, social) and information on exposures and potential confounders	6
		(b) Indicate number of participants with missing data for each variable of interest	NA
		(c) *Cohort study*—Summarise follow-up time (eg, average and total amount)	NA
Outcome data	15*	*Cohort study*—Report numbers of outcome events or summary measures over time	NA
		Case-control study—Report numbers in each exposure category, or summary measures of exposure	NA
		Cross-sectional study—Report numbers of outcome events or summary measures	NA
Main results	16	(*a*) Give unadjusted estimates and, if applicable, confounder-adjusted estimates and their precision (eg, 95% confidence interval). Make clear which confounders were adjusted for and why they were included	7-12
		(*b*) Report category boundaries when continuous variables were categorized	7-12
		(*c*) If relevant, consider translating estimates of relative risk into absolute risk for a meaningful time period	NA
Other analyses	17	Report other analyses done—eg analyses of subgroups and interactions, and sensitivity analyses	7-12
**Discussion**			
Key results	18	Summarise key results with reference to study objectives	12
Limitations	19	Discuss limitations of the study, taking into account sources of potential bias or imprecision. Discuss both direction and magnitude of any potential bias	14-15
Interpretation	20	Give a cautious overall interpretation of results considering objectives, limitations, multiplicity of analyses, results from similar studies, and other relevant evidence	12-14
Generalisability	21	Discuss the generalisability (external validity) of the study results	14-15
**Other information**			
Funding	22	Give the source of funding and the role of the funders for the present study and, if applicable, for the original study on which the present article is based	16

*Give information separately for cases and controls in case-control studies and, if applicable, for exposed and unexposed groups in cohort and cross-sectional studies.

For peer review only

Note: An Explanation and Elaboration article discusses each checklist item and gives methodological background and published examples of transparent reporting. The STROBE checklist is best used in conjunction with this article (freely available on the Web sites of PLoS Medicine at http://www.plosmedicine.org/, Annals of Internal Medicine at http://www.annals.org/, and Epidemiology at http://www.epidem.com/). Information on the STROBE Initiative is available at www.strobe-statement.org.

For peer review only
